# Know Your Heart: Rationale, design and conduct of a cross-sectional study of cardiovascular structure, function and risk factors in 4500 men and women aged 35-69 years from two Russian cities, 2015-18

**DOI:** 10.12688/wellcomeopenres.14619.3

**Published:** 2018-12-03

**Authors:** Sarah Cook, Sofia Malyutina, Alexander V Kudryavtsev, Maria Averina, Natalia Bobrova, Sergey Boytsov, Soren Brage, Taane G. Clark, Ernest Diez Benavente, Anne Elise Eggen, Laila A Hopstock, Alun Hughes, Heidi Johansen, Kamila Kholmatova, Anastasiya Kichigina, Anna Kontsevaya, Michael Kornev, Darryl Leong, Per Magnus, Ellisiv Mathiesen, Martin McKee, Katy Morgan, Odd Nilssen, Ilya Plakhov, Jennifer K Quint, Alicja Rapala, Andrey Ryabikov, Lyudmila Saburova, Henrik Schirmer, Marina Shapkina, Suhail Shiekh, Vladimir M Shkolnikov, Michael Stylidis, Michael Voevoda, Kate Westgate, David A Leon

**Affiliations:** 1 London School of Hygiene & Tropical Medicine, London, WC1E 7HT, UK; 2 Research Institute of Internal and Preventive Medicine, Branch of Institute of Cytology and Genetics, Siberian Branch of the Russian Academy of Sciences, Novosibirsk, 630090, Russian Federation; 3 Novosibirsk State Medical University, Russian Ministry of Health, Novosibirsk, 630091, Russian Federation; 4 Northern State Medical University, Arkhangelsk, 163000, Russian Federation; 5 UiT the Arctic University of Norway, Tromsø, 9037, Norway; 6 Federal State budget organization, National medical research center of cardiology, Russian Ministry of Health, Moscow, 121552, Russian Federation; 7 MRC Epidemiology Unit, School of Clinical Medicine, University of Cambridge, Cambridge, CB2 0QQ, UK; 8 UCL Institute of Cardiovascular Science, University College London, London, WC1E 6BT, UK; 9 National research center for preventive medicine, Moscow, 101990, Russian Federation; 10 McMaster University , Ontario, L8S 4K1, Canada; 11 Centre for Fertility and Health, Norwegian Institute of Public Health, Oslo, 0851, Norway; 12 Lytech Laboratory LLC, Moscow, 107023, Russian Federation; 13 Royal Brompton Campus, Imperial College London, London, SW3 6LY, UK; 14 Institute of Philosophy and Law, Ural Branch of the Russian Academy of Sciences, Ekaterinburg, 620990, Russian Federation; 15 Akerhus University Hospital, Oslo, 1478, Norway; 16 Max Planck Institute for Demographic Research, Rostock, 18057, Germany; 17 Higher School for Economics, National Research University , Moscow, 101000, Russian Federation

**Keywords:** Russian Federation, cardiovascular disease, cross-sectional study, epidemiology, international comparison

## Abstract

Russia has one of the highest rates of cardiovascular disease in the world. The International Project on Cardiovascular Disease in Russia (IPCDR) was set up to understand the reasons for this. A substantial component of this study was the Know Your Heart Study devoted to characterising the nature and causes of cardiovascular disease in Russia by conducting large cross-sectional surveys in two Russian cities Novosibirsk and Arkhangelsk. The study population was 4542 men and women aged 35-69 years recruited from the general population. Fieldwork took place between 2015-18. There were two study components: 1) a baseline interview to collect information on socio-demographic characteristics and cardiovascular risk factors, usually conducted at home, and 2) a comprehensive health check at a primary care clinic which included detailed examination of the cardiovascular system. In this paper we describe in detail the rationale for, design and conduct of these studies.

## Introduction

Russia has one of the highest rates of mortality from cardiovascular disease (CVD) in the world (see non-communicable disease mortality data from the World Health Organisation (WHO)), despite an ongoing pattern of decline that began in 2005. In 2015 the CVD mortality rate was four times higher in Russia than in England and Wales or Norway (see Human Cause-of-Death Database and WHO mortality database). These exceptional CVD mortality rates are an important reason for the lower life expectancy in Russia compared to other industrial countries (70.9 years in 2014; see The Demographic Yearbook of Russia 2015).

CVD mortality in Russia has a number of specific features that pose a challenge to our understanding. In most countries, the risk of death from CVD correlates well with levels of established risk factors such as smoking, serum cholesterol, blood pressure and obesity ^1^. However in Russia, while some of the risk of CVD death is explained by conventional risk factors such as smoking (in men) and a high prevalence of uncontrolled hypertension, some aspects of the cardiovascular risk profile of the population do not appear to be high risk ^1, 2^. Lipid profiles appear to be particularly surprising. Previous studies dating from 1975–2000 have tended to find relatively low risk lipid profiles in Russia compared to Western countries, with unexceptional low density lipoprotein (LDL) cholesterol, higher levels of high density lipoprotein (HDL) ^3^ cholesterol and more favourable ratios of ApoB/A1 ^4^ or HDL/total cholesterol ^2, 5^.

One specific and highly distinctive feature of CVD mortality in Russia, that it shares with several other countries that were previously part of the Soviet Union, is that it has shown dramatic fluctuations over the past 30 years. Remarkably, these fluctuations parallel those from rates of mortality from acute alcohol poisoning ^6^. This suggests that hazardous alcohol drinking in Russia over this period has been one of the main drivers of fluctuations in CVD deaths ^7^. However the mechanisms underlying this association have not been identified and contrast with the dominant literature on alcohol and CVD that has in the past tended to be preoccupied with the apparent cardio-protective effects of moderate drinking ^8^.

The International Project on Cardiovascular Disease in Russia (IPCDR) was set up to throw new light on the high rates of premature mortality from cardiovascular disease in Russia. The project has four separate but inter-related themes. These are: 1) investigating the extent to which the differences between Russia and other countries in CVD mortality rates may be biased because of differences in the way in which deaths are certified and coded; 2) generating improved overviews of trends and differences on CVD mortality and established risk factors in Russia by bringing together and synthesising already collected data; 3) examining the potential role of the health-care system and treatment in contributing to the trends in CVD rates within Russia and to differences with other countries; 4) characterising the nature and causes of cardiovascular disease in Russia by conducting large cross-sectional surveys in two Russian cities Novosibirsk and Arkhangelsk. This paper describes in detail the rationale, objectives, design and conduct of these cross-sectional studies that are collectively known in Russia as “Узнай своё сердце” (Know Your Heart).

### Rationale

To help uncover the nature and causes of the higher CVD mortality in Russia today compared to other countries, it is desirable to be able to compare the cardiovascular health of a random cross-sectional sample of the Russian population with data from an equivalent sample from a country with much lower CVD mortality (such as Norway). In this context, cardiovascular health refers to objectively measured aspects of the structure and function of the cardiovascular system (such as echocardiography derived indices), blood and urine derived biomarkers and behavioural risk factors. This detailed information may be thought of as the cardiovascular phenotypic profile of a population. The assumption underlying this approach is that the future CVD event rates in the surveyed populations in Russia will be appreciably higher than the event rates found in the population surveyed in the lower mortality country. If this is true, then these future differences should be prefigured in differences in the cardiovascular phenotypic profile. Identifying the principle differences in the phenotype between Russia and a lower mortality country will throw light on the drivers of these differences. Aside from the international comparisons, information on the cardiovascular phenotype of a sample of the Russian population today will also be valuable for understanding the distribution and determinants of CVD within Russia, including socio-economic differences, use of health systems, treatment and the potential role of particular risk factors including alcohol.

## Protocol

### Objectives

The objectives of the cross-sectional studies conducted as part of the fourth component of the IPCDR study were as follows:

1) To characterise the CVD phenotypes of the Russian population samples, including in depth objective measures of cardiac and vascular structure and function, laboratory-derived biomarkers from biological samples and behaviours including risk factors as well as health service use;
2) Determine the extent to which the CVD phenotypes in two Russian cities, Arkhangelsk and Novosibirsk differ from those seen in other countries, and to identify whether any such differences may plausibly explain the excess of CVD mortality seen in Russia. In particular comparisons will be made with the 7th wave of the Tromsø Study in Norway conducted in 2015–16. Key aspects of the protocol of the medical examination were aligned in order to be able to make direct comparisons. These comparisons are being taken forward under the “ *Heart to Heart*” initiative established jointly with UiT, The Arctic University of Norway.
3) Investigate the associations of these CVD phenotypes with socio-demographic factors, health behaviours including alcohol use and known cardiovascular risk factors within Russia in order to improve understanding of the determinants of these phenotypes;
4) Undertake exploratory studies of the association of gut microbiota with behaviours (especially heavy drinking) and the CVD phenotypes.

**Figure 1.**
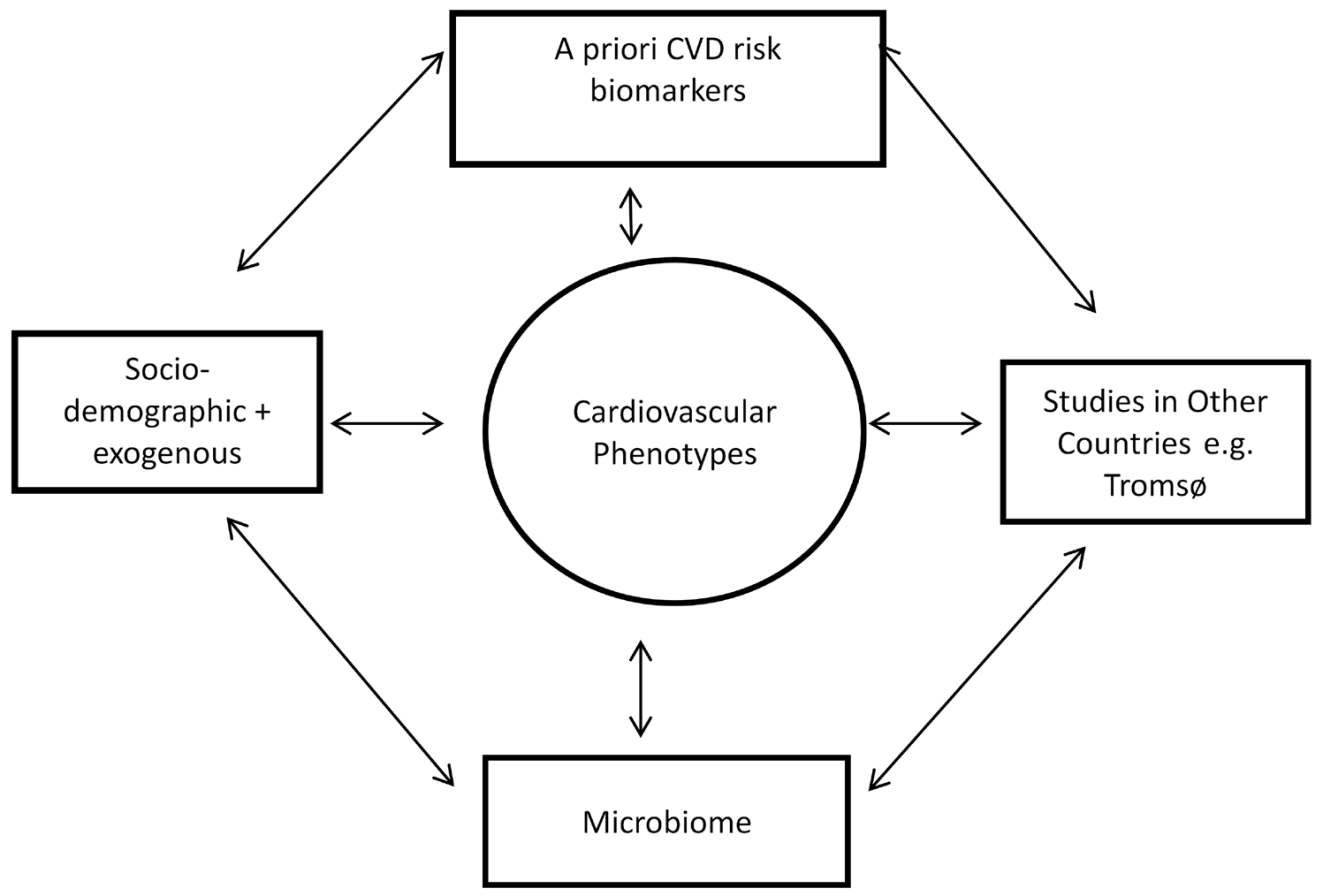
Key associations and comparisons of interest.

The key associations and comparisons of interest are shown in Figure 1. Examples of the types of data collected on cardiovascular phenotype are shown in Table 1.

**Table 1.** Examples of data available on different aspects of cardiovascular phenotype.

Cardiovascular phenotypes		
Type	Source	Biomarker/proxy measure
Arteriosclerosis/ Atherosclerosis	Questionnaire	Previous Myocardial Infarction
	ECG	Evidence of previous Myocardial Infarction
	Carotid ultrasound	Carotid Intima Media Thickness, plaque
	Vicorder	Pulse wave velocity
Cardiac remodelling	Blood samples	B-type natriuretic peptide, High sensitivity Troponin T
	Echocardiography	Myocardial function and size
Arrhythmia	ECG	Baseline rhythm

### Sample size calculation

The original target sample size was determined based on both the power needed to make comparisons with other population based studies and to investigate associations of interest within the Know Your Heart Study. For example, if we wished to compare the prevalence of a binary ECHO phenotype between Know Your Heart (N=4500) with a smaller study with data available on this phenotype for N=1500 (e.g. the UK 1946 National Birth Cohort study) we would have 80% power to detect an odds ratio of 1.4 significant at an alpha of 0.01 assuming a prevalence in the smaller study of 10%. Comparisons with the larger Tromsø 7 study will be even more powerful. Within the Know Your Heart Study we estimate that we will have 80% power to detect an OR of 1.6 or more between the top and bottom 20% of a continuous exposure variable (e.g. levels of a particular lipoprotein entity) and a binary CVD phenotype with a prevalence of 10% in the lowest group, that is significant with an alpha of 0.01. We are aware that applying many statistical tests can lead to false-positive correlations / associations, and we propose to apply stringent significance cut-offs (less than the nominal 0.05) to be determined through data simulation (e.g. permutation), complemented by a false discovery rate approach. While these sample size calculation are based on estimates for a range of plausible scenarios it should be noted that the study may be under powered for the investigation of some associations of interest.

### Target population and study setting

We undertook identical cross-sectional studies of clinical and life style factors in two Russian cities (Arkhangelsk and Novosibirsk) in the period 2015–18 with a target sample size of 4500 adults. These cities were chosen as they had a previous track-record of conducting large population-based epidemiological surveys and thus could be expected to conduct complex research to a high standard ^2, 9– 12^. The target population was men and women aged 35–69 years. This is the age group in which in relative terms mortality from cardiovascular disease and many other conditions is much higher than in Western countries.

**Figure 2.**
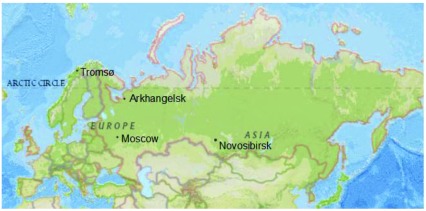
Location of Arkhangelsk, Novosibirsk and Tromsø.

The location of the cities is shown in Figure 2. The city of Novosibirsk, in Western Siberia, has a population of more than 1,500,000 people and is the third largest city in Russia, after Moscow and Saint Petersburg. Arkhangelsk, located in the North of European Russia, is a smaller city with a population of about 350,000 people. Levels of cardiovascular mortality vary across Russia. In the period 2012–16, mortality from total circulatory disease at ages 35–69 years among the urban population of Novosibirsk region was slightly lower than the national average, while in the urban population of Arkhangelsk region it was above the national average ( Table 2). Mortality from ischaemic heart disease was above the national average in both cities. Mortality rates from total circulatory disease and ischaemic heart were considerably higher in Russia and in both of the Russian cities compared to Tromsø and Norway overall.

**Table 2.** Mortality rates by sex and cause (age standardized/100,000) for Russia and the urban populations of Arkhangelsk and Novosibirsk oblasts and Norway as a whole and the municipality of Tromsø, Norway, aged 35–69 for years 2012–16.

Cause of death	Men					Women				
	Russia	Arkhangelsk (urban)	Novosibirsk (urban)	Norway	Tromsø *	Russia	Arkhangelsk (urban)	Novosibirsk (urban)	Norway	Tromsø *
All circulatory diseases **	735	821	711	89	*75*	239	245	236	33	*19*
Ischaemic heart disease ***	407	517	447	49	*36*	111	125	124	12	*7*
All causes	1755	1852	1772	410	*393*	619	612	610	267	*157*

Notes:

Rates age standardized to 1976 Standard European Population

Data for Russia, Arkhangelsk and Novosibirsk from the Russian Fertility and Mortality database of the Centre of Demographic Research of the New Economic School http://www.demogr.nes.ru/index.php/en/demogr_indicat/data

Data for Norway and Tromsø provided by Section of Health Data and Digitalisation, Norwegian Institute of Public Health

* Rates for municipality of Tromsø (90% of population living in the urban area of the city) are based on only 318 all cause deaths for men and 184 all cause deaths for women. Numbers of deaths from IHD are 30 for men and 8 for women. To indicate their associated imprecision they are shown in italics.

** ICD 10 codes I00-I99 (Diseases of the circulatory system)

*** ICD 10 codes I20-I25 (Ischemic Heart Diseases)

**Figure 3.**
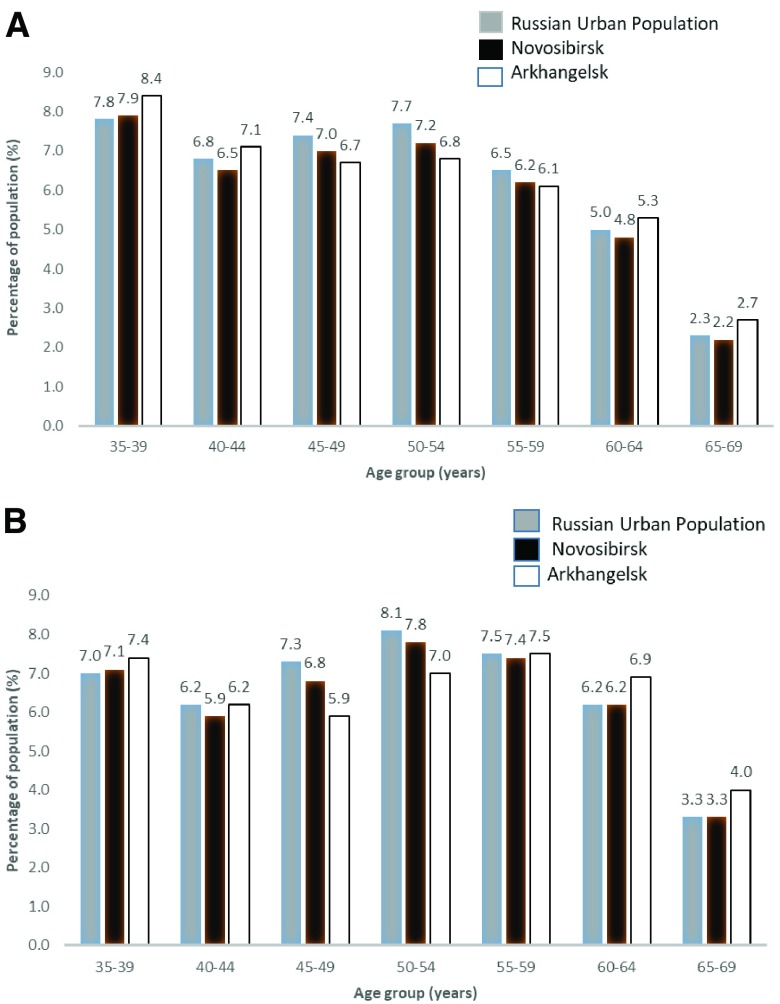
Age profile of Novosibirsk and Arkhangelsk compared to the Russian Urban Population from the 2010 Russian census for men ( **a**) and women ( **b**).

**Figure 4.**
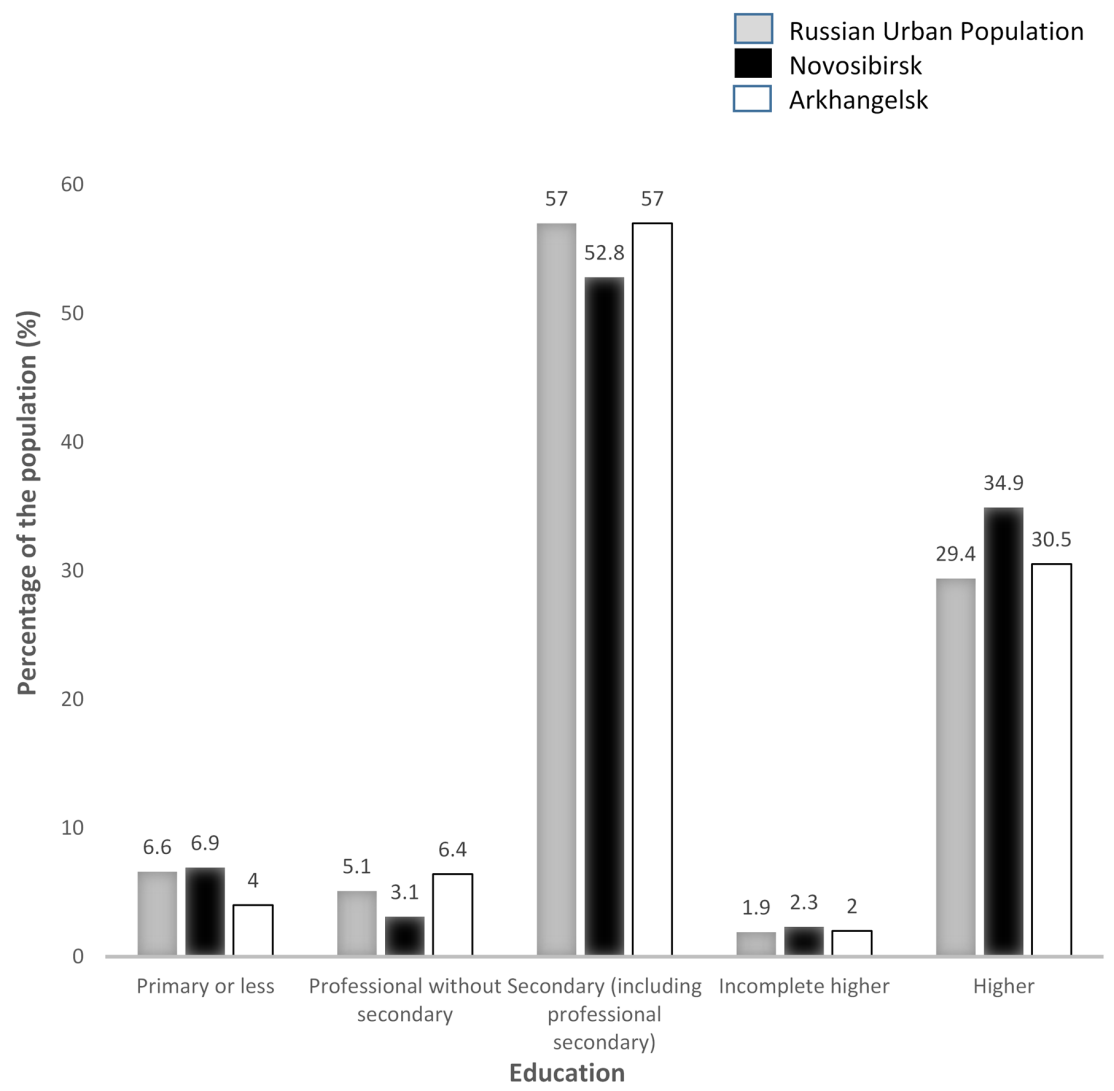
Educational Profile of Novosibirsk and Arkhangelsk (35–69 years) compared to the Russian urban population from the 2010 Russian census.

The age and education distribution of the populations of Novosibirsk and Arkhangelsk compared to the total Russian urban population, according to 2010 census data, are shown in Figure 3 and Figure 4. The age distribution was similar to the national average in both cities but the proportion of people with higher education was higher in Novosibirsk.

### Study design

**Figure 5.**
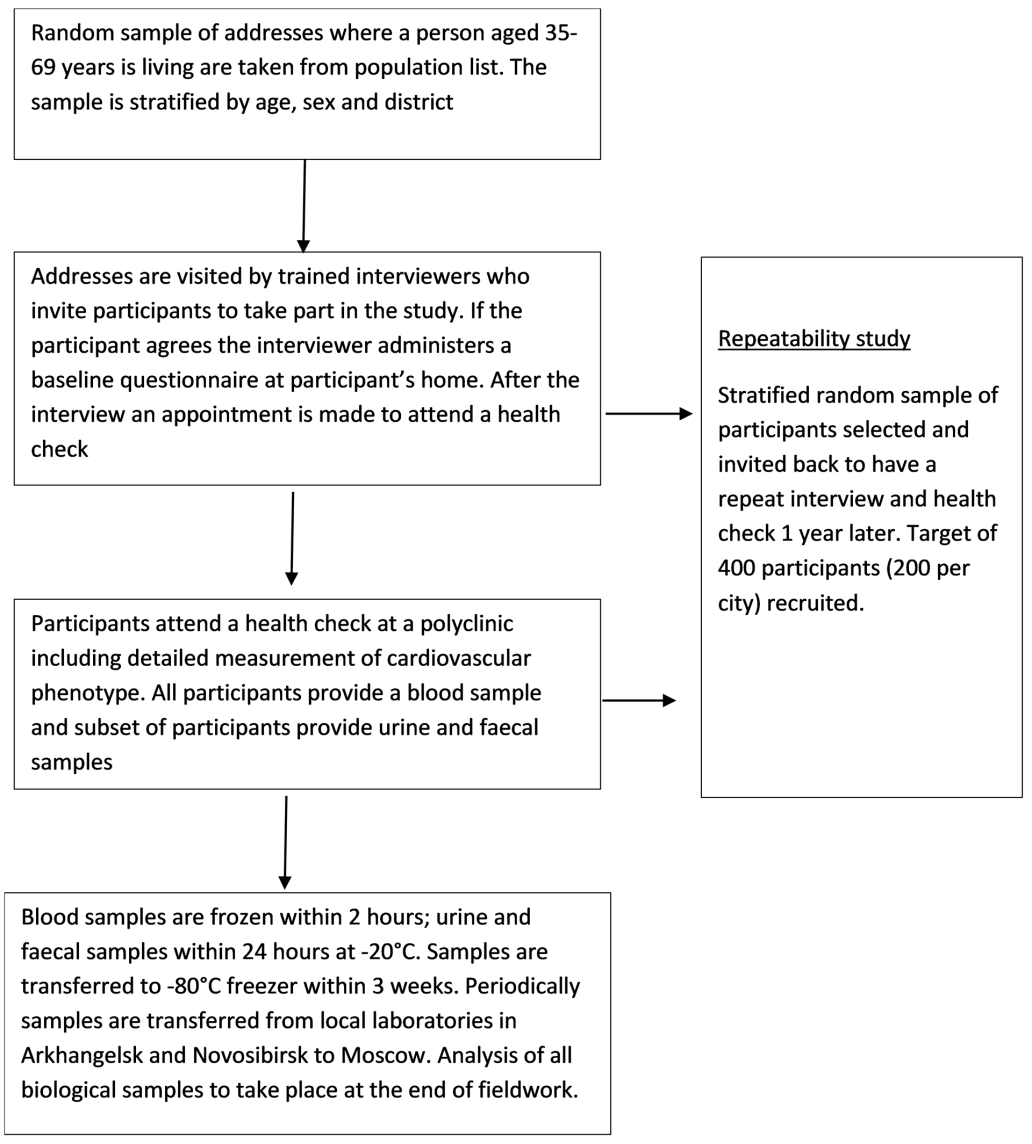
Study Design (Main population survey).

The study had two components: 1) a baseline interview to collect information on socio-demographic characteristics and cardiovascular risk factors, usually conducted at home, and 2) a subsequent comprehensive health check at a primary care clinic (polyclinic) which included examination of the cardiovascular system. An overview of the study design is shown in Figure 5.

***Recruitment of Participants from the general population***. Within each city four districts were selected for the recruitment of participants. In Arkhangelsk these were Lomonosovsky, Maymaksansky, Mayskaya Gorka and Oktyabrsky. In Novosibirsk these were Dzerzinsky, Kirovsky, Leninsky and Oktyabrsky. The districts were selected purposefully (not through random sampling) to represent a range of socio-demographic and mortality levels in each city. A sampling frame of people within each district using information on age and sex of occupants at individual addresses was provided by the regional health insurance funds. Because of data protection regulations, the study team was not provided with individual names. From the sampling frames we selected at random addresses to visit stratified by age (in 5-year bands), sex and district. The aim was to recruit equal numbers of participants in each sex and 5-year age group in the city as a whole.

Participants were recruited to the study by home visits carried out by trained and experienced interviewers from a local commercial survey company. They attempted to identify a person of the correct age and sex who, according to the sampling frame, should be living at the selected addresses. If the participant was not available on the first visit addresses were visited a minimum of two more times at varying times of day and at weekends. At the end of a successful interview participants were invited to attend the health check at a polyclinic and if they agreed an appointment was made for them straight away using an online calendar.

To maximize the probability of participants agreeing to take part in the study information campaigns were conducted in both cities. The campaigns were implemented on the assumption that if people had previously heard that the study was legitimate and important through the media they would be more likely to participate. Special consideration was given to the name of the study used for participants “Know your heart” (in Russian “Узнай своё сердце”), the study logo and the visual style of study materials. We used focus groups with the general public to guide the final design. The information campaign included production of two short films about the study (one for each city) which were shown regularly on TV throughout the period of the study ( Supplementary File S1). In addition, news items about the study progress, and the experience of participants who had taken part in the study were periodically disseminated on TV, radio and in print media. Large bill board advertisements about the study were also placed on rotation throughout the city at bus stops, super markets and areas where advertisements were concentrated in the city ( Supplementary File S2). These activities were more intensive and consistent in Arkhangelsk than in Novosibirsk.

Recruitment of participants from the general population started in November 2015 and finished in December 2017. Recruitment paused at Christmas and over the summer (July and August) in keeping with Russian holidays when participants were likely to leave the city.

***Recruitment of participants receiving treatment for alcohol problems***. Given the potential importance of hazardous alcohol use as a risk factor for cardiovascular disease in Russia, an additional 275 participants aged 35–69 years with a primary diagnosis of alcohol problems were recruited from Arkhangelsk Regional Psychiatric hospital. Where possible, participants were recruited from the same four districts of the city as the general population sample. By using a clinical facility as a source of participants we were aware that we would be recruiting a highly selected group of heavy drinkers. However, our aim was to be able to characterize the cardiovascular phenotype in a group of heavy drinkers per se.

Participants were recruited by clinical staff at the hospital at least one week after admission in order to ensure that the acute detoxification stage of treatment was complete and they were not suffering from alcohol withdrawal. The same interviewers involved in the general population study visited participants at the hospital and administered a shortened version of the baseline questionnaire with some supplementary questions on alcohol use included to obtain more detailed characterization of drinking behaviour in this sub-group ( Supplementary File 3). The day after their interview, participants were provided with free transport to attend the health check. The health check took place in the same polyclinic as for the general population survey, but to avoid placing an excessive burden on the participants, the health check itself was shortened by dropping a few of the more onerous aspects of the examination: pulse wave velocity, physical function tests, spirometry, and use of the Actiheart devices to measure physical activity continuously over a period of days.

Recruitment of and examination of participants for this sub-study began in January 2017 and ended in October 2017.

***Repeatability study***. In each city approximately 200 participants from the general population sample (397 participants in total) were re-interviewed and had a repeated health check one year after their initial health check. The main aim was to estimate correction factors that can be used to correct for measurement error during the analysis stage, specifically when regressing an outcome on a single continuous predictor variable that is measured with error (i.e. to correct for regression dilution bias). The time period of one year was chosen to minimize the effects of seasonal variation on within-person variability. A secondary aim of the repeatability study was to investigate reproducibility of those characteristics that by definition should not change, such as educational level, whether an ever smoker and drinker, and so on.

### Fieldwork outcomes

***General population sample***. The study recruited 5089 participants for the baseline interview of whom 4542 participants went on to attend a health check. Of these 4542 participants, 2381 were from Arkhangelsk (41.5% male) and 2161 were from Novosibirsk (42.0% male). The median age of participants from Arkhangelsk was 54 years (IQR 45–62) and from Novosibirsk 56 years (IQR 47–64) with a higher percentage of participants in the older age categories in Novosibirsk than Arkhangelsk.

Response percentages were calculated from individual level data on the outcome of every visit made to each address. A list of the codes used to classify the outcome of the visits is provided in Supplementary Table S1 ( Supplementary File 4). Three types of response percentages were defined based on the denominator used:

 Type 1: The denominator was all households in the sampling frame where an attempt was made to contact a participant. This is the most conservative estimation of response percentage.

Type 2: The denominator excluded addresses which were found to be invalid or where no participant of the correct age or sex was living. These exclusions are largely accounted for by the original sampling frame being out of date or inaccurate.

Type 3: The denominator was restricted to addresses where it was determined that an eligible participant of the correct age and sex lived there. This response percentage reflected the willingness and ability of households to engage and the skill of the interviewer in motivating them to do so. The primary reason for non-response here was mainly a refusal to take part.

The response percentages with respect to obtaining a baseline interview for each city by age and sex are shown in Table 3. The overall response percentages for both cities were: Type 1 28.1% Type 2 35.1% and Type 3 51.0%. For all types, percentages were higher in Arkhangelsk than Novosibirsk, in women compared to men, and among older compared to younger participants.

**Table 3.** Baseline interview response percentages by age, sex, and city.

Type of response percentage *	Age group **	Arkhangelsk						Novosibirsk					
		Men		Women		Total		Men		Women		Total	
		Number interviewed	Response %	Number interviewed	Response %	Number interviewed	Response %	Number interviewed	Response %	Number interviewed	Response %	Number interviewed	Response %
Response type 1	35–39	95	20.9	144	34.6	239	27.4	90	8.8	136	14.6	226	11.6
	40–44	141	29.0	218	46.0	359	37.4	112	11.1	173	17.8	285	14.4
	45–49	145	32.9	195	47.2	340	39.8	155	15.4	194	20.5	349	17.9
	50–54	164	40.2	203	52.7	367	46.3	145	16.2	221	29.5	366	22.3
	55–59	159	43.0	222	58.3	381	50.7	166	17.7	216	30.5	382	23.2
	60–64	169	50.3	208	58.4	377	54.5	236	25.8	246	35.3	482	29.9
	65–69	158	50.6	259	62.9	417	57.6	239	25.2	280	38.6	519	31.0
	All ages	1031	36.7	1449	51.0	2480	43.9	1143	17.0	1466	25.6	2609	20.9
Response type 2	35–39	95	27.1	144	42.6	239	34.7	90	12.1	136	18.7	226	15.3
	40–44	141	36.3	218	54.0	359	45.3	112	15.0	173	22.6	285	18.9
	45–49	145	41.4	195	56.2	340	48.8	155	20.6	194	26.6	349	23.6
	50–54	164	50.8	20	62.5	367	56.6	145	20.9	221	35.1	366	27.6
	55–59	159	50.0	222	67.3	381	58.8	166	21.8	216	36.5	382	28.2
	60–64	169	59.1	208	66.2	377	58.2	236	31.5	246	39.7	482	35.2
	65–69	158	65.6	259	73.2	417	69.5	239	33.0	280	47.1	519	39.4
	All ages	1031	45.7	1449	60.1	2480	53.1	1143	22.1	1466	31.5	2609	26.5
Response type 3	35–39	95	44.6	144	63.2	239	54.2	90	21.2	136	32.2	226	26.3
	40–44	141	52.2	218	72.7	359	63.0	112	25.7	173	36.7	285	31.3
	45–49	145	58.2	195	71.7	340	65.3	155	35.5	194	44.3	349	39.3
	50–54	164	64.3	20	78.4	367	71.4	145	34.0	221	53.5	366	43.2
	55–59	159	60.2	222	77.6	381	69.3	166	37.6	216	52.8	382	44.4
	60–64	169	69.0	208	76.8	377	73.1	236	44.5	246	55.9	482	49.5
	65–69	158	75.2	259	83.0	417	79.9	239	45.0	280	60.5	519	51.6
	All ages	1031	60.4	1449	75.2	2480	68.2	1143	35.4	1466	48.0	2609	41.1

* Response type 1 denominator is total number of potential participants whose address was issued to interviewers. Type 2 denominator excluded addresses that could not be found or where no one of expected age and sex was found. Type 3 denominator restricted to those addresses where it was established that person of expected age and sex was resident. Further details can be found in Supplementary Table S1.

** Age self-reported at baseline interview or where participant was not interviewed age defined using expected age of participant at address from sampling frame

One way of judging the extent of sampling bias introduced by non-response is to compare the educational distribution of those with a baseline interview and health check with the educational distribution for each city as determined at the 2010 Russian Census. Table 4 shows the observed distribution against the expected distribution from the Census distribution using indirect standardisation for age and sex for both completing the baseline interview and attending the health check. For Arkhangelsk the ratio overall for completing baseline questionnaire was 0.98 and that for attending the health check was 0.99. However, younger participants were more likely than expected to have higher education and older participants were less likely than expected to have higher education. For Novosibirsk the ratio of observed to expected education was above 1 for both completion of the baseline interview (1.14) and attending the health check (1.26).

**Table 4.** Ratio of observed to expected (based on 2010 census) participants with higher education by age.

Age group	Arkhangelsk Interviewed		Arkhangelsk Health Check		Novosibirsk Interviewed		Novosibirsk Health Check	
	Ratio	95% CI	Ratio	95% CI	Ratio	95% CI	Ratio	95% CI
35–39	1.32	1.10, 1.57	1.35	1.12, 1.61	1.28	1.07, 1.53	1.43	1.15, 1.75
40–44	1.30	1.11, 1.51	1.32	1.12, 1.53	1.00	0.83, 1.20	1.12	0.91, 1.36
45–49	1.26	1.06, 1.48	1.28	1.08, 1.50	1.17	1.00, 1.37	1.29	1.08, 1.53
50–54	1.07	0.90, 1.27	1.11	0.93, 1.32	1.35	1.15, 1.57	1.42	1.19 1.68
55–59	0.82	0.67, 0.99	0.82	0.68, 0.99	1.14	0.96,1.35	1.13	0.93, 1.36
60–64	0.66	0.54, 0.81	0.66	0.53, 0.80	1.02	0.87,1.19	1.07	0.89, 1.27
65–69	0.70	0.58, 0.83	0.71	0.59, 0.84	1.08	0.93, 1.26	1.38	1.18, 1.60
All ages	0.98	0.92, 1.04	0.99	0.93, 1.06	1.14	1.07, 1.21	1.26	1.17, 1.34

Not everybody who had a baseline interview had a subsequent health check. Some people elected not to have one, while others were unable to arrange a suitable time or failed to attend at an arranged time. These proportions varied by city, with 96% attending in Arkhangelsk, but only 83% in Novosibirsk. The proportions of interviewed participants by age and sex for each city are shown in Table 5. The response percentages with respect to health check attendance using the three types of response are shown in Supplementary Table S2 by age, sex and city ( Supplementary File 4). As with response percentages for the baseline interview these were higher in Arkhangelsk and among women and older people.

**Table 5.** Summary of health check attendance if interviewed at baseline by age, sex, and city.

Age group	Arkhangelsk						Novosibirsk					
	Men		Women		Total		Men		Women		Total	
	Number attending health check	Proportion of baseline interviewees attending health check	Number attending health check	Proportion of baseline interviewees attending health check	Number attending health check	Proportion of baseline interviewees attending health check	Number attending health check	Proportion of baseline interviewees attending health check	Number attending health check	Proportion of baseline interviewees attending health check	Number attending health check	Proportion of baseline interviewees attending health check
35–39	92	96.8	136	94.4	228	95.4	64	71.1	99	72.8	163	72.1
40–44	135	95.7	208	95.4	343	95.5	88	78.6	139	80.3	227	79.6
45–49	137	94.5	186	95.4	323	95.0	120	77.4	162	83.5	282	80.8
50–54	159	97.0	193	95.1	352	95.9	120	82.8	189	85.5	309	84.4
55–59	153	96.2	215	97.3	368	96.8	140	84.3	197	91.2	337	88.2
60–64	160	94.7	205	98.6	365	96.8	186	78.8	222	90.2	408	84.6
65–69	153	96.8	249	96.1	402	96.4	191	79.9	244	87.1	435	83.8
All ages	989	95.9	1392	96.1	2381	96.0	909	79.5	1252	85.4	2161	82.8

* Age from self-report of age at baseline interview

There is evidence that those who did not attend the health check were different to those who did. The associations of characteristics measured at baseline with not subsequently having a health check are shown in the form of odds ratios in Supplementary Table S3 ( Supplementary File 4). Adjusting simultaneously for city, age, sex, and education and distance from the clinic, those who did not have a the health check were more likely to be younger, male, with lower educational level, not in regular paid employment, have a worse financial situation, problem drinkers, smokers and report symptoms of major depression. Those who self-reported a history of hypertension, high cholesterol, myocardial infarction, heart failure or angina were more likely to have a health check but those who with self-reported previous stroke were less likely to do so. Participants living further away from the clinic were also less likely to attend the health check.

***Patients receiving treatment for alcohol problems***. In total 275 participants receiving treatment for alcohol problems were recruited from Arkhangelsk out of 322 patients invited to take part (85.4%). It should be noted that although clinicians were instructed to invite all eligible participants they were allowed to use their clinical judgement as to who should be approached, as the patient’s well-being was considered paramount. However this sample was not intended to be representative of all patients receiving treatment for alcohol problems in Arkhangelsk but to obtain a sample of people who drank extremely heavily. The sample was predominantly male (76.4% men) and the age distribution was skewed toward younger aged participants (median age 47 IQR 41–55).

### Data collection

***Ethical Approval and Consent***. Ethical approval for the study was received from the ethics committees of the London School of Hygiene & Tropical Medicine (approval number 8808 received 24.02.2015; for sub-study involving patients in treatment for alcohol problems approval number 12018; received 11/01/2017), Novosibirsk State Medical University (approval number 75 approval received 21/05/2015), the Institute of Preventative Medicine (no approval number ; approval received 26/12/2014), Novosibirsk and the Northern State Medical University, Arkhangelsk(approval number 01/01-15 received 27/01/2015; for sub-study involving patients in treatment for alcohol problems approval number 05/11-16 received 02/11/2016).

Signed informed consent was obtained both at baseline interview and at the health check. At baseline interview the consent was obtained for passing on name, address, and telephone number to the polyclinic medical team for those deciding to have a health check. Agreement for interview *per se* was obtained verbally. At the health check written informed consent was obtained for participation in the study. Participants were given the option also to consent to be re-contacted by the study team in the future.

***Baseline Interview***. At the baseline interview, a questionnaire was administered by a trained interviewer using a computer assisted personal interviewing device (CAPI) implemented on a tablet computer. For quality assurance purposes these devices were programmed so both location of the interview (using GPS) and the time taken for each question were recorded automatically. The topics covered at the interview are summarised in Table 6. Where appropriate we used established and validated questions or question sets, as indicated in Table 6. The interview, which took a median of 36 minutes (IQR 30.9-44.2 minutes), included sections on socio-demographic factors, physical activity, physical health (including health service use and adherence to medications for hypertension and hypercholesterolemia), various measures of self-reported health including the Short-Form 12 health survey (SF-12) ^13^, depression symptoms using the Patient Health Questionnaire 9 (PHQ-9) and anxiety symptoms using the Generalized Anxiety Disorder 7 (GAD-7) both from the Patient Health Questionnaire ^14^, diet quality score ^15^, smoking, household structure and socio-economic circumstances, and psychosocial factors and life events ^16, 17^. The questionnaire included particularly detailed questions on alcohol use including standard questions on the frequency and usual quantity of beverage alcohols (designed for and previously used in Russia ^18^), frequency of acute dysfunctional drinking behaviours such as hangover and excessive drunkenness, period of continuous drunkenness lasting 2 or more days during which a participant is withdrawn from normal life (known in Russian as zapoi) and the CAGE score for detecting problem drinking ^19^.

**Table 6.** Data items collected at each stage.

Data Item		Source of questions
Baseline questionnaire		
Socio-demographic factors	• Age • Sex • Marital status • Education • Employment status and Occupation	Questions were taken from questionnaires used previously in Russia (Izhevsk Family Studies 1 and 2) ^22, 23^. Educational categories were based on those used in the Russian census and used in the Russian Longitudinal Monitoring Survey ^24^ Additional questions on occupation were based on the International Standard Classification of Occupations ^25^.
Physical activity	• EPIC Physical Activity Questionnaire	The EPIC Physical Activity Questionnaire ^26, 27^ is a short questionnaire validated in 10 European countries ^28^.
Health Care Service use, contact and treatment	• Use of health care services in the past 12 months • Received any advice from doctor on lifestyle change • Treatment and adherence to treatment for hypertension and high cholesterol • Attendance of health screening programme (Dispansarisation)	Question on knowledge about hypertension from the NCD Knowledge, Attitudes and Practices Survey ^29^. Other questions drafted for this study.
Self-reported health	• SF-12 • Self-reported morbidity	SF-12 ^13^ is a validated questionnaire used for measuring self-reported physical and mental health. Self-reported morbidity questions based on the Tromsø 7 questionnaire ( http://Tromsoundersokelsen.uit.no/Tromso/).
Mental Health	• Depression (PHQ-9) • Anxiety (GAD-7)	Validated screening tools (PHQ-9, GAD-7) from the Patient Health Questionnaire ^14^ were used.
Diet	• Dietary Quality Score	Modified version of short questionnaire found previously to predict cardiovascular disease ^15^.
Smoking	• Smoking status • Amount smoked per day • Offered help or advice on smoking cessation	Questions were taken from Izhevsk family Study 2 Questionnaires ^22, 23^. Additional questions drafted for this study on seeking help or advice on smoking cessation.
Alcohol Use	• Beverage specific quantity-frequency • Frequency of alcohol-related dysfunctional behaviours such as hangover • Consumption of non-beverage alcohol • CAGE • Use of services for problem drinking	Questions were taken from Izhevsk Family Study questionnaires ^22, 23^. CAGE ^19^ score is a well-established tool for identifying problem drinking. It has been used previously in Russia in the HAPIEE study ^9^ and the same translation and adaption to use 12 month reference period were used to be comparable with this study.
Household	• Household structure • Household asset index • Household financial situation	Questions were taken from Izhevsk family Study questionnaires ^22, 23^. Additional questions on squared metres and number of rooms in the house or apartment taken from Russian Longitudinal Monitoring Survey ^24^.
Pyschosocial factors	• Life events in the past 6 months ^16, 17^ • Physical assault in the past year • Relations with family • Beliefs about gender roles • Social support • Social capital and trust ^30, 31^ • Health-related self-efficacy	Life events from the List of Threatening Experiences ^16, 17^. Gender roles questions from the Russian Longitudinal Monitoring Survey 2003 ^24^. Social capital and trust – validated questions ^30, 31^. Health-related self-efficacy questions from the Tromsø 7 questionnaire ( http://Tromsoundersokelsen.uit.no/Tromso/) Other questions from the Izhevsk Family Study questionnaires ^22, 23^.
Health Check Questionnaire		
Cardiovascular Health	• Self-reported cardiovascular morbidities • Rose angina questionnaire (short form) ^21^ and complete questionnaire (subset) ^32, 33^	One question on self-reported health from the Tromsø 7 questionnaire ( https://tromsoundersokelsen.uit.no/tromso/). Other questions from the Izhevsk Family Study-2 questionnaire ^23^ or questions drafted for this study. Rose angina questionnaire is an established validated instrument ^32, 33^.
General health	• Use of medications • Symptoms of respiratory disease • MRC breathlessness scale • Self-reported morbidities (cancer, liver disease, injuries)	Reporting of medications using structured proforma as in the Izhevsk Family Study-2 questionnaire ^23^. Respiratory disease questions from the MRC (UK) Respiratory Questionnaire 1986 and the MRC breathlessness scale ^20^. Self-reported morbidities from the Izhevsk Family Study-2 questionnaire ^23^ or questions drafted for this study.
Women’s health	• Pregnancy and menstrual history • Use of hormone replacement therapy	Subset of questions from the Children of the 1950s Aberdeen Study ^34^ and MRC National Survey of Health and Development ^35^. Additional questions on pregnancy history drafted for this study.
Alcohol use	• Alcohol Use Disorders Identification Test (AUDIT) ^36, 37^	The AUDIT is a validated tool for screening for hazardous or harmful drinking ^36, 37^.
Smoking	• Smoking status • Amount smoked per day	Identical sub-set of questions from the baseline questionnaire.
Health check Physical Examination		
Anthropometry	• Height • Weight • Waist circumference • Hip circumference • Body composition	
Physical function	• Grip strength • Time for 10 chair stands • Standing balance (eyes open and closed)	
Physical activity	• 5 days physical activity monitoring with an actiheart device: offered to 50% of participants	
Lung function	• Spirometry (FVC, FEV _1_ ): offered to 50% of participants • Pulse oximetry	
Cardiovascular profile	• Blood pressure • Heart rate • ECG • Pulse wave velocity • Echocardiography • Vascular ultrasound	
Collection of biological samples	• Blood sample • Urine sample (provided by subset of participants) • Faecal sample (provided by subset of participants)	

***Health Check examination***. The health check included a questionnaire and a physical examination. All aspects of the health check were specified in detail in the form of standard operating procedures. The whole health check took an average of approximately three hours. The questionnaire was administered by either a nurse or a cardiologist. It included questions on past medical history including previous diagnoses of breathlessness measured using the Medical Research Council Breathlessness Scale ^20^ and the short form of the Rose Angina questionnaire ^21^. Participants were asked to bring all their medications with them to the health check including inhalers (although only 27% of participants did so) and names and doses used per day were recorded. A maximum of 7 medications were recorded for each person. Women were asked questions about their pregnancy history including history of gestational diabetes and hypertension, and their use of hormone replacement therapy. Hazardous alcohol use was assessed using the Alcohol Use Disorder Identification Test (AUDIT) ^36^. Baseline interview questions on smoking were repeated.

A summary of the components of the physical examination including the devices used for measurement is shown in Table 7. Briefly the physical examination included measurements of blood pressure, pulse oximetry, anthropometry (height, waist and hip circumference, weight and body composition), digital ECGs, pulse wave velocity and pulse wave analysis. Physical function was assessed through measurement of grip strength using the Southampton protocol ^38^, the time taken to stand up and sit down from a chair ten times in line with the MRC National Survey of Health and Development Protocols ^35^, and standing balance on one leg with eyes open and eyes closed using protocol from the National Health and Aging Trends Study (Funded by the National Institute of Aging (U01AG032947); 2011).

**Table 7.** Summary of physical examinations components.

Measurement	Device or equipment used	Comments on protocol	Percent of participants attending health check with data collected *
Blood pressure	OMRON 705 IT automatic blood pressure monitors (OMRON Healthcare) ^39^	Three measurements of sitting blood pressure taken 2 minutes apart.	98.9%
Pulse Oximetry	Nonin Onyx II 9550 non-invasive finger tip pulse oximeters (Nonin Medical Inc, USA)		99.7%
Weight and body composition	TANITA BC 418 body composition analyser (TANITA, Europe GmbH)	Weight only (not body composition) measured in participants with pacemaker, pregnant or refused	98.1% with body composition data
Height	Seca® 217 portable stadiometer (Seca Limited)	Two measurements	99.9%
Hip and waist circumference	Seca measuring tapes (Seca®201) (Seca Limited)	Two measurements	99.9%
Grip strength	JAMAR® digital hand dynamometers (Patterson Medical, UK)	Three measurements per hand in accordance with the Southampton protocol ^38^	96.9%
Chair stands	-	Time taken to stand up and sit down from a chair ten times	97.3%
Standing balance	-	Time standing on one leg 1)with eyes open and 2)eyes closed	97.3%
Digital ECG	Cardiax devices (IMED ltd, Hungary)		99.8%
Pulse wave velocity and pulse wave analysis	Non-invasive Vicorder devices (Skidmore Medical Ltd, UK) ^40^	Three measurements taken 1 minute apart. If the measurements were greater than 0.5m/s apart or there were concerns about record quality further measurements (up to 7) were taken	99.5%
Vascular ultrasound and echocardiography examination	GE VividQ machines (GE Health care)	In accordance with a strict protocol	99.5%
Energy expenditure over 5 days	Actiheart (CamNtech Ltd, Cambridge, UK)	Offered to approximately 50% of participants. Participants who agreed to this component of the study took part in a 200m walking test first for calibration purposes.	21.6%
Spirometry	6800 pneumotrac spirometers (Vitalograph®, UK)	Offered to approximately 50% of participants. Three measurements were taken. If less than two acceptable measurement taken additional measurements could be taken up to a maximum of eight.	45.7%

* Denominator all main study participants where health check was completed

The clinics were requested to offer 50% of the participants lung function tests and the option to wear a combined heart rate and movement sensor (Actiheart, CamNtech Ltd, UK) on the chest for 5 days in order to provide an objective measure of physical activity ^41^. Those wearing the monitor were asked to complete a 200m self-paced walk test for the purposes of individual calibration of the heart rate response ^42^. This approach was recently found to be valid for estimating free-living activity energy expenditure ^43^. For practical reasons, the selection of participants to be offered these additional components was done on the basis of offering them to all participants on days when medical personnel trained in these procedures were working in the clinic. The days of the week these procedures were offered on varied throughout the course of the fieldwork and included weekends.

Vascular ultrasound and echocardiography examination were done in accordance with a very detailed protocol. Participants underwent transthoracic echocardiography (ECHO) in the left lateral decubitus position using a commercially available systems equipped with a 1.0 ~ 5.0 MHz matrix sector transducer (Vingmed Vivid *q* or E9, GE Healthcare, Horten, Norway). A common standard operational procedure (SOP) for ECHO was developed for the study by an international team of nine leading experts (including AR, SM, HS, AH, DL) which was used in the Know Your Heart (Russia) study and in the Tromsø 7 (Norway) study. ECG-gated M-mode and two-dimensional grey-scale images as well as pulsed, continuous and colour Doppler data were acquired in the parasternal and apical views with breath hold to ensure image quality. Gray-scale images were obtained with only one focal zone to ensure a frame rate of at least 50 frames per second.

Images were recorded digitally in cine-loop format or still images as appropriate and analysed off-line with commercial software EchoPAC (v.113, GE-Vingmed AS, Horten, Norway). Off-line ECHO analysis was performed by 1 investigator (MS) for images obtained in Norway (Tromsø 7 study) and by the central reading laboratory in Novosibirsk by 2 investigators (AR, SM) for images obtained in Russia. Left ventricular (LV) and atrial volumes were measured from the apical 2- and 4-chamber views and LV ejection fraction (LVEF) calculated using the biplane Simpson’s technique ^44^. LV mass and relative wall thickness (RWT) were estimated from M-mode recordings according to current recommendations ^44^. Chamber volumes and LV mass were indexed to body surface area. Doppler measurements of aortic, mitral, pulmonary and tricuspid valve flow were obtained according to current guidelines and the recommended grading of any detected valvular heart disease were followed ^45, 46^. We evaluated global longitudinal strain and strain rate of LV by 2D speckle tracking technique. PW Doppler tissue velocities of mitral annulus were traced for additional quantification of systolic and diastolic ventricular function ^45^. Intra- and inter-reader variability was regularly assessed within both reading laboratories and between the Russian and Norwegian reading teams.

Vascular ultrasound (VUS) of carotid arteries was conducted in accordance with the study SOP for VUS with the participant in a supine position using a commercially available system equipped with a 3~13 MHz linear transducer (Vingmed Vivid *q* or E9, GE Healthcare, Horten, Norway). ECG-gated high-resolution two-dimensional grey-scale images were obtained in longitudinal and transverse views. The highest probe frequency was applied with only 1 focal zone and the highest frame rate (at least 40 frames per sec). VUS images were recorded digitally in cine-loop format or still images and analyzed off-line with software EchoPAC (v.113, GE-Vingmed AS, Horten, Norway).

Off-line vascular analysis was performed by 2 investigators (AR, SM) in the reading laboratory in Novosibirsk, Russia. Computer-assisted measurement of both common carotid arteries intima-media thickness and assessment of carotid plaques (Mannheim Consensus; 2004-2006-2011) and patterns of artery wall structure were conducted. Intra- and inter-reader variability was regularly assessed.

All participants were asked to give a blood sample. Since the health checks took place throughout the day it was not considered feasible to ask participants to fast for 12 hours but participants were asked to fast for 4 hours prior to attending the health check. Questions about time of last meal and drinks consumed in the past four hours including caffeine and consumption of alcohol in the past 24 hours were asked by the receptionist on arrival and these data were recorded.

Blood samples were collected in 4 SST II vacutainers (8.5ml) and 2 EDTA vacutainers (10ml and 6ml) BD® (Beckton, Dickinson and Company, Preanalytical Systems, US). Serum vacutainers were left at room temperature for 30 minutes and then stored at 4°C while EDTA vacutainers were stored immediately at 4°C. The 10ml EDTA tube and the 4 SST tubes were centrifuged in cooled centrifuges at 4°C at 2100–2200g for 15 minutes. Samples were aliquoted in to 1.8 ml Nunc® cryotubes® (10 cryovials of serum, 3 cryovials of plasma and 4 cryovials of whole blood). We aimed for processing, aliquoting and freezing of blood samples within a target of 2 hours after sample collection (using time stamps from modules used within the laboratories at time of sample processing we confirmed this this was achieved for 84% of samples: 100% of samples in Arkhangelsk and 63% of samples Novosibirsk). Vacutainers and cryovials were uniquely identified using bar-coded labels.

Participants were asked to volunteer a spot urine sample and faecal samples for analysis of the gut microbiome. Those who agreed were provided with appropriate collection kits and instructions and requested to provide samples while they were in the clinic, or to return samples to the clinic later. The proportion of participants providing both types of optional sample was considerably higher in Arkhangelsk (urine 59%, faecal 43%) than in Novosibirsk (urine 26%; faecal 9%) and was particularly high for the participants recruited from alcohol services (urine 99.6%; faecal 89%). If providing the sample at home participants were instructed to store samples at 4°C and return to the clinic within 18 hours in order to meet target of freezing samples within 24 hours.

Blood, urine and faecal samples were initially processed and stored at -20°C for a maximum of three weeks and then transferred to -80°C freezers. Periodically throughout the study biological samples were shipped to Moscow and stored at -80°C. Analysis was performed in one period at the end of the study with samples from both sites analysed in parallel and not sequentially. The core set of biochemistries analysed using the blood and urine samples are listed in Table 8. The target and achieved number of cryovials per participant of each biological sample type is shown in Table 9.

**Table 8.** Core set of biological analyses on blood and urine sample.

Target	Specific measures	Biological sample	Method	Technology used for analysis
Lipids	Total cholesterol	Serum	Enzymatic Color Test	AU 680 Chemistry System Beckman Coulter
	High Density Lipoprotein Cholesterol (HDL)	Serum	Enzymatic Color Test	AU 680 Chemistry System Beckman Coulter
	Low Density Lipoprotein Cholesterol (LDL)	Serum	Enzymatic Color Test	AU 680 Chemistry System Beckman Coulter
	Triglycerides	Serum	Enzymatic Color Test	AU 680 Chemistry System Beckman Coulter
	Apolipoprotein A1	Serum	Immuno-turbidimetric Test	AU 680 Chemistry System Beckman Coulter
	Apolipoprotein B	Serum	Immuno-turbidimetric Test	AU 680 Chemistry System Beckman Coulter
	Lp(a)	Serum	Particle Enhanced Immunoturbidimetric Test	AU 680 Chemistry System Beckman Coulter
Renal function	Creatinine	Serum	Kinetic Color Test (Jaffe)	AU 680 Chemistry System Beckman Coulter
	Cystatin C	Serum	Particle Enhanced Immunoturbidimetric Test	AU 680 Chemistry System Beckman Coulter
	Albumin	Urine	Immuno-turbidimetric Test	AU 680 Chemistry System Beckman Coulter
	Creatinine	Urine	Kinetic Color Test (Jaffe)	AU 680 Chemistry System Beckman Coulter
Inflammatory markers	High sensitivity C Reactive Protein (CRP)	Serum	Immuno-turbidimetric Test	AU 680 Chemistry System Beckman Coulter
Iron Pathways	Transferrin	Serum	Immuno-turbidimetric Test	AU 680 Chemistry System Beckman Coulter
Metabolites	HbA1c	Whole blood (EDTA)	Immuno-turbidimetric Test	AU 680 Chemistry System Beckman Coulter
Liver function tests	Gamma glutamyl transferase (GGT)	Serum	Kinetic Color Test (IFCC)	AU 680 Chemistry System Beckman Coulter
	Aspartate Transanimase (AST)	Serum	Kinetic UV-Test P5P activated (IFCC)	AU 680 Chemistry System Beckman Coulter
	Alanine Transanimase (ALT)	Serum	Kinetic UV-Test P5P activated (IFCC)	AU 680 Chemistry System Beckman Coulter
Cardiac micronecrosis	High sensitivity Troponin T	Serum	The electrochemi-luminescence immunoassay “ECLIA”	Cobas e411 analyser (Roche Diagnostics GmbH, Hitachi, Japan)
	NT-Pro-B type Natriuretic Peptide (Nt-Pro-BNP)	Serum		Cobas e411 analyser (Roche Diagnostics GmbH, Hitachi, Japan)
Alcohol biomarker	Carbohydrate Deficient Transferrin (CDT) (Subset 2500 Participants)	Serum	Capillary Electrophoresis, CAPILLARYS-2	Capillarys automatic capillary electrophoresis system (CAPILLARYS-2), Sebia S.A., France.

**Table 9.** Target and achieved number of biological sample cryovials per participant.

Biological sample type	Sample collection	Target number of cryovials per participant by use					Achieved mean number of cryovials / participant among those who provided samples	Percentage of participants who provided at least one cryovial of each sample type for analysis *
		Immediate feedback to participants	Local biobank in Arkhangelsk / Novosibirsk	Planned analyses in Moscow	Long-term biobank in Moscow	Total target number of cryovials		
Serum	4 X SST II8.5 ml Beckton Dickinson vacutainer	1	2	5	2	10	9.67	97.5
Plasma	1 X 10ml EDTA Becton Dickinson vacutainer	0	2	0	1	3	2.93	96.6
Whole blood	1 X 6ml EDTA Becton Dickinson vacutainer	0	1	1	2	4	3.87	96.3
Urine	Becton Dickinson urine collection pots	0	0	1	2	3	3.00	45.3
Faecal sample	Nuova Aptaca faecal sample collection pots	0	0	2	1	3	2.70	28.8

* Denominator was number of health check participants in both cities including all sub-studies. As the first serum cryovial was used for participant feedback it was not included in calculation

Electronic data capture for all aspects of the health check was used to reduce data entry errors that occur when using paper forms. This included all interview responses as well as output from all measurement devices. In some cases the data files created by the devices were also automatically captured. Exceptions were measurement of height, waist and hip circumference, pulse oximetry and the physical function tests (grip strength, chair stands, standing balance) in which the values were entered via the keyboard. The processing of laboratory samples was also done using a bespoke sample handling application. Data capture software was created using SURVANT (Netelixis IT Solutions. SURVANT survey authoring and data collection software: version 1.0).

Detailed reports on data quality were created each month on key areas to be monitored such as characteristics of participants, GPS location of interviews, and inter-operator variability. These reports were reviewed by the central study team in a monthly meeting with immediate feedback provided to the fieldwork sites.

The questionnaires and data collection tools used in the study are shown in Supplementary File 3. These files show paper versions of the questions used however all data collections was done electronically (CAPI for the baseline questionnaire and SURVANT for the health check.)

### Gut microbiome sub-study

The gut microbiome refers to digestive-tract associated microbes, and more than 1,000 microbial species-level phylotypes can be accessed by sequencing the 16S ribosomal RNA genic region of faecal DNA samples. An Imbalance of the normal gut microbiome has been linked with gastrointestinal conditions (e.g. Inflammatory bowel disease, Irritable bowel syndrome), systemic metabolic diseases (e.g. obesity, type 2 diabetes), and atopy, but underlying studies tend to have low sample size. Whilst, the microbiome is affected by factors such as age, antibiotic use, and diet, the composition of the microbiota is thought to be relatively stable within healthy adult individuals over time ^47^. Within this study it is proposed to establish the relative abundance of microbes by 16S (V3-V4 region) sequencing of faecal samples (n=1000) collected in Arkhangelsk. The resulting characterization of the gut microbiome will be correlated with CVD outcomes, using regression-based association test that accounts for the rich set of confounders collected in the study. Analysis of the repeat samples collected a year later as part of the repeatability sub-study will facilitate an assessment of within-person microbiota stability over time. The presence of phylotypes that may be linked to cardiovascular outcomes will be confirmed using a metagenomic approach, where whole genomes, rather than targeted 16S, are characterized.

### Comparison with Tromsø 7 “Heart to Heart Study”

The central objective of IPCDR is to understand why Russia has such high cardiovascular mortality compared to other countries. From the outset we planned to make comparisons of the cardiovascular phenotypes observed in Arkhangelsk and Novosibirsk with those observed in the 7 ^th^ wave of the Tromsø study. To facilitate this comparison UiT, the Arctic University of Norway which runs the Tromsø study has created the *Heart to Heart* initiative which provides an umbrella under which this comparative scientific work can be developed.

The Tromsø Study is a population based survey of residents of the municipality of Tromsø in Northern Norway ^48^. To date there have been 7 waves of data collection with the first wave starting in 1974. Tromsø, the largest city in Northern Norway and situated ~400km north of the Arctic Circle, is geographically close to Arkhangelsk and as such is a particularly interesting study with which to make international comparisons.

Data collection for the Tromsø 7 Study took place between 2015 and 16 with a total of 21,000 men and women aged 40 and above living in Tromsø examined. During the development phases of both Tromsø 7 and the IPCDR Know Your Heart Study we aligned aspects of the medical examination and laboratory analyses to make the data as comparable as possible. In particular an identical protocol was used for the ECHO measurements in both studies. A validation study has been carried out by the University Hospital of North Norway laboratories whereby split samples were analysed in Norway and in the Moscow laboratories used for conventional biochemistries. Some of the key areas for comparison between the Tromsø study and IPCDR studies are shown in Supplementary Table S4. The University Hospital in Tromsø has also undertaken a validation study to compare measurement of body composition using the two types of device used in each study. A similar validation study was done for the measurement of physical activity.

### Data and statistical analysis plan

Data analyses will be focused around examining the associations and comparisons of interest shown in Figure 1. The analysis plan consists broadly of two parts: analysis of associations between key exposures of interest and cardiovascular phenotypes within Russia and 2) comparisons between Russia and other studies, particularly Tromsø 7. Examples of proposed analyses include:

1) Comparisons between Know Your Heart and Tromsø 7 on cardiovascular risk factors such as blood pressure and body mass index will be carried out by calculating means for continuous variables and proportions for categorical variables for all participants aged 40–69 stratified by sex and age standardized to the 2013 European Standard Population.
2) One of our exposures of interest will be alcohol use. The data on self-reported consumption from the questionnaires and biomarker data (gamma glutamyl transferase, carbohydrate deficient transferrin for a sub-set) will be used to divide participants into appropriate groups (non-drinker, light drinker, moderate drinker, hazardous drinker (population sample), and hazardous drinker (diagnosed alcohol use disorder). Associations with cardiovascular disease phenotypes will be analysed using logistic and linear regression with adjustment for pre-specified confounders.
3) Alongside well-established, clinical measures of cardiovascular phenotype such as left ventricular ejection fraction, carotid intima media thickness (cIMT) and plaque, we are proposing to investigate whether there are underlying latent dimensions of cardiovascular phenotypes which can be obtained from the data using factor analysis.

A range of statistical analysis programs will be used including STATA (StataCorp LP), IBM SPSS stastistics (IBM Corporation) and R.

## Discussion

### Strengths and limitations

This study has collected very detailed data on cardiovascular profile and risk factors for cardiovascular disease from the general population of two geographically distinct cities within Russia. The close connection with the Tromsø 7 study allows for comparisons between Russia and Norway in the same calendar years with considerably less chance that differences are due to study methodology than many comparisons between population-based surveys. To our knowledge the inclusion of participants receiving treatment for alcohol problems in an in-depth study of cardiovascular phenotypes alongside a general population sample is unique. The use of the same tools and measurement procedures in both populations is an important strength of the study.

One of the potential limitations of the study is the low response rate for Novosibirsk. This creates uncertainty about the generalizability of study findings particularly around estimation of prevalences and mean values of parameters that will affect comparisons that can be made with other countries. In both sites response rates were higher in women and older people. However, non-response patterns were complex, and the extent to which they may limit inferences with the Tromsø 7 study will depend upon the direction and magnitude of the differences found. For example, the fact that in Novosibirsk the educational profile of participants was weighted more towards those with higher education than the population of the city as a whole, might be expected in many cases to minimize differences with Tromsø.

There are several possible reasons for the poorer response rates in Novosibirsk than Arkhangelsk. Novosibirsk is an appreciably larger city than Arkhangelsk, with citizens being potentially more suspicious of approaches to take part in research. The smaller size of Arkhangelsk was one of the factors that facilitated good links with the city government who provided extensive support for an intensive public information campaign about the study in the city that was on a larger and more sustained basis than in Novosibirsk. Finally, the smaller size of Arkhangelsk and the location of the research polyclinic in the center of the city made it easier for participants to attend than may have been the case in Novosibirsk.

Despite these limitations the richness of the data collected means there is an unparalleled opportunity for in-depth analysis of cardiovascular phenotypes and much greater understanding of how these are associated with a wide range of biological, psychological and socio-economic risk factors within Russia and with the Tromsø 7 study ( Figure 1).

While this study was designed as a cross-sectional study consent for recontacting participants and accessing health records was obtained therefore there is potential to obtain follow up data in the future.

### Dissemination of information

Bona fide researchers will be able to apply for subsets of the data from this study for research purposes. In addition, we are establishing a biobank of the biological samples collected in Russia. Researchers will be able to apply to analyse these samples within Russia. Further information about the study including details of how to access data and samples will be available at https://knowyourheart.science/ [ *active from June 2018*]. This website will be updated from time to time with summaries of findings and links to a separate meta-data website documenting all variables available. Dissemination of the study findings will be through publishing in peer reviewed journals, presenting at conferences within Russia and internationally and though meetings with policy makers.

### Study status

Data collection for the study is completed. Data cleaning is now underway and key analyses are in process.

## Data availability

No data is associated with this article.

## Supplementary material

**Supplementary File 1: Promotional video shown on locally on TV (Arkhangelsk version)**

**Supplementary File 2: Promotional materials (billboard advertisements) used to advertise the study within each city and photographs**

**Supplementary File 3: Questionnaires and data collection tools.** These are paper versions of the questions used however all data entry was done electronically

**Supplementary File 4: Supplementary Tables (S1–S4)**

**Supplementary Table S1: Codes used for recording the outcome of visits to addresses**

**Supplementary Table S2: Health Check response percentages by age, sex and city**

**Supplementary Table S3: Associations between selected characteristics at baseline interview and not having a health check**

**Supplementary Table S4: Potential areas of comparison between Tromsø 7 and International Project on Cardiovascular Disease in Russia (IPCDR) Know Your Heart Study**
