# Supplementary material for: Know Your Heart: Rationale, design and conduct of a cross-sectional study of cardiovascular structure, function and risk factors in 4500 men and women aged 35-69 years from two Russian cities, 2015-18
Source: Wellcome Open Res. 2018 Dec 3;3:67. Originally published 2018 Jun 4. [Version 3] doi: 10.12688/wellcomeopenres.14619.3 (PMC6073094; doi:10.12688/wellcomeopenres.14619.3)
Supplement: Supplementary file 4 [file wellcomeopenres-3-16294-s0003.tgz › f5db3f84-a41d-4b43-92c2-b322d3386096.docx]

**Supplementary Material**

**Supplementary Table S1: Codes used for recording the outcome of visits to addresses**

| Code | Denominator  response  type 1 | Denominator  response  type 2 | Denominator  response  type 3 |
| --- | --- | --- | --- |
| 1 Invalid address |  |  |  |
| 2 Not possible to reach the apartment* |  |  |  |
| 3 No one at home |  |  |  |
| 4 No adults or capable adults are at home |  |  |  |
| 5 Refused to open the door |  |  |  |
| 6 No person with needed age/sex |  |  |  |
| 7 Person no longer at the address (new address is unknown) |  |  |  |
| 8 Person no longer at the address (new address is known) |  |  |  |
| 9 In hospital or on a long leave |  |  |  |
| 10 The needed respondent was not at home |  |  |  |
| 11 Not able to answer questions |  |  |  |
| 12 Does not speak Russian |  |  |  |
| 13 Interview rescheduled |  |  |  |
| 14 Refused to take part |  |  |  |
| 15 Interview interrupted |  |  |  |
| 88 Interview conducted |  |  |  |

*This category primarily affected apartment buildings with restricted access due to security gates

Type 1 response % denominator: All addresses where any attempt at contact was made (all codes)

Type 2 response % denominator: The outcome of the final visit was not possible to reach the apartment (code 2)/ no one at home (code 3)/ no adults or capable adults at home (code 4)/refused to open the door (code 5) / in hospital or long leave (code 9)/ the needed respondent was not at home (code 10)/not able to answer questions (code 11)/ does not speak Russian (code 12)/ interview rescheduled (code 13)/ refused to take part (code 14)/ interview interrupted and not completed (code 15)/interview conducted (code 88)

Type 3 response % denominator: The outcome of the final visit was in hospital or long leave (code 9)/the needed respondent was not at home (code 10)/ /interview rescheduled (code 13)/refused to take part (code 14)/ interview interrupted and not completed (code 15)/interview conducted (code 88)

**Supplementary Table S2: Health Check response percentage by age, sex and city**

| Type of response % * | Age group** | Arkhangelsk | | | | | | Novosibirsk | | | | | |
| --- | --- | --- | --- | --- | --- | --- | --- | --- | --- | --- | --- | --- | --- |
|  |  | Men | | Women | | Total | | Men | | Women | | Total | |
|  |  | Number attended health check | Response % | Number attended health check | Response % | Number attended health check | Response % | Number attended health check | Response % | Number attended health check | Response % | Number attended health check | Response % |
| Response type 1 | 35-39 | 92 | 20.2 | 136 | 32.7 | 228 | 26.2 | 64 | 6.3 | 99 | 10.6 | 163 | 8.3 |
|  | 40-44 | 135 | 27.7 | 208 | 43.9 | 343 | 35.7 | 88 | 8.8 | 139 | 14.3 | 227 | 11.5 |
|  | 45-49 | 137 | 31.1 | 186 | 45.0 | 323 | 37.8 | 120 | 11.9 | 162 | 17.1 | 282 | 14.5 |
|  | 50-54 | 159 | 39.0 | 193 | 50.1 | 352 | 44.4 | 120 | 13.4 | 189 | 25.3 | 309 | 18.8 |
|  | 55-59 | 153 | 41.4 | 215 | 56.4 | 368 | 49.0 | 140 | 15.0 | 197 | 27.8 | 337 | 20.5 |
|  | 60-64 | 160 | 47.6 | 205 | 57.6 | 365 | 52.8 | 186 | 20.3 | 222 | 31.9 | 408 | 25.3 |
|  | 65-69 | 153 | 49.0 | 249 | 60.4 | 402 | 55.5 | 191 | 20.2 | 244 | 33.7 | 435 | 26.0 |
|  | All ages | 989 | 35.2 | 1392 | 49.0 | 2381 | 42.2 | 909 | 13.5 | 1252 | 21.8 | 2161 | 17.4 |
| Response type 2 | 35-39 | 92 | 26.2 | 136 | 40.2 | 228 | 33.1 | 64 | 8.6 | 99 | 13.6 | 163 | 11.1 |
|  | 40-44 | 135 | 34.7 | 208 | 51.5 | 343 | 43.3 | 88 | 11.8 | 139 | 18.2 | 227 | 15.0 |
|  | 45-49 | 137 | 39.1 | 186 | 53.6 | 323 | 46.3 | 120 | 15.9 | 162 | 22.3 | 282 | 19.0 |
|  | 50-54 | 159 | 49.2 | 193 | 59.4 | 352 | 54.3 | 120 | 17.3 | 189 | 30.0 | 309 | 23.3 |
|  | 55-59 | 153 | 48.1 | 215 | 65.2 | 368 | 56.8 | 140 | 18.4 | 197 | 33.3 | 337 | 24.9 |
|  | 60-64 | 160 | 55.9 | 205 | 65.3 | 365 | 56.3 | 186 | 24.8 | 222 | 35.9 | 408 | 29.8 |
|  | 65-69 | 153 | 63.5 | 249 | 70.3 | 402 | 67.0 | 191 | 26.4 | 244 | 41.1 | 435 | 33.0 |
|  | All ages | 989 | 43.8 | 1392 | 57.7 | 2381 | 51.0 | 909 | 17.6 | 1252 | 26.9 | 2161 | 22.0 |
| Response type 3 | 35-39 | 92 | 43.2 | 136 | 59.7 | 228 | 51.7 | 64 | 15.1 | 99 | 23.5 | 163 | 19.0 |
|  | 40-44 | 135 | 50.0 | 208 | 69.3 | 343 | 60.2 | 88 | 20.2 | 139 | 29.5 | 227 | 24.9 |
|  | 45-49 | 137 | 55.0 | 186 | 68.4 | 323 | 62.0 | 120 | 27.5 | 162 | 37.0 | 282 | 31.8 |
|  | 50-54 | 159 | 62.4 | 193 | 74.5 | 352 | 68.5 | 120 | 28.1 | 189 | 45.8 | 309 | 36.4 |
|  | 55-59 | 153 | 58.0 | 215 | 75.2 | 368 | 66.9 | 140 | 31.7 | 197 | 48.2 | 337 | 39.1 |
|  | 60-64 | 160 | 65.3 | 205 | 75.7 | 365 | 70.7 | 186 | 35.1 | 222 | 50.5 | 408 | 41.9 |
|  | 65-69 | 153 | 72.9 | 249 | 79.8 | 402 | 77.0 | 191 | 36.0 | 244 | 52.7 | 435 | 43.2 |
|  | All ages | 989 | 58.0 | 1392 | 72.2 | 2381 | 65.5 | 909 | 28.2 | 1252 | 41.0 | 2161 | 34.1 |

* Response type 1 denominator is total number of potential participants whose address was issued to interviewers. Type 2 denominator excluded addresses that could not be found or where no one of expected age and sex was found. Type 3 denominator restricted to those addresses where it was established that person of expected age and sex was resident. Further details can be found in Supplementary Table S1.

**Age self-reported at baseline interview or where participant was not interviewed age defined using expected age of participant at address from sampling frame

**Supplementary Table S3: Associations between selected characteristics at baseline interview and not having a health check**

|  | | Unadjusted | | | | Adjusted for city, age, sex, education, and distance from clinic | | | |
| --- | --- | --- | --- | --- | --- | --- | --- | --- | --- |
|  |  | OR | Lower 95% CI | Upper 95% CI | p-value (LR test) | OR | Lower 95% CI | Upper 95% CI | p-value (LR test) |
| City | Arkhangelsk | 1.00 |  |  | <0.001 | 1.00 |  |  | <0.001 |
|  | Novosibirsk | 5.10 | 4.07 | 6.38 |  | 5.70 | 4.53 | 7.18 |  |
| Self-reported Age from baseline interview 5-year categories | 35-39 | 1.00 |  |  | 0.002 | 1.00 |  |  | <0.001 |
|  | 40-44 | 0.68 | 0.48 | 0.96 |  | 0.65 | 0.45 | 0.94 |  |
|  | 45-49 | 0.74 | 0.53 | 1.03 |  | 0.65 | 0.46 | 0.93 |  |
|  | 50-54 | 0.58 | 0.41 | 0.82 |  | 0.53 | 0.37 | 0.76 |  |
|  | 55-59 | 0.47 | 0.33 | 0.67 |  | 0.37 | 0.26 | 0.54 |  |
|  | 60-64 | 0.59 | 0.43 | 0.83 |  | 0.41 | 0.29 | 0.58 |  |
|  | 65-69 | 0.63 | 0.45 | 0.86 |  | 0.45 | 0.32 | 0.63 |  |
| Sex | Male | 1.00 |  |  | <0.001 | 1.00 |  |  | 0.001 |
|  | Female | 0.71 | 0.59 | 0.85 |  | 0.74 | 0.61 | 0.89 |  |
| Education | Incomplete secondary | 1.00 |  |  | <0.001 | 1.00 |  |  | <0.001 |
|  | Secondary | 0.59 | 0.45 | 0.78 |  | 0.56 | 0.42 | 0.76 |  |
|  | Higher or incomplete higher | 0.35 | 0.26 | 0.47 |  | 0.29 | 0.21 | 0.40 |  |
| Current marital status | Married | 1.00 |  |  | 0.001 | 1.00 |  |  | <0.001 |
|  | Living together without marriage | 1.64 | 1.20 | 2.26 |  | 1.49 | 1.07 | 2.10 |  |
|  | Divorced or separated | 1.35 | 1.05 | 1.73 |  | 1.63 | 1.24 | 2.13 |  |
|  | Widower | 1.18 | 0.87 | 1.60 |  | 1.46 | 1.04 | 2.06 |  |
|  | Never married | 1.64 | 1.22 | 2.21 |  | 1.71 | 1.23 | 2.36 |  |
| In regular paid work | No | 1.00 |  |  | 0.108 | 1.00 |  |  | 0.015 |
|  | Yes | 0.79 | 0.67 | 0.95 |  | 0.77 | 0.62 | 0.95 |  |
| Officially registered disability | No | 1.00 |  |  | 0.014 | 1.00 |  |  | 0.065 |
|  | Yes | 1.42 | 1.08 | 1.87 |  | 1.33 | 0.99 | 1.79 |  |
| Total physical activity index | Inactive | 1.00 |  |  | <0.001 | 1.00 |  |  | 0.002 |
|  | Moderately inactive | 1.98 | 1.23 | 3.20 |  | 1.52 | 0.92 | 2.51 |  |
|  | Moderately active | 1.10 | 0.71 | 1.70 |  | 0.87 | 0.55 | 1.37 |  |
|  | Active | 1.51 | 0.96 | 2.36 |  | 1.04 | 0.64 | 1.67 |  |
| SF12 physical health | Quartile1 | 1.00 |  |  | 0.072 | 1.00 |  |  | 0.010 |
|  | Quartile2 | 0.87 | 0.68 | 1.13 |  | 0.89 | 0.68 | 1.16 |  |
|  | Quartile3 | 1.00 | 0.78 | 1.28 |  | 1.08 | 0.82 | 1.41 |  |
|  | Quartile4 | 1.21 | 0.95 | 1.54 |  | 1.40 | 1.06 | 1.84 |  |
| SF12 mental health | Quartile1 | 1.00 |  |  | 0.194 | 1.00 |  |  | 0.071 |
|  | Quartile2 | 1.11 | 0.86 | 1.43 |  | 1.22 | 0.94 | 1.59 |  |
|  | Quartile3 | 1.03 | 0.79 | 1.34 |  | 1.14 | 0.87 | 1.51 |  |
|  | Quartile4 | 1.28 | 1.00 | 1.62 |  | 1.40 | 1.09 | 1.82 |  |
| Depression severity (PHQ-9) | <5 No depression | 1.00 |  |  | <0.001 | 1.00 |  |  | <0.001 |
|  | 5-9 Mild depression | 0.75 | 0.60 | 0.94 |  | 0.67 | 0.53 | 0.84 |  |
|  | 10-14 Moderate depression | 1.34 | 0.96 | 1.86 |  | 1.20 | 0.84 | 1.71 |  |
|  | 15-19 Major depression, moderately severe | 1.03 | 0.57 | 1.85 |  | 0.77 | 0.42 | 1.41 |  |
|  | >20 Major depression, severe | 5.43 | 2.50 | 11.78 |  | 5.29 | 2.29 | 12.23 |  |
| Anxiety severity  (GAD-7) | <5 no anxiety | 1.00 |  |  | 0.005 | 1.00 |  |  | 0.012 |
|  | 5-9 Mild anxiety | 0.75 | 0.58 | 0.97 |  | 0.71 | 0.55 | 0.93 |  |
|  | 10-14 moderate anxiety | 1.43 | 0.93 | 2.20 |  | 1.16 | 0.73 | 1.83 |  |
|  | >15 severe anxiety | 1.90 | 1.07 | 3.37 |  | 1.79 | 0.97 | 3.28 |  |
|  |  |  |  |  |  |  |  |  |  |
| Total volume of ethanol (litres/year) | Non drinker | 1.00 |  |  | <0.001 | 1.00 |  |  | <0.001 |
|  | 0-1 | 0.49 | 0.39 | 0.61 |  | 0.59 | 0.46 | 0.74 |  |
|  | 2-4 | 0.86 | 0.65 | 1.13 |  | 0.89 | 0.66 | 1.20 |  |
|  | 5-9 | 0.63 | 0.45 | 0.88 |  | 0.60 | 0.42 | 0.87 |  |
|  | 10-19 | 0.97 | 0.67 | 1.41 |  | 0.97 | 0.64 | 1.46 |  |
|  | 20+ | 1.67 | 1.13 | 2.48 |  | 1.65 | 1.06 | 2.58 |  |
| CAGE score total | 0 | 1.00 |  |  | <0.001 | 1.00 |  |  | <0.001 |
|  | 1 | 0.96 | 0.70 | 1.33 |  | 0.91 | 0.65 | 1.28 |  |
|  | 2 | 2.17 | 1.62 | 2.91 |  | 2.22 | 1.61 | 3.06 |  |
|  | 3 | 1.25 | 0.82 | 1.91 |  | 1.04 | 0.66 | 1.64 |  |
|  | 4 | 4.24 | 2.68 | 6.72 |  | 3.45 | 2.06 | 5.78 |  |
| Current smoker | Never smoker | 1.00 |  |  | <0.001 | 1.00 |  |  | <0.001 |
|  | No, ex-smoker | 1.17 | 0.91 | 1.49 |  | 1.03 | 0.79 | 1.35 |  |
|  | Yes, Current smoker | 2.79 | 2.29 | 3.40 |  | 2.13 | 1.69 | 2.69 |  |
| Household financial situation | Not even enough money for food | 2.60 | 1.73 | 3.90 | <0.001 | 2.42 | 1.55 | 3.77 | 0.003 |
|  | Enough money for food | 1.08 | 0.85 | 1.37 |  | 0.93 | 0.73 | 1.20 |  |
|  | Enough money for food and clothes | 1.00 |  |  |  | 1.00 |  |  |  |
|  | Can afford to buy large domestic appliances | 0.69 | 0.55 | 0.88 |  | 0.89 | 0.69 | 1.15 |  |
|  | Can afford to buy a large new car | 0.60 | 0.33 | 1.10 |  | 1.03 | 0.55 | 1.93 |  |
|  | Have no financial constraints | 1.12 | 0.61 | 2.08 |  | 1.51 | 0.77 | 2.96 |  |
| Self-reported Hypertension | No | 1.00 |  |  | <0.001 | 1.00 |  |  | 0.018 |
|  | Yes | 0.72 | 0.60 | 0.86 |  | 0.79 | 0.65 | 0.96 |  |
| Self-reported High Cholesterol | No | 1.00 |  |  | <0.001 | 1.00 |  |  | <0.001 |
|  | Yes | 0.49 | 0.40 | 0.60 |  | 0.57 | 0.46 | 0.71 |  |
| Self-reported Myocardial Infarction | No | 1.00 |  |  | 0.331 | 1.00 |  |  | 0.084 |
|  | Yes | 0.82 | 0.55 | 1.23 |  | 0.70 | 0.46 | 1.06 |  |
| Self-reported heart failure | No | 1.00 |  |  | 0.056 | 1.00 |  |  | 0.008 |
|  | Yes | 0.77 | 0.58 | 1.01 |  | 0.68 | 0.51 | 0.91 |  |
| Self-reported atrial fibrillation | No | 1.00 |  |  | 0.247 | 1.00 |  |  | 0.165 |
|  | Yes | 0.71 | 0.39 | 1.30 |  | 0.66 | 0.36 | 1.22 |  |
| Self-reported Angina | No | 1.00 |  |  | 0.004 | 1.00 |  |  | 0.006 |
|  | Yes | 0.67 | 0.50 | 0.89 |  | 0.66 | 0.48 | 0.89 |  |
| Self-reported Stroke | No | 1.00 |  |  | 0.063 | 1.00 |  |  | 0.048 |
|  | Yes | 1.48 | 1.00 | 2.21 |  | 1.56 | 1.02 | 2.39 |  |
| Distance from clinic | 0-1.99km | 1.00 |  |  | 0.061 | 1.00 |  |  | 0.002 |
|  | 2-3.99km | 1.08 | 0.86 | 1.36 |  | 1.27 | 1.00 | 1.61 |  |
|  | 4+km | 1.29 | 1.03 | 1.61 |  | 1.52 | 1.20 | 1.93 |  |

*Denominator is all participants with a baseline interview

**Supplementary Table S4: Potential areas of comparison between Tromsø 7 and International Project on Cardiovascular Disease in Russia (IPCDR)**

| **Topic area** | **IPCDR technology** | **Tromsø 7 technology** | **Comments on protocol convergence** |
| --- | --- | --- | --- |
| Echocardiography | GE Vivid Q (GE Health care) | GE Vivid E9 (GE Health care) | Using same core protocol jointly developed. |
| Blood pressure | Omron 705 IT (OMRON Healthcare) | Dinamap (CARESCAPE V100 Monitor, GE Healthcare) | 3 measurements after two minutes seated rest, with variable cuff size |
| Height | Two measures with Seca® 217 portable stadiometer (Seca Limited) | 1 measure Jenix® height & weight scale DS-103 (Jenix Co, Ltd) |  |
| Weight | TANITA BC 418 body composition analyser (TANITA, Europe GmbH) | 1 measure Jenix® height & weight scale DS-103 (Jenix Co, Ltd) |  |
| Waist and hip circumference | Two measures using Seca® measuring tapes (Seca 201) (Seca Limited) | 1 measure Seca® measuring tapes (Seca 201) (Seca Limited) |  |
| Body composition | Bioimpedance (TANITA BC 418 body composition analyser) | DEXA (Lunar GE Prodigy Advance, GE Medical Systems) |  |
| Spirometry | Vitalograph pneumotrac 6800 (offered to 50% of participants) (Vitalograph®, UK) | Vmax® Encore (Sensormedics® Corporation, USA) |  |
| Pulse oximetry | Finger pulse oximeter (Onyx II 9550) (Nonin Medical Inc, USA) | Finger pulse oximeter (Onyx II 9550) (Nonin, Medical Inc, USA) |  |
| Grip strength | Jamar Digital Hand Dynamometer Plus+ (Patterson Medical, UK) | Jamar Digital Hand Dynamometer Plus+ (Patterson Medical, UK) | . |
| Standing balance (eyes open and eyes closed) | - | - | Standard Operating Procedures for standing balance for both studies based on National Health and Aging trends study([30](#_ENREF_30)) |
| ECG | Cardiax digital device (IMED ltd, Hungary) | Schiller AT-104 PC (Schiller, USA) | IPCDR and T7 digital output being automatically coded into Minnesota codes at the University of Glasgow |
| Physical activity | ActiHeart (CamNtech Ltd) worn for 5 days (offered to 50% of participants) | ActiGraph wGT3X-BT (ActiGraph Corp.) worn for 7 full days (day and night) plus Actiwave Cardio (CamNtech Ltd) worn for 27 hours | These are similar technologies with three-axis accelerometry used for all three devices. The ActiHeart and the Actiwave Cardio also include continuous heart rate measurements. |
| Biochemistry | - |  | Basic calibration of Lytech lab (Moscow) by UNN reference laboratory |
