# Supplementary material for: Know Your Heart: Rationale, design and conduct of a cross-sectional study of cardiovascular structure, function and risk factors in 4500 men and women aged 35-69 years from two Russian cities, 2015-18
Source: Wellcome Open Res. 2018 Dec 3;3:67. Originally published 2018 Jun 4. [Version 3] doi: 10.12688/wellcomeopenres.14619.3 (PMC6073094; doi:10.12688/wellcomeopenres.14619.3)
Supplement: Supplementary file 3 [file wellcomeopenres-3-16294-s0002.tgz › d9a03f66-5fce-45a3-8a2e-d18febd22997_Supplementary_file_3._Questionnaires_and_data_collection_tools.zip!/questionnaires_181121_publish_version2/181118_Baseline_survey_main_EN.docx]

|  |  |
| --- | --- |

**Baseline questionnaire**

International Project on Cardiovascular Disease in Russia

2015-2018

Published version 2.0 November 2018

Russia has one of the highest rates of cardiovascular disease in the world. The International Project on Cardiovascular Disease in Russia (IPCDR) was set up to understand the reasons for this. A substantial component of this study was the Know Your Heart Study devoted to characterising the nature and causes of cardiovascular disease in Russia by conducting large cross-sectional surveys in two Russian cities Novosibirsk and Arkhangelsk. The study population was 4542 men and women aged 35-69 years recruited from the general population. Fieldwork took place between 2015-18. There were two study components: 1) a baseline interview to collect information on socio-demographic characteristics and cardiovascular risk factors, usually conducted at home, and 2) a comprehensive health check at a primary care clinic which included detailed examination of the cardiovascular system.

The International Project on Cardiovascular Disease in Russia (IPCDR) project was funded in part by a Wellcome Trust Strategic Award [100217]. The project was also funded by the Arctic University of Norway, UiT in Tromsø; Norwegian Institute of Public Health; the Norwegian Ministry of Health and Social Affairs.

Development of the questionnaire was led by the London School of Hygiene & Tropical Medicine in collaboration with the Northern State Medical University, Arkhangelsk, Novosibirsk State Medical University, the Research Institute of Internal and Preventive Medicine - Branch of IC&G SB RAS, Novosibirsk and colleagues from other institutions.

This is a paper version questionnaire used for the baseline interview although the interview was administered electronically using Computer Assisted Personal Interviewing (CAPI) devices.

Further details about the study are available here: Know Your Heart: Rationale, design and conduct of a cross-sectional study of cardiovascular structure, function and risk factors in 4500 men and women aged 35-69 years from two Russian cities, 2015-18
Cook S, Malyutina S, Kudryavtsev AV, Averina M, Bobrova N, Boytsov S, Brage S, Clark T et al.

Content

[Module A. Socio-demographic factors	4](#_Toc512505986)

[Module B. Physical Activity	9](#_Toc512505991)

[Module C. Self-Reported Health	11](#_Toc512505992)

[Module D. Use of health care services.	14](#_Toc512505994)

[Module E. Depressions and Anxiety.	20](#_Toc512505995)

[Module F. Diet.	22](#_Toc512505996)

[Module G. Alcohol consumption.	24](#_Toc512505996)

[Module H. Smoking	33](#_Toc512505997)

[Module I. Household	35](#_Toc512505999)

[Module K. Psychosocial factors	38](#_Toc512506001)

[Section X. Interview context	41](#_Toc512506003)

[Section Y. Consent for the health check participation	42](#_Toc512506009)

Date of the interview__ __ / __ __ / __ __ __ __ Interviewer ID___Respondent ID________

| **Module A** | **SOCIO-DEMOGRAPHIC FACTORS** |
| --- | --- |

**I would like to begin by asking you some questions about yourself.**

| A1. | How old are you? _________Years |
| --- | --- |
| 97  98 | difficult to answer  refuse to answer |
|  |  |
| A2. | What is your date of birth?  Date of birth: __ __ / __ __ / __ __ __ __ |
| 97  98 | Difficult to answer  Refuse to answer |
| **A3.** | Interviewer! Please mark the gender of the respondent. |
| 1  2 | Male  Female |
|  |  |
| A4. | What is your nationality? *Region specific*: *Arkhangelsk/Novosibirsk.* *Please choose the single most appropriate answer.* |
| 1 2  3  4  5 | Russian  Ukranian/Tatar  Nenets/Ukraininan  Belorus/Uzbek  Other |
| 5a | Other. Specify .................... .................... |
| 97  98 | Difficult to answer  Refuse to answer |
|  |  |
| A5. | Please could you tell me where you were born? *Please choose the single most appropriate answer.* |
| 1 2 3 4 5 | Arkhangelsk city/Novosibirsk city  Arkhangelsk/Novosibirsk region  A different region of Russia  A part of the former Soviet Union outside Russia  Outside the former Soviet Union |
| 97  98 | Difficult to answer  Refuse to answer |
|  |  |
| **A6.** | **How long have you continuously lived in Arkhangelsk/Novosibirsk?** *Please choose the single most appropriate answer.* |
| 1  2  3  4  5 | Up to 12 months  One year to 5 years  From 5, up to 10 years  More than 10 years, but not your whole life  Since birth (excluding service in the army and temporary periods away) |
| 97  98 | Difficult to answer  Refuse to answer |
|  |  |
| **A7.** | **What is your current marital status? Are you:** *Please choose the single most appropriate answer.* |
| 1 2 3 4 5 | Living together in a registered marriage  Living together not in a registered marriage  Divorced or separated  Widower  Never married |
| 97  98 | Difficult to answer  Refuse to answer |
|  |  |
| **A8.** | **How many children do you have?** *Please choose the single most appropriate answer.* |
| 1  2  3  4  5 | 0  1  2  3  4 or more |
| 97  98 | Difficult to answer  Refuse to answer |
|  |  |

I would now like to ask you about your education and occupation.

| A9. | What is your level of education? *SHOW CARD 1. Please choose the single most appropriate answer.* | |
| --- | --- | --- |
| 1 2 3 4 5  6  7 | Incomplete secondary or lower  Complete secondary  Professional school (without secondary degree, PTU)  Professional school and secondary (e.g. PTU and secondary education)  Specialised secondary (e.g. medical, pedagogical college, technicum)  Incomplete higher  Higher | |
| 97  98 | Difficult to answer  Refuse to answer | |
|  |  | |
| **A10.** | **Are you studying at the moment (are you a student)?** | |
| 1  2 | Yes  No | |
| 97  98 | Difficult to answer  Refuse to answer | |
|  |  | |
| **A11.** | **Are you….** *Please choose the single most appropriate answer* | |
| 1 | Retired, except for retirement due to disabilty | |
| 2 | Retired due to disability | |
| 3 | None of the above | |
| 97  98 | Difficult to answer  Refuse to answer | |
|  |  | |
| **A12.** | **Are you in regular paid work?** | |
| 1 | Yes | |
| 2 | No *Go to A 14* | |
| 97 | Difficult to answer | |
| 98 | Refuse to answer | |
| **A13.** | **During the past 12 months how many months did you work on this job?**  Number of months…………………………………. *Go to A17* | |
|  |  | |
| 97  98 | Difficult to answer  Refuse to answer | |
|  |  | |
| **A14.** | **Are you…***Ask only those who are not in regular paid work (A12-NO)* | |
| 1 | In irregular paid work | |
| 2 | Unemployed, seeking work | |
| 3 | Unemployed, not seeking work | |
| 4 | Housewife | |
| 5  5a | Other  Other. Specify .................... .................... | |
| 6 | None of the above | |
| 97  98 | Difficult to answer  Refuse to answer | |
| **A15.** | **How long ago did you cease this regular employment?** *Please choose the single most appropriate answer.* | |
| 1  2  3  4  5  6 | Have never been in regular paid employment *Go to A17*  Within the past week  More than 1, up to 4 weeks ago  More than 1, up to 6 months ago  More than 6, up to 12 months ago  More than 1 year ago | |
| 97  98 | Difficult to answer  Refuse to answer | |
| **A16.** | **What was the main reason for ceasing regular paid employment?** *Please choose the single most appropriate answer.* | |
| 1 2 3 4 5  6  7  8  9  10  11  11a | Started to study  Retired  Could not find a job after finishing education  Business (organization) was shut down or was made redundant  A temporary job ended  Was asked to leave/was fired  Gave up voluntarily due to unsatisfactory work salary/work conditions  Always worked in temporary jobs  Gave up work because of ill health  Gave up work to take care of children, other people  Gave up my job for other reasons  Other. Specify .................... .................... | |
| 97  98 | Difficult to answer  Refuse to answer | |
|  |  | |
| I would now like to ask you some questions about your main regular employment over the past year or in the last period that you were working. | | |
|  |  | |
|  |  | |
| **A17.** | What was your main paid occupation during the past year? *Interviewer types in full description (e.g. teacher in primary school)*  Your paid occupation:................................................................. | |
| 97  98 | Difficult to answer  Refuse to answer | |
| **A18. Which of the descriptions best describe your work?** *SHOW CARD 2.* *Please choose the single most appropriate answer.* | | |
| 1 2 3 4 5 6 7  8  9  10  10a | Legislator, Senior official or Manager  Professional  Technician or associate professional  Clerk  Service or sales worker  Craft and related trades workers  Plant/machine operator or assembler  Elementary worker  Military/armed forces  Other. Specify .................... ...................  Other. Specify .................... .................... | |
| 97  98 | Difficult to answer  Refuse to answer | |
| **A19.** | **Have you missed any days of work due to your own ill health in the last 12 months?** | |
| 1  2 | Yes  No *Go to A21* | |
| 97  98 | Difficult to answer  Refuse to answer *Go to A21* | |
| **A20** | **Please specify how many days you have missed.** | |
| 1 | 1-3 days |  |
| 2 | 4-5 days |  |
| 3 | 6-10 days |  |
| 4 | 11+ days |  |
| 97 | Difficult to answer |  |
| 98 | Refuse to answer |  |
| A21 | Do you have currently officialy registered disability? | |
| 1  2 | Yes  No *Go to B1* | |
| 97  98 | Difficult to answer *Go to B1*  Refuse to answer *Go to B1* | |
|  |  | |
| A22. | How long ago were you registered? | |
| 1  2  3  4  5  6  97  98 | Less than 6 months ago  6 months to 12 months ago  More than 1, up to 5 years ago  More than 5, up to 10 years ago  More than 10 years ago but not my whole life  Have been disabled from birth  Difficult to answer  Refuse to answer | |
|  |  | |
| A23. | What is the class of the disability at the moment? | |
| 1 2 3 | Class 1  Class 2  Class 3 | |
| 97  98 | Difficult to answer  Refuse to answer | |

| **Module B** \| **PHYSICAL ACTIVITY** | |
| --- | --- |
| **B1.** | **Next questions will be related to your physical activity.**  **We would like to know the type and amount of physical activity involved in your work (only asked those who worked in the past 12 months)**  **Please choose what best corresponds to your present activities from the following four possibilities.** *SHOW CARD 3.* |
| 1  2  3  4 | **Sedentary occupation** - You spend most of your time sitting (such as in an office)  **Standing occupation -** You spend most of your time standing or walking. However, your work does not require intense physical effort (e.g. shop assistant, hairdresser,  guard, etc.)  **Physical work -** This involves some physical effort including handling of heavy objects and use of tools (e.g. plumber, cleaner, nurse, sports instructor, electrician,  carpenter, etc.)  **Heavy manual work -** This involves very vigorous physical activity including handling of very heavy objects (e.g. docker, miner, bricklayer, construction worker, etc.) |
| 97  98 | Difficult to answer  Refuse to answer |

| **B2.** | **In a typical week during the past 12 months, how many hours did you spend on each of the following activities?** (Put ‘0’ if none) |
| --- | --- |
| 1  2  3  4  5  6 | Walking, including walking to work, shopping and leisure  In summer, number of hours.................In winter, number of hours................  Cycling, including cycling to work and during leisure time  In summer, number of hours.................In winter, number of hours................  Gardening  In summer, number of hours.................In winter, number of hours................  Housework such as cleaning, washing, cooking, childcare  Hours per week.................  Do-it-yourself (painting, repairs)  Hours per week.................  Other physical exercise to keep fit such as aerobics, swimming, jogging, skating, skiing  In summer, number of hours.................In winter, number of hours................ |
| 97  98 | Difficult to answer  Refuse to answer |
| **B3.** | **In a typical week during the past year did you practise any of these activities vigorously enough to cause sweating or a faster heartbeat?** |
| 1 | Yes  If yes, for how many hours per week in total did you practise such vigorous physical activity? (Put ‘0’ if none)  Hours per week............................................. |
|  |  |
| 97  98 | Difficult to answer  Refuse to answer |
| **B4.** | **In a typical day during the past 12 months, how many floors of stairs did you climb up? (Put ‘0’ if none)** |
|  | Floors per day............................................. |
|  |  |
| 97  98 | Difficult to answer  Refuse to answer |

| **Module C** | **GENERAL HEALTH** |
| --- | --- |

This part of survey is related to your general health. This information will help to understand how do you feel, and you manage your usual tasks.

| **C1.** | **In general, would you say your health is**: *Please choose the single most appropriate answer.* | |
| --- | --- | --- |
|  |  | |
| 1 2  3  4  5 | Excellent  Very good  Good  Fair  Poor | |
|  | | |
|  | | **The following two questions are about activities you might do in a typical day. Does YOUR HEALTH NOW LIMIT YOU in these activities? If so, how much?** |
|  | |  |
| **C2.** | | **MODERATE ACTIVITIES, such as moving a table, pushing a vacuum cleaner, picking mushrooms or working in the garden:** *Please chosse the single most appropriate answer.* |
|  | |  |
| 1  2  3 | | Yes, limited a lot  Yes, limited a little  No, not limited at all |
|  | | |
| **C3.** | | **Climbing SEVERAL flights of stairs:** *Please choose the single most appropriate answer.* |
|  | |  |
| 1  2  3 | | Yes, limited a lot  Yes, limited a little  No, not limited at all |
|  | |  |
| **During the PAST 4 WEEKS have you had any of the following problems with your work or other regular activities AS A RESULT OF YOUR PHYSICAL HEALTH?** | | |
| **C4.** | | **ACCOMPLISHED LESS than you would like:** *SHOW CARD 4.* |
|  | |  |
| 1  2  3  4  5 | | All of the time  Most of the time  Some of the time  A little of the time  None of the time |
|  | |  |
| C5. | | **Were limited in the KIND of work or other activities:** *SHOW CARD 4.* |
|  | |  |
| 1  2  3  4  5 | | All of the time  Most of the time  Some of the time  A little of the time  None of the time |
|  | | |
| **During the PAST 4 WEEKS, have you had any of the following problems with your work or other regular daily activities AS A RESULT OF ANY EMOTIONAL PROBLEMS (such as feeling depressed or anxious)?** | | |
|  | |  |
| **C6.** | | **ACCOMPLISHED less than you would like:** *SHOW CARD 4.* |
|  | |  |
| 1  2  3  4  5 | | All of the time  Most of the time  Some of the time  A little of the time  None of the time |
|  | |  |
| **C7.** | | **Did work or other activities LESS CAREFULLY THAN USUAL:** *SHOW CARD 4.* |
|  | |  |
| 1  2  3  4  5 | | All of the time  Most of the time  Some of the time  A little of the time  None of the time |
|  | |  |
| **C8.** | | **During the PAST 4 WEEKS, how much did PAIN interfere with your normal work (including both work outside the home and housework?** |
|  | |  |
| 1  2  3  4  5 | | Not at all  A little bit  Moderately  Quite a bit  Extremely |
|  | | |
| The next three questions are about how you feel and how things have been DURING THE PAST 4 WEEKS. For each question, please give the one answer that comes closest to the way you have been feeling. How much of the time during the PAST 4 WEEKS.... | | |
|  | |  |
| C9. | | Have you felt calm and peaceful? *SHOW CARD 4* |
|  | |  |
| 1  2  3  4  5 | | All of the time  Most of the time  Some of the time  A little of the time  None of the time |
|  | |  |
| **C10.** | | **Did you have a lot of energy?** *SHOW CARD 4.* |
|  | |  |
| 1  2  3  4  5 | | All of the time  Most of the time  Some of the time  A little of the time  None of the time |
|  | |  |
| C11. | | **How much of the time during the PAST 4 WEEKS-have you felt downhearted and blue?** *SHOW CARD 4.* |
| 1  2  3  4  5 | | All of the time  Most of the time  Some of the time  A little of the time  None of the time |
| C12. | | **During the PAST 4 WEEKS, how much of the time has your PHYSICAL HEALTH OR EMOTIONAL PROBLEMS interfered with your social activities (like visiting with friends, relatives, etc.)?** *SHOW CARD 4.* |
|  | |  |
| 1  2  3  4  5 | | All of the time  Most of the time  Some of the time  A little of the time  None of the time |
|  | |  |

| **Module D** \| **USE OF HEALTH CARE SERVICES** | | | | | | | | | | | |
| --- | --- | --- | --- | --- | --- | --- | --- | --- | --- | --- | --- |
| D1. | **This module contains questions about use of medical services, presence of some diseases and use of medications.** **How many times did you use the following sources of health care in the last 12 months? (Please check number of times for each type of doctor)** Type of doctor \| Number of times visited in last 12 months \| \| \| \| \|; 1.GP \| 0 \| 1 \| 2 \| 3 \| 4 \| 5+; 2. Polyclinic cardiologist \| 0 \| 1 \| 2 \| 3 \| 4 \| 5+; 3. Other polyclinic specialist \| 0 \| 1 \| 2 \| 3 \| 4 \| 5+; 4. Hospital cardiologist \| 0 \| 1 \| 2 \| 3 \| 4 \| 5+; 5. Other hospital doctor \| 0 \| 1 \| 2 \| 3 \| 4 \| 5+ | | | | | | | | | | |
|  |  | | | | | | | | | | |
| **D2.** | **In the last 12 months, how many times have you been hospitalised (stayed in the hospital overnight)?**  Number of times.................................... If “0” **-** *Go to D4* | | | | | | | | | | |
| 97  98 | Difficult to answer  Refuse to answer | | | | | | | | | | |
| **D3.** | **For how many nights did you stay in hospital for each of those hospitalizations?** | | | | | | | | | | |
|  | **Hospitalization** | **Number of days/nights** | | | | | | | | | |
| 1  2  3  4  5  6  7  8  9  10 |  | .....................................  .....................................  .....................................  .....................................  .....................................  .....................................  .....................................  .....................................  .....................................  ..................................... | | | | | | | | | |
| 97  98 | Difficult to answer  Refuse to answer | | | | | | | | | | |
| **D4.** | **How many times have you called or someone called for you an ambulance in the last 12 months?** | | | | | | | | | | |
|  | Number of times.................. | | | | | | | | | | |
| 97  98 | Difficult to answer  Refuse to answer | | | | | | | | | | |
|  |  | | | | | | | | | | |
| **D5.** | **In the last 12 months, when you visited a doctor (GP, cardiologist any other medical professional), have you been advised to modify your lifestyle in any of the following ways to improve your health?** *Skip if D1, D2 and D4=0* | | | | | | | | | | |
|  |  |  | | | | | | | | | |
|  |  | | Yes | No | | Difficult to answer | | Refuse to answer | |  |  |
| **D5a.** | **Change diet** | | 1 | 2 | | 97 | | 98 | |  |  |
|  |  | |  |  | |  | |  | |  |  |
| **D5b.** | **Take exercise/sport** | | 1 | 2 | | 97 | | 98 | |  |  |
|  |  | |  |  | |  | |  | |  |  |
| **D5c.** | **Lose weight** | | 1 | 2 | | 97 | | 98 | |  |  |
|  |  | |  |  | |  | |  | |  |  |
| **D5d.** | **Stop smoking** | | 1 | 2 | | 97 | | 98 | |  |  |
|  |  | |  |  | |  | |  | |  |  |
| **D6.** | **How familiar are you with arterial hypertension (high blood pressure)?** | | | | | | | | | | |
| 1  2  3  4 | Not at all  I have only heard the term before  I know a little about it  I am very familiar with it | | | | | | | | | | |
| 97  98 | Difficult to answer  Refuse to answer | | | | | | | | | | |
| **D7.** | **Have you had your blood pressure checked by a doctor or other medical professional in the last 2 years?** | | | | | | | | | | |
| 1  2 | Yes *Go to D9*  No *Go to D8* | | | | | | | | | | |
| 97  98 | Difficult to answer *Go to D9*  Refuse to answer *Go to D9* | | | | | | | | | | |
|  |  | | | | | | | | | | |
| **D8.** | **Why you did not check your blood pressure by a doctor?** *Please choose all that apply.* | | | | | | | | | | |
| 1 | I don’t think having my blood pressure checked will have any positive impact on my health | | | | | | | | | | |
| 2 | I feel well so I don’t need to get checked | | | | | | | | | | |
| 3 | It is too far to travel to clinics | | | | | | | | | | |
| 4 | The waiting times at clinics are too long | | | | | | | | | | |
| 5 | Clinics are not open at times that are convenient | | | | | | | | | | |
| 6 | I don’t know where can get my blood pressure checked | | | | | | | | | | |
| 7 | I check my own blood pressure at home | | | | | | | | | | |
| 8  8a | Other  Other. Specify .................... .................... | | | | | | | | | | |
| 97  98 | Difficult to answer  Refuse to answer | | | | | | | | | | |
|  |  | | | | | | | | | | |
| **D9.** | **Which of the following is true?** *Please choose one option.* | | | | | | | | | |  |
| 1 | Most people with hypertension always feel symptoms | | | | | | | | | |  |
| 2  3 | Most people with hypertension feel symptoms some of the time  Most people with hypertension never feel symptoms | | | | | | | | | |  |
| 97  98 | Difficult to answer  Refuse to answer | | | | | | | | | |  |
| **D10.** | **Have you ever been told by a doctor (been diagnosed) that you have:** | | | | | | | | | |  |
|  |  | | | |  |  |  | |  | |  |
|  |  | | | | Yes | No | Difficult to answer | | Refuse to answer | |  |
| 1 | Arterial hypertension (high blood pressure). For women “except during pregnancy” | | | | 1 | 2 | 97 | | 98 | |  |
| 1a | Arterial hypertension during pregnancy (*women only*) | | | | 1 | 2 | 97 | | 98 | |  |
| 2 | High cholesterol level | | | | 1 | 2 | 97 | | 98 | |  |
| 3 | Myocardial Infarction/Heart attack | | | | 1 | 2 | 97 | | 98 | |  |
| 4 | Heart failure | | | | 1 | 2 | 97 | | 98 | |  |
| 5 | Atrial fibrillation | | | | 1 | 2 | 97 | | 98 | |  |
| 6 | Angina | | | | 1 | 2 | 97 | | 98 | |  |
| 7 | Stroke | | | | 1 | 2 | 97 | | 98 | |  |
| 8 | Diabetes | | | | 1 | 2 | 97 | | 98 | |  |
| 9 | Kidney disease | | | | 1 | 2 | 97 | | 98 | |  |
| 10 | Chronic bronchitis/COPD | | | | 1 | 2 | 97 | | 98 | |  |
| 11 | Cancer | | | | 1 | 2 | 97 | | 98 | |  |
| 12 | Asthma | | | | 1 | 2 | 97 | | 98 | |  |
| 13 | Rheumatoid arthritis | | | | 1 | 2 | 97 | | 98 | |  |
| 14 | Osteoarthritis (osteoarthritis) | | | | 1 | 2 | 97 | | 98 | |  |
| 15 | Migraine | | | | 1 | 2 | 97 | | 98 | |  |
| 16 | Depression | | | | 1 | 2 | 97 | | 98 | |  |
| 17 | Anxiety | | | | 1 | 2 | 97 | | 98 | |  |
|  |  | | | | | | | | | |  |
|  | *Note: Participants who report they have hypertension should be asked questions D11-D15, and D21-D25. Participants who report they have high cholesterol should be asked questions D16-D21. Participants who do not report hypertension or high cholesterol should go directly to D22*. | | | | | | | | | |  |
| **D11.** | **Were you prescribed medicines by a doctor or other medical professional to treat your arterial hypertension?** | | | | | | | | | |  |
| 1  2 | Yes  No *Go to D21 (unless report high cholesterol, then go to D16)* | | | | | | | | | |  |
| 97  98 | Difficult to answer *Go to D21 (unless report high cholesterol, then go to D16)*  Refuse to answer *Go to D21(unless report high cholesterol, then go to D16)* | | | | | | | | | |  |
| **D12.** | **Did you obtain all the medicines you were prescribed by a medical professional?** | | | | | | | | | |  |
| 1  2 | Yes *Go to D14*  No | | | | | | | | | |  |
| 97  98 | Difficult to answer  Refuse to answer *Go to D14* | | | | | | | | | |  |
|  |  | | | | | | | | | |  |
|  |  | | | | | | | | | |  |
|  |  | | | | | | | | | |  |
|  |  | | | | | | | | | |  |
| **D13.** | **What were the reasons for not obtaining all prescribed medicines?** *Please choose all that apply.* | | | | | | | | | |  |
| 1 | The prescribed medicines were not available at the pharmacy | | | | | | | | | |  |
| 2 | The prescribed medicines are too expensive | | | | | | | | | |  |
| 3 | The prescribed medicines are ineffective | | | | | | | | | |  |
| 4 | The prescribed medicines have harmful side effects | | | | | | | | | |  |
| 5 | I prefer to treat my high blood pressure with other pharmaceuticals that are recommended by friends/relatives | | | | | | | | | |  |
| 6 | I prefer to treat my high blood pressure with other pharmaceuticals that are recommended by the pharmacist | | | | | | | | | |  |
| 7 | I prefer to treat my high blood pressure with alternative (non-pharmaceutical) medicines | | | | | | | | | |  |
| 8  8a | Other  Other. Specify .................... .................... | | | | | | | | | |  |
| 97  98 | Difficult to answer  Refuse to answer | | | | | | | | | |  |
|  |  | | | | | | | | | |  |
| **D14.** | **Do you take your prescribed medications every day?** | | | | | | | | | |  |
| 1  2  3 | Yes *Go to D16*  No  Did not obtain any medication | | | | | | | | | |  |
| 97  98 | Difficult to answer  Refuse to answer | | | | | | | | | |  |
| **D15.** | **Why you do not take prescribed medication every day?** *Please choose all that apply* | | | | | | | | | |  |
| 1 | I do not have high blood pressure any more | | | | | | | | | |  |
| 2 | I did not know it is necessary to take the medication every day | | | | | | | | | |  |
| 3 | My doctor told me to take it only when I have symptoms | | | | | | | | | |  |
| 4 | I cannot afford to take it every day | | | | | | | | | |  |
| 5 | I forget to take it every day | | | | | | | | | |  |
| 6 | I stop taking it when I feel worse | | | | | | | | | |  |
| 7 | I stop taking it when I feel better | | | | | | | | | |  |
| 8 | Taking this medication every day would have harmful side effects | | | | | | | | | |  |
| 9 | I prefer to use medication that I purchased without a prescription | | | | | | | | | |  |
| 10  10a | Other  Other. Specify .................... .................... | | | | | | | | | |  |
| 97  98 | Difficult to answer  Refuse to answer  *If participant reported having been told by a doctor that they have high cholesterol, proceed to question D16.* | | | | | | | | | |  |
|  |  | | | | | | | | | |  |
| **D16.** | **Were you prescribed medicines by a medical professional to treat your cholesterol?** | | | | | | | | | |  |
| 1  2 | Yes  No *Go to D21* | | | | | | | | | |  |
| 97  98 | Difficult to answer *Go to D21*  Refuse to answer *Go to D21* | | | | | | | | | |  |
|  |  | | | | | | | | | |  |
|  |  | | | | | | | | | |  |
| **D17.** | **Did you obtain all the medicines you were prescribed by a medical professional?** | | | | | | | | | |  |
| 1  2 | Yes *Go to D19*  No | | | | | | | | | |  |
| 97  98 | Difficult to answer  Refuse to answer *Go to D19* | | | | | | | | | |  |
| **D18.** | **What were the reasons for not obtaining all prescribed medicines?** *Please choose all that apply* | | | | | | | | | |  |
| 1 | The prescribed medicines were not available at the pharmacy | | | | | | | | | |  |
| 2 | The prescribed medicines are too expensive | | | | | | | | | |  |
| 3 | The prescribed medicines are ineffective | | | | | | | | | |  |
| 4 | The prescribed medicines have harmful side effects | | | | | | | | | |  |
| 5 | I prefer to treat my high cholesterol with other pharmaceuticals that are recommended by friends/relatives | | | | | | | | | |  |
| 6 | I prefer to treat my high cholesterol with other pharmaceuticals that are recommended by the pharmacist | | | | | | | | | |  |
| 7 | I prefer to treat my high cholesterol with alternative (non-pharmaceutical) medicines. | | | | | | | | | |  |
| 8  8a | Other  Other. Specify .................... .................... | | | | | | | | | |  |
| 97  98 | Difficult to answer  Refuse to answer | | | | | | | | | |  |
|  |  | | | | | | | | | |  |
| **D19.** | **Do you take your prescribed cholesterol medication every day?** | | | | | | | | | |  |
| 1  2  3 | Yes *Go to D21*  No  Did not obtain any medication | | | | | | | | | |  |
| 97  98 | Difficult to answer  Refuse to answer *Go to D21* | | | | | | | | | |  |
|  |  | | | | | | | | | |  |
| **D20.** | **What was the reason for not taking prescribed medication every day?** *Please choose all that apply* | | | | | | | | | |  |
| 1  2  3  4  5  6  7  8  9  10  10a | I do not have high cholesterol any more  I did not know it is necessary to take the medication every day  My doctor told me to take it only when I have symptoms  I cannot afford to take it every day  I forget to take it every day  I stop taking it when I feel worse  I stop taking it when I feel better  Taking this medication every day would have harmful side effects  I prefer to use medication that I purchased without a prescription  Other  Other. Specify .................... .................... | | | | | | | | | |  |
| 97  98 | Difficult to answer  Refuse to answer | | | | | | | | | |  |
|  |  | | | | | | | | | |  |
|  |  | | | | | | | | | |  |
|  |  | | | | | | | | | |  |
| **D21.** | **Aside from any prescribed medicines, do you purchase pharmaceutical medicines that are not prescribed by a medical professional (e.g. suggested by the pharmacist or family/friend), to treat your hypertension or high cholesterol?** | | | | | | | | | |  |
| 1  2 | Yes  No | | | | | | | | | |  |
| 97  98 | Difficult to answer  Refuse to answer | | | | | | | | | |  |
| **D22.** | **Are you aware of the current polyclinic-based dispansarisation program in Russia?** | | | | | | | | | |  |
| 1  2 | Yes  No *Go to E1* | | | | | | | | | |  |
| 97  98 | Difficult to answer *Go to E1*  Refuse to answer *Go to E1* | | | | | | | | | |  |
| **D23.** | **Have you received an invitation to participate in dispansarisation?** | | | | | | | | | |  |
| 1  2 | Yes  No *Go to E1* | | | | | | | | | |  |
| 97  98 | Difficult to answer *Go to E1*  Refuse to answer *Go to E1* | | | | | | | | | |  |
| **D24.** | **Did you (or do you intend to) attend this dispansarisation?** | | | | | | | | | |  |
| 1  2 | Yes *Go to E1*  No | | | | | | | | | |  |
| 97  98 | Difficult to answer  Refuse to answer *E1* | | | | | | | | | |  |
| **D25.** | **What is the reason for this decision?** *Please choose all that apply.* | | | | | | | | | |  |
| 1  2  3  4  5  6  7  7a | I don’t believe attending dispansarisation will have any positive effect on my health  I feel well so I don’t need to get checked  It is too far to travel to dispansarisation clinics  The waiting times at dispansarisation clinics are too long  Dispansarisation clinics are not open at times that are convenient  I do not know where I could attend dispansarisation  Other  Other. Specify .................... .................... | | | | | | | | | |  |
| 97  98 | Difficult to answer  Refuse to answer | | | | | | | | | |  |

| **Module E** \| **DEPRESSION AND ANXIETY** **Now I would like to talk to you about your feelings in the past 2 weeks.** **Over the last 2 weeks, how often have you been bothered by any of the following problems?** *SHOW CARD 5.* | | Not at all | Several days | More than half of the days | Nearly every day |
| --- | --- | --- | --- | --- | --- |
| **E1.** | Little interest or pleasure in doing things | 0 | 1 | 2 | 3 |
| **E2.** | Feeling down, depressed or hopeless | 0 | 1 | 2 | 3 |
| **E3.** | Trouble falling or staying asleep, or sleeping too much | 0 | 1 | 2 | 3 |
| **E4.** | Feeling tired or having little energy | 0 | 1 | 2 | 3 |
| **E5.** | Poor appetite or overeating | 0 | 1 | 2 | 3 |
| **E6.** | Feeling bad about yourself- or that you are a failure or have let yourself or your family down | 0 | 1 | 2 | 3 |
| **E7.** | Trouble concentrating on things, such as reading the newspaper or watching television | 0 | 1 | 2 | 3 |
| **E8.** | Moving or speaking so slowly that other people have noticed. Or the opposite- being so fidgety or restless that you have been moving around a lot more than usual | 0 | 1 | 2 | 3 |
| **E9.** | Thoughts that you would be better off dead or of hurting yourself in some way | 0 | 1 | 2 | 3 |
| **E10.** | How difficult have these problems made it for you to do your work, take care of things at home, or get along with other people? | Not at all difficult | Somewhat difficult | Very difficult | Extremely difficult |
|  |  | 0 | 1 | 2 | 3 |

| **Over the last 2 weeks, how often have you been bothered by any of the following problems?** | | Not at all | Several days | More than half of the days | Nearly every day |
| --- | --- | --- | --- | --- | --- |
| **E11.** | Feeling nervous, anxious, or on edge | 0 | 1 | 2 | 3 |
| **E12.** | Not being able to sleep or control worrying | 0 | 1 | 2 | 3 |
| **E13.** | Worrying too much about different things | 0 | 1 | 2 | 3 |
| **E14.** | Trouble relaxing | 0 | 1 | 2 | 3 |
| **E15.** | Being so restless that it is hard to sit still | 0 | 1 | 2 | 3 |
| **E16.** | Becoming easily annoyed or irritable | 0 | 1 | 2 | 3 |
| **E17.** | Feeling afraid, as if something awful might happen | 0 | 1 | 2 | 3 |
| **E18.** | How difficult have these problems made it for you to do your work, take care of things at home, or get along with other people? | Not at all difficult | Somewhat difficult | Very difficult | Extremely difficult |
|  |  | 0 | 1 | 2 | 3 |

| **Module F** | **DIET** |
| --- | --- |

**I would like now to ask you about your dietary habits.**

| **F1.** | **What type of bread do you USUALLY choose?** *Please choose all that apply.* | | | | | | |
| --- | --- | --- | --- | --- | --- | --- | --- |
| 1  2  3  4  5  5a | No bread at all *Skip to F4*  Dark bread  White bread  White bread, coarse grain  Other  Other. Specify .................... .................... | | | | | | |
| 97  98 | Difficult to answer  Refuse to answer | | | | | | |
| **F2.** | **What type of fats do you USUALLY use on your bread?** *Please choose all that apply.* | | | | | | |
| 1  2  3  4  5  6 | None  Minarine  Vegetable margarine  Butter  Blended spread  Lard | | | | | | |
| 97  98 | Difficult to answer (DA)  Refuse to answer (RA) | | | | | | |
| **F3.** | **How often have you been eating the following foods with bread the PAST WEEK (not as a main course but as an appetizer/sandwich)?** | | | | | | |
| **Times a week** | |  |  |  |  |  |  |
| Cheese | | 0 | 1-2 | 3-4 | 5-7 | DA 97 | RA98 |
| Meat | | 0 | 1-2 | 3-4 | 5-7 | DA 97 | RA98 |
| Fish | | 0 | 1-2 | 3-4 | 5-7 | DA 97 | RA98 |
| Vegetables (excluding potatoes) | | 0 | 1-2 | 3-4 | 5-7 | DA 97 | RA98 |

| **F4.** | **How often have you been eating the following kinds of hot meals the PAST WEEK?** | | | | | | |
| --- | --- | --- | --- | --- | --- | --- | --- |
| **Times a week** | |  |  |  |  |  |  |
| Meat | | 0 | 1-2 | 3-4 | 5-7 | DA 97 | RA98 |
| Fish | | 0 | 1-2 | 3-4 | 5-7 | DA 97 | RA98 |
| Vegetable dishes (excluding potatoes) | | 0 | 1-2 | 3-4 | 5-7 | DA 97 | RA98 |
| Legumes (beans, peas) | | 0 | 1-2 | 3-4 | 5-7 | DA 97 | RA98 |

| **F5.** | | **What kind of fats do you use for cooking?** *Please choose all that apply.* | | | | | | | | | | | | | | | |  |
| --- | --- | --- | --- | --- | --- | --- | --- | --- | --- | --- | --- | --- | --- | --- | --- | --- | --- | --- |
| 1 | | None | | | | | | | | | | | | | | | |  |
| 2 | | Vegetable margarine | | | | | | | | | | | | | | | |  |
| 3 | | Margarine | | | | | | | | | | | | | | | |  |
| 4 | | Butter | | | | | | | | | | | | | | | |  |
| 5 | | Blended spread | | | | | | | | | | | | | | | |  |
| 6 | | Lard | | | | | | | | | | | | | | | |  |
| 7 | | Olive oil | | | | | | | | | | | | | | | |  |
| 8 | | Sunflower oil or other vegetable oil (e.g.corn oil or hemp oil) | | | | | | | | | | | | | | | |  |
| 9  9a | | Other  Other. Specify .................... .................... | | | | | | | | | | | | | | | |  |
| 97  98 | | Difficult to answer  Refuse to answer | | | | | | | | | | | | | | | |  |
| **F6.** | | **How often have you been eating vegetables (excluding potatoes) as accompaniments to the hot meals the PAST WEEK. Please exlude vegetables which you mentioned above?** | | | | | | | | | | | | | | | |  |
| **Times a week** | | | | |  | |  | |  | | |  | |  | |  | |  |
| Salad or raw vegetables | | | | | 0 | | 1-2 | | 3-4 | | | 5-7 | | DA 97 | | RA98 | |  |
| Boiled vegetables | | | | | 0 | | 1-2 | | 3-4 | | | 5-7 | | DA 97 | | RA98 | |  |
| Sauteed vegetables | | | | | 0 | | 1-2 | | 3-4 | | | 5-7 | | DA 97 | | RA98 | |  |
|  | | | | |  | |  | |  | | |  | |  | |  | |  |
| **F7.** |  | | **How much fruit and berries do you usually eat during a day/week 1 piece/100 grams?** | | | | | | | | | | | | | | | |
| 1  2  3  4  5  6  7  8 |  | | None  1-2 per week  3-4 per week  5-6 per week  1-2 per day  3-4 per day  5-6 per day  More than 6 per day | | | | | | | | | | | | | | | |
| 97  98 |  | | Difficult to answer  Refuse to answer | | | | | | | | | | | | | | | |
| **F8.** |  | | **How often do you usually drink the following drinks?** | | | | | | | | | | | | | | | |
|  |  | |  | | | | | | | | | | | | | | | |
|  | | | | Never | | 1-3 times a month | | 1-6 times a week | | Once a day | 2-3 times a day | | 4+ times a day | | DA 97 | | RA98 | |
|  | | | |  | |  | |  | |  |  | |  | |  | |  | |
| Fruit juice | | | | 1 | | 2 | | 3 | | 4 | 5 | | 6 | | DA 97 | | RA98 | |
| Sodas / soft drinks with sugar (exclude diet cola) | | | | 1 | | 2 | | 3 | | 4 | 5 | | 6 | | DA 97 | | RA98 | |
| Tea | | | | 1 | | 2 | | 3 | | 4 | 5 | | 6 | | DA 97 | | RA98 | |
| Coffee  Energy drinks (e.g. Red bull) | | | | 1  1 | | 2  2 | | 3  3 | | 4  4 | 5  5 | | 6  6 | | DA 97  DA97 | | RA98  RA98 | |

| **Module G** | **ALCOHOL CONSUMPTION** |
| --- | --- |

Now I will ask you about your alcohol consumption in the past 12 months. We ask these questions everyone even those who do not drink, drink little a bit or drink only on holidays such as New Year or at the birthday. Please think about any occasions you could consume alcohol beverages.

**G1 – G10. For each type of drink listed in the left hand column, please indicate how often each was usually drunk in the last 12 months.** *SHOW CRAD 6.*

|  | Every day or more often | Nearly every day | 3-4 times per week | Once or twice a week | 1-3 times a month | A few times a year | Never or almost never | Difficult to answer | Refuse to answer |
| --- | --- | --- | --- | --- | --- | --- | --- | --- | --- |
| **G1.** Any alcohol | 1 | 2 | 3 | 4 | 5 | 6 | 7 | 97 | 98 |

***If respondent answers 98 go to G29***

| **G2.** Beer | 1 | 2 | 3 | 4 | 5 | 6 | 7 | 97 | 98 |
| --- | --- | --- | --- | --- | --- | --- | --- | --- | --- |
| **G3.** Wine (not home produced) | 1 | 2 | 3 | 4 | 5 | 6 | 7 | 97 | 98 |
| **G4.** Fortified wine (e.g. port wine) | 1 | 2 | 3 | 4 | 5 | 6 | 7 | 97 | 98 |
| **G5.** Spirits (vodka, cognac, whisky, gin, rum, etc.) | 1 | 2 | 3 | 4 | 5 | 6 | 7 | 97 | 98 |
| **G6.** Ethanol-containing substances (These are substances not intended for drinking, including eau de colognes and medicinal tinctures as well as other things. They may be found in shops, chemists and kiosks) | 1 | 2 | 3 | 4 | 5 | 6 | 7 | 97 | 98 |
| **G7.**Spirit not intended for drinking | 1 | 2 | 3 | 4 | 5 | 6 | 7 | 97 | 98 |
| **G8.** Samogon | 1 | 2 | 3 | 4 | 5 | 6 | 7 | 97 | 98 |
| **G9.** Homemade wine, braga | 1 | 2 | 3 | 4 | 5 | 6 | 7 | 97 | 98 |
| **G10.** Alcoholic cocktails( premixed bottles) | 1 | 2 | 3 | 4 | 5 | 6 | 7 | 97 | 98 |

*If respondent answers 7, 97 or 98 to ALL questions G1-G10 go to G28*

*If respondent answers 7 or 98 on G2 or G3 or G5, G6, G7, G8 according questions on the amount of these beverages should be skipped.*

| G11. | During last 12 months how much beer did you usually drink on one occasion? (‘occasion’ means a single continuous period of drinking). *Please choose the single most appropriate answer.* |
| --- | --- |
| 1 2 3 4 5 | Never drinks beer  1 bottle (0.5l) or less  2-4 bottles (0.5l)  5-6 bottles (0.5l)  More than 6 bottles(0.5l) |
| 97  98 | Difficult to answer  Refuse to answer |
|  |  |
| G12. | During last 12 months how much wine did you usually drink on one occasion? *Please choose the single most appropriate answer.* |
| 1 2 3 4 5 6 | Never drinks wine  Up to 200g  Between 200 - 400g  Between 400 - 600g  Between 600 - 1000g  More than 1 litre |
| 97  98 | Difficult to answer  Refuse to answer |
|  |  |
| G13. | During last 12 months how much spirits, such as vodka or other spirits, do you usually drink on one occasion? *Please choose the single most appropriate answer.* |
| 1 2 3 4 5 6 7 8 | Never drinks spirits  Up to 50g  Between 50 – 100g  Between 100 - 200g  Between 200 - 300g  Between 300 - 400g  Between 400 - 500g  More than 500g |
| 97  98 | Difficult to answer  Refuse to answer |
|  |  |
| G14. | During last 12 months what was the maximum quantity of beer ever drunk on one occasion? *Please choose the single most appropriate answer.* |
| 1 2 3 4 5 | Never drinks beer  1 bottle (0.5l) or less  2-4 bottles (0.5l)  5-6 bottles (0.5l)  More than 6 bottles (0.5l) |
| 97  98 | Difficult to answer  Refuse to answer |
|  |  |
|  |  |
|  |  |
|  |  |
|  |  |
| G15. | During last 12 months what was the maximum quantity of wine ever drunk on one occasion? *Please choose the single most appropriate answer.* |
| 1 | Never drinks wine |
| 2 | Up to 200g |
| 3 | Between 200 - 400g |
| 4 5 6 | Between 400 - 600g  Between 600 - 1000g  More than 1 litre |
| 97  98 | Difficult to answer  Refuse to answer |
|  |  |
| G16. | During last 12 months what was the maximum quantity of spirits ever drunk on one occasion? *Please choose the single most appropriate answer.* |
| 1  2  3  4  5  6  7  8 | Never drinks spirits  Up to 50g  Between 50 – 100g  Between 100 - 200g  Between 200 - 300g  Between 300 - 400g  Between 400 - 500g  More than 500g |
| 97  98 | Difficult to answer  Refuse to answer |
|  |  |
| G17. | Do you ever drink large quantities of spirits without also eating some food at the same sitting? *Please choose the single most appropriate answer.* |
| 1 2 3 | Always  Sometimes  Rarely/never |
| 97  98 | Difficult to answer  Refuse to answer |
|  |  |
| G18. | How often do you become excessively drunk? *SHOW CARD 7. Please choose the single most appropriate answer.* |
| 1 2 3 4 5 6  7 | Every day  Several times a week  Once a week  Several times a month  Once a month  Less than once a month  Never or almost never |
| 97  98 | Difficult to answer  Refuse to answer |
|  |  |
| G19. | Do you ever drink alcohol before noon? *Please choose the single most appropriate answer.* |
| 1 2 3 | No  Yes, occasionally  Yes, frequently |
| 97  98 | Difficult to answer  Refuse to answer |
|  |  |
| G20. | How often do you have a hangover? *Please choose the single most appropriate answer.SHOW CARD 7.* |
| 1 | Every day |
| 2 3 4 5 6 7 | Several times a week  Once a week  Several times a month  Once a month  Less than once a month  Never or almost never |
| 97  98 | Difficult to answer  Refuse to answer |
|  |  |
| G21. | How often do you fail to fulfil your family or personal obligations due to drinking alcohol? *Respondents should be reminded that the time-period in this question is last 12 months. SHOW CARD 7.* |
| 1 2 3 4 5 6 7 | Every day  Several times a week  Once a week  Several times a month  Once a month  Less than once a month  Never or almost never |
| 97  98 | Difficult to answer  Refuse to answer |
|  |  |
| G22. | Do you ever go to sleep at night with your clothes on because of being drunk? *SHOW CARD 7. Please choose the single most appropriate answer.* |
| 1 2 3 4 5 6 7 | Every day  Several times a week  Once a week  Several times a month  Once a month  Less than once a month  Never or almost never |
| 97  98 | Difficult to answer  Refuse to answer |
|  |  |

**I would now like to ask you about episodes of ‘zapoi’ in your life.**

**By ‘zapoi’, I mean a period of continuous drunkenness of several days or more during which the person does not work and is withdrawn from normal life.**

| G23.  1  2  97  98 | | | **Have you had episodes of zapoi in the past year?**  Yes  No *Go to G31*  Difficult to answer *Go to G31*  Refuse to answer *Go to G31* |
| --- | --- | --- | --- |
|  | | | |
|  | | | |
| **G24.** | | | **How many episodes did you have last year** |
| 1 2 3  4 | | | 1  2-4  5-9  10 or mor*e* |
| 97  98 | | | Difficult to answer *Go to G31*  Refuse to answer *Go to G31* |
| **G25.** | | | Have you had one or more episodes of zapoi in the past **month**? |
| 1 2 | | | Yes  No |
| 97  98 | | | Difficult to answer  Refuse to answer |
| **G26.** | | | **How long does a typical episode last?** |
| 1 2 3  4 | | | 2-3 days  4-5 days  6-9 days  10 and more days |
| 97  98 | | | Difficult to answer  Refuse to answer |
|  | | |  |
| G27. | | | During your most recent episode of zapoi, did you drink ethanol-containing substances? Here we mean any alcoholic substances other than those intended for drinking. |
| 1 2 | | | Yes *Go to G31*  No *Go to G31* |
| 97  98 | | | Difficult to answer *Go to G31*  Refuse to answer *Go to G31* |
|  | | |  |
|  | | | *QUESTIONS FOR CURRENT NON DRINKERS (abstainers in the past 12 months).* |
|  | | |  |
| G28. | | | Have you ever drunk alcohol in your life other than on a few occasions? |
| 1 2 | | | Yes  No |
| 97  98 | | | Difficult to answer *Go to H1*  Refuse to answer *Go to H1* |
|  | | |  |
| G29. | | | When did you stop drinking alcohol? |
| 1  2  3  4 | | | Up to 6 months ago  More than 6, up to 12 months ago  More than 1, up to 5 years ago  More than 5 years ago |
| 97  98 | | | Difficult to answer  Refuse to answer |
|  | | |  |
|  | | |  |
|  | | |  |
|  | | |  |
|  | | |  |
|  | | |  |
| G30. | | | Why did you stop drinking alcohol? *Please choose all applicable* |
| 1 | | | I was afraid of losing my job |
| 2 | | | Advised by doctor to stop |
| 3 | | | After treatment for alcohol problems |
| 4 | | | Felt too ill to drink |
| 5 | | | Pressure from or influence of my family or friends |
| 6 | | | Financial reasons |
| 7  7a | | | I decided I don’t want to drink alcohol any more for other health-/illness-related reasons.  Other. Specify .................... .................... |
| 8  8a | | | I decided I don’t want to drink alcohol any more for other non health-/illness-related reasons.  Other. Specify .................... .................... |
| 97  98 | | | Difficult to answer  Refuse to answer |
|  | | |  |
| G31 | | | Have you ever had help or advice from a doctor, narcologist, social worker or some other professional for an alcohol problem? |
| 1 2 | | | Yes  No *Go* to G37 |
| 97  98 | | | Difficult to answer *Go* to G37  Refuse to answer *Go* to G37 |
|  | | |  |
| G32. | | | What kind of help/advice did you receive? *Please choose all that apply.* |
| 1 2  3  4  5  6  7  8 | | | Narcological help in state clinic (detoxification, alcohol withdrawal treatment)  Narcological help in private clinic or at home (e.g. medicalized help, IV drip)  Help from psychologist  Psychotherapy  Behavioural therapy  Group therapy (12 steps, AAs)  Coding  Help from traditional healer |
| 97  98 | | | Difficult to answer  Refuse to answer |
|  | | |  |
| G33. | | | Did you get such help or advice in the past year? |
| 1 2 | | | Yes  No |
| 97  98 | | | Difficult to answer  Refuse to answer |
|  | | |  |
| G34. | | | Have you ever attended (or were referred) the narcology dispensary? |
| 1 2 | | | Yes  No |
| 97  98 | | | Difficult to answer  Refuse to answer |
|  |  | | |
|  |  | | |
| G35. | When did you start consuming ethanol-containing substances? *Please choose the single most appropriate answer.* | | |
| 1  2  3  4 | Within the past month  Within past 6 months  Within the last year  More than a year ago | | |
| 97  98 | Difficult to answer  Refuse to answer | | |
| G36. | What ethanol-containing substances do you drink? Region specific. *Please choose all that apply.* | | |
| 1  2  3  4  5  6  7  8  9  10  10a | Infusion of hawthorn  Troyar  Spirits (technical, medical or other)  Composition  Troynoy cologne  “Pepper” lotion  “Juniper” lotion  “Clear light” lotion Windows cleaning liquid, other cleaners  Other types of liquids containing spirits  Other. Specify .................... .................... | | |
| 97  98 | Difficult to answer  Refuse to answer | | |
|  |  | | |
|  | | G37. In the last 12 months, have you had any of the following experiences? \| \| Yes \| No \| Difficult to answer \| Refuse to answer; a \| Have you ever felt you should cut down on your drinking? \| 1 \| 2 \| 97 \| 98; b \| Have people ever annoyed you by criticising your drinking? \| 1 \| 2 \| 97 \| 98; c \| Have you ever felt bad or guilty about your drinking? \| 1 \| 2 \| 97 \| 98; d \| Have you ever had a drink first thing in the morning to steady your nerves or get rid of a hangover? \| 1 \| 2 \| 97 \| 98 | |
|  | | | |
|  | | | |
|  | |  | |

| **Module H** | **SMOKING** |
| --- | --- |

I will now ask you some questions concerning your smoking habits.

| H1. | Are you a current smoker? *Please choose most appropriate answer.* |
| --- | --- |
| 1 2 3 | Never smoked *Go to I1*  No, ex-smoker *Go to H2*  Yes, a current-smoker *Go to H3* |
| 97  98 | Difficult to answer *Go to H3*  Refuse to answer *Go to I1* |
| H2. | How many years ago did you stop smoking regularly? *Please choose most appropriate answer.* |
| 1 2 3 4 | Up to 1 year ago  More than 1, up to 5 years ago  More than 5, up to 10 years ago  More than 10 years ago |
| 97  98 | Difficult to answer  Refuse to answer *Go to I1* |
| H3. | What do/did you smoke most often? *Please choose most appropriate answer.* |
| 1 2 3 4  4a | Papyrosi  Filtered cigarettes  Unfiltered cigarettes  Other  Other. Specify .................... .................... |
| 97  98 | Difficult to answer  Refuse to answer |
| H4. | When you smoke/smoked, how many per day is/was usual? *Please choose most appropriate answer.* |
| 1 2 3 | Up to 10  More than 10, up to 20  More than 20 |
| 97  98 | Difficult to answer  Refuse to answer |
| H5. | How old were you when you started smoking regularly?________Years |
|  | |
| 97  98 | Difficult to answer  Refuse to answer |
|  |  |
| H6. | Have you ever been advised by medical professional (your GP, cardiologist, any other physician) to stop smoking? |
| 1  2 | Yes  No *Go to I1* |
| 97  98 | Difficult to answer  Refuse to answer |
| **H7.** | **Was any assistance offered?** |
| 1  2 | Yes  No *Go to I1* |
| 97  98 | Difficult to answer  Refuse to answer |
|  |  |
| **H8.** | **What kind of assistance was offered? Please choose all that apply** |
| 1 | Provision of nicotine replacement therapy |
| 2 | Referral to the behavioral therapy |
| 3 | 12 steps programmes |
| 4 | Coding |
| 5 | Material motivation |
| 6  6a | Other  Other. Specify .................... .................... |
| 97  98 | Difficult to answer  Refuse to answer |
|  |  |
|  |  |

| **Module I** | **HOUSEHOLD** |
| --- | --- |

I would now like to ask you some additional questions about your household and people who live in it.

| I1. | When did you move to the current address? |
| --- | --- |
|  | |
| 1 2  3  4 | Less than 6 months ago  More than 6, up to 12 months ago  More than 1 year, up to 2 years ago  More than 2 years ago |
| 97  98 | Difficult to answer  Refuse to answer |
| I2. | How many people currently live in this household (excluding you)? _____People |
|  | |
| 97  98 | Difficult to answer  Refuse to answer |

*Interviewer! This table excludes the respondent*

*Interviewer! Where options are given, please choose the appropriate response*

Relationship to you

Age

(yrs)

M

F

M

F

M

F

M

F

M

F

M

F

M

F

M

F

**I3. I am now going to ask you some questions about people living in your household**

**Education codes:**

1 Incomplete secondary

2 Complete secondary

3 Professional w/t secondary

4 Professional with secondary

5 Specialized secondary

6 Incomplete higher

7 Higher

8 Not applicable

97 Difficult to answer

98 Refuse to answer

**Relationship codes:**

1 Spouse or partner

2 Parent

3 Brother

4 Sister

5 Daughter

6 Daughter in law

7 Son

8 Son in law

9 Grandchild

10 Other relatives

11 Unrelated people

97 Difficult to answer

98 Refuse to answer

**Contribution to income**

**codes:**

1 Yes

2 No

97 Difficult to answer

98 Refuse to answer

**Codes for age**

997 Difficult to answer

998 Refuse to answer

Education

Contributes to

household income?

Sex

| **I would now like to ask you some questions about your home.** | | | | | | |  |
| --- | --- | --- | --- | --- | --- | --- | --- |
| I4. | | What type of dwelling is it? *Please choose the single most appropriate answer.* | | | | |  |
| 1 2 3 4 5 6  6a | | Hostel  Shared/communal flat  Flat, sole use  Part of shared house  House, sole use  Other  Other. Specify .................... .................... | | | | |  |
| 97  98 | | Difficult to answer  Refuse to answer | | | | |  |
|  | |  | | | | |  |
| I5. | | How many metres squared are there? ____________Metres squared | | | | |  |
| 97  98 | | Difficult to answer  Refuse to answer | | | | |  |
| I6. | | **How many rooms do you have in the house/apartment?** ________Number of rooms | | | | |  |
| 97  98 | | Difficult to answer  Refuse to answer | | | | |  |
|  | |  | | | | |  |
| I7. | | Which of the following amenities does your household have access to? | | | | |  |
|  | |  |  |  |  |  |  |
|  | |  | Yes | No | Difficult to answer | Refuse to answer |  |
| 1 | | Car | 1 | 2 | 97 | 98 |  |
| 2 | | Hot water supplied | 1 | 2 | 97 | 98 |  |
| 3 | | Central heating | 1 | 2 | 97 | 98 |  |
| 4 | | Internet connection | 1 | 2 | 97 | 98 |  |
| 5 | | Dish washer | 1 | 2 | 97 | 98 |  |
| 6 | | Modern washing mashine | 1 | 2 | 97 | 98 |  |
| I8. | Do you have dacha? | | | | | | |
| 1  2  97  98 | Yes  No  Difficult to answer  Refuse to answer | | | | | | |
|  |  | | | | | | |
| **I9**. | **Which of the phrases below best describes this household’s financial situation during the past year?** *SHOW CARD 8 Please choose most appropriate answer.* | | | | | | |
| 1 | There is not even enough money for food, it’s difficult to make ends meet | | | | | | |
| 2 | We have enough money for food, but we find it difficult to afford clothes and other items | | | | | | |
| 3 | We have enough money for food and clothes, but would find it difficult to buy large domestic appliances | | | | | | |
| 4 | We can afford to buy large domestic appliances, but would find it difficult to buy a large new car | | | | | | |
| 5 | We can afford to buy a large new car, but would find it difficult to buy a flat or a house (or other property) | | | | | | |
| 6 | We have no financial constraints. We can afford to buy a flat or a house (or other property). | | | | | | |
| 97  98 | Difficult to answer  Refuse to answer | | | | | | |

| **Module K** | **PSYCHOSOCIAL FACTORS** |
| --- | --- |

| **K1. Have any of the following life events or problems happened to you during the last 6 months?** | No | Yes | Difficult to answer | Refuse to answer |
| --- | --- | --- | --- | --- |
| You yourself suffered serious illness, injury or an assault | 2 | 1 | 97 | 98 |
| A serious illness, injury or assault happened to a close relative | 2 | 1 | 97 | 98 |
| Your parent, child or partner died | 2 | 1 | 97 | 98 |
| A close family friend or another relative (aunt, cousin, grandparent) died | 2 | 1 | 97 | 98 |
| You had a divorce/separation due to marital difficulties | 2 | 1 | 97 | 98 |
| You broke off a steady relationship | 2 | 1 | 97 | 98 |
| You had a serious problem with a close friend, neighbour or relative | 2 | 1 | 97 | 98 |
| You became unemployed or you were seeking work unsuccessfully for more than one month | 2 | 1 | 97 | 98 |
| You were sacked from your job | 2 | 1 | 97 | 98 |
| You had a major financial crisis | 2 | 1 | 97 | 98 |
| You had problems with the police and a court appearance | 2 | 1 | 97 | 98 |
| Something you valued was lost or stolen | 2 | 1 | 97 | 98 |

**We have reached the final stage of our questionnaire. To begin with I would like to ask you some questions about events of the past 6 months in your and your relatives lives.**

| K2. | Did someone physically assault you in the past year? |
| --- | --- |
| 1 | Yes |
| 2 | No |
| 97  98 | Difficult to answer  Refuse to answer |
|  |  |
| K3. | What are your relations with your family? |
| 1  2  3 | Harmonious, peaceful  Occasional quarrels and conflicts  Frequent quarrels and conflicts |
| 97  98 | Difficult to answer  Refuse to answer |
|  |  |
| **K4.** | **To what extent do you agree or disagree with each of the following statements?** *SHOW CARD 9. Use a scale from absolutely agree to absolutely disagree* |
| 1  2  3 | It’s husband’s responsibility to earn money and a wife’s responsibility to take care of the house and children  Men and women should paly equally important parts in politics  It’s more suitable for a man than for a woman to be a leader or manager  1 Absolutely agree  2 Agree  3 Yes and no  4 Disagree  5 Absolutely disagree |
| 97  98 | Difficult to answer  Refuse to answer |
|  |  |
| **K5.**  1  2  3 | **In our time various opinions are given on who should be the head of the family. Which statement is closest to your opininon on this subject?**  The husband should be responsible for the family, the head of the family, and the wife should be obedient to her husband  The husband and the wife shoyuld have equal right and be equally responsible for the family  The wife should be responsible for the family, the head of the family |
| 97  98 | Difficult to answer  Refuse to answer |
|  |  |
| **K6.** | **Do you have people that you can confide in, to talk about personal matters when you need it?** |
| 1 | Yes |
| 2 | No |
| 97  98 | Difficult to answer  Refuse to answer |
|  |  |
| **K7.** | **Do you have people who can help you physically or materially when you need it, e.g. pick up a child from school or take you to the hospital?** |
| 1 | Yes |
| 2 | No |
| 97  98 | Difficult to answer  Refuse to answer |
| **K8.** | **Generally speaking, would you say that most people can be trusted, or that you can’t be too careful in dealing with people?**  *Please tell me on a score of 0 to 10, where 0 means you can’t be too careful and 10 means that most people can be trusted*  *0................................................SHOW CARD 10…….……............................10* |
| 97  98 | Difficult to answer  Refuse to answer |
| **K9.** | **Do you think that most people would try to take advantage of you if they got the chance, or would they try to be fair?** *Please tell me on a score of 0 to 10.*  *0................................................SHOW CARD 11…….……............................10* |
| 97  98 | Difficult to answer  Refuse to answer |
| **K10.** | **Would you say that most of the time people try to be helpful or that they are mostly looking out for themselves?** *Please tell me on a score of 0 to 10.*  *0................................................SHOW CARD 12…….……............................10* |
| 97  98 | Difficult to answer  Refuse to answer |
| **K11.** | **How much you agree or disagree with each of the statements on a score 0 to 7.** *SHOW CARD* *13.* **Please tell me on a score of 0 to 7.**  0...............1...............2..............3............4...............5...............6..............7  *Strongly agree Strongly disagree* |
|  | I can find solutions when there are new problems with my health  0...............1...............2..............3............4...............5...............6..............7  *Strongly agree Strongly disagree*  I know how to prevent the deterioration of my health  0...............1...............2..............3............4...............5...............6..............7  *Strongly agree Strongly disagree*  By living healthy I can prevent serious diseases  0...............1...............2..............3............4...............5...............6..............7  *Strongly agree Strongly disagree*  After all, I am responsible for taking care of my own health  0...............1...............2..............3............4...............5...............6..............7  *Strongly agree Strongly disagree* |
| 97  98 | Difficult to answer  Refuse to answer |

| **Module X** | **INTERVIEW CONTEXT** |
| --- | --- |

| *The following questions are answered only by you as an interviewer and are not to be read out.* | |
| --- | --- |
|  |  |
| X1. | How would you judge the reliability of the answers from this interview? |
| 1  2    3 | Satisfactory  Not entirely satisfactory. For example, a moderate level of non-response by *the subject*, or perhaps small interruptions affected the quality of the responses  Poor. For example, a high level of non-response by *the subject*, or perhaps many/constant interruptions affected the quality of the responses. |
|  |  |
| X2. | Were there any other people present in the same room while the interview was taking place? |
| 1 2 | Yes  No |
|  |  |
| X3. | Were there any interruptions to the interview? |
| 1 2 | Yes  No *Go to X5* |
|  |  |
| X4. | Please provide details of interruptions, including their duration_____ |
| X5. | Any other comments, including indication of questions that were particularly hard to answer_______ |
|  |  |
| X6. | Place of interview |
| 1 | At the respondent home |
| 2 | At the respondent work |
| 3 | Near the place of medical examination |
| 4  4a | Other  Other. Specify .................... .................... |

| **Module Y** | **HEALTH CHECK APPOINTMENT** |
| --- | --- |

| **Y1.** | **Was the participant willing to come to a health check?** |
| --- | --- |
| 1 | No, refused |
| 2 | Potentially interested but no health check booked at this time |
| 3 | Yes, participant was interested and health check appointment was booked |
|  |  |
| **Y2.** | **Was written consent to collect participant details obtained?** |
| 1 | Yes |
| 2 | No *Go to Y2a* |
|  |  |
| **Y2a** | **If no written consent was obtained please give the reason**­­­_______ |
|  |  |
| **Y3.** | **Did you see the video clip about this research?** |
| 1 | Yes |
| 2 | No |
| 97  98 | Difficult to answer  Refuse to answer |
| **Y4.** | **Did you see any other information (for example, a billboard on the street) about this research?** |
| 1 | Yes |
| 2 | No |
| 97  98 | Difficult to answer  Refuse to answer |
