# Supplementary material for: Know Your Heart: Rationale, design and conduct of a cross-sectional study of cardiovascular structure, function and risk factors in 4500 men and women aged 35-69 years from two Russian cities, 2015-18
Source: Wellcome Open Res. 2018 Dec 3;3:67. Originally published 2018 Jun 4. [Version 3] doi: 10.12688/wellcomeopenres.14619.3 (PMC6073094; doi:10.12688/wellcomeopenres.14619.3)
Supplement: Supplementary file 3 [file wellcomeopenres-3-16294-s0002.tgz › d9a03f66-5fce-45a3-8a2e-d18febd22997_Supplementary_file_3._Questionnaires_and_data_collection_tools.zip!/questionnaires_181121_publish_version2/181118_Baseline_survey_main_RU.docx]

|  |  |
| --- | --- |

**Опросник первого этапа**

Международный проект по сердечно-сосудистым заболеваниям в России

2015-2018

Версия публикации 2.0 Ноябрь 2018

В России один из самых высоких уровней сердечно-сосудистых заболеваемости в мире. Целью международного проекта "Сердечно-сосудистые заболевания в России" является понимание причин данного положения. Весомым компонентом данного исследования было исследование От Сердца к Сердцу, направленное на выявление природы и причин сердечно-сосудистой заболеваемости в России. В рамках данного проекта были проведены масштабные перекрёстные опросы в двух городах России, Новосибирске и Архангельске. Ислледование охватило 4542 мужчин и женщин в возрате 35-69 лет, набранных из генеральной популяции в период 2015-2018. Исследование включало два компонента: 1)опрос населения для получения информации по социально-демографическим характеристикам и сердечно-сосудистым факторам риска; в большинстве случаев опрос проводился на дому. 2)комплексное медицинское обследование в клинике, включающее детальное изучение сердечно-сосудистой системы.

Международный проект "Сердечно-сосудистые заболевания в России" был частично финансирован Wellcome Trust Strategic Award [100217]. Проект также был финансирован Арктическим Университетом Норвегии, Университет Тромсё, Норвежским Институтом Общественного Здоровья и Министерством Здравоохранения и Социального Обеспечения Норвегии.

Разработка опросников велась под руководством Лондонской Школы Гигиены и Тропической Медицины совместно с Северным государственным медицинским университетом, г.Архангельск, Новосибирским государственным медицинским университетом, Научно-исследовательским институтом терапии и профилактической медицины-филиала «ФИЦ ИЦиГ» СО РАН, г. Новосибирск и коллегами других институтов.

Данная бумажная версия опросника была использована для проведения интервью первого этапа. Интервью проводилось электронно с использованием Computer Assisted Personal Interviewing CAPI устройства.

Подробные детали исследования опубликованы в Know Your Heart: Rationale, design and conduct of a cross-sectional study of cardiovascular structure, function and risk factors in 4500 men and women aged 35-69 years from two Russian cities, 2015-18
Cook S, Malyutina S, Kudryavtsev AV, Averina M, Bobrova N, Boytsov S, Brage S, Clark T et al.

Содержание

[Модуль A. Социально-демографические факторы	4](#_Toc512505986)

[Модуль B. Физическая активность	8](#_Toc512505991)

[Модуль C. Самооценка здоровья	10](#_Toc512505992)

[Модуль D. Обращение за медицинской помощью.	13](#_Toc512505994)

[Модуль E. Депрессия и Тревога.	19](#_Toc512505995)

[Модуль F. Питание.	21](#_Toc512505996)

[Модуль G. Употребление алкоголя.	23](#_Toc512505996)

[Модуль H. Курение	32](#_Toc512505997)

[Модуль I. Домохозяйство	34](#_Toc512505999)

[Модуль K. Психосоциальные факторы	37](#_Toc512506001)

[Модуль X. Обстоятельства интервью	40](#_Toc512506003)

[Модуль Y. Приглашение на медицинское обследование	41](#_Toc512506009)

| Дата Интервью__ __ / __ __ / __ __ __ __ ID Интервьюера___ ID Респондента________ | |
| --- | --- |
| **Модуль A** | **СОЦИАЛЬНО-ДЕМОГРАФИЧЕСКИЕ ФАКТОРЫ** |

**Для начала позвольте задать несколько вопросов о Вас.**

| A1. | Сколько Вам лет?_________Лет |
| --- | --- |
| 97  98 | Затрудняюсь ответить  Отказ от ответа |
| A2. | Назовите дату Вашего рождения? __ __ / __ __ / __ __ __ __ |
| 97  98 | затрудняюсь ответить  отказ от ответа |
| **A3.** | Интервьюер! Отметьте пол респондента. |
| 1  2 | Мужской  Женский |
|  |  |
| A4. | Кто Вы по национальности? *Архангельск/Новосибирск.* *Возможен только один вариант ответа.* |
| 1 2  3  4  5  5a | Русский  Украинец/Татарин  Ненец/Украинец  Белорус/Узбек  Другое  Другое. Уточните .................... .................... |
| 97  98 | Затрудняюсь ответить  Отказ от ответа |
|  |  |
| A5. | Где Вы родились? *Возможен только один вариант ответа.* |
| 1 2 3 4 5 | В Архангельске/Новосибирске  В Архангельской/Новосибирской области  В другом регионе России  В регионе бывшего Советского Союза за пределами России  За пределами бывшего Советского Союза |
| 97  98 | Затрудняюсь ответить  Отказ от ответа |
|  |  |
| A6. | Как давно Вы постоянно проживаете в Архангельске/Новосибирске? *Возможен только один вариант ответа.* |
| 1  2  3  4  5 | Не более 12 месяцев  От 1 года до 5 лет  От 5 лет до 10 лет  Более 10 лет, но не всю жизнь  С рождения (не считая периоды службы в армии, другие периоды временного отсутствия) |
| 97  98 | Затрудняюсь ответить  Отказ от ответа |
|  |  |
|  |  |
| A7. | Каково Ваше семейное положение в настоящее время? Вы *Возможен только один вариант ответа*. |
| 1 2 3 4 5 | Проживаете в зарегистрированном браке  Проживаете в незарегистрированном браке  Разведены или живете раздельно с женой/мужем  В довец/вдова  Не женаты/не замужем и никогда не были |
| 97  98 | Затрудняюсь ответить  Отказ от ответа |
|  |  |
| A8. | Сколько у Вас детей? *Возможен только один вариант ответа.* |
| 1  2  3  4  5 | 0  1  2  3  4 или больше |
| 97  98 | Затрудняюсь ответить  Отказ от ответа  I wo |

Сейчас я хочу спросить о Вашем образовании и работе.

| A9. | **Каков уровень Вашего образования?** *Покажите карточку 1. Возможен только один вариант ответа.* | | |
| --- | --- | --- | --- |
| 1 2 3 4 5  6  7 | Неполное среднее или ниже  Полное среднее  Начальное профессиональное (ПТУ, училище без среднего образования)  Профессиональное среднее (н-р, ПТУ с полным средним образованием)  Среднее специальное (н-р, медицинское, педагогическое училище, техникум)  Незаконченное высшее  Высшее | | |
| 97  98 | Затрудняюсь ответить  Отказ от ответа | | |
| **A10.** | **Учитесь ли Вы в данный момент (являетесь ли студентом)?** | | |
| 1 | Да | | |
| 2 | Нет | | |
| 97 | Затрудняюсь ответить | | |
| 98 | Отказ от ответа | | |
| **A11.** | **Являетесь ли Вы…** Возможен только один вариант ответа. | | |
| 1  2  3  97  98 | Пенсионером (кроме пенсионеров по инвалидности)  Пенсионером по инвалидности  Ничего из перечисленного  Затрудняюсь ответить  Отказ от ответа | | |
|  |  | | |
| **A12.**  1  2  97  98 | **Являетесь ли Вы работающим на постоянно оплачиваемой работе?**  Да  Нет *Переход к A14*  Затрудняюсь ответить  Отказ от ответа | | |
|  |  | | |
| **A13.**  **A14.**  1  2  3  4  5  5a  6 | **В течение последних 12 месяцев сколько месяцев Вы работали на данной работе?**  Количество месяцев:……………………….. *Переход к A17*  **Являетесь ли Вы…** *Вопрос не задаётся тем, кто на постоянной оплачиваемой работе. (A12-* *Нет)*  Работающим временно или от случая к случаю  Безработным, ищущим работу  Неработающим, не ищущим работу  Домохозяйкой  Другое  Другое. Уточните .................... ....................  Ничего из перечисленного | | |
| 97  98 | Затрудняюсь ответить  Отказ от ответа | | |
| **A15.** | **Как давно Вы оставили постоянную работу?** *Возможен только один вариант ответа.* | | |
| 1 2 3 4 5 6 | Никогда не было постоянной работы *Переход к A17*  В течение последней недели  От 1 до 4 недель назад  От 1 до 6 месяцев назад  От 6 до 12 месяцев назад  Более года назад | | |
| 97  98 | Затрудняюсь ответить  Отказ от ответа | | |
| **A16.** | **По какой причине Вы оставили постоянную оплачиваемую работу?** *Возможен только один вариант ответа.* | | |
| 1 2 3 4 5  6  7  8  9  10  11  11a | Начали учиться  Вышли на пенсию  Не могли найти работу после окончания учебы  Предприятие (организация) закрылось или произошло сокращение  Закончился срок временного трудоустройства  Предложили уйти/Вас уволили  Оставили работу добровольно из-за неудовлетворенности оплатой или условиями труда  всегда работал (-а) на временных работах  Оставили работу по состоянию здоровья  Оставили работу, чтобы сидеть с детьми, ухаживать за другими людьми  Оставили работу по другой причине  Другое  Другое. Уточните .................... .................... | | |
| 97  98 | Затрудняюсь ответить  Отказ от ответа | | |
| Я хочу задать несколько вопросов о Вашей основной работе в последний год или в тот год, когда Вы работали. | | | |
| **A17.** | Кем Вы работали в течение этого последнего года, какая была ваша основная оплачиваемая работа? *Интервьюер впечатывает полное название (н-р, учитель начальных классов)*  Ваша оплачиваемая работа:................................................................. | | |
| 97  98 | Затрудняюсь ответить  Отказ от ответа | | |
| **A18.**  1  2  3  4  5  6  7  8  9 | **Что из перечисленного лучше всего описывает Вашу работу?** *Покажите карточку 2. Возможен только один вариант ответа.*  Законодатель, крупный чиновник, руководитель высшего звена  Специалист высшего уровня квалификации  Специалист среднего уровня квалификации  Служащий офисный и по обслуживанию клиентов  Работник сферы торговли и услуг  Квалифицированный рабочий, занятый ручным трудом  Квалифицированный рабочий, использующий машины и механизмы Неквалифицированный рабочий  Военнослужащий | | |
| 10  10a | Другое  Другое. Уточните .................... .................... | | |
| 97  98 | Затрудняюсь ответить  Отказ от ответа | | |
| **A19.** | **Пропускали ли Вы работу по состоянию своего здоровья в последние 12 месяцев?** *(Вопрос только для тех, кто выполнял оплачиваемую работу в последние 12 месяцев)* | | |
| 1  2 | Да  Нет *Переход к A21* | | |
| 97  98 | Затрудняюсь ответить  Отказ от ответа *Переход к A21* | | |
| **A20** | **Сколько дней вы пропустили по состоянию здоровья?** | | |
| 1 | 1-3 дня | |  |
| 2 | 4-5 дней | |  |
| 3 | 6-10 дней | |  |
| 4 | 11+ дней | |  |
| 97 | Затрудняюсь ответить | |  |
| 98 | Отказ от ответа | |  |
| A21 | Есть ли у Вас в данный момент официально установленная инвалидность? | | |
| 1  2 | Да  Нет *Переход к B1* | | |
| 97  98 | Затрудняюсь ответить *Переход к B1*  Отказ от ответа *Переход к B1* | | |
| A22. | Когда она была установлена? | | |
| 1  2  3  4  5  6  97  98 | Не более 6 месяцев назад  От 6 до 12 месяцев назад  От 1 до 5 лет назад  От 5 до 10 лет назад  Более 10 лет назад, но не с рождения  С самого рождения  Затрудняюсь ответить  Отказ от ответа | | |
| A23. | Какая у Вас в данный момент группа инвалидности? | | |
| 1 2 3 | 1-я  2-я  3-я | | |
| 97  98 | Затрудняюсь ответить  Отказ от ответа | | |
| **Модуль B** | | **ФИЗИЧЕСКАЯ АКТИВНОСТЬ** | |
| **B1.** | **Следующие вопросы будут касаться Вашей физической активности.**  **Мы хотим узнать xарактер и объем физической активности на Вашей работе.**  **Пожалуйста, выбирите наиболее подходящий вариант Вашей активности из нижеследующих четырех возможных вариантов:** *Покажите карточку 3. Возможен только один вариант ответа.* | | |
| 1  2  3  4 | **Сидячая работа**: Вы проводите большую часть рабочего времени сидя (например, в офисе)  **Стоячая работа**: Вы проводите большую часть рабочего времени стоя или ходите. Но тем не менее, Ваша работа не требует большого физического напряжения (например, продавец, парикмахер, охранник)  **Физическая работа**: Это включает применение физической силы, например, перенос тяжестей, работа с инструментами (водопроводчик, дворник, спортивный тренер, медсестра, электрик, плотник).  **Ручной труд**: Это работа, требующая больших физических усилий, включая перенос очень тяжелых предметов (докер, шахтер, каменщик, строитель) | | |
| 97  98 | Затрудняюсь ответить  Отказ от ответа | | |
| **B2.** | **Если говорить о последних 12 месяцах, сколько часов Вы в течениe обычной недели занимались следующими видами физической активности?** | | |
| 1  2  3  4  5  6 | Ходьбой, включая ходьбу на работу, по магазинам, пешие прогулки?  Количество часов летом................. Количество часов зимой................  Катались на велосипеде, включая езду на велосипеде на работу, прогулки на велосипеде  Количество часов летом................. Количество часов зимой................  Работали в саду, огороде  Количество часов летом................. Количество часов зимой................  Выполняли домашнюю работу: убирали, стирали, готовили, ухаживали за ребенком  Количество часов в неделю……………..  Занимались ремонтом, починкой, мастерили, плотничали  Количество часов в неделю……………..  Занимались спортом, физическими упражнениями, фитнессом: аэробикой, плаванием, бегом, катанием на коньках, лыжах?  Количество часов летом................. Количество часов зимой............................. | | |
| 97  98 | Затрудняюсь ответить  Отказ от ответа | | |
|  |  | | |
| **B3.** | **Случалось ли в течение последних 12 месяцев, что Вы занимались перечисленными выше видами физической активности настолько интенсивно, что Вы вспотели или у Вас участилось сердцебиение?** | | |
| 1 | Да  Если да, то сколько всего часов в неделю Вы занимались такой интенсивной физической активностью, которая вызывала у Вас учащение сердцебиения?  Количество часов в неделю…………….. | | |
| 2 | Нет | | |
| 97  98 | Затрудняюсь ответить  Отказ от ответа | | |
| **B4.** | **Если говорить о последних 12 месяцах, на сколько этажей по лестнице Вы обычно поднимаетесь за день?** | | |
|  | Количество этажей за день............................................ | | |
|  |  | | |
| 97  98 | Затрудняюсь ответить  Отказ от ответа | | |

| **Модуль C** | **САМООЦЕНКА ЗДОРОВЬЯ** |
| --- | --- |

Эта часть анкеты содержит вопросы, касающиеся ваших взглядов на своё здоровье. Предоставленная информация поможет понять, как вы себя чувствуете и насколько хорошо справляетесь со своими обычными нагрузками.

| **C1.** | **В целом вы оценили бы состояние вашего здоровья как:** *Возможен только один вариант ответа.* | |
| --- | --- | --- |
|  |  | |
| 1 2  3  4  5 | Отличное  Очень хорошее  Хорошее  Посредственно  Плохое | |
|  |  | |
|  | | **Ограничивает ли вас состояние здоровья в настоящее время в выполнении перечисленных ниже действий? Если да, то в какой степени?** |
|  | |  |
| **C2.** | | **Умеренные физические нагрузки, такие как перемещение стола, работа с пылесосом, сбор грибов или работа на огороде.** *Возможен только один вариант ответа.* |
|  | |  |
| 1  2  3 | | Да, значительно ограничивает  Да, немного ограничивает  Нет, совсем не ограничивает |
| C3. | | **Подъем по лестнице на НЕСКОЛЬКО пролетов** *Возможен только один вариант ответа.* |
|  | |  |
| 1  2  3 | | Да, значительно ограничивает  Да, немного ограничивает  Нет, совсем не ограничивает |
|  | |  |
| **В течение последних 4-х недель испытывали ли вы затруднения в выполнении работы или обычных привычных занятий, СВЯЗАННЫХ С ФИЗИЧЕСКИМ СОСТОЯНИЕМ здоровья?** *Покажите карточку 4.* | | |
| **C4.** | | **ВЫПОЛНИЛИ МЕНЬШЕ, чем хотели:** |
| 1  2  3  4  5 | | Все время  Большую часть времени  Иногда  Редко  Ни разу |
| **C5.** | | **Вы были ограничены в выполнении какого-либо ОПРЕДЕЛЕННОГО ВИДА работы или другой деятельности** *Покажите карточку 4.* |
| 1  2  3  4  5 | | Все время  Большую часть времени  Иногда  Редко  Ни разу |
| **В течение последних 4-х недель испытывали ли вы затруднения в выполнении работы или обычных привычных занятий ИЗ-ЗА ЭМОЦИОНАЛЬНОГО СОСТОЯНИЯ (например, подавленности или беспокойства)?** *Покажите карточку 4.* | | |
|  | | |
| **C6.** | | **ВЫПОЛНИЛИ МЕНЬШЕ, чем хотели:** |
| 1  2  3  4  5 | | Все время  Большую часть времени  Иногда  Редко  Ни разу |
| **C7.** | | **Выполняли свою работу или другие дела МЕНЕЕ АККУРАТНО, чем обычно:** *Покажите карточку 4.* |
| 1  2  3  4  5 | | Все время  Большую часть времени  Иногда  Редко  Ни разу |
| **C8.** | | **В какой степени боль в течение последних 4 недель мешала вам заниматься вашей обычной работой (включая работу вне дома и по дому)** |
| 1  2  3  4  5 | | Совсем не мешала  Немного  Умеренно  Сильно  Очень сильно |
| **Следующие вопросы касаются того, как Вы себя чувствовали, и каким было Ваше настроение в течение последних 4 недель. Пожалуйста, на каждый вопрос дайте ОДИН ответ, который наиболее соответствует вашим ощущениям.**  **Как часто в течение последних 4 недель…**.... | | |
| **C9.** | | **Вы чувствовали себя спокойным и умиротворенным?** *Покажите карточку 4.* |
| 1  2  3  4  5 | | Все время  Большую часть времени  Иногда  Редко  Ни разу |
|  | |  |
| **C10.** | | **Вы чувствовали себя полным сил и энергии?** *Покажите карточку 4.* |
| 1  2  3  4  5 | | Все время  Большую часть времени  Иногда  Редко  Ни разу |
|  | |  |
| **C11.** | | **Вы ощущали упадок духа, или депрессию?** *Покажите карточку 4* |
| 1  2  3  4  5 | | Все время  Большую часть времени  Иногда  Редко  Ни разу |
|  | |  |
| **C12.** | | **Как часто за последние 4 недели ваше физическое или эмоциональное состояние мешало вам активно общаться с людьми (навещать друзей, родственников и т. п.)?** |
| 1  2  3  4  5 | | Все время  Большую часть времени  Иногда  Редко  Ни разу |
|  | |  |

|  | **Модуль D** | | | | | **ОБРАЩЕНИЕ ЗА МЕДИЦИНСКОЙ ПОМОЩЬЮ** | | |
| --- | --- | --- | --- | --- | --- | --- | --- | --- |
| D1. | | **Данный раздел будет касаться обращения за медицинской помощью, а также наличию некоторых заболеваний и применению медицинских препаратов.** **Сколько раз за последние 12 месяцев Вы обращались за медицинской помощью к нижеперечисленным специалистам? Пожалуйста отметьте количество визитов к каждому специалисту.** Вид помощи \| Количество посещений за последние 12 месяцев \| \| \| \| \|; 1. Участковый врач/терапевт \| 0 \| 1 \| 2 \| 3 \| 4 \| 5+; 2. Кардиолог в поликлиннике \| 0 \| 1 \| 2 \| 3 \| 4 \| 5+; 3. Другой специалист в поликлиннике \| 0 \| 1 \| 2 \| 3 \| 4 \| 5+; 4. Кардиолог в больнице/ госпитале/ стационаре \| 0 \| 1 \| 2 \| 3 \| 4 \| 5+; 5. Другой специалист в больнице/госпитале/ стационаре \| 0 \| 1 \| 2 \| 3 \| 4 \| 5+ | | | | | | |
|  | | |  | | | | | |
| **D2.** | | | **Сколько раз Вы были госпитализированы за последние 12 месяцев? (провели в госпитале/больнице ночь/сутки)?**  Количество раз.................................... If “0” *Переход к D4* | | | | | |
| 97  98 | | | Затрудняюсь ответить  Отказ от ответа | | | | | |
| **D3.** | | | **Сколько дней (суток) Вы провели в больнице/госпитале в каждую из этих госпитализаций?** | | | | | |
|  | | | Госпитализация | Количество дней/суток | | | | |
| 1  2  3  4  5 | | |  | .......................................  .......................................  .......................................  .......................................  ....................................... | | | | |
| 6 | | |  | ....................................... | | | | |
| 7 | | |  | ....................................... | | | | |
| 8 | | |  | ....................................... | | | | |
| 9 | | |  | ....................................... | | | | |
| 10 | | |  | ....................................... | | | | |
| 97  98 | | | Затрудняюсь ответить  Отказ от ответа |  | | | | |
|  | | |  | | | | | |
| **D4.** | | | **Сколько раз Вы или кто-либо для Вас вызывали скорую помощь на протяжении последних 12 месяцев?** Количество раз.................. | | | | | |
| 97  98 | | | Затрудняюсь ответить  Отказ от ответа | | | | | |
|  | | |  | | | | | |
| **D5.** | | | **При посещении врача/ей (участкового, кардиолога, другого специалиста) в последние 12 месяцев советовали ли Вам изменить свой образ жизни для улучшения своего здоровья, в том числе:** | | | | | |
|  | | |  | Да | Нет | | Затрудняюсь ответить | Отказ от ответа |
| **D5a.** | | | **Изменить питание** | 1 | 2 | | 97 | 98 |
|  | | |  |  |  | |  |  |
| **D5b.** | | | **Заняться физическими упражнениями/спортом** | 1 | 2 | | 97 | 98 |
|  | | |  |  |  | |  |  |
| **D5c.** | | | **Снизить вес** | 1 | 2 | | 97 | 98 |
|  | | |  |  |  | |  |  |
| **D5d.** | | | **Бросить курить** | 1 | 2 | | 97 | 98 |
|  | | |  | | | | | |
| **D6.** | | | **Насколько Вы осведомлены об артериальной гипертензии, или повышенном артериальном давлении?** | | | | | |
| 1  2  3  4 | | | Вообще не осведомлен  я только слышал/а этот термин  Я немного осведомлен/а.  Я очень хорошо осведомлен/а | | | | | |
| 97  98 | | | Затрудняюсь ответить  Отказ от ответа | | | | | |
|  | | |  | | | | | |
| **D7.** | | | **Измеряли ли Вы артериальное давление у врача или другого медицинского работника за последние 2 года?** | | | | | |
| 1  2 | | | Да *Переход к D9*  Нет *Переход к D8* | | | | | |
| 97  98 | | | Затрудняюсь ответить *Переход к D9*  Отказ от ответа *Переход к D9* | | | | | |
|  | | |  | | | | | |
| **D8.** | | | **Почему Вы не измеряли артериальное давление у врача?** *Возможно несколько вариантов ответа* | | | | | |
| 1 | | | Я не думаю, что это положительно повлияет на моё здоровье | | | | | |
| 2 | | | Я чувствую себя хорошо, не нуждаюсь в измерении давления | | | | | |
| 3 | | | Клиника (поликлиника) находится далеко | | | | | |
| 4 | | | Время ожидания в клиниках (поликлиниках) достаточно длительное | | | | | |
| 5 | | | Поликлиники открыты в неудобное время (время работы поликлиник неудобно) | | | | | |
| 6 | | | Я не знаю, где можно измерить своё артериальное давление | | | | | |
| 7 | | | Я измеряю своё артериальное давление дома | | | | | |
| 8  8a | | | Другое  Другое. Уточните .................... .................... | | | | | |
| 97  98 | | | Затрудняюсь ответить  Отказ от ответа | | | | | |

| **D9.** | **Какое из нижеследующих утверждений верно?** *Возможен только один вариант ответа.* | | | | |
| --- | --- | --- | --- | --- | --- |
| 1  2  3 | Большинство людей с повышенным артериальным давлением ощущают его симптомы  Большинство людей с повышенным артериальным давлением иногда ощущают его симптомы  Большинство людей с повышенным артериальным давлением никогда не ощущают его симптомы | | | | |
| 97  98 | Затрудняюсь ответить  Отказ от ответа | | | | |
|  |  | | | | |
| **D10.** | **Говорил ли Вам когда-либо врач, что у Вас имеются/имелись, или были диагностированы следующие заболевания**: | | | | |
|  |  | Да | Нет | Затрудняюсь ответить | Отказ от ответа |
| 1 | Артериальная гипертензия (повышенное давление). *Для женщин: за исключением периода беременности.* | 1 | 2 | 97 | 98 |
| 2 | Артериальная гипертензия во время беременности (*Для женщин)* | 1 | 2 | 97 | 98 |
| 3 | Повышенный уровень холестерина | 1 | 2 | 97 | 98 |
| 4 | Инфаркт миокарда/Cердечный приступ | 1 | 2 | 97 | 98 |
| 5 | Сердечная недостаточность | 1 | 2 | 97 | 98 |
| 6 | Фибрилляция предсердий | 1 | 2 | 97 | 98 |
| 7 | Стенокардия/Ишемическая болезнь сердца | 1 | 2 | 97 | 98 |
| 8 | Инсульт | 1 | 2 | 97 | 98 |
| 9 | Сахарный диабет | 1 | 2 | 97 | 98 |
| 10 | Заболевание почек | 1 | 2 | 97 | 98 |
| 11 | Хронический бронхит | 1 | 2 | 97 | 98 |
| 12 | Онкологические заболевания/Рак | 1 | 2 | 97 | 98 |
| 13 | Бронхиальная астма | 1 | 2 | 97 | 98 |
| 14 | Ревматический артрит | 1 | 2 | 97 | 98 |
| 15 | Остеоартрит (остеоартроз) | 1 | 2 | 97 | 98 |
| 16 | Мигрень | 1 | 2 | 97 | 98 |
| 17 | Депрессия | 1 | 2 | 97 | 98 |
| 18 | Повышенная тревожность | 1 | 2 | 97 | 98 |
|  | *Примечание: D11-D15 вопросы задаются тем респондентам, кто ответил, что имеет повышенное давление. D16-D21 вопросы задаются тем респондентам, кто ответил, что имеет повышенный холестерин. Остальные респонденты переходят к вопросу D22*. | | | | |
|  |  | | | | |
| **D11.** | **Назначал ли Вам врач (или другой медицинский работник) медикаментозные препараты для лечения артериальной гипертензии?** | | | | |
| 1  2 | Да  Нет *Переход к D21 (повышенный уровень холестерина- переход к D16)* | | | | |
| 97  98 | Затрудняюсь ответить *Переход к D21* *(повышенный уровень холестерина- переход к D16)*  Отказ от ответа *Переход к D21(повышенный уровень холестерина- переход к D16)* | | | | |
|  |  | | | | |
|  |  | | | | |
| **D12.** | **Приобрели ли Вы все медицинские препараты, назначенные врачом?** | | | | |
| 1  2 | Да *Переход к D14*  Нет | | | | |
| 97  98 | Затрудняюсь ответить  Отказ от ответа *Переход к D14* | | | | |
|  |  | | | | |
| **D13.** | **Каковы были причины того, что Вы не приобрели все назначенные препараты?** *Возможно несколько вариантов ответа.* | | | | |
| 1  2  3  4  5  6  7  8  8a | Назначенные препараты отсутствовали в аптеке  Назначенные препараты дорого стоили  Назначенные препараты неэффективны  Назначенные препараты имеют побочные эффекты  Я предпочитаю принимать для снижения артериального давления другие медикаментозные препараты, рекомендованными родственниками/друзьями  Я предпочитаю принимать для лечения артериальной гипертензии другие медикаментозные препараты, рекомендованные фармацевтом  Я предпочитаю принимать для лечения артериальной гипертонии другие альтернативные, немедицинские методы  Другое  Другое. Уточните .................... .................... | | | | |
| 97  98 | Затрудняюсь ответить  Отказ от ответа | | | | |
|  |  | | | | |
| **D14.** | **Принимаете ли Вы рекомендованные медикаментозные препараты каждый день?** | | | | |
| 1  2  3 | Да *Переход к D16*  Нет  Не приобрел/а никаких медицинских препаратов | | | | |
| 97  98 | Затрудняюсь ответить  Отказ от ответа | | | | |
| **D15.** | **В чем причина того, что Вы не принимали рекомендованные препараты каждый день?** *Возможно несколько вариантов ответа.* | | | | |
| 1 | У меня больше нет повышенного давления | | | | |
| 2 | Я не знал, что лекарства необходимо принимать каждый день | | | | |
| 3 | Врач сказал мне, что я должен принимать лекарства только тогда, когда есть симптомы | | | | |
| 4  5 | Я не могу (финансово) позволить себе принимать их каждый день  Я забываю принимать их каждый день | | | | |
| 6 | Я прекращаю принимать их, когда начинаю чувствовать себя хуже | | | | |
| 7 | Я прекращаю принимать их, когда начинаю чувствовать себя лучшe | | | | |
| 8 | Употребление препаратов каждый день влечет за собой появление побочных эффектов | | | | |
| 9 | Я предпочитаю принимать безрецептурные препараты | | | | |
| 10  10a | Другое  Другое. Уточните .................... .................... | | | | |
| 97  98 | Затрудняюсь ответить  Отказ от ответа | | | | |
|  |  | | | | |
|  |  | | | | |
|  |  | | | | |
| **D16.** | **Рекомендовал ли Вам врач (или другой медицинский работник) медикаментозные препараты (лекарства) для снижения повышенного уровня холестерина)?** | | | | |
| 1  2 | Да  Нет *Переход к D21* | | | | |
| 97  98 | Затрудняюсь ответить *Переход к D21*  Отказ от ответа *Переход к D21* | | | | |
| **D17.** | **Приобрели ли Вы все медикаментозные препараты (лекарства), выписанные врачом?** | | | | |
| 1  2 | Да *Переход к D19*  Нет | | | | |
| 97  98 | Затрудняюсь ответить  отказ от ответа *Переход к D19* | | | | |
|  |  | | | | |
| **D18.** | **Каковы были причины того, что Вы не приобрели все выписанные препараты?** *Возможно несколько вариантов ответа.* | | | | |
| 1  2  3  4  5  6  7  8  8a | Назначенные препараты отсутствовали в аптеке  Назначенные препараты дорого стоили  Назначенные препараты неэффективны  Назначенные препараты имеют побочные эффекты  Я предпочитаю принимать для снижения холестерина другие медикаментозные препараты, рекомендованныв родственниками/друзьями  Я предпочитаю принимать для снижения холестерина другие медикаментозные препараты, рекомендованные фармацевтом  Я предпочитаю принимать для снижения холестерина другие альтернативные, немедицинские методы  Другое  Другое. Уточните .................... .................... | | | | |
| 97  98 | Затрудняюсь ответить  Отказ от ответа | | | | |
|  |  | | | | |
| **D19.** | **Принимаете ли Вы рекомедованные лекарства каждый день?** | | | | |
| 1  2  3 | Да *Переход к D21*  Нет  Не приобрел/а никаких медицинских препаратов | | | | |
| 97  98 | Затрудняюсь ответить  Отказ от ответа *Переход к D21* | | | | |
| **D20.** | **В чем причина того, что Вы не принимали рекомендованные препараты каждый день?** *Возможно несколько вариантов ответа.* | | | | |
| 1  2  3  4  5  6  7  8  9  10  10a | У меня больше нет повышенного холестерина  Я не знал, что лекарства необходимо принимать каждый день.  Врач сказал мне, что я должен принимать лекарства только тогда, когда есть симптомы.  Я не могу (финансово) позволить себе принимать их каждый день.  Я забываю принимать их каждый день.  Я прекращаю принимать их, когда начинаю чувствовать себя хуже.  Я прекращаю принимать их, когда начинаю чувствовать себя лучшe.  Употребление препаратов каждый день влечет за собой появление побочных эффектов.  Я предпочитаю принимать безрецептурные препараты.  Другое  Другое. Уточните .................... .................... | | | | |
| 97  98 | Затрудняюсь ответить  Отказ от ответа | | | | |
| **D21.** | **Помимо выписанных препаратов приобретали ли Вы другие медикаменты/лекарства не рекомендованные врачом (те, которые порекомендовали фармацевт, друзья/родственники) для снижения повышенного давления и/или холестерина?** | | | | |
| 1  2 | Да  Нет | | | | |
| 97  98 | Затрудняюсь ответить  Отказ от ответа | | | | |
|  |  | | | | |
| **D22.** | **Знаете ли Вы о программе диспансеризации (в поликлиниках), которая проводится в настоящее время в России?** | | | | |
| 1  2 | Да  Нет *Переход к E1* | | | | |
| 97  98 | Затрудняюсь ответить *Переход к E1*  Отказ от ответа *Переход к E1* | | | | |
| **D23.** | **Получали ли Вы приглашение на участие в диспансеризации?** | | | | |
| 1  2 | Да  Нет *Переход к E1* | | | | |
| 97  98 | Затрудняюсь ответить *Переход к E1*  Отказ от ответа *Переход к E1* | | | | |
| **D24.** | **Собираетесь ли Вы пройти или уже прошли эту диспансеризацию?** | | | | |
| 1  2 | Да *Переход к E1*  Нет | | | | |
| 97  98 | Затрудняюсь ответить *Переход к E1*  Отказ от ответа *Переход к E1* | | | | |
| **D25.** | **В чем причина Вашего решения?** *Возможно несколько вариантов ответа.* | | | | |
| 1  2  3  4  5  6  7  7a | Я не думаю, что диспансеризация положительно повлияет на моё здоровье  Я чувствую себя хорошо, не нуждаюсь в обследовании  Клиника (поликлиника), где проходит диспансеризация находится далеко  Длительное время ожидания в клиниках (поликлиниках), где можно пройти диспансеризацию  Поликлиники работают в неудобное время (Время работы поликлинник неудобно)  Я не знаю, где можно пройти диспансеризацию  Другое  Другое. Уточните .................... .................... | | | | |
| 97  98 | Затрудняюсь ответить  Отказ от ответа | | | | |

| **Модуль E** \| **ДЕПРЕССИЯ И ТРЕВОГА** **Сейчас я хотела бы поговорить о том, как Вы себя чувствовали в последние 2 недели.** **Как часто за последние 2 недели Вас беспокоили** **следующие проблемы?** *Покажите карточку 5.* | | Ни разу | Несколько дней | Более  Недели | Почти  каждый  день |  |  |
| --- | --- | --- | --- | --- | --- | --- | --- |
| **E1.** | Вам не хотелось ничего делать | 0 | 1 | 2 | 3 |  |  |
| **E2.** | У Вас было плохое настроение, Вы были подавлены или испытывали чувство безысходности | 0 | 1 | 2 | 3 |  |  |
| **E3.** | Вам было трудно заснуть, у Вас был прерывистый сон, или Вы слишком много спали | 0 | 1 | 2 | 3 |  |  |
| **E4.** | Вы были утомлены, или у Вас было мало сил | 0 | 1 | 2 | 3 |  |  |
| **E5.** | У Вас был плохой аппетит, или Вы переедали | 0 | 1 | 2 | 3 |  |  |
| **E6.** | Вы плохо о себе думали: считали себя неудачником (неудачницей)  или были в себе разочарованы, или считали, что подвели свою семью | 0 | 1 | 2 | 3 |  |  |
| **E7.** | Вам было трудно сосредоточиться (например, на чтении газеты или на просмотре телепередач) | 0 | 1 | 2 | 3 |  |  |
| **E8.** | Вы двигались или говорили настолько медленно, что окружающие это замечали? Или, наоборот, Вы были настолько суетливы или взбудоражены, что двигались гораздо больше обычного | 0 | 1 | 2 | 3 |  |  |
| **E9.** | Вас посещали мысли о том, что Вам лучше было бы умереть, или о том, чтобы причинить себе какой-нибудь вред | 0 | 1 | 2 | 3 |  |  |
| **E10.** | Oцените, насколько трудно Вам было работать, заниматься домашними делами или общаться с людьми из-за этих проблем? | Совсем не трудно | Немного трудно | Очень трудно | Чрезвычайно трудно |  |  |
|  |  | 0 | 1 | 2 | 3 |  |  |
|  |  |  |  |  |  |  |  |

| **Как часто за последние 2 недели Вас беспокоили**  **следующие проблемы?** *Покажите карточку 5* | | Ни разу | Несколько дней | Более  Недели | Почти  каждый  день |  |  |
| --- | --- | --- | --- | --- | --- | --- | --- |
| **E11.** | Вы нервничали, тревожились или испытывали сильный стресс | 0 | 1 | 2 | 3 |  |  |
| **E12.** | Вы были неспособны успокоиться или контролировать свое волнение | 0 | 1 | 2 | 3 |  |  |
| **E13.** | Вы слишком сильно волновались по различным поводам | 0 | 1 | 2 | 3 |  |  |
| **E14.** | Вам было трудно расслабиться | 0 | 1 | 2 | 3 |  |  |
| **E15.** | Вы были настолько суетливы, что Вам было тяжело усидеть на месте | 0 | 1 | 2 | 3 |  |  |
| **E16.** | Вы легко злились или раздражались | 0 | 1 | 2 | 3 |  |  |
| **E17.** | Вы испытывали страх, словно должно произойти нечто ужасное | 0 | 1 | 2 | 3 |  |  |
| **E18.** | Oцените, насколько трудно Вам было работать, заниматься домашними делами или общаться с людьми из-за этих проблем? | Совсем не трудно | Немного трудно | Очень трудно | Чрезвычайно трудно |  |  |
|  |  | 0 | 1 | 2 | 3 |  |  |

| **Модуль F** | **ПИТАНИЕ** |
| --- | --- |

**Сейчас я хочу поговорить о Ваших пищевых привычках**

| **F1.** | **Какой хлеб Вы ОБЫЧНО едите?** *Возможно несколько вариантов ответа.* | | | | | | |
| --- | --- | --- | --- | --- | --- | --- | --- |
| 1  2  3  4  5  5a | Не ем хлеб вообще *Переход к F4*  Черный  Белый  Белый с отрубями  Другое  Другое. Уточните .................... .................... | | | | | | |
| 97  98 | Затрудняюсь ответить  Отказ от ответа | | | | | | |
| **F2.** | **Какой вид жира ОБЫЧНО Вы намазываете на хлеб?** *Возможно несколько вариантов ответа***.** | | | | | | |
| 1  2  3  4  5  6 | Не использую жир  Спред с пониженным уровнем жирности (н-р, Rama Light)  Растительный маргарин  Сливочное масло  Спред (смесь молочных и растительных жиров)  Смалец/сало | | | | | | |
| 97  98 | Затрудняюсь ответить  Отказ от ответа | | | | | | |
| **F3.** | **Как часто в течение ПОСЛЕДНЕЙ НЕДЕЛИ Вы ели следующие продукты с хлебом (НЕ НА ГОРЯЧЕЕ, В КАЧЕСТВЕ ЗАКУСКИ/БУТЕБРОД)?** | | | | | | |
|  |  | | | | | | |
| **Количество раз в неделю** | |  |  |  |  |  |  |
| Сыр | | 0 | 1-2 | 3-4 | 5-7 | DA 97 | RA98 |
| Мясные продукты | | 0 | 1-2 | 3-4 | 5-7 | DA 97 | RA98 |
| Рыбу | | 0 | 1-2 | 3-4 | 5-7 | DA 97 | RA98 |
| Овощи (не считая картофеля) | | 0 | 1-2 | 3-4 | 5-7 | DA 97 | RA98 |

| **F4.** | **Как часто в течение ПОСЛЕДНЕЙ НЕДЕЛИ Вы ели следующие продукты на ГОРЯЧЕЕ (в горячем виде)?** | | | | | | |
| --- | --- | --- | --- | --- | --- | --- | --- |
|  |  | | | | | | |
| **Количество раз в неделю** | |  |  |  |  |  |  |
| Мясные продукты | | 0 | 1-2 | 3-4 | 5-7 | DA 97 | RA98 |
| Рыбу | | 0 | 1-2 | 3-4 | 5-7 | DA 97 | RA98 |
| Овощные/вегетариаские блюда (Не считая картофеля) | | 0 | 1-2 | 3-4 | 5-7 | DA 97 | RA98 |
| Бобовые (фасоль, горох) | | 0 | 1-2 | 3-4 | 5-7 | DA 97 | RA98 |

| **F5.** | **Какой вид жира Вы используете при приготовлении пищи дома?** *Возможно несколько вариантов ответа.* |
| --- | --- |
| 1 | Не использую жир |
| 2  3  4  5  6  7  8  9  9a | Растительный маргарин  Maргарин  Сливочное масло  Спред (смесь молочных и растительных жиров)  Смалец/сало  Оливковое масло  Подсолнечное масло или другое растительное масло (н-р, кукурузное, конопляное)  Другое  Другое. Уточните .................... .................... |
| 97  98 | Затрудняюсь ответить  Отказ от ответа |

| **F6.** | | **Как часто в течение ПОСЛЕДНЕЙ НЕДЕЛИ Вы употребляли овощи** (**Не считая картофеля) в качестве ГАРНИРA к горячей пище? Пожалуйста, исключите овощи, которые Вы указали выше** | | | | | | | | | | | | | | | | | |
| --- | --- | --- | --- | --- | --- | --- | --- | --- | --- | --- | --- | --- | --- | --- | --- | --- | --- | --- | --- |
|  | |  | | | | | | | | | | | | | | | | | |
| **Количество раз в неделю** | | | | | | |  | |  | |  | | |  | |  | |  | |
| Салат из овощей, свежие овощи | | | | | | | 0 | | 1-2 | | 3-4 | | | 5-7 | | DA 97 | | RA98 | |
| Вареные овощи (н-р, цветная капуста, морковь, свёкла) | | | | | | | 0 | | 1-2 | | 3-4 | | | 5-7 | | DA 97 | | RA98 | |
| Тушеные овощи | | | | | | | 0 | | 1-2 | | 3-4 | | | 5-7 | | DA 97 | | RA98 | |
|  | | | | | | |  | |  | |  | | |  | |  | |  | |
| **F7.** |  | | **Сколько фруктов и ягод Вы употребляете в день/в неделю? Порция: 1 фрукт/100 грамм?** | | | | | | | | | | | | | | | | |
| 1  2  3  4  5  6  7  8 |  | | Не ем фрукты  1-2 в неделю  3-4 в неделю  5-6 в неделю  1-2 в день  3-4 в день  5-6 в день  Более 6 в день | | | | | | | | | | | | | | | | |
| 97  98 |  | | Затрудняюсь ответить  Отказ от ответа | | | | | | | | | | | | | | | | |
| **F8.** |  | | | **Как часто в течение ПОСЛЕДНЕЙ НЕДЕЛИ Вы употребляли следующие напитки** | | | | | | | | | | | | | | | |
|  | | | | | Никогда | | | 1-3 раза в месяц | | 1-6 раз в неделю | | 1 раз в день | 2-3 раз в день | | 4+ раз в день | | DA 97 | | RA98 |
| Сок | | | | | | 1 | | 2 | | 3 | | 4 | 5 | | 6 | | DA 97 | | RA98 |
| Лимонад, кока-колу (не диетическую), пепси, другие сладкие напитки | | | | | | 1 | | 2 | | 3 | | 4 | 5 | | 6 | | DA 97 | | RA98 |
| Чай | | | | | | 1 | | 2 | | 3 | | 4 | 5 | | 6 | | DA 97 | | RA98 |
| Кофе | | | | | | 1 | | 2 | | 3 | | 4 | 5 | | 6 | | DA 97 | | RA98 |
| Энергитические напитки (н-р Red bull) | | | | | | 1 | | 2 | | 3 | | 4 | 5 | | 6 | | DA 97 | | RA98 |

| **Модуль G** | **УПОТРЕБЛЕНИЕ АЛКОГОЛЯ** |
| --- | --- |

**Теперь я задам Вам вопросы о Вашем употреблении алкоголя в течение последних 12 месяцев. Мы задаем эти вопросы всем, даже тем, кто не пьет, выпивает совсем немного или пьёт только по праздникам, таким как Новый год или День рождения. Пожалуйста, вспомните все случаи, когда Вы могли употреблять алкогольные напитки.**

**G1 – G10. Для каждого из видов алкоголя, обозначенного слева, укажите, как часто Вы употребляете данный вид алкоголя в течение последних 12 месяцев?** *Покажите карточку 6.*

|  | каждый день и чаще | почти каждый день | 3-4 раза в неделю | 1-2 раза в неделю | 1-3 раза в месяц | несколько раз в год | никогда или почти никогда | затрудняюсь ответить | отказ от ответа |
| --- | --- | --- | --- | --- | --- | --- | --- | --- | --- |
| **G1.** Любой алкоголь | 1 | 2 | 3 | 4 | 5 | 6 | 7 | 97 | 98 |

| **G2.** Пиво | 1 | 2 | 3 | 4 | 5 | 6 | 7 | 97 | 98 |
| --- | --- | --- | --- | --- | --- | --- | --- | --- | --- |
| **G3.** Вино (Не домашнего приготовления) | 1 | 2 | 3 | 4 | 5 | 6 | 7 | 97 | 98 |
| **G4.** Крепленое вино (н-р, портвейн) | 1 | 2 | 3 | 4 | 5 | 6 | 7 | 97 | 98 |
| **G5.** Крепкие напитки (водка, коньяк, виски, джин, ром, и т.д.) | 1 | 2 | 3 | 4 | 5 | 6 | 7 | 97 | 98 |
| **G6.** Cпиртосодержащие жидкости (спиртосодержащие жидкости, не предназначенные для употребления в качестве напитков, включая одеколоны, медицинские настойки, и другие подобные вещи, которые можно купить в магазинах, киосках, аптеках) | 1 | 2 | 3 | 4 | 5 | 6 | 7 | 97 | 98 |
| **G7.** Cпирт | 1 | 2 | 3 | 4 | 5 | 6 | 7 | 97 | 98 |
| **G8.** Cамогон | 1 | 2 | 3 | 4 | 5 | 6 | 7 | 97 | 98 |
| **G9.** Домашнее вино, брага | 1 | 2 | 3 | 4 | 5 | 6 | 7 | 97 | 98 |
| **G10.** Алкогольные коктейли в банках | 1 | 2 | 3 | 4 | 5 | 6 | 7 | 97 | 98 |

*Если респондент выбирает 7, 98 вариант ответа на вопросы о пиве, вине и крепких спиртных напитках, то соответственные вопросы G11,G12, G13 и G14,G15, G16 (and G17) им не задаются.*

| G11. | Если говорить о последних 12 месяцах, в случаях, когда Вы выпивали пиво, какое количество Вы обычно употребляли? *Возможен только один вариант ответа.* |
| --- | --- |
| 1 2 3 4 5 | Не пьете пиво  1 бутылку (0,5л) и меньше  2-4 бутылки (по 0,5л)  5-6 бутылки (по 0,5л)  Более 6 бутылок (по 0,5л) |
| 97  98 | Затрудняюсь ответить  Отказ от ответа |
|  |  |
| G12. | Если говорить о последних 12 месяцах, в случаях, когда Вы выпивали вино, какое количество Вы обычно употребляли? *Возможен только один вариант ответа.* |
| 1 2 3 4 5 6  97  98 | Не пьете вино  Менее 200г  200 – 400г  400 – 600г  600 – 1000г  Более 1 литра  Затрудняюсь ответить  Отказ от ответа |
|  |  |
| G13. | Если говорить о последних 12 месяцах, в случаях, когда Вы выпивали крепкие спиртые напитки (водку, коньяк, виски), какое количество Вы обычно употребляли? *Возможен только один вариант ответа.* |
| 1 2 3 4 5 6 7  8 | Не пьете эти напитки  До 50 г  50 – 100г  100 – 200г  200 – 300г  300 – 400г  400 – 500г  Более 500г |
| 97  98 | Затрудняюсь ответить  Отказ от ответа |
|  |  |
| G14. | Если говорить о последних 12 месяцах, в случаях, когда Вы выпивали пиво, какое самое максималное количество Вы употребляли? *Возможен только один вариант ответа.* |
| 1 2 3 4 5 | Не пьете пиво  1 бутылку (0,5л) и меньше  2-4 бутылки (0,5л)  5-6 бутылки (0,5л)  Более 6 бутылок (0,5л) |
| 97  98 | Затрудняюсь ответить  Отказ от ответа |
|  |  |
|  |  |
|  |  |
|  |  |
|  |  |
|  |  |
|  |  |
| G15. | Если говорить о последних 12 месяцах, в случаях, когда Вы выпивали вино, какое самое максималное количество Вы употребляли? *Возможен только один вариант ответа.* |
| 1 2 3 4 5 6 | Не пьете вино  Менее 200г  200 – 400г  400 – 600г  600 – 1000г  Более 1 литра |
| 97  98 | Затрудняюсь ответить  Отказ от ответа |
|  |  |
| G16. | Если говорить о последних 12 месяцах, в случаях, когда Вы выпивали крепкие спиртые напитки (водку, коньяк, виски), какое самое максималное количество Вы употребляли? *Возможен только один вариант ответа.* |
| 1  2  3  4  5  6  7  8 | Не пьете крепкие напитки  До 50 г  50 – 100г  100 – 200г  200 – 300г  300 – 400г  400 – 500г  Более 500г |
| 97  98 | Затрудняюсь ответить  Отказ от ответа |
|  |  |
| G17. | Как часто Вы выпиваете крепкие напитки, такие, как водка или коньяк, в большом количестве без закуски? *Возможен только один вариант ответа.* |
| 1 2 3 | Всегда  Иногда  Никогда или почти никогда |
| 97  98 | Затрудняюсь ответить  Отказ от ответа |
|  |  |
| G18. | Как часто Вы «перепиваете»? *Покажите карточку 7. Возможен только один вариант ответа.* |
| 1 2 3 4 5 6  7 | Каждый день  Несколько раз в неделю  Примерно один раз в неделю  Несколько раз в месяц  Примерно один раз в месяц  Меньше, чем один раз в месяц  Никогда или почти никогда |
| 97  98 | Затрудняюсь ответить  Отказ от ответа |
|  |  |
| G19. | Употребляете ли Вы когда-нибудь алкоголь в первой половине дня (до 12 часов)? *Возможен только один вариант ответа.* |
| 1 2 3 | Нет  Да, иногда  Да, часто |
| 97  98 | Затрудняюсь ответить  Отказ от ответа |
| G20. | Как часто у Вас бывает похмелье? *Покажите карточку 7. Возможен только один вариант ответа.* |
| 1 2 3 4 5 6 7 | Каждый день  Несколько раз в неделю  Примерно один раз в неделю  Несколько раз в месяц  Примерно один раз в месяц  Меньше, чем один раз в месяц  Никогда или почти никогда |
| 97  98 | Затрудняюсь ответить  Отказ от ответа |
|  |  |
| G21. | Как часто Вы не можете выполнять какие-то семейные или личные обязанности из-за употребления алкоголя? *Покажите карточку 7. Возможен только один вариант ответа.* |
| 1 2 3 4 5 6 7 | Каждый день  Несколько раз в неделю  Примерно один раз в неделю  Несколько раз в месяц  Примерно один раз в месяц  Меньше, чем один раз в месяц  Никогда или почти никогда |
| 97  98 | Затрудняюсь ответить  Отказ от ответа |
|  |  |
| G22. | Ложитесь ли Вы когда-либо спать (на ночь) не раздеваясь из-за алкогольного опьянения? *Покажите карточку 7. Возможен только один вариант ответа.* |
| 1 2 3 4 5 6 7 | Каждый день  Несколько раз в неделю  Примерно один раз в неделю  Несколько раз в месяц  Примерно один раз в месяц  Меньше, чем один раз в месяц  Никогда или почти никогда |
| 97  98 | Затрудняюсь ответить  Отказ от ответа |
|  |  |
|  | **Теперь я хочу спросить об эпизодах «запоя». Запой – это многодневное и непрерывное употребление алкоголя, при котором человек не участвует в социальной и профессиональной жизни.** |
|  |  |
| G23.  1  2  97  98 | **Были ли у Вас запои в течение последнего года?**  Да  Нет *Переход к 31*  Затрудняюсь ответить *Переход к 31*  Отказ от ответа *Переход к G31* |
| **G24.** | **Сколько запоев было у Вас в течение последнего года?** |
| 1 | 1 |
| 2 | 2-4 |
| 3 | 5-9 |
| 4 | 10 и болеe |
| 97  98 | Затрудняюсь ответить  Отказ от ответа *Переход к G31* |
| **G25.** | **Были ли у Вас запои в течение последнего месяца**? |
| 1 2 | Да  Нет |
| 97  98 | Затрудняюсь ответить  Отказ от ответа |
| **G26.** | **Как долго у Вас длится типичный запой?** |
| 1 2 3  4 | 2-3 дня  4-5 дней  6-9 дней  10 дней и более |
| 97  98 | Затрудняюсь ответить  Отказ от ответа |
|  |  |
| G27. | Во время последнего запоя употребляли ли Вы спиртосодержащие жидкости, не предназначенные для употребления в качестве напитков? Здесь мы имеем ввиду такие жидкости как одеколоны, медицинские настойки, непитьевой спирт. |
| 1 2 | Да *Переход к G31*  Нет *Переход к G31* |
| 97  98 | Затрудняюсь ответить *Переход к G31*  Отказ от ответа *Переход к G31* |
|  |  |
|  | *Вопросы для тех, кто не употреблял алкоголь в течение последних 12 месяцев.* |
|  |  |
| G28. | Употребляли ли Вы алкоголь когда-либо в большем (чем в настоящее время, в последние 12 месяцев) количестве? |
| 1 2 | Да  Нет |
| 97  98 | Затрудняюсь ответить *Переход к H1*  Отказ от ответа *Переход к H1* |
|  |  |
| G29. | Когда Вы перестали употреблять алкоголь? |
| 1  2  3  4 | 6 месяцев назад  От 6 до12 месяцев назад  От 1 года до 5 лет назад  5 и более лет назад |
| 97  98 | Затрудняюсь ответить  Отказ от ответа |
|  |  |
| G30. | Почему Вы перестали употреблять алкоголь? *Возможно несколько вариантов ответа.* |
| 1 | Боялся потерять работу |
| 2 | Порекомендовал врач |
| 3 | Прошел лечение от алкоголизма |
| 4 | Плохо себя чувствовал, употребляя алкоголь |
| 5 | Повлияли просьбы кого-то из близких или друзей |
| 6 | Были финансовые причины |
| 7 | Решили, что не хотите больше пить по другим причинам, связанным с состоянием здоровья |
| 7a | Другое. Уточните .................... .................... |
| 8  8a | Решили, что не хотите больше пить по другим причинам, не связанным с состоянием здоровья Другое. Уточните .................... .................... |
| 97  98 | Затрудняюсь ответить  Отказ от ответа |
| G31 | Получали ли Вы когда-либо помощь (или консультации) медиков, наркологов, других специалистов по поводу проблем с алкоголем? |
| 1 2 | Да  Нет Переход к G37 |
| 97  98 | Затрудняюсь ответить Переход к G37  Отказ от ответа Переход к G37 |
| G32. | Какого рода (какую) помощь Вы получили? *Возможно несколько вариантов ответа.* |
| 1  2  3  4  5  6  7  8 | Помощь в наркологическом диспансере, медикаментозую помощь (детоксификацию, купирование абстинентного синдрома)  Помощь нарколога на дому или в частной клинике (медикаментозная помощь, н-р, капельница)  Помощь психолога  Курс психотерапии  Курс поведенческой терапии  Групповая терапия (группы анонимных алкоголиков, 12-ти шаговые проргаммы)  Кодирование  Помощь знахарки/знахаря |
| 97  98 | Затрудняюсь ответить  Отказ от ответа |
|  |  |
| G33. | Получали ли Вы такую помощь (или консультацию) в течение последних 12 месяцев? |
| 1 2 | Да  Нет |
| 97  98 | Затрудняюсь ответить  Отказ от ответа |
|  |  |
| G34. | Обращались ли Вы (или направлялись) когда-либо в наркологический диспансер? |
| 1 2 | Да  Нет |
| 97  98 | Затрудняюсь ответить  Отказ от ответа |
|  |  |

Интервьюер: задавайте следующие вопросы тем, кто пьет спиртосодержащие жидкости несколько раз в год и чаще. Если респондент не употребляет спиртосодержащие жидкости переход к G37

| G35. | Когда Вы начали употреблять спиртосодержащие жидкости? *Возможен только один вариант ответа.* |
| --- | --- |
| 1  2  3  4 | В последний месяц  В последние 6 месяцев  В последний год  Больше года назад |
| 97  98 | Затрудняюсь ответить  Отказ от ответа |
|  |  |
|  |  |
|  |  |
|  |  |
|  |  |
|  |  |
|  |  |
| G36. | Какие спиртосодержащие жидкости Вы употребляете? *Возможно несколько вариантов ответа.* |
| 1  2  3  4  5  6  7  8  9  10  10a | Настойка боярышника  Трояр  Непитьевой спирт (технический, медицинский и т.д.)  Композиция  Тройной одеколон  Лосьон «Перец»  Лосьон "Зверобой"  Лосьон "Чистый свет"  Жидкость для мытья окон, другие моющие средства  Другие спиртовые настойки  Другое. Уточните .................... .................... |
| 97  98 | Затрудняюсь ответить  Отказ от ответа |

| G37. За последние 12 месяцев испытывали ли Вы следующее? | | Да | Нет | Затрудняюсь ответить | Отказ от ответа |
| --- | --- | --- | --- | --- | --- |
| a | Чувствовали ли Вы, что должны уменьшить употребление алкоголя? | 1 | 2 | 97 | 98 |
| b | Надоедали ли Вам люди, критикующие Вас за выпивку? | 1 | 2 | 97 | 98 |
| c | Вы чувствовали себя плохим или виноватым из-за Вашей выпивки? | 1 | 2 | 97 | 98 |
| d | Вы когда-нибудь выпивали первым делом утром, чтобы успокоить нервы или избавиться от похмелья? | 1 | 2 | 97 | 98 |

| **Модуль H** | **КУРЕНИЕ** |
| --- | --- |

**Сейчас я задам несколько вопросов о курении**

| **H1.** | **Курите ли Вы в настоящее время?** *Возможен только один вариант ответа.* |
| --- | --- |
| 1 2 3 | Нет и никогда не курил (а) *Переход к K1*  Нет, бросил (а) *Переход к H2*  Да, курю *Переход к H3* |
| 97  98 | Затрудняюсь ответить *Переход к H3*  Отказ от ответа |
|  |  |
| H2. | Как давно Вы бросили курить? *Возможен только один вариант ответа.* |
| 1 2 3 4 | Не более1 года назад  От 1 до 5 лет назад  От 5 до 10 лет назад  Более 10 лет назад |
| 97  98 | Затрудняюсь ответить  Отказ от ответа |
|  |  |
| H3. | Что Вы курите (курили)чаще всего? *Возможен только один вариант ответа.* |
| 1 2 3 4  4a | Папиросы  Сигареты с фильтром  Сигареты без фильтра  Другое  Другое. Уточните .................... .................... |
| 97  98 | Затрудняюсь ответить  Отказ от ответа |
|  |  |
| H4. | **Когда Вы курите (курили), сколько Вы обычно выкуриваете (выкуривали) за день?** *Возможен только один вариант ответа.* |
| 1 2 3 | 10 сигарет и менее  Более 10, но не более 20 сигарет  Более 20-сигарет |
| 97  98 | Затрудняюсь ответить  Отказ от ответа |
| H5. | **В каком возрасте Вы начали курить регулярно?** ________Лет |
| 97  98 | Затрудняюсь ответить  Отказ от ответа |
|  |  |
| H6. | Советовали/рекомендовали ли Вам когда-либо врачи (участковый врач, терапевт, кардиолог, другие специалисты) бросить курить? |
| 1  2 | Да *Переход к H7*  Нет *Переход к K1* |
| 97  98 | Затрудняюсь ответить  Отказ от ответа |
|  |  |
|  |  |
|  |  |
| **H7.** | **Предложена ли была Вам помощь для того, чтобы бросить курить?** |
| 1  2 | Да  Нет *Переход к K1* |
| 97  98 | Затрудняюсь ответить  Отказ от ответа *Переход к K1* |
| **H8.** | **Какая именно помощь была предложена?** *Возможно несколько вариантов ответа***.** |
| 1 | Направление на никотиновую заместительную терапию |
| 2 | Направление на поведенческую терапию |
| 3 | Направление на 12-ти шаговую программу |
| 4 | Кодирование |
| 5 | Материальная мотивация |
| 6  6a | Другое  Другое. Уточните .................... .................... |
| 97  98 | Затрудняюсь ответить  Отказ от ответа |

| **Модуль I** | **ДОМОХОЗЯЙСТВО** |
| --- | --- |

Сейчас мне хотелось бы задать несколько дополнительных вопросов о людях, которые проживают с Вами и Ваших жилищных условиях.

| I1. | Как давно Вы проживаете по данному адресу? *Возможен только один вариант ответа.* |
| --- | --- |
| 1 2  3  4 | Не более 6 месяцев  От 6 до 12 месяцев  От 1 года до 2 лет  Более 2 лет |
| 97  98 | Затрудняюсь ответить  Отказ от ответа |
| I2. | Сколько человек в настоящее время проживает вместе с Вами*,* исключая Вас? _____ Человек |
|  | |
| 97  98 | Затрудняюсь ответить  Отказ от ответа |

| **I3.** **Сейчас я задам несколько вопросов о каждом человеке, проживающем с Вами, начиная с самого старшего и дальше по старшинству** | | | | | | | | | | | | | | | **Коды для родственных отношений**: 1 Супруг/а или "проживающая/й в гражданском браке" 2 Родитель 3 Брат 4 Сестра  5 Дочь 6 Жена сына  7 Сын 8 Муж дочери 9 Внук 10 Другие близкие родственники 11 Неродственники  97 Затрудняюсь ответить  98 Отказ от ответа    **Коды для уровня образования:**  1 Неполное среднее 2 Полное среднее 3 Начальное профессиональное (без среднего образования)  4 Профессиональное (со средним образованием) 5 Среднее специальное  6 Незаконченное высшее 7 Высшее 8 Не относится (ребенок)  97 Затрудняюсь ответить 98 Отказ от ответа    **Коды для вклада в семейный бюджет:** 1 Да  2 Нет  97 Затрудняюсь ответить 98 Отказ от ответа    **Коды для возраста:**  997 Затрудняюсь ответить  998 Отказ от ответа |
| --- | --- | --- | --- | --- | --- | --- | --- | --- | --- | --- | --- | --- | --- | --- | --- |
| Кем приходится Вам этот человек |  | Пол | |  | Возраст (полных лет) |  | Уровень образования | | |  | Вносит ли вклад в семейный бюджет | | |  |  |
|  |  | M | Ж |  |  |  |  |  |  |  |  |  |  |  |  |
|  | | | | | | | | | | | | | | |  |
|  |  | M | Ж |  |  |  |  |  |  |  |  |  |  |  |  |
|  | | | | | | | | | | | | | | |  |
|  |  | M | Ж |  |  |  |  |  |  |  |  |  |  |  |  |
|  | | | | | | | | | | | | | | |  |
|  |  | M | Ж |  |  |  |  |  |  |  |  |  |  |  |  |
|  | | | | | | | | | | | | | | |  |
|  |  | M | Ж |  |  |  |  |  |  |  |  |  |  |  |  |
|  | | | | | | | | | | | | | | |  |
|  |  | M | Ж |  |  |  |  |  |  |  |  |  |  |  |  |
|  | | | | | | | | | | | | | | |  |
|  |  | M | Ж |  |  |  |  |  |  |  |  |  |  |  |  |
|  | | | | | | | | | | | | | | |  |

| **Несколько вопросов о Ваших жилищных условиях.** | | | | | | | | |  |  |
| --- | --- | --- | --- | --- | --- | --- | --- | --- | --- | --- |
| **I4.** | В каком типе жилья Вы проживаете? *Возможен только один вариант ответа.* | | | | | | | |  |  |
| 1 2 3 4 5 6  6a | Общежитие  Комната (комнаты) в коммунальной квартире  Отдельная квартира  Часть дома  Отдельный дом  Другое  Другое. Уточните .................... .................... | | | | | | | |  |  |
| 97  98 | Затрудняюсь ответить  Отказ от ответа | | | | | | | |  |  |
|  |  | | | | | | | |  |  |
| I5. | Какова общая площадь Вашего жилья? Количество квадратных метров.................... | | | | | | | |  |  |
| 97  98 | Затрудняюсь ответить  Отказ от ответа | | | | | | | |  |  |
| I6. | **Сколько комнат в Вашей квартире/ доме?** Количество комнат..................................... | | | | | | | |  |  |
| 97  98 | Затрудняюсь ответить  Отказ от ответа | | | | | | | |  |  |
|  |  | | | | | | | |  |  |
| I7. | Какими из перечисленных ниже удобств/ вещей располагает Ваша семья? *Возможно несколько вариантов ответа*. | | | | | | | |  |  |
|  |  | | Да | | Нет | Затрудняюсь ответить | Отказ от ответа |  |  |  |
| 1 | Автомобиль | | 1 | | 2 | 97 | 98 |  |  |  |
| 2 | Горячее водоснабжение | | 1 | | 2 | 97 | 98 |  |  |  |
| 3 | Центральное отопление | | 1 | | 2 | 97 | 98 |  |  |  |
| 4 | Интернет | | 1 | | 2 | 97 | 98 |  |  |  |
| 5 | Посудомоечная машина | | 1 | | 2 | 97 | 98 |  |  |  |
| 6 | Современная стиральная машина | | 1 | | 2 | 97 | 98 |  |  |  |
|  |  | |  | |  |  |  | |  |  |
| I8. | | Есть ли у Вас дача? | | | | | | | | |
| 1  2  97  98 | | Да  Нет  Затрудняюсь ответить  Отказ от ответа | | | | | | | | |
|  | |  | | | | | | | | |
| I9. | | Выберите высказывание, которое наиболее точно описывает финансовые возможности семьи в течение последнего года *Покажите карточку 8. Возможен только один вариант ответа.* | | | | | | | | |
| 1 | | Денег не хватает даже на продукты, "едва сводим концы с концами" | | | | | | | | |
| 2 | | На продукты денег хватает, но покупка одежды вызывает затруднения | | | | | | | | |
| 3 | | Денег хватает на продукты и одежду, но покупка крупной бытовой техники затруднительна | | | | | | | | |
| 4 | | Можем без труда приобретать крупную бытовую технику, но покупка нового легкового автомобиля затруднительна | | | | | | | | |
| 5 | | Хватает доходов на новый легковой автомобиль, однако покупка квартиры или дома (иной недвижимости) является для нас затруднительной | | | | | | | | |
| 6 | | Материальных затруднений не испытываем; при необходимости можем купить квартиру, дом (иную недвижимость) | | | | | | | | |
| 97  98 | | Затрудняюсь ответить  Отказ от ответа | | | | | | | | |
| **Модуль K** | | | | **ПСИХОСОЦИАЛЬНЫЕ ФАКТОРЫ** | | | | | |  |

| **K1.** **Происходили ли с Вами следующие события или проблемы в течение последних 6 месяцев?** | НЕТ | ДА | Затрудняюсь ответить | Отказ от ответа |
| --- | --- | --- | --- | --- |
| Вы перенесли тяжелое заболевания, травму, или физическое насилие? | 2 | 1 | 97 | 98 |
| Кто-то из Ваших близких перенес тяжелое заболевания, травму, или физическое насилие? | 2 | 1 | 97 | 98 |
| Умер кто-то из Ваших родителей, ребенок, супруг (а), проживающий (ая) в гражданском браке | 2 | 1 | 97 | 98 |
| Умер кто-то близкий Вашей семье: друг, другой родственник (тётя, бабушка, двоюродный брат/сестра) | 2 | 1 | 97 | 98 |
| Вы расстались/пережили развод из-за семейных сложностей | 2 | 1 | 97 | 98 |
| Вы разорвали крепкие взаимоотношения | 2 | 1 | 97 | 98 |
| У Вас возникли серьeзные проблемы с близким другом, родственником, соседями | 2 | 1 | 97 | 98 |
| Вы стали безработным/ой или безрезультатно искали работу больше месяца | 2 | 1 | 97 | 98 |
| Вас уволили | 2 | 1 | 97 | 98 |
| У Вас возникли серьезные финансовые проблемы | 2 | 1 | 97 | 98 |
| У Вас были проблемы с полицией и Вы должны были предстать перед судом | 2 | 1 | 97 | 98 |
| У Вас украли что-то ценное или Вы потеряли что-то ценное для Вас | 2 | 1 | 97 | 98 |

**Сейчас мы подходим к заключительному разделу нашего опроса. Вначале я задам Вам несколько вопросов о событиях, которые могли случиться с Вами и Вашими близкими за последние 6 месяцев.**

| **K2. Наносили ли Вам телесные повреждения в течение последнего года?** | |
| --- | --- |
| 1  2 | Да  Нет |
| 97  98 | Затрудняюсь ответить  Отказ от ответа |
|  |  |
| K3. | Каковы Ваши взаимоотношения в Вашей семье? *Возможен только один вариант ответа.* |
| 1  2  3 | Гармоничные, спокойные  Иногда случаются ссоры и конфликты  Часто бывают ссоры и конфликты |
| 97  98 | Затрудняюсь ответить  Отказ от ответа |
| **K4.** | **Насколько Вы согласны или не согласны с каждым из следующих утверждений?** *Покажите карточку 9.* |
| 1 | Дело мужа - зарабатывать деньги, а дело жены - присматривать за домом и детьми. |
| 2 | Мужчины и женщины должны играть равные роли в политике |
| 3 | Мужчины больше, чем женщины, подходят для того, чтобы быть руководителями |
|  | 1 Абсолютно согласен  2 Согласен  3 И да и нет  4 Не согласен  5 Абсолютно не согласен |
| 97  98 | Затрудняюсь ответить  Отказ от ответа |
| **K5.** | **В наше время высказываются разные мнения о том, кто должен быть главой семьи. Какое суждение ближе всего к Вашим представлениям по этому вопросу?** |
| 1 | Муж должен нести ответственность за семью, быть её главой, а жена - быть покорной мужу |
| 2 | Муж и жена должны быть равными в своих правах, нести равную ответственность за семью |
| 3 | Жена должна нести ответственность за семью, быть её главой |
|  |  |
| 97  98 | Затрудняюсь ответить  Отказ от ответа |
| **K6.** | **Есть ли у вас кто-то, с кем Вы могли бы поговорить по душам, о личном, которые могут Вас выслушать в трудную минуту, когда вам это нужно?** |
| 1  2 | Да  Нет |
| 97  98 | Затрудняюсь ответить  Отказ от ответа |
|  |  |
| **K7.** | **Есть ли у вас кто-то, кто может Вам помочь, физически, материально когда вам это нужно, например, забрать ребенка из школы, отвезти в больницу?** |
| 1 | Да |
| 2 | Нет |
| 97  98 | Затрудняюсь ответить  Отказ от ответа |
| **K8.** | **Вы считаете, что большинству людей можно доверять или Вы склоняетесь к мнению, что даже излишняя осторожность в отношениях с людьми не помешает?**  *Пожалуйста, дайте ответ по шкале от 0 до 10, где «0» означает «даже излишняя осторожность не помешает», а «10» - «большинству людей можно доверять».*  *0................................................ Покажите карточку 10…….……............................10*  даже излишняя осторожность не помешает большинству людей можно доверять |
| 97  98 | Затрудняюсь ответить  Отказ от ответа |
| **K9.** | **Как Вы думаете, большинство людей постарались бы Вас использовать, если бы им представилась такая возможность, или же, на Ваш взгляд, люди постараются вести себя с Вами честно**/**справедливо?**  *0................................................ Покажите карточку 11…….……............................10*  Большинство людей постарались бы меня использовать большинство людейпостараются вести себя честно |
|  |  |
| 97  98 | Затрудняюсь ответить  Отказ от ответа |
| **K10.** | **По Вашему мнению, в большинстве случаев люди стараются помогать друг другу или они чаще всего заботятся только о себе?**  *Выбирите одно число*  *0................................................ Покажите карточку 12…….……............................10*  *Чаще всего В большинстве случаев стараются помогать друг другу*  *люди заботятся*  *только о себе* |
| 97  98 | Затрудняюсь ответить  Отказ от ответа |
| **K11.** | **Насколько Вы согласны или не согласны с каждым из следующих утверждений?** *Покажите карточку 13***.** *Пожалуйста, дайте ответ по шкале от 0 до 7.*    Если у меня возникнут новые проблемы со здоровьем, я смогу справиться с ними  0...............1...............2..............3............4...............5...............6..............7  Абсолютно согласен Абсолютно не согласен  Я знаю, что сделать, чтобы предотвратить ухудшение моего здоровья  0...............1...............2..............3............4...............5...............6..............7  Абсолютно согласен Абсолютно не согласен  Если я буду вести здоровый образ жизни, я смогу предотвратить серьезные заболевания  0...............1...............2..............3............4...............5...............6..............7  Абсолютно согласен Абсолютно не согласен  В конце концов, я несу ответственность и заботу о состоянии своего здоровья  0...............1...............2..............3............4...............5...............6..............7  Абсолютно согласен Абсолютно не согласен |

| 97  98 | Затрудняюсь ответить  Отказ от ответа |
| --- | --- |

| **Модуль X** | **ОБСТОЯТЕЛЬСТВА ИНТЕРВЬЮ** |
| --- | --- |

*Последние вопросы – об обстоятельствах проведения интервью. На них отвечает только сам интервьюер. Их зачитывать не надо.*

| **X1.** | **Как Вы оцениваете надежность ответов в данном интервью?** |
| --- | --- |
|  |  |
| 1 | Удовлетворительно |
| 2 | Не вполне удовлетворительно. Например, не на все вопросы следовали ответы, или возможно, небольшие перерывы влияли на качество ответов |
| 3 | Плохо. Например, на многие вопросы не было ответов, или, возможно, частые (постоянные) перерывы влияли на качество ответов. |
|  |  |
| **X2.** | **Присутствовал ли кто-либо из других людей (кроме респондента) в том же помещении во время проведения интервью?** |
|  |  |
| 1 | Да |
| 2 | Нет |
| X3. | **Были ли перерывы в ходе интервью?** |
| 1 2 | Да  Нет *Переход к X5* |
| X4. | Опишите причины перерывов и их длительность……………….. |
| X5. | **Любые другие комментарии, включая указания на вопросы, которые казались трудными респонденту**………………….. |
| X6. | **Место проведения интервью.** |
|  |  |
| 1 | Дома у респондента |
| 2 | У респондента на работе |
| 3 | Возле места проведения медицинского обследования |
| 4  4a | В другом месте  Другое. Уточните .................... .................... |

| **Модуль Y** | **ПРИГЛАШЕНИЕ НА МЕДИЦИНСКОЕ ОБСЛЕДОВАНИЕ** |
| --- | --- |

| **Y1.** | **Согласился ли респондент пройти медицинское обследование?** |
| --- | --- |
|  |  |
| 1 | Нет |
| 2 | Потенциально заинтересован, но время визита не назначено |
| 3 | Да, респондент заинтересован и время визита не назначено |
|  |  |
| **Y2.** | **Получено ли письменное согласие респондента?** |
|  |  |
| 1 | Да |
| 2 | Нет *Переход к Y2a* |
|  |  |
| **Y2a.** | **Если письменное согласие не получено, уточните причину…………….** |
|  |  |
| **Y3.** | **Видели ли Вы информационный ролик о данном исследовании?** |
| 1  2 | Да  Нет |
| 97  98 | Затрудняюсь ответить  Отказ от ответа |
|  |  |
| **Y4.** | **Видели ли Вы другую информацию (например, баннеры на улице) о данном исследовании?** |
| 1  2 | Да  Нет |
| 97  98 | Затрудняюсь ответить  Отказ от ответа |
