# Supplementary material for: Know Your Heart: Rationale, design and conduct of a cross-sectional study of cardiovascular structure, function and risk factors in 4500 men and women aged 35-69 years from two Russian cities, 2015-18
Source: Wellcome Open Res. 2018 Dec 3;3:67. Originally published 2018 Jun 4. [Version 3] doi: 10.12688/wellcomeopenres.14619.3 (PMC6073094; doi:10.12688/wellcomeopenres.14619.3)
Supplement: Supplementary file 3 [file wellcomeopenres-3-16294-s0002.tgz › d9a03f66-5fce-45a3-8a2e-d18febd22997_Supplementary_file_3._Questionnaires_and_data_collection_tools.zip!/questionnaires_181121_publish_version2/181118_Baseline_survey_narcology_ENG.docx]

|  |  |
| --- | --- |

**Baseline questionnaire for the patients from the in-patient alcohol addiction treatment facility**

International Project on Cardiovascular Disease in Russia

2015-2018

Published version 2.0 November 2018

Russia has one of the highest rates of cardiovascular disease in the world. The International Project on Cardiovascular Disease in Russia (IPCDR) was set up to understand the reasons for this. A substantial component of this study was the Know Your Heart Study devoted to characterising the nature and causes of cardiovascular disease in Russia by conducting large cross-sectional surveys in two Russian cities Novosibirsk and Arkhangelsk. The study population was 4542 men and women aged 35-69 years recruited from the general population. Fieldwork took place between 2015-18. There were two study components: 1) a baseline interview to collect information on socio-demographic characteristics and cardiovascular risk factors, usually conducted at home, and 2) a comprehensive health check at a primary care clinic which included detailed examination of the cardiovascular system.

The International Project on Cardiovascular Disease in Russia (IPCDR) project was funded in part by a Wellcome Trust Strategic Award [100217]. The project was also funded by the Arctic University of Norway, UiT in Tromsø; Norwegian Institute of Public Health; the Norwegian Ministry of Health and Social Affairs.

Development of the questionnaire was led by the London School of Hygiene & Tropical Medicine in collaboration with the Northern State Medical University, Arkhangelsk, Novosibirsk State Medical University, the Research Institute of Internal and Preventive Medicine - Branch of IC&G SB RAS, Novosibirsk and colleagues from other institutions.

This is a paper version questionnaire used for the baseline interview modified for use in a sub sample of study participants recruited from inpatients receiving treatment for alcohol problems although the interview was administered electronically using Computer Assisted Personal Interviewing (CAPI) devices.

Further details about the study are available here: Know Your Heart: Rationale, design and conduct of a cross-sectional study of cardiovascular structure, function and risk factors in 4500 men and women aged 35-69 years from two Russian cities, 2015-18
Cook S, Malyutina S, Kudryavtsev AV, Averina M, Bobrova N, Boytsov S, Brage S, Clark T *et al.*

Content

[Module A. Socio-demographic factors	4](#_Toc512505986)

[Module G. Alcohol consumption	9](#_Toc512505991)

[Module D. Use of health care services.	27](#_Toc512505994)

[Module E. Depressions and Anxiety.	31](#_Toc512505995)

[Module H. Smoking	33](#_Toc512505997)

[Module K. Psychosocial factors	35](#_Toc512506001)

Section X. Interview context	36

Date of the interview__ __ / __ __ / __ __ __ __ Interviewer ID___Respondent ID________

| **Module A** | **SOCIO-DEMOGRAPHIC FACTORS** |
| --- | --- |

I would like to begin by asking you some questions about yourself.

| A1. | How old are you? _________Years |
| --- | --- |
|  | |
| 97  98 | Difficult to answer  Refuse to answer |
|  |  |
| A2. | What is your date of birth? __ __ / __ __ / __ __ __ __ |
|  | |
| 97  98 | Difficult to answer  Refuse to answer |
| **A3.** | Interviewer! Please type in the gender of the respondent. |
| 1  2 | Male  Female |
|  |  |
| A4. | What is your nationality? *Please choose the single most appropriate answer.* |
| 1 2  3  4  5  5a | Russian  Ukraininan  Nenets  Belorus  Other  Other. Specify .................... .................... |
| 97  98 | Difficult to answer  Refuse to answer |
|  |  |
| A5. | Please could you tell me where you were born? *Please choose the single most appropriate answer.* |
| 1 2 3 4 5 | Arkhangelsk city  Another part of Arkhangelsk region  A different oblast of Russia  A part of the former Soviet Union outside Russia  Outside the former Soviet Union |
| 97  98 | Difficult to answer  Refuse to answer |
|  |  |
| A6. | How long have you continuously lived in Arkhangelsk? *Please choose the single most appropriate answer.* |
| 1  2  3  4  5 | Up to 12 months  More than 12 months-up to 5 years  More than 5, up to 10 years  More than 10 years, but not your whole life  Since birth (excluding army and temporary periods away) |
| 97 | Difficult to answer |
| 98 | Refuse to answer |
| **I4_a.** | **Where did you live before this hopitalization?** |
| 1a | Did not have specific place to live (homeless) |
| 1 | Dormitory |
| 2 | Room in communal flat |
| 3 | Apartment |
| 4 | Part of the house |
| 5 | A house |
| 6 | Other |
| 6a | Other. Specify .................... .................... |
| 97  98 | Difficult to answer  Refuse to answer |
| A7. | What is your current marital status? Are you... *Please choose the single most appropriate answer*. |
| 1 2 3 4 5 | Living together with a spouse/partner in a registered marriage  Living together with a spouse/partner but not in a registered marriage  Divorced or separated  Widower  Never married |
| 97  98 | Difficult to answer  Refuse to answer |
| A8. | How many children do you have? *Please choose the single most appropriate answer.* |
| 1  2  3  4  5 | 0  1  2  3  4 or more |
| 97  98 | Difficult to answer  Refuse to answer  I wo |

I would now like to ask you about your education and occupation

| A9. | What is your level of education? *Please choose the single most appropriate answer. SHOW CARD 1.* | |
| --- | --- | --- |
| 1 2 3 4 5  6  7 | Incomplete secondary  Complete secondary  Professional school (without secondary degree, PTU)  Professional school and secondary (e.g. PTU and secondary education)  Specialised secondary (e.g. technicum, college, medical, pedagogical college)  Incomplete higher  Higher | |
| 97  98 | Difficult to answer  Refuse to answer | |
|  |  | |
| **A10.** | **Are you studying at the moment (are you a student)?** | |
| 1 | Yes | |
| 2 | No | |
| 97  98 | Difficult to answer  Refuse to answer | |
| **A11.** | **Are you….** | |
| 1 | Retired, except for retirement due to invalidity | |
| 2 | Retired due to invalidity | |
| 3 | None of the above | |
| 97  98 | Difficult to answer  Refuse to answer | |
|  |  | |
| **A12.** | **Are you in regular paid work?** | |
| 1 | Yes | |
| 2 | No *Go to A14* | |
| 97  98 | Difficult to answer  Refuse to answer | |
|  |  | |
| **A13.** | **During the past 12 months how many months did you work on this job?** | |
|  | Number of months.................... | |
| 97  98 | Difficult to answer  Refuse to answer | |
|  |  | |
| **A14.** | **Are you…** *Ask only those who are not in regular paid work (A12-NO)* | |
| 1 | In irregular paid work | |
| 2 | Unemployed, seeking work | |
| 3 | Unemployed, not seeking work | |
| 4 | Housewife | |
| 5  5a | Other  Other. Specify .................... .................... | |
| 6 | None of the above | |
| 97  98 | Difficult to answer  Refuse to answer | |
|  |  | |
| **A15.** | **How long ago did you cease this regular employment?** | |
| 1  2  3  4  5  6 | Have never been in regular paid employment  Within the past week  More than 1, up to 4 weeks ago  More than 1, up to 6 months ago  More than 6, up to 12 months ago  More than 1 year ago | |
| 97  98 | Difficult to answer  Refuse to answer | |
|  |  | |
| **A16.** | **What was the main reason for ceasing regular paid employment?** *Please choose the single most appropriate answer.* | |
| 1 | Started to study | |
| 2 | Retired | |
| 3 | Could not find a job after finishing education | |
| 4 | Was made redundant | |
| 5 | A temporary job ended | |
| 6 | Was fired | |
| 7 | Gave up voluntarily due to unsatisfactory work salary/work conditions | |
| 8 | Have never been in regular paid employment/have always worked in temporary jobs | |
| 9 | Gave up work because of ill health | |
| 10 | Gave up work to take care of children, other people | |
|  |  | |
| 11  11a | Gave up my job for other reasons  Other. Specify .................... .................... | |
| 97  98 | Difficult to answer  Refuse to answer | |
|  |  | |
| I would now like to ask you some questions about your main regular employment over the past year or in the last period that you were working. | | |
| **A17.** | What was your main occupation during the past year or in the last period that you were in regular paid work? *Interviewer types in full description (e.g. teacher in primary school).*  Your occupation:................................................................. | |
| **A18. Which of the descriptions best describe your work?** *Please choose the single most appropriate answer SHOW CARD 2.* | | |
| 1 2 3 4 5 6 7  8  9  10  10a | Legislator, Senior official or Manager  Professional  Technician or associate professional  Clerk  Service or sales worker  Craft and related trades workers  Plant/machine operator or assembler  Elementary worker  Military/armed forces  Other  Other. Specify .................... .................... | |
| 97  98 | Difficult to answer  Refuse to answer | |
| **A19.** | **Have you missed any days of work due to your own ill health in the last 12 months?** *(Asked only those who were employed last 12 months).* | |
| 1  2 | Yes  No *Go to A21* | |
| 97  98 | Difficult to answer  Refuse to answer | |
| **A20** | **Please specify how many days have you missed?** | |
| 1 | 1-3 days |  |
| 2 | 4-5 days |  |
| 3 | 6-10 days |  |
| 4 | 11+ days |  |
| 97 | Difficult to answer |  |
| 98 | Refuse to answer |  |
|  |  | |
| A21 | Do you have currently officialy registered disability? | |
| 1  2 | Yes  No *Go to I9* | |
| 97  98 | Difficult to answer *Go to I9*  Refuse to answer *Go to I9* | |
| A22. | How long ago were you registered? | |
| 1 | Less than 6 months ago | |
| 2  3  4  5  6 | 6 months to 12 months ago  More than 1, up to 5 years ago  More than 5, up to 10 years ago  More than 10 years ago but not my whole life  Have always been disabled | |
| 97  98 | Difficult to answer  Refuse to answer | |
|  |  | |
| A23. | What is the class of the disability at the moment? | |
| 1 2 3 | Class 1  Class 2  Class 3 | |
| 97  98 | Difficult to answer  Refuse to answer | |
| I9. | Which of the phrases below best describes this household’s financial situation during the past year? *SHOW CARD 3.* | |
| 1  2  3  4  5  6 | There is not even enough money for food, it’s difficult to make ends meet  We have enough money for food, but we find it difficult to afford clothes and other items  We have enough money for food and clothes, but would find it difficult to buy large domestic appliances  We can afford to buy large domestic appliances, but would find it difficult to buy a large new car  We can afford to buy a large new car, but would find it difficult to buy a flat or a house (or other property)  We have no financial constraints. We can afford to buy a flat or a house (or other property). | |
|  |  | |
| 97  98 | Difficult to answer  Refuse to answer | |

| **Module G** | **ALCOHOL CONSUMPTION** |
| --- | --- |

Now I will ask you about your alcohol consumption in the past 12 months. We ask these questions everyone even those who do not drink, drink little a bit or drink only on holidays such as New Year or at the birthday. Please think about any occasions you could consume alcohol beverages.

**G1 – G10. For each type of drink listed in the left hand column, please indicate how often each was usually drunk in the last 12 months.** *SHOW CARD 4.*

|  | Every day or more often | Nearly every day | 3-4 times per week | Once or twice a week | 1-3 times a month | A few times a year | Never or almost never | Difficult to answer | Refuse to answer |
| --- | --- | --- | --- | --- | --- | --- | --- | --- | --- |
| **G1.** Any alcohol (beer, vodka, anything else containing alcohol) | 1 | 2 | 3 | 4 | 5 | 6 | 7 | 97 | 98 |

| **G2.** Beer | 1 | 2 | 3 | 4 | 5 | 6 | 7 | 97 | 98 |
| --- | --- | --- | --- | --- | --- | --- | --- | --- | --- |
| **G3.** Wine (not home produced) | 1 | 2 | 3 | 4 | 5 | 6 | 7 | 97 | 98 |
| **G4.** Fortified wine | 1 | 2 | 3 | 4 | 5 | 6 | 7 | 97 | 98 |
| **G5.** Spirits (vodka, cognac, whisky, gin, rum, etc.) | 1 | 2 | 3 | 4 | 5 | 6 | 7 | 97 | 98 |
| **G6.** Ethanol-containing substances (These are manufactured substances not intended for drinking, including eau de colognes and medicinal tinctures as well as other things. They may be found in shops, chemists and kiosks) | 1 | 2 | 3 | 4 | 5 | 6 | 7 | 97 | 98 |
| **G7.** Spirit not intended for drinking | 1 | 2 | 3 | 4 | 5 | 6 | 7 | 97 | 98 |
| **G8.** Homemade samogon | 1 | 2 | 3 | 4 | 5 | 6 | 7 | 97 | 98 |
| **G9.** Homemade wine, braga | 1 | 2 | 3 | 4 | 5 | 6 | 7 | 97 | 98 |
| **G10.** Alcoholic cocktails( premixed bottles) | 1 | 2 | 3 | 4 | 5 | 6 | 7 | 97 | 98 |

| G10_1 | **How many abstinent days did you have in the past three months prior to this admission?**______ |
| --- | --- |
| 97  98 | Difficult to answer  Refuse to answer |
|  |  |
| G11. | During last 12 months how much beer did you usually drink on one occasion? (‘occasion’ means a single continuous period of drinking). |
| 1 2 3 4 | 1 bottle (0.5l) or less  2-4 bottles (0.5l)  5-6 bottles (0.5l)  More than 6 bottles(0.5l) |
| 4a Please specify___________Bottles (if more than 6 bottles) | |
|  |  |
| 97  98 | Difficult to answer  Refuse to answer |
|  |  |
| G12. | During last 12 months how much wine did you usually drink on one occasion? |
| 1 2 3 4 5 | Up to 200g  Between 200 - 400g  Between 400 - 600g  Between 600 - 1000g  More than 1 litre |
| 5a Please specify___________Grams (if more than 1000 grams) | |
|  |  |
| 97  98 | Difficult to answer  Refuse to answer |
|  |  |
| G13. | During last 12 months how much spirits, such as vodka or other strong drinks, do you usually drink on one occasion? |
| 1 2 3 4 5 6 7 | Up to 50g  Between 50 – 100g  Between 100 - 200g  Between 200 - 300g  Between 300 - 400g  Between 400 - 500g  More than 500g |
| 7a Please specify___________ Grams (if more than 500 grams) | |
|  |  |
| 97  98 | Difficult to answer  Refuse to answer |
|  |  |
| G14. | During last 12 months what was the maximum quantity of beer ever drunk on one occasion? |
| 1 2 3 4 | 1 bottle (0.5l) or less  2-4 bottles (0.5l)  5-6 bottles (0.5l)  More than 6 bottles (0.5l) |
| 4a | Please specify_______________________Bottles (if more than 6 bottles) |
| 97  98 | Difficult to answer  Refuse to answer |
|  |  |
| G15. | During last 12 months what was the maximum quantity of wine ever drunk on one occasion? |
| 1 2 3 4 5 | Up to 200g  Between 200 - 400g  Between 400 - 600g  Between 600 - 1000g  More than 1 litre |
| 5a Please specify___________Grams (if more than 1000 grams) | |
|  |  |
| 97  98 | Difficult to answer  Refuse to answer |
|  |  |
| G16. | During last 12 months what was the maximum quantity of spirits ever drunk on one occasion? |
| 1  2  3  4  5  6  7 | Up to 50g  Between 50 – 100g  Between 100 - 200g  Between 200 - 300g  Between 300 - 400g  Between 400 - 500g  More than 500g |
| 7a Please specify___________Grams (If more than 500 grams) | |
|  |  |
| 97  98 | Difficult to answer  Refuse to answer |
|  |  |
| G17. | Do you ever drink large quantities of spirits without also eating some food at the same sitting? |
| 1 2 3 | Always  Sometimes  Rarely/never |
| 97  98 | Difficult to answer  Refuse to answer |
|  |  |
| G18. | How often do you become excessively drunk? *SHOW CARD 5.* |
| 1 2 3 4 5 6  7 | Every day  Several times a week  Once a week  Several times a month  Once a month  Less than once a month  Never or almost never |
| 97  98 | Difficult to answer  Refuse to answer |
|  |  |
|  |  |
| G19. | Do you ever drink alcohol before noon? |
| 1 2 3 | No  Yes, occasionally  Yes, frequently |
| 97  98 | Difficult to answer  Refuse to answer |
|  |  |
| G20. | How often do you have a hangover? *SHOW CARD 5.* |
| 1 2 3 4 5 6 7 | Every day  Several times a week  About once a week  Several times a month  About once a month  Less than once a month  Never or almost never |
| 97  98 | Difficult to answer  Refuse to answer |
|  |  |
| G21. | How often do you fail to fulfil your family or personal obligations due to drinking alcohol? *SHOW CARD 5.* |
| 1 2 3 4 5 6 7 | Every day  Several times a week  About once a week  Several times a month  About once a month  Less than once a month  Never |
| 97  98 | Difficult to answer  Refuse to answer |
|  |  |
| G22. | Do you ever go to sleep at night with your clothes on because of being drunk? *SHOW CARD 5.* |
| 1 2 3 4 5 6 7 | Every day  Several times a week  About once a week  Several times a month  About once a month  Less than once a month  Never or almost never |
| 97  98 | Difficult to answer  Refuse to answer |
| G23.  1  2  97  98 | **Have you had episodes of zapoi in the past year?**  Yes  No *Go to G37*  Difficult to answer *Go to G37*  Refuse to answer *Go to G37* |
|  | |
|  |  |
|  |  |
| **G24.** | **How many episodes did you have last year?** |
| 1 | 1 |
| 2 | 2-4 |
| 3 | 5-9 |
| 4 | 10 or more |
| 97  98 | Difficult to answer *Go to G37*  Refuse to answer *Go to G37* |
| **G25.** | Have you had one or more episodes of zapoi in the past **month**? |
| 1 2 | Yes  No |
| 97  98 | Difficult to answer  Refuse to answer |
| **G26.** | **How long does a typical episode last?** |
| 1 2 3  4 | 2-3 days  4-5 days  6-9 days  10 and more days |
| 97  98 | Difficult to answer  Refuse to answer |
|  |  |
| **GN26a** | **When have zapois started?** |
| 1 | 6 months ago or less |
| 2 | 7-12 months ago |
| 3 | 1-3 years ago |
| 4 | 4-5 years ago |
| 5 | 6-9 years ago |
| 6 | 10 years ago or more |
| 97  98 | Difficult to answer  Refuse to answer |

| G37. In the last 12 months, have you had any of the following experiences? | | Yes | No | Difficult to answer | Refuse to answer |
| --- | --- | --- | --- | --- | --- |
| a | Have you ever felt you should cut down on your drinking? | 1 | 2 | 97 | 98 |
| b | Have people ever annoyed you by criticising your drinking? | 1 | 2 | 97 | 98 |
| c | Have you ever felt bad or guilty about your drinking? | 1 | 2 | 97 | 98 |
| d | Have you ever had a drink first thing in the morning to steady your nerves or get rid of a hangover? | 1 | 2 | 97 | 98 |

|  | Last problem drinking episode.  Now I would like to talk to you about the last (before this hospitalization) epizode of problem drinking. Problem drinking – is drinking which leads to negative consequences. For example, problem with health, family, police or at work. |
| --- | --- |
|  |  |
| **GN38.** | **Was this zapoi?** |
|  | **By ‘zapoi’, I mean a period of continuous drunkenness of two or more days during which the person does not work and is withdrawn from normal life.** |
| 1 | Yes *Go to GN49* |
| 2 | No |
| 97  98 | Difficult to answer  Refuse to answer |
|  |  |
| **GN39.** | When did this last heavy drinking episode started? |
|  | Date: __ __ / __ __ / __ __ __ __ |
| 97  98 | Difficult to answer *Go to GN40*  Refuse to answer |
| GN40. | How many:  Days ago______Weeks ago_____Months ago_______Years ago______ |
| 97  98 | Difficult to answer  Refuse to answer |
| GN41. | How long did this drinking episode last?  _____________Days_________Weeks_________Months_______Years |
| 97  98 | Difficult to answer  Refuse to answer |
|  |  |
|  | Now I would ask you about alcohol beverages which you drank during these last episode. |
| GN42. | **During this drinking episode did you drink beer?** |
| 1  2  97  98 | Yes  No *Go to GN43*  Difficult to answer  Refuse to answer |
| GN42a. | **When you drank beer, how much did you drink on average per day?** (Bottles (0,5l)________ |
| 97  98 | Difficult to answer  Refuse to answer |
| GN42b. | **When you drank beer, what was the maximum amount per day?** (Bottles (0,5l)___________ |
| 97  98 | Difficult to answer  Refuse to answer |
|  |  |
|  |  |
|  |  |
| GN43. | **During this drinking episode did you drink wine?** |
| 1 | Yes |
| 2 | No *Go to GN44* |
| 97  98 | Difficult to answer  Refuse to answer |
| GN43a. | **When you drank wine, how much did you drink on average per day?** (Grams)________________ |
| 97  98 | Difficult to answer  Refuse to answer |
| GN43b. | **When you drank wine, what was the maximum amount per day?** (Grams)____________ |
| 97  98 | Difficult to answer  Refuse to answer |
|  |  |
| GN44. | **During this drinking episode did you drink fortified wine?** |
| 1  2  97  98 | Yes  No *Go to GN45*  Difficult to answer  Refuse to answer |
| GN44a. | **When you drank fortified wine, how much did you drink on average per day?** (Grams)____________ |
| 97  98 | Difficult to answer  Refuse to answer |
| GN44b. | **When you drank fortified wine, what was the maximum amount per day?** (Grams)____________ |
| 97  98 | Difficult to answer  Refuse to answer |
|  |  |
| GN45. | **During this drinking episode did you drink spirits (vodka, cognak, whisky)?** |
| 1  2  97  98 | Yes  No *Go to GN46*  Difficult to answer  Refuse to answer |
| GN45a. | **When you drank spirits, how much did you drink on average per day?** (Grams)________________ |
| 97  98 | Difficult to answer  Refuse to answer |
| GN45b. | **When you drank spirits, what was the maximum amount per day?** (Grams)____________ |
| 97  98 | Difficult to answer  Refuse to answer |
|  |  |
|  |  |
|  |  |
| GN46. | **During this drinking episode did you drink spirt (syrgical/industrial or other)?** |
| 1  2  97  98 | Yes  No *Go to GN47*  Difficult to answer  Refuse to answer |
| GN46a. | **When you drank spirt (syrgical/industrial or other), how much did you drink on average per day?** (Grams)________________ |
| 97  98 | Difficult to answer  Refuse to answer |
|  |  |
| GN46b. | **When you drank spirt(syrgical/industrial or other), what was the maximum amount per day?** (Grams)____________ |
| 97  98 | Difficult to answer  Refuse to answer |
|  |  |
| GN47. | **During this drinking episode did you drink alcohol cocktails (gin and tonic, etc.)?** |
| 1  2  97  98 | Yes  No *Go to GN48*  Difficult to answer  Refuse to answer |
| GN47a. | **When you drank alcohol cocktails, how much did you drink on average per day?** (Grams)________________ |
| 97  98 | Difficult to answer  Refuse to answer |
|  |  |
| GN47b. | **When you drank alcohol cocktails, what was the maximum amount per day?** (Grams)____________ |
| 97  98 | Difficult to answer  Refuse to answer |
|  |  |
| GN48. | **During this drinking episode did you drink ethanol containing substances ("Hawthorne", "Pepper", "Clear light", "Junioper", "Troynoy cologne", "Troyar")?** |
| 1  2  97  98 | Yes  No *Go to GN49*  Difficult to answer  Refuse to answer |
|  |  |
| GN48a. | **When you drank ethanol containing substances, how much did you drink on average per day?** (Grams)________________ |
| 97  98 | Difficult to answer  Refuse to answer |
|  |  |
| GN48b.  97  98 | **When you drank ethanol containing substances, what was the maximum amount per day?** (Grams)____________  Difficult to answer  Refuse to answer |
|  |  |

|  | **Last drinking episode. Zapoi.** | |
| --- | --- | --- |
| GN49. | **When did this last heavy drinking episode started?**  **Date:** __ __ / __ __ / __ __ __ __ | |
|  | | 97 Difficult to answer *Go to GN50*  98 Refuse answer |
| GN50. | | **How many:** Days ago______Weeks ago_____Months ago_______Years ago______ |
| 97  98 | | Difficult to answer  Refuse to answer |
|  | |  |
| GN51. | | **How long did this zapoi last?**  Days ago______Weeks ago_____Months ago_______Years ago______ |
| 97  98 | | Difficult to answer  Refuse to answer |
|  | | **Now I would ask you about alcohol beverages which you drank during these last episode.** |
|  | |  |
| GN52. | | **During this episode of zapoi did you drink beer?** |
| 1  2  97  98 | | Yes  No *Go to GN53*  Difficult to answer  Refuse to answer |
| GN52a. | | **When you drank beer, how much did you drink on average per day**? (Bottles (0,5l)________________ |
| 97  98 | | Difficult to answer  Refuse to answer |
| GN42b. | | **When you drank wine, what was the maximum amount per day?** (Bottles (0,5l)____________ |
| 97  98 | | Difficult to answer  Refuse to answer |
|  | |  |
| GN53. | | **During this episode of zapoi did you drink wine?** |
| 1  2  97  98 | | Yes  No *Go to GN54*  Difficult to answer  Refuse to answer |
| GN53a. | | **When you drank wine, how much did you drink on average per day?** (Grams)________________ |
| 97  98 | | Difficult to answer  Refuse to answer |
| GN53b. | | **When you drank wine, what was the maximum amount per day?** (Grams)____________ |
| 97  98 | | Difficult to answer  Refuse to answer |
|  | |  |
| GN54. | | **During this episode of zapoi did you drink fortified wine?** |
| 1  2  97  98 | | Yes  No *Go to GN55*  Difficult to answer  Refuse to answer |
| GN54a. | | **When you drank fortified wine, how much did you drink on average per day?** (Grams)____________ |
| 97  98 | | Difficult to answer  Refuse to answer |
| GN54b. | | **When you drank fortified wine, what was the maximum amount per day?** (Grams)____________ |
| 97  98 | | Difficult to answer  Refuse to answer |
|  | |  |
| GN55. | | **During this episode of zapoi did you drink spirits (vodka, cognak, whisky)?** |
| 1  2  97  98 | | Yes  No *Go to GN56*  Difficult to answer  Refuse to answer |
| GN55a. | | **When you drank spirits, how much did you drink on average per day?** (Grams)________________ |
| 97  98 | | Difficult to answer  Refuse to answer |
| GN55b. | | **When you drank spirits, what was the maximum amount per day?** (Grams)____________ |
| 97  98 | | Difficult to answer  Refuse to answer |
|  | |  |
| GN56. | | **During this episode of zapoi did you drink spirit (syrgical/industrial or other)?** |
| 1  2  97  98 | | Yes  No *Go to GN57*  Difficult to answer  Refuse to answer |
| GN56a. | | **When you drank spirit (syrgical/industrial or other), how much did you drink on average per day?** (Grams)________________ |
| 97  98 | | Difficult to answer  Refuse to answer |
|  | |  |
| GN56b. | | **When you drank spirt, what was the maximum amount per day?** (Grams)____________ |
| 97  98 | | Difficult to answer  Refuse to answer |
|  | |  |
|  | |  |
|  | |  |
|  | |  |
|  | |  |
| GN57. | | **During this episode of zapoi did you drink alcohol cocktails (gin and tonic, etc.)?** |
| 1  2  97  98 | | Yes  No *Go to G27*  Difficult to answer  Refuse to answer |
|  | |  |
| GN57a. | | **When you drank alcohol cocktails, how much did you drink on average per day?** (Grams)____________ |
| 97  98 | | Difficult to answer  Refuse to answer |
|  | |  |
| GN57b. | | **When you drank alcohol cocktails, what was the maximum amount per day?** (Grams)____________ |
| 97  98 | | Difficult to answer  Refuse to answer |
| G27. | | During your most recent episode of zapoi, did you drink ethanol-containing substances? Here we mean any alcoholic substances other than those intended for drinking such as eau de colognes, medicinal tinctures or lotions? |
| 1 2 | | Yes *Go to GN58*  No *Go to GN58* |
| 97  98 | | Difficult to answer *Go GN58*  Refuse to answer *Go to GN58* |
|  | |  |
| G27a. | | **When you drank ethanol containing substances, how much did you drink on average per day?** (Grams)_______ |
| 97  98 | | Difficult to answer  Refuse to answer |
|  | |  |
| G27b.  97  98 | | **When you drank ethanol containing substances, what was the maximum amount per day?** (Grams)____________  Difficult to answer  Refuse to answer |

|  | Narcological help. | |
| --- | --- | --- |
|  |  | |
| GN58. | Why you decided to seek narcological help/were admitted to narcology? *Please choose all that apply.* | |
| 1 | Could not stop drinking myself | |
| 2 | Could not continue drinking anymore because of health reasons/ Felt too ill to drink | |
| 3 | Had a delirum | |
| 4 | Relatives insisted/ Pressure from or influence of my family or friends | |
| 5 | Employer insisted/referred | |
| 6 | I was afraid of losing my job | |
| 7 | I wanted to receive coding and stay abstinent for some time | |
| 8 | I was scared that I could die | |
| 9 | I came for coding | |
| 10 | There were financial reasons | |
| 11 | I want to receive detoxification and “clean my body” | |
| 97  98 | Difficult to answer  Refuse to answer | |
|  | Now I will ask you about all types of treatment, which you could have, that aimed to reduce or cease your alcohol consumption. | |
| **GN59.** | **Have you ever before had help or advice from a doctor, narcologist, social worker or some other professional for an alcohol problem excluding this treatment episode?** | |
| 1 2 | Yes  No Go to GN69 | |
| 97  98 | Difficult to answer  Refuse to answer | |
|  |  | |
| **GN60.** | | **Have you ever received help in narcological dispansery, medical help?** |
| 1  2  97  98 | | Yes  No Go to GN61  Difficult to answer  Refuse to answer |
|  | |  |
| **GN60a.** | | **How many times you have received such help?** Number of times_____________ |
| 97  98 | | Difficult to answer  Refuse to answer |
|  | |  |
| **GN61.** | | **Have you ever received narcological help in private clinic or at home (medical help such as IV)?** |
| 1  2 | | Yes  No Go to GN62 |
| 97  98 | | Difficult to answer  Refuse to answer Go to GN62 |
|  | |  |
| **GN61a.** | | **How many times you have received such help?** Number of times____________ |
| 97  98 | | Difficult to answer  Refuse to answer |
|  | |  |
|  | |  |
| **GN62.** | | **Have you ever received coding (e.g. with hypnosis, injections)?** |
| 1  2 | | Yes  No Go to GN63 |
| 97  98 | | Difficult to answer  Refuse to answer Go to GN63 |
|  | |  |
| **GN62a.** | | **How many times you have received coding?** Number of times____________ |
| 97  98 | | Difficult to answer  Refuse to answer |
|  | |  |
| **GN63.** | | **Have you ever received help from psychologist?** |
| 1  2 | | Yes  No Go to GN64 |
| 97  98 | | Difficult to answer  Refuse to answer Go to GN64 |
|  | |  |
| **GN63a.** | | **How many times you have received such help?** Number of times____________ |
| 97  98 | | Difficult to answer  Refuse to answer |
|  | |  |
| **GN64.** | | **Have you ever received psychotherapy in relation to alcohol use?** |
| 1  2 | | Yes  No Go to GN65 |
| 97  98 | | Difficult to answer  Refuse to answer Go to GN65 |
|  | |  |
| **GN64a.** | | **How many times you have received such help?** Number of times____________ |
| 97  98 | | Difficult to answer  Refuse to answer |
|  | |  |
| **GN65.** | | **Have you ever received behavioral therapy in relation to alcohol use?** |
| 1  2 | | Yes  No Go to GN66 |
| 97  98 | | Difficult to answer  Refuse to answer Go to GN66 |
|  | |  |
| **GN65a.** | | **How many times you have received such help?** Number of times____________ |
| 97  98 | | Difficult to answer  Refuse to answer |
|  | |  |
| **GN66.** | | **Have you ever received group therapy (12 steps, Alcoholics Anonymous) in relation to alcohol use?** |
| 1  2 | | Yes  No Go to GN67 |
| 97  98 | | difficult to answer  refuse to answer Go to GN67 |
|  | |  |
|  | |  |
|  | |  |
| **GN66a.** | | **How many times you have received such help?** Number of times____________ |
| 97  98 | | Difficult to answer  Refuse to answer |
| **GN67.** | | **Have you ever received help from a traditional healer?** |
| 1  2 | | Yes  No Go to GN68 |
| 97  98 | | Difficult to answer  Refuse to answer Go to GN68 |
|  | |  |
| **GN67a.** | | **How many times you have received such help?** Number of times____________ |
| 97  98 | | Difficult to answer  Refuse to answer |
|  | |  |
| **GN68.** | | **Did you get such help or advice in the past 12 months (prior to this hospitalization)?** |
| 1  2 | | Yes  No |
| 97  98 | | Difficult to answer  Refuse to answer |
|  | |  |

|  | | **Non-beverage alcohol use.** |
| --- | --- | --- |
|  | |  |
| GN69. | Have you ever drank ethanol-containing substances not intended for drinking? Here we mean eau de colognes, medicinal tinctures, spirit, lotions (e.g.Hawthorn, Pepper, Clear light, Juniper, Troynoj eau de cologne). | |
| 1 2 | Yes  No *Go to D1* | |
| 97  98 | Difficult to answer  Refuse to answer | |
|  |  | |
| GN70. | When did you start consuming ethanol-containing substances? | |
| 1  2  3  4 | Within the past month  Within past 6 months  Within the last year  More than a year ago | |
| 97  98 | Difficult to answer  Refuse to answer | |
|  |  | |
| GN71. | What ethanol-containing substances do you drink? *Please choose all that apply.* | |
| 1  2  3  4  5  6  7  8  9  10  10a | Infusion of hawthorn  Troyar  Spirits (technical, medical or other)  Composition  Troynoy cologne  “Pepper” lotion  “Juniper” lotion  “Clear light” lotion Windows cleaning liquid, other cleaners  Other types of liquids containing spirits  Other. Specify .................... ................... | |
| 97  98 | Difficult to answer  Refuse to answer | |
|  |  | |
| **GN72.** | **When you consume ethanol-containing substances including spirit, do you dilute them with water or other non-alcoholic beverage (e.g. with juice, coca-cola)?** | |
| 1  2  3  4  5 | Never  Rarely  Sometimes  Most of the time  Always | |
| 97  98 | Difficult to answer  Refuse to answer | |
|  |  | |
|  |  | |
|  |  | |
|  |  | |
| **GN73.** | **Where do you purchase ethanol-containing substances including spirit?** *Please choose all that apply.* | |
| 1  2  3  4  5 | In pharmacies  In kiosks  In stores  At work  Neighbours sell it (sold in the neighbourhood) | |
| 6  7  7a | On a street  Other  Other Specify | |
| 97  98 | Difficult to answer  Refuse to answer | |

|  | **Module D** | | | **USE OF HEALTH CARE SERVICES** | | | | |
| --- | --- | --- | --- | --- | --- | --- | --- | --- |
| D1. | | **This module contains questions about use of medical services, presence of some diseases and use of medications.** **How many times you used the following sources of health care in the last 12 months? (Please check number of times for each type of doctor).** Type of doctor \| Number of times visited in last 12 months \| \| \| \| \|; 1.District physician \| 0 \| 1 \| 2 \| 3 \| 4 \| 5+; 2. Polyclinic cardiologist \| 0 \| 1 \| 2 \| 3 \| 4 \| 5+; 3. Other polyclinic specialist \| 0 \| 1 \| 2 \| 3 \| 4 \| 5+; 4. Hospital cardiologist \| 0 \| 1 \| 2 \| 3 \| 4 \| 5+; 5. Other hospital doctor \| 0 \| 1 \| 2 \| 3 \| 4 \| 5+ | | | | | | |
|  | |  | | | | | | |
| **D2.** | | **In the last 12 months, how many times have you been hospitalised (stayed in the hospital overnight)?**  Number of times.................................... *If “0” Go to D4*  97 Difficult to answer  98 Refuse to answer | | | | | | |
| **D3.** | | **For how many nights did you stay in hospital for each of those hospitalizations?** | | | | | | |
|  | | Hospitalization | | | | Number of days/nights | | |
| 1  2  3  4  5 | |  | | | | .....................................  .....................................  .....................................  .....................................  ..................................... | | |
| 6  7  8  9  10 | |  | | | | .....................................  .....................................  .....................................  .....................................  ..................................... | | |
| 97 | | Difficult to answer | | | |  | | |
| 98 | | Refuse to answer | | | | | | |
| **D4.** | | **How many times have you called or someone called for you an ambulance in the last 12 months?**  Number of times.................. | | | | | | |
|  | |  | | | | | | |
| 97 | | Difficult to answer | | | | | | |
| 98 | | Refuse to answer | | | | | | |
| **D10.** | | **Have you ever been told by a doctor (been diagnosed) that you have:** | | | | | | |
|  | |  | | | | | | |
|  | |  | Yes | | No | | Difficult to answer | Refuse to answer |
| 1 | | Arterial hypertension (high blood pressure) For WOMEN: except during pregnancy | 1 | | 2 | | 97 | 98 |
| 1a | | Arterial hypertension during pregnancy (*women only*) | 1 | | 2 | | 97 | 98 |
| 2 | | High cholesterol level | 1 | | 2 | | 97 | 98 |
| 3 | | Myocardial Infarction/Heart attack | 1 | | 2 | | 97 | 98 |
| 4 | | Heart failure | 1 | | 2 | | 97 | 98 |
| 5 | | Atrial fibrillation | 1 | | 2 | | 97 | 98 |
| 6 | | Angina | 1 | | 2 | | 97 | 98 |
| 7 | | Stroke | 1 | | 2 | | 97 | 98 |
| 8 | | Diabetes | 1 | | 2 | | 97 | 98 |
| 9 | | Kidney disease | 1 | | 2 | | 97 | 98 |
| 10 | | Chronic bronchitis/COPD | 1 | | 2 | | 97 | 98 |
| 11 | | Cancer | 1 | | 2 | | 97 | 98 |
| 12 | | Asthma | 1 | | 2 | | 97 | 98 |
| 13 | | Rheumatoid arthritis | 1 | | 2 | | 97 | 98 |
| 14 | | Osteoarthritis (osteoarthritis) | 1 | | 2 | | 97 | 98 |
| 15 | | Migraine | 1 | | 2 | | 97 | 98 |
| 16 | | Depression | 1 | | 2 | | 97 | 98 |
| 17 | | Anxiety | 1 | | 2 | | 97 | 98 |
|  | | *Note Participants who report they have hypertension should be asked questions D11-D15. Participants who report they have high cholesterol should be asked questions D16-D21. Participants who do not report hypertension or high cholesterol should go directly to E1*. | | | | | | |
|  | |  | | | | | | |
| **D11.** | | **Were you prescribed medicines by a medical professional to treat your hypertension?** | | | | | | |
| 1  2 | | Yes  No *Go to E1 (unless report high cholesterol, then go to D16)* | | | | | | |
| 97  98 | | Difficult to answer *Go to E1* *(unless report high cholesterol, then go to D16)*  Refuse to answer *Go to E1(unless report high cholesterol, then go to D16)* | | | | | | |
| **D12.** | | **Did you obtain all the medicines you were prescribed by a medical professional?** | | | | | | |
| 1  2 | | Yes *Go to D14*  No | | | | | | |
| 97  98 | | Difficult to answer  Refuse to answer *Go to D14* | | | | | | |
|  | |  | | | | | | |
|  | |  | | | | | | |
| **D13.** | | **If not, what was the reason for not obtaining all prescribed medicines?** *Please choose all that apply.* | | | | | | |
| 1 | | The prescribed medicines were not available at the pharmacy | | | | | | |
| 2 | | The prescribed medicines are too expensive | | | | | | |
| 3 | | The prescribed medicines are ineffective | | | | | | |
| 4 | | The prescribed medicines have harmful side effects | | | | | | |
| 5 | | I prefer to treat my high blood pressure with other pharmaceuticals that are recommended by friends/relatives | | | | | | |
| 6 | | I prefer to treat my high blood pressure with other pharmaceuticals that are recommended by the pharmacist | | | | | | |
| 7 | | I prefer to treat my high blood pressure with alternative (non-pharmaceutical) medicines | | | | | | |
| 8  8a | | Other  Other. Specify .................... .................... | | | | | | |
| 97  98 | | Difficult to answer  Refuse to answer | | | | | | |
|  | |  | | | | | | |
| **D14.** | | **Do you take your prescribed blood pressure medication every day?** | | | | | | |
| 1  2  3 | | Yes *Go to D16*  No  Did not obtain any medication | | | | | | |
| 97  98 | | Difficult to answer  Refuse to answer | | | | | | |
|  | |  | | | | | | |
| **D15.** | | **If not, what was the reason?** *Please choose all that apply.* | | | | | | |
| 1 | | I do not have high blood pressure any more | | | | | | |
| 2 | | I did not know it is necessary to take the medication every day | | | | | | |
| 3 | | My doctor told me to take it only when I have symptoms | | | | | | |
| 4 | | I cannot afford to take it every day | | | | | | |
| 5 | | I forget to take it every day | | | | | | |
| 6 | | I stop taking it when it makes me feel worse | | | | | | |
| 7 | | I stop taking it when I feel better | | | | | | |
| 8 | | Taking this medication every day would have harmful side effects | | | | | | |
| 9 | | I prefer to use medication that I purchased without a prescription | | | | | | |
| 10  10a | | Other  Other. Specify .................... .................... | | | | | | |
| 97  98 | | Difficult to answer  Refuse to answer | | | | | | |
| **D16.** | | **Were you prescribed medicines by a medical professional to treat your cholesterol?** | | | | | | |
| 1  2 | | Yes  No *Go to E1* | | | | | | |
| 97  98 | | Difficult to answer *Go to E1*  Refuse to answer *Go to E1* | | | | | | |
| **D17.** | | **Did you obtain all the medicines you were prescribed by a medical professional?** | | | | | | |
| 1  2 | | Yes *Go to D19*  No | | | | | | |
| 97  98 | | Difficult to answer  Refuse to answer *Go to D21* | | | | | | |
| **D18.** | | **If not, what was the reason for not obtaining all prescribed medicines?** *Please choose all that apply.* | | | | | | |
| 1 | | The prescribed medicines were not available at the pharmacy | | | | | | |
| 2 | | The prescribed medicines are too expensive | | | | | | |
| 3 | | The prescribed medicines are ineffective | | | | | | |
| 4 | | The prescribed medicines have harmful side effects | | | | | | |
| 5 | | I prefer to treat my high cholesterol with other pharmaceuticals that are recommended by friends/relatives | | | | | | |
| 6 | | I prefer to treat my high cholesterol with other pharmaceuticals that are recommended by the pharmacist | | | | | | |
| 7 | | I prefer to treat my high cholesterol with alternative (non-pharmaceutical) medicines | | | | | | |
| 8  8a | | Other  Other. Specify .................... .................... | | | | | | |
| 97  98 | | Difficult to answer  Refuse to answer | | | | | | |
|  | |  | | | | | | |
| **D19.** | | **Do you take your prescribed cholesterol medication every day?** | | | | | | |
| 1  2  3 | | Yes *Go to E1*  No  Did not obtain any medication | | | | | | |
| 97  98 | | Difficult to answer  Refuse to answer | | | | | | |
| **D20.** | | **If not, what was the reason?** *Please choose all that apply.* | | | | | | |
| 1  2  3  4  5  6  7  8  9  10  10a | | I do not have high cholesterol any more  I did not know it is necessary to take the medication every day  My doctor told me to take it only when I have symptoms  I cannot afford to take it every day  I forget to take it every day  I stop taking it when it makes me feel worse  I stop taking it when I feel better  Taking this medication every day would have harmful side effects  I prefer to use medication that I purchased without a prescription  Other  Other. Specify .................... .................... | | | | | | |
| 97  98 | | Difficult to answer  Refuse to answer | | | | | | |
| **D21.** | | **Aside from any prescribed medicines, do you purchase pharmaceutical medicines that are not prescribed by a medical professional (e.g. suggested by the pharmacist or family/friend), to treat your hypertension or high cholesterol?** | | | | | | |
| 1  2 | | Yes  No | | | | | | |
| 97  98 | | Difficult to answer  Refuse to answer | | | | | | |

| **Module E** \| **DEPRESSION AND ANXIETY** **Now I would like to talk to you about your feelings in the past 2 weeks.** **Over the last 2 weeks, how often have you been bothered by any of the following problems?** *SHOW CARD 6.* | | Not at all | Several days | More than half of the days | | Nearly every day |
| --- | --- | --- | --- | --- | --- | --- |
| **E1.** | Little interest or pleasure in doing things | 0 | 1 | 2 | | 3 |
| **E2.** | Feeling down, depressed or hopeless | 0 | 1 | 2 | | 3 |
| **E3.** | Trouble falling or staying asleep, or sleeping too much | 0 | 1 | 2 | | 3 |
| **E4.** | Feeling tired or having little energy | 0 | 1 | 2 | | 3 |
| **E5.** | Poor appetite or overeating | 0 | 1 | 2 | | 3 |
| **E6.** | Feeling bad about yourself- or that you are a failure or have let yourself or your family down | 0 | 1 | 2 | | 3 |
| **E7.** | Trouble concentrating on things, such as reading the newspaper or watching television | 0 | 1 | 2 | | 3 |
| **E8.** | Moving or speaking so slowly that other people have noticed. Or the opposite- being so fidgety or restless that you have been moving around a lot more than usual | 0 | 1 | 2 | | 3 |
| **E9.** | Thoughts that you would be better off dead or of hurting yourself in some way | 0 | 1 | 2 | | 3 |
| **E10.** | How difficult have these problems made it for you to do your work, take care of things at home, or get along with other people? | Not at all difficult | Somewhat difficult | | Very difficult | Extremely difficult |
|  |  | 0 | 1 | 2 | | 3 |

| **Over the last 2 weeks, how often have you been bothered by any of the following problems?** *SHOW CARD 6* | | Not at all | Several days | More than half of the days | Nearly every day |
| --- | --- | --- | --- | --- | --- |
| **E11.** | Feeling nervous, anxious, or on edge | 0 | 1 | 2 | 3 |
| **E12.** | Not being able to sleep or control worrying | 0 | 1 | 2 | 3 |
| **E13.** | Worrying too much about different things | 0 | 1 | 2 | 3 |
| **E14.** | Trouble relaxing | 0 | 1 | 2 | 3 |
| **E15.** | Being so restless that it is hard to sit still | 0 | 1 | 2 | 3 |
| **E16.** | Becoming easily annoyed or irritable | 0 | 1 | 2 | 3 |
| **E17.** | Feeling afraid, as if something awful might happen | 0 | 1 | 2 | 3 |
| **E18.** | How difficult have these problems made it for you to do your work, take care of things at home, or get along with other people? | Not at all difficult | Somewhat difficult | Very difficult | Extremely difficult |
|  |  | 0 | 1 | 2 | 3 |

| **Module H** | **SMOKING** |
| --- | --- |

I will now ask you some questions concerning your smoking habits.

| H1. | Are you a current smoker? *Please choose the single most appropriate answer.* |
| --- | --- |
| 1 2 3 | Never a smoker *Go to H8a*  No, ex-smoker *Go to H2*  Yes, a current-smoker *Go to H3* |
| 97  98 | Difficult to answer *Go to H3*  Refuse to answer *Go to H8a* |
| H2. | How many years ago did you stop smoking regularly? *Please choose the single most appropriate answer.* |
| 1 2 3 4 | Up to 1 year ago  More than 1, up to 5 years ago  More than 5, up to 10 years ago  More than 10 years ago |
| 97  98 | Difficult to answer  Refuse to answer |
| H3. | What do/did you smoke most often? *Please choose the single most appropriate answer.* |
| 1 2 3 4  4a | Papyrosi  Filtered cigarettes  Unfiltered cigarettes  Other  Other. Specify .................... .................... |
| 97  98 | Difficult to answer  Refuse to answer |
| H4. | When you smoke/smoked, how many per day is/was usual? *Please choose the single most appropriate answer.* |
| 1 2 3 | Up to 10  More than 10, up to 20  More than 20 |
| 97  98 | Difficult to answer  Refuse to answer |
| H5. | How old were you when you started smoking regularly?________Years |
| 97  98 | Difficult to answer  Refuse to answer |
| H6. | Have you ever been advised by medical proffesional (your district doctor, cardiologist, any other physician) to stop smoking? |
| 1  2 | Yes  No *Go to H8a* |
| 97  98 | Difficult to answer  Refuse to answer |
| **H7.** | **Was any assistance offered?** |
| 1  2 | Yes  No *Go to H8a* |
| 97  98 | Difficult to answer  Refuse to answer |
|  |  |
| **H8.** | **What kind of assistance was offered? Please choose all that apply** |
| 1 | provision of nicotine replacement therapy |
| 2 | referral to the behavioral therapy |
| 3 | 12 steps programmes |
| 4 | Coding |
| 5 | Material motivation |
| 6  6a | Other  Other. Specify .................... .................... |
| 97  98 | Difficult to answer  Refuse to answer |
|  |  |
| **H8a** | **Has frequency of your smoking changed after this admission?** |
| 1 | No |
| 2  3 | Yes, increased  Yes, decreased |
| 4  5 | Yes, I stopped smoking  Yes, I started smoking |
| 97  98 | Difficult to answer  Refuse to answer |

**K2.** **Did someone physically assault you in the past year?**

| **Module K** \| **PSYCHOSOCIAL FACTORS** **We have reached the final stage of our questionnaire. To begin with I would like to ask you some questions about events of the past 6 months in your and your relatives lives.** | | | | |
| --- | --- | --- | --- | --- |
| **K1. Have any of the following life events or problems happened to you during the last 6 months?** | No | Yes | Difficult to answer | Refuse to answer |
| **You yourself suffered serious illness, injury or an assault** | 2 | 1 | 97 | 98 |
| **A serious illness, injury or assault happened to a close relative** | 2 | 1 | 97 | 98 |
| **Your parent, child or partner died** | 2 | 1 | 97 | 98 |
| **A close family friend or another relative (aunt, cousin, grandparent) died** | 2 | 1 | 97 | 98 |
| **You had a separation due to marital difficulties** | 2 | 1 | 97 | 98 |
| **You broke off a steady relationship** | 2 | 1 | 97 | 98 |
| **You had a serious problem with a close friend, neighbour or relative** | 2 | 1 | 97 | 98 |
| **You became unemployed or you were seeking work unsuccessfully for more than one month** | 2 | 1 | 97 | 98 |
| **You were sacked from your job** | 2 | 1 | 97 | 98 |
| **You had a major financial crisis** | 2 | 1 | 97 | 98 |
| **You had problems with the police and a court appearance** | 2 | 1 | 97 | 98 |
| **Something you valued was lost or stolen** | 2 | 1 | 97 | 98 |

| 1  2 | Yes  No |
| --- | --- |
| 97  98 | Difficult to answer  Refuse to answer |

| **Module X** | **CIRCUMSTANCES OF INTERVIEW** |
| --- | --- |

| *The following questions are answered only by you as an interviewer and are not to be read out.* | |
| --- | --- |
| X1. | How would you judge the reliability of the answers from this interview? |
|  |  |
| 1  2    3 | Satisfactory  Not entirely satisfactory. For example, a moderate level of non-response by *the subject*, or perhaps small interruptions affected the quality of the responses  Poor. For example, a high level of non-response by *the subject*, or perhaps many/constant interruptions affected the quality of the responses |
|  |  |
| X2. | Were there any other people present in the same room while the interview was taking place? |
| 1 2 | Yes  No |
|  |  |
| X3. | Were there any interruptions to the interview? |
| 1 2 | Yes  No *Go to X5* |
| X4. | Please provide details of interruptions, including their duration_______ |
| X5. | Any other comments, including indication of questions that were particularly hard to answer________ |
