# Supplementary material for: Know Your Heart: Rationale, design and conduct of a cross-sectional study of cardiovascular structure, function and risk factors in 4500 men and women aged 35-69 years from two Russian cities, 2015-18
Source: Wellcome Open Res. 2018 Dec 3;3:67. Originally published 2018 Jun 4. [Version 3] doi: 10.12688/wellcomeopenres.14619.3 (PMC6073094; doi:10.12688/wellcomeopenres.14619.3)
Supplement: Supplementary file 3 [file wellcomeopenres-3-16294-s0002.tgz › d9a03f66-5fce-45a3-8a2e-d18febd22997_Supplementary_file_3._Questionnaires_and_data_collection_tools.zip!/questionnaires_181121_publish_version2/181118_Baseline_survey_narcology_RU.docx]

|  |  |
| --- | --- |

**Опросник первого этапа для пациентов наркологического профиля, проходящих лечение в связи со злоупотреблением алкоголем**

Международный проект по сердечно-сосудистым заболеваниям в России

2015-2018

Версия публикации 2.0 Ноябрь 2018

В России один из самых высоких уровней сердечно-сосудистых заболеваемости в мире. Целью международного проекта "Сердечно-сосудистые заболевания в России" является понимание причин данного положения. Весомым компонентом данного исследования было исследование От Сердца к Сердцу, направленное на выявление природы и причин сердечно-сосудистой заболеваемости в России. В рамках данного проекта были проведены масштабные перекрёстные опросы в двух городах России, Новосибирске и Архангельске. Ислледование охватило 4542 мужчин и женщин в возрате 35-69 лет, набранных из генеральной популяции в период 2015-2018. Исследование включало два компонента: 1)опрос населения для получения информации по социально-демографическим характеристикам и сердечно-сосудистым факторам риска; в большинстве случаев опрос проводился на дому. 2)комплексное медицинское обследование в клинике, включающее детальное изучение сердечно-сосудистой системы.

Международный проект "Сердечно-сосудистые заболевания в России" был частично финансирован Wellcome Trust Strategic Award [100217]. Проект также был финансирован Арктическим Университетом Норвегии, Университет Тромсё, Норвежским Институтом Общественного Здоровья и Министерством Здравоохранения и Социального Обеспечения Норвегии.

Разработка опросников велась под руководством Лондонской Школы Гигиены и Тропической Медицины совместно с Северным государственным медицинским университетом, г.Архангельск, Новосибирским государственным медицинским университетом, Научно-исследовательским институтом терапии и профилактической медицины-филиала «ФИЦ ИЦиГ» СО РАН, г. Новосибирск и коллегами других институтов.

Данная бумажная версия опросника была использована для проведения интервью первого этапа среди подгруппы пациентов, проходящих лечение в связи с злоупотреблением алкоголем в наркологическом отделении. Интервью проводилось электронно с использованием Computer Assisted Personal Interviewing CAPI устройства.

Подробные детали исследования опубликованы в Know Your Heart: Rationale, design and conduct of a cross-sectional study of cardiovascular structure, function and risk factors in 4500 men and women aged 35-69 years from two Russian cities, 2015-18
Cook S, Malyutina S, Kudryavtsev AV, Averina M, Bobrova N, Boytsov S, Brage S, Clark T et al.

Содержание

[Модуль A. Социально-демографические факторы	4](#_Toc512505986)

Модуль G. Употребление алкоголя	9

[Модуль D. Обращение за медицинской помощью.	27](#_Toc512505994)

[Модуль E. Депрессия и Тревога.	31](#_Toc512505995)

[Модуль H. Курение	33](#_Toc512505997)

[Модуль K. Психосоциальные факторы	35](#_Toc512506001)

[Модуль X. Обстоятельства интервью	36](#_Toc512506003)

| Дата Интервью__ __ / __ __ / __ __ __ __ ID Интервьюера___ ID Респондента________ | |
| --- | --- |
| **Модуль A** | **СОЦИАЛЬНО-ДЕМОГРАФИЧЕСКИЕ ФАКТОРЫ** |

**Для начала позвольте задать несколько вопросов о Вас.**

| A1. | Сколько Вам лет?_________Лет |
| --- | --- |
|  | |
| 97  98 | Затрудняюсь ответить  Отказ от ответа |
| A2. | Назовите дату Вашего рождения? __ __ / __ __ / __ __ __ __ |
|  | |
| 97  98 | затрудняюсь ответить  отказ от ответа |
| **A3.** | Интервьюер! Отметьте пол респондента. |
| 1  2 | Мужской  Женский |
|  |  |
| A4. | Кто Вы по национальности? *Возможен только один вариант ответа.* |
| 1 2  3  4  5  5a | Русский  Украинец  Ненец  Белорус  Другое.  Другое. Уточните .................... .................... |
| 97  98 | Затрудняюсь ответить  Отказ от ответа |
|  |  |
| A5. | Где Вы родились? *Возможен только один вариант ответа.* |
| 1 2 3 4 5 | В Архангельске  В Архангельской области  В другом регионе России  В регионе бывшего Советского Союза за пределами России  За пределами бывшего Советского Союза |
| 97  98 | Затрудняюсь ответить  Отказ от ответа |
| A6. | Как давно Вы постоянно проживаете в Архангельске? *Возможен только один вариант ответа.* |
| 1  2  3  4  5 | Не более 12 месяцев  От 1 года до 5 лет  От 5 лет до 10 лет  Более 10 лет, но не всю жизнь  С рождения (не считая периоды службы в армии, другие периоды временного отсутствия) |
| 97  98 | Затрудняюсь ответить  Отказ от ответа |
|  |  |
| **I4_a** | **До данной госпитализации где Вы проживали?** |
| 1a  1 2 3 4 5 6  6a | Не было определенного места жительства  Общежитие  Комната (комнаты) в коммунальной квартире  Отдельная квартира  Часть дома  Отдельный дом  Другое  Другое Уточните .................... .................... |
| 97  98 | Затрудняюсь ответить  Отказ от ответа |
| A7. | Каково Ваше семейное положение в настоящее время? Вы *Возможен только один вариант ответа*. |
| 1 2 3 4 5 | Проживаете в зарегистрированном браке  Проживаете в незарегистрированном браке  Разведены или живете раздельно с женой/мужем  В довец/вдова  Не женаты/не замужем и никогда не были |
| 97  98 | Затрудняюсь ответить  Отказ от ответа |
| A8. | Сколько у Вас детей? *Возможен только один вариант ответа.* |
| 1  2  3  4  5 | 0  1  2  3  4 или больше |
| 97  98 | Затрудняюсь ответить  Отказ от ответа  I wo |

Сейчас я хочу спросить о Вашем образовании и работе.

| A9. | | **Каков уровень Вашего образования?** *Покажите карточку 1. Возможен только один вариант ответа.* | | |  |
| --- | --- | --- | --- | --- | --- |
| 1 2 3 4 5  6  7 | | Неполное среднее или ниже  Полное среднее  Начальное профессиональное (ПТУ, училище без среднего образования)  Профессиональное среднее (н-р, ПТУ с полным средним образованием)  Среднее специальное (н-р, медицинское, педагогическое училище, техникум)  Незаконченное высшее  Высшее | | |  |
| 97  98 | | Затрудняюсь ответить  Отказ от ответа | | |  |
| **A10.** | | **Учитесь ли Вы в данный момент (являетесь ли студентом)?** | | |  |
| 1 | | Да | | |  |
| 2 | | Нет | | |  |
| 97 | | Затрудняюсь ответить | | |  |
| 98 | | Отказ от ответа | | |  |
|  | |  | | |  |
|  | |  | | |  |
|  | | | | |  |
| **A11. Являетесь ли Вы…** Только один вариант ответа**.** | | | | |  |
| 1 2  3  97  98  **A12.**  1  2  97  98  **A13**.  **A14.**  1  2  3  4  5  5a  6 | | Пенсионером (кроме пенсионеров по инвалидности)  Пенсионером по инвалидности  Ничего из перечисленного  Затрудняюсь ответить  Отказ от ответа  **Являетесь ли Вы работающим на постоянно оплачиваемой работе?**  Да  Нет *Переход к A14*  Затрудняюсь ответить  Отказ от ответа  **В течение последних 12 месяцев сколько месяцев Вы работали на данной работе?**  Количество месяцев:……………………….. *Переход к A17*  **Являетесь ли Вы…***Вопрос не задаётся тем, кто на постоянной оплачиваемой работе. (A12-* *Нет)*  Работающим временно или от случая к случаю  Безработным, ищущим работу  Неработающим, не ищущим работу  Домохозяйкой  Другое  Другое. Уточните .................... ....................  Ничего из перечисленного | | |  |
| 97  98 | | Затрудняюсь ответить  Отказ от ответа | | |  |
|  | |  | | |  |
| **A15.** | | **Как давно Вы оставили постоянную работу?** *Возможен только один вариант ответа.* | | |  |
| 1 2 3 4 5 6 | | Никогда не было постоянной работы *Переход к A17*  В течение последней недели  От 1 до 4 недель назад  От 1 до 6 месяцев назад  От 6 до 12 месяцев назад  Более года назад | | |  |
| 97  98 | | Затрудняюсь ответить  Отказ от ответа | | |  |
| **A16.** | | **По какой причине Вы оставили постоянную оплачиваемую работу?** *Возможен только один вариант ответа.* | | |  |
| 1 2 3 4 5  6  7  8  9  10  11  11a | Начали учиться  Вышли на пенсию  Не могли найти работу после окончания учебы  Предприятие (организация) закрылось или произошло сокращение  Закончился срок временного трудоустройства  Предложили уйти/Вас уволили  Оставили работу добровольно из-за неудовлетворенности оплатой или условиями труда  всегда работал (-а) на временных работах  Оставили работу по состоянию здоровья  Оставили работу, чтобы сидеть с детьми, ухаживать за другими людьми  Оставили работу по другой причине  Другое  Другое. Уточните .................... .................... | | | |  |
| 97  98 | Затрудняюсь ответить  Отказ от ответа | | | |  |
|  |  | | | |  |
| Я хочу задать несколько вопросов о Вашей основной работе в последний год или в тот год, когда Вы работали. | | | | |  |
|  | |  | | |  |
| **A17.** | | Кем Вы работали в течение этого последнего года, какая была ваша основная оплачиваемая работа? *Интервьюер впечатывает полное название (н-р, учитель начальных классов)*  Ваша оплачиваемая работа:................................................................. | | |  |
|  | | | | |  |
| **A18.**  1  2  3  4  5  6  7  8  9 | | **Что из перечисленного лучше всего описывает Вашу работу?** *Покажите карточку 2. Только один вариант ответа.*  Законодатель, крупный чиновник, руководитель высшего звена  Специалист высшего уровня квалификации  Специалист среднего уровня квалификации  Служащий офисный и по обслуживанию клиентов  Работник сферы торговли и услуг  Квалифицированный рабочий, занятый ручным трудом  Квалифицированный рабочий, использующий машины и механизмы  Неквалифицированный рабочий  Военнослужащий | | |  |
| 10  10a | | Другое  Другое. Уточните .................... .................... | | |  |
| 97  98 | | Затрудняюсь ответить  Отказ от ответа  *Интервьюер! Если возникают противоречия между должностным статусом и образованием респондентов, то выбирайте ответ в соответствии с должностным статусом* | | |  |
| **A19.** | | **Пропускали ли Вы работу по состоянию своего здоровья в последние 12 месяцев?** *(Вопрос только для тех, кто выполнял оплачиваемую работу в последние 12 месяцев)* | | |  |
| 1  2 | | Да  Нет *Переход к A21* | | |  |
| 97  98 | | Затрудняюсь ответить  Отказ от ответа *Переход к A21* | | |  |
| **A20** | | **Сколько дней вы пропустили по состоянию здоровья?** | | |  |
| 1 | | 1-3 дня | |  |  |
| 2 | | 4-5 дней | |  |  |
| 3 | | 6-10 дней | |  |  |
| 4 | | 11+ дней | |  |  |
| 97 | | Затрудняюсь ответить | |  |  |
| 98 | | Отказ от ответа | | |  |
|  | |  | | |  |
| A21 | | Есть ли у Вас в данный момент официально установленная инвалидность? | | |  |
| 1  2 | | Да  Нет *Переход к I9* | | |  |
| 97  98 | | Затрудняюсь ответить *Переход к I9*  Отказ от ответа *Переход к I9* | | |  |
|  | |  | | |  |
|  | |  | | |  |
|  | |  | | |  |
|  | |  | | |  |
| A22. | | Когда она была установлена? | | |  |
| 1 | | Не более 6 месяцев назад | | |  |
| 2  3 | | От 6 до 12 месяцев назад  От 1 до 5 лет назад | | |  |
| 4 | | От 5 до 10 лет назад | | |  |
| 5  6  97  98 | | Более 10 лет назад, но не с рождения  С самого рождения  Затрудняюсь ответить  Отказ от ответа | | |  |
| A23. | | Какая у Вас в данный момент группа инвалидности? | | |  |
| 1 2 3 | | 1-я  2-я  3-я | | |  |
| 97  98 | | Затрудняюсь ответить  Отказ от ответа | | |  |
| I9. | | | Выберите высказывание, которое наиболее точно описывает финансовые возможности семьи в течение последнего года. *Покажите карточку 3.* | | |
| 1  2  3  4  5  6 | | | Денег не хватает даже на продукты, "едва сводим концы с концами"  На продукты денег хватает, но покупка одежды вызывает затруднения  Денег хватает на продукты и одежду, но покупка крупной бытовой техники затруднительна  Можем без труда приобретать крупную бытовую технику, но покупка нового легкового автомобиля затруднительна  Хватает доходов на новый легковой автомобиль, однако покупка квартиры или дома (иной недвижимости) является для нас затруднительной  Материальных затруднений не испытываем; при необходимости можем купить квартиру, дом (иную недвижимость) | | |
| 97  98 | | | Затрудняюсь ответить  Отказ от ответа | | |

| **Модуль G** | **УПОТРЕБЛЕНИЕ АЛКОГОЛЯ** |
| --- | --- |

**Теперь я задам Вам вопросы о Вашем употреблении алкоголя в течение последних 12 месяцев. Мы задаем эти вопросы всем, даже тем, кто не пьет, выпивает совсем немного или пьёт только по праздникам, таким как Новый год или День рождения. Пожалуйста, вспомните все случаи, когда Вы могли употреблять алкогольные напитки.**

**G1 – G10. Для каждого из видов алкоголя, обозначенного слева, укажите, как часто Вы употребляете данный вид алкоголя в течение последних 12 месяцев?** *Покажите карточку 4.*

|  | каждый день и чаще | почти каждый день | 3-4 раза в неделю | 1-2 раза в неделю | 1-3 раза в месяц | несколько раз в год | никогда или почти никогда | затрудняюсь ответить | отказ от ответа |
| --- | --- | --- | --- | --- | --- | --- | --- | --- | --- |
| **G1.** Любой алкоголь | 1 | 2 | 3 | 4 | 5 | 6 | 7 | 97 | 98 |

| **G2.** Пиво | 1 | 2 | 3 | 4 | 5 | 6 | 7 | 97 | 98 |
| --- | --- | --- | --- | --- | --- | --- | --- | --- | --- |
| **G3.** Вино (Не домашнего приготовления) | 1 | 2 | 3 | 4 | 5 | 6 | 7 | 97 | 98 |
| **G4.** Крепленое вино (н-р, портвейн) | 1 | 2 | 3 | 4 | 5 | 6 | 7 | 97 | 98 |
| **G5.** Крепкие напитки (водка, коньяк, виски, джин, ром, и т.д.) | 1 | 2 | 3 | 4 | 5 | 6 | 7 | 97 | 98 |
| **G6.** Cпиртосодержащие жидкости (спиртосодержащие жидкости, не предназначенные для употребления в качестве напитков, включая одеколоны, медицинские настойки, и другие подобные вещи, которые можно купить в магазинах, киосках, аптеках) | 1 | 2 | 3 | 4 | 5 | 6 | 7 | 97 | 98 |
| **G7.** Cпирт | 1 | 2 | 3 | 4 | 5 | 6 | 7 | 97 | 98 |
| **G8.** Cамогон | 1 | 2 | 3 | 4 | 5 | 6 | 7 | 97 | 98 |
| **G9.** Домашнее вино, брага | 1 | 2 | 3 | 4 | 5 | 6 | 7 | 97 | 98 |
| **G10.** Алкогольные коктейли в банках | 1 | 2 | 3 | 4 | 5 | 6 | 7 | 97 | 98 |

*.*

| G10_1 | Сколько дней в течение последних 3х месяцев (до данной госпитализации) Вы НЕ ВЫПИВАЛИ/не употребляли алкоголь?___________________ | | | | | | | | |
| --- | --- | --- | --- | --- | --- | --- | --- | --- | --- |
| 97  98 | Затрудняюсь ответить  Отказ от ответа | | | | | | | | |
| G11. | Если говорить о последних 12 месяцах, в случаях, когда Вы выпивали пиво, какое количество Вы обычно употребляли? *Возможен только один вариант ответа.* | | | | | | | | |
| 1 2 3 4 4a | 1 бутылку (0,5л) и меньше  2-4 бутылки (по 0,5л)  5-6 бутылки (по 0,5л)  Более 6 бутылок (по 0,5л)  Пожалуйста уточните сколько бутылок __________ (Если более 6) | | | | | | | | |
| 97  98 | Затрудняюсь ответить  Отказ от ответа | | | | | | | | |
|  |  | | | | | | | | |
| G12. | Если говорить о последних 12 месяцах, в случаях, когда Вы выпивали вино, какое количество Вы обычно употребляли? *Возможен только один вариант ответа.* | | | | | | | | |
| 1 2 3 4 5 5a  97  98 | Менее 200г  200 – 400г  400 – 600г  600 – 1000г  Более 1 литра  Пожалуйста уточните сколько грамм __________ (Если более 1000)  Затрудняюсь ответить  Отказ от ответа | | | | | | | | |
| G13. | Если говорить о последних 12 месяцах, в случаях, когда Вы выпивали крепкие спиртые напитки (водку, коньяк, виски), какое количество Вы обычно употребляли? *Возможен только один вариант ответа.* | | | | | | | | |
| 1 2 3 4 5 6 7  7a | До 50 г  50 – 100г  100 – 200г  200 – 300г  300 – 400г  400 – 500г  Более 500г  Пожалуйста уточните сколько грамм __________ (Если более 500) | | | | | | | | |
| 97  98 | Затрудняюсь ответить  Отказ от ответа | | | | | | | | |
|  |  | | | | | | | | |
| G14. | Если говорить о последних 12 месяцах, в случаях, когда Вы выпивали пиво, какое самое максималное количество Вы употребляли? *Возможен только один вариант ответа.* | | | | | | | | |
| 1 2 3 4 4a | 1 бутылку (0,5л) и меньше  2-4 бутылки (0,5л)  5-6 бутылки (0,5л)  Более 6 бутылок (0,5л)  Пожалуйста уточните сколько бутылок __________ (Если более 6) | | | | | | | | |
| 97  98 | Затрудняюсь ответить  Отказ от ответа | | | | | | | | |
|  |  | | | | | | | | |
|  |  | | | | | | | | |
| G15. | Если говорить о последних 12 месяцах, в случаях, когда Вы выпивали вино, какое самое максималное количество Вы употребляли? *Возможен только один вариант ответа.* | | | | | | | | |
| 1 2 3 4 5 5a | Менее 200г  200 – 400г  400 – 600г  600 – 1000г  Более 1 литра  Пожалуйста уточните сколько грамм __________ (Если более 1000) | | | | | | | | |
| 97  98 | Затрудняюсь ответить  Отказ от ответа | | | | | | | | |
|  |  | | | | | | | | |
| G16. | Если говорить о последних 12 месяцах, в случаях, когда Вы выпивали крепкие спиртые напитки (водку, коньяк, виски), какое самое максималное количество Вы употребляли? *Возможен только один вариант ответа.* | | | | | | | | |
| 1  2  3  4  5  6  7  7a | До 50 г  50 – 100г  100 – 200г  200 – 300г  300 – 400г  400 – 500г  Более 500г  Пожалуйста уточните сколько грамм __________ (Если более 500) | | | | | | | | |
| 97  98 | Затрудняюсь ответить  Отказ от ответа | | | | | | | | |
|  |  | | | | | | | | |
| G17. | Как часто Вы выпиваете крепкие напитки, такие, как водка или коньяк, в большом количестве без закуски? *Возможен только один вариант ответа.* | | | | | | | | |
| 1 2 3 | Всегда  Иногда  Никогда или почти никогда | | | | | | | | |
| 97  98 | Затрудняюсь ответить  Отказ от ответа | | | | | | | | |
|  |  | | | | | | | | |
| G18. | Как часто Вы «перепиваете»? *Возможен только один вариант ответа. Покажите карточку 5.* | | | | | | | | |
| 1 2 3 4 5 6  7 | Каждый день  Несколько раз в неделю  Примерно один раз в неделю  Несколько раз в месяц  Примерно один раз в месяц  Меньше, чем один раз в месяц  Никогда или почти никогда | | | | | | | | |
| 97  98 | Затрудняюсь ответить  Отказ от ответа | | | | | | | | |
|  |  | | | | | | | | |
| G19. | Употребляете ли Вы когда-нибудь алкоголь в первой половине дня (до 12 часов)? *Возможен только один вариант ответа.* | | | | | | | | |
| 1 2 3 | Нет  Да, иногда  Да, часто | | | | | | | | |
| 97  98 | Затрудняюсь ответить  Отказ от ответа | | | | | | | | |
| G20. | Как часто у Вас бывает похмелье? *Возможен только один вариант ответа. Покажите карточку 5.* | | | | | | | | |
| 1 2 3 4 5 6 7 | Каждый день  Несколько раз в неделю  Примерно один раз в неделю  Несколько раз в месяц  Примерно один раз в месяц  Меньше, чем один раз в месяц  Никогда или почти никогда | | | | | | | | |
| 97  98 | Затрудняюсь ответить  Отказ от ответа | | | | | | | | |
| G21. | Как часто Вы не можете выполнять какие-то семейные или личные обязанности из-за употребления алкоголя? *Возможен только один вариант ответа. Покажите карточку 5.* | | | | | | | | |
| 1 2 3 4 5 6 7 | Каждый день  Несколько раз в неделю  Примерно один раз в неделю  Несколько раз в месяц  Примерно один раз в месяц  Меньше, чем один раз в месяц  Никогда или почти никогда | | | | | | | | |
| 97  98 | Затрудняюсь ответить  Отказ от ответа | | | | | | | | |
|  |  | | | | | | | | |
| G22. | Ложитесь ли Вы когда-либо спать (на ночь) не раздеваясь из-за алкогольного опьянения? *Возможен только один вариант ответа. Покажите карточку 5.* | | | | | | | | |
| 1 2 3 4 5 6 7 | Каждый день  Несколько раз в неделю  Примерно один раз в неделю  Несколько раз в месяц  Примерно один раз в месяц  Меньше, чем один раз в месяц  Никогда или почти никогда | | | | | | | | |
| 97  98 | Затрудняюсь ответить  Отказ от ответа | | | | | | | | |
| **G23.** | **Были ли у Вас запои в течение последнего года?** | | | | | | | | |
| 1 | Да | | | | | | | | |
| 2 | Нет *Переход к G37* | | | | | | | | |
| 97 | Затрудняюсь ответить *Переход к G37* | | | | | | | | |
| 98 | отказ от ответа *Переход G37* | | | | | | | | |
|  |  | | | | | | | | |
| **G24.** | **Сколько запоев было у Вас в течение последнего года?** | | | | | | | | |
| 1 2 3  4 | 1  2-4  5-9  10 и болеe | | | | | | | | |
| 97  98 | Затрудняюсь ответить  Отказ от ответа *Переход к G37* | | | | | | | | |
|  |  | | | | | | | | |
|  |  | | | | | | | | |
|  |  | | | | | | | | |
|  |  | | | | | | | | |
| **G25.** | **Были ли у Вас запои в течение последнего месяца**? | | | | | | | | |
| 1 2 | Да  Нет | | | | | | | | |
| 97  98 | Затрудняюсь ответить  Отказ от ответа | | | | | | | | |
| **G26.** | **Как долго у Вас длится типичный запой?** | | | | | | | | |
|  |  | | | | | | | | |
| 1 2 3  4 | 2-3 дня  4-5 дней  6-9 дней  10 дней и более | | | | | | | | |
| 97  98 | Затрудняюсь ответить  Отказ от ответа | | | | | | | | |
|  |  | | | | | | | | |
| **GN26a.** | | **Когда у Вас начались запои?** | | | | | | | |
| 1 | | 6 месяцев назад или менее | | | | | | | |
| 2 | | 7-12 месяцев назад | | | | | | | |
| 3 | | 1-3 года назад | | | | | | | |
| 4 | | 4-5 лет назад | | | | | | | |
| 5 | | 6-9 лет назад | | | | | | | |
| 6 | | 10 лет назад и более | | | | | | | |
| 97  98 | | Затрудняюсь ответить  Отказ от ответа | | | | | | | |
| G37. За последние 12 месяцев испытывали ли Вы следующее? | | | Да | Нет | Затрудняюсь ответить | | | Отказ от ответа |  |
| a | Чувствовали ли Вы, что должны уменьшить употребление алкоголя? | | 1 | 2 | | 97 | 98 | |  |
| b | Надоедали ли Вам люди, критикующие Вас за выпивку? | | 1 | 2 | | 97 | 98 | |  |
| c | Вы чувствовали себя плохим или виноватым из-за Вашей выпивки? | | 1 | 2 | | 97 | 98 | |  |
| d | Вы когда-нибудь выпивали первым делом утром, чтобы успокоить нервы или избавиться от похмелья? | | 1 | 2 | | 97 | 98 | |  |

**Последний эпизод проблемного употребления алкоголя.**

**Сейчас я хотела бы поговорить с Вами о последнем (до настоящей госпитализации) проблемном употреблении алкоголя**

**Проблемное употребление алкоголя - это такое употребление спиртного, которое приводит к негативным последствиям. Например, возникают проблемы со здоровьем, неприятности в семейной жизни или на работе, проблемы с полицией и т.д.**

| GN38.  1  2  97  98 | **Это был запой? Запой – это многодневное и непрерывное употребление алкоголя, при котором человек не участвует в социальной и профессиональной жизни**  Да *Переход к GN49*  Нет  Затрудняюсь ответить  Отказ от ответа |
| --- | --- |
| GN39. | **Когда начался этот последний эпизод?** |
|  | Дата: __ __ / __ __ / __ __ __ __ |
| 97  98 | Затрудняюсь ответить *Переход к GN40*  Отказ от ответа |
|  |  |
| GN40. | **Сколько** Дней назад ______ Недель назад _____ Месяцев назад_______ Лет назад ______ |
|  |  |
| 97  98 | Затрудняюсь ответить  Отказ от ответа |
|  |  |
| GN41. | **Сколько длился данный эпизод проблемного употребления алкоголя?** |
|  | Дней______ Недель_____ Месяцев_______ Лет |
|  |  |
| 97  98 | Затрудняюсь ответить  Отказ от ответа |

**Теперь я задам Вам вопросы о спиртных напитках, которые Вы употребляли в этот последний эпизод.**

| GN42. | **Во время данного эпизода Вы выпивали пиво?** |
| --- | --- |
|  |  |
| 1  2  97  98 | Да  Нет *Переход к GN43*  Затрудняюсь ответить  Отказ от ответа |
| GN42a. | **В случаях, когда Вы выпивали пиво, сколько пива Вы выпивали в среднем в сутки?** (Бутылок (по 0,5л)________________ |
| 97  98 | Затрудняюсь ответить  Отказ от ответа |
| GN42b. | **В** **случаях, когда Вы выпивали пиво, какое самое максималное количество Вы употребляли в сутки?** (Бутылок (по 0,5л)____________ |
| 97  98 | Затрудняюсь ответить  Отказ от ответа |
|  |  |
| GN43. | **Во время данного эпизода Вы выпивали вино?** |
| 1  2  97  98 | Да  Нет *Переход к GN44*  Затрудняюсь ответить  Отказ от ответа |
| GN43a. | **В случаях, когда Вы выпивали вино, сколько вина Вы выпивали в среднем в сутки?**  (Грамм)________________ |
| 97  98 | Затрудняюсь ответить  Отказ от ответа |
| GN43b. | **В** **случаях, когда Вы выпивали вино, какое самое максималное количество вина Вы употребляли в сутки?** (Грамм)____________ |
| 97  98 | Затрудняюсь ответить  Отказ от ответа |
|  |  |
| GN44. | **Во время данного эпизода Вы выпивали креплёное вино?** |
| 1  2  97  98 | Да  Нет *Переход к GN45*  Затрудняюсь ответить  Отказ от ответа |
| GN44a. | **В случаях, когда Вы выпивали креплёное вино, сколько креплёного вина Вы выпивали в среднем в сутки?** (Грамм)____________ |
| 97  98 | Затрудняюсь ответить  Отказ от ответа |
| GN44b. | **В** **случаях, когда Вы выпивали креплёное вино, какое самое максималное количество креплёного вина Вы употребляли в сутки ?** (Грамм)____________ |
| 97  98 | Затрудняюсь ответить  Отказ от ответа |
|  |  |
| GN45. | **Во время данного эпизода Вы выпивали крепкие спиртые напитки (водку, коньяк, виски)?** |
| 1  2  97  98 | Да  Нет *Переход к GN46*  Затрудняюсь ответить  Отказ от ответа |
| GN45a. | **В случаях, когда Вы выпивали крепкие спиртые напитки, сколько Вы выпивали в среднем в сутки?** (Грамм)____________ |
| 97  98 | Затрудняюсь ответить  Отказ от ответа |
| GN45b. | **В** **случаях, когда Вы выпивали крепкие спиртые напитки, какое самое максималное количество Вы употребляли в сутки?** (Грамм)____________ |
| 97  98 | Затрудняюсь ответить  Отказ от ответа |
| GN46. | **Во время данного эпизода Вы выпивали спирт** **(медицинский/технический/другой)?** |
| 1  2  97  98 | Да  Нет *Переход к GN47*  Затрудняюсь ответить  Отказ от ответа |
| GN46a. | **В случаях, когда Вы выпивали спирт** **(медицинский/технический/другой), сколько Вы выпивали в среднем в сутки?** (Грамм)____________ |
| 97  98 | Затрудняюсь ответить  Отказ от ответа |
|  |  |
| GN46b. | **В** **случаях, когда Вы выпивали спирт** **(медицинский/технический/другой), какое самое максималное количество Вы употребляли в сутки?** (Грамм)____________ |
| 97  98 | Затрудняюсь ответить  Отказ от ответа |
|  |  |
| GN47. | **Во время данного эпизода Вы выпивали алкогольные коктейли (н-р, джин-и-тоник)?** |
| 1  2  97  98 | Да  Нет Переход к GN48  Затрудняюсь ответить  Отказ от ответа |
| GN47a. | **В случаях, когда Вы выпивали алкогольные коктейли, сколько Вы выпивали в среднем в сутки?** (Грамм)____________ |
| 97  98 | Затрудняюсь ответить  Отказ от ответа |
|  |  |
| GN47b. | **В** **случаях, когда Вы выпивали алкогольные коктейли, какое самое максималное количество Вы употребляли в сутки?** (Грамм)____________ |
| 97  98 | Затрудняюсь ответить  Отказ от ответа |
|  |  |
| GN48. | **Во время данного эпизода Вы выпивали другие спиртовые настойки, лосьоны (“Боярышник”, “Перец”, "Чистый свет", "Зверобой", “Тройной одеколон”, “Трояр”)?** |
| 1  2  97  98 | Да  Нет Переход к GN49  Затрудняюсь ответить  Отказ от ответа |
|  |  |
| GN48a. | **В случаях, когда Вы выпивали другие спиртовые настойки, сколько Вы выпивали в среднем в сутки?** (Грамм)____________ |
| 97  98 | Затрудняюсь ответить  Отказ от ответа |
|  |  |
| GN48b. | **В** **случаях, когда Вы выпивали другие спиртовые настойки, какое самое максималное количество Вы употребляли в сутки?** (Грамм)____________ |
| 97  98 | Затрудняюсь ответить  Отказ от ответа |

|  | **Последний проблемный эпизод употребления алкоголя. Запой.** |
| --- | --- |
|  |  |
| GN49. | **Когда начался этот запой?**  Дата: __ __ / __ __ / __ __ __ __ |
| 97  98 | Затрудняюсь ответить *Переход к GN50*  Отказ от ответа |
|  |  |
| GN50. | **Сколько**  Дней назад ______ Недель назад _____ Месяцев назад_______ Лет назад ______ |
| 97  98 | Затрудняюсь ответить  Отказ от ответа |
|  |  |
| GN51. | **Сколько длился этот запой?**  Дней______ Недель_____ Месяцев_______ Лет |
| 97  98 | Затрудняюсь ответить  Отказ от ответа |
|  |  |
| GN52. | **Во время данного эпизода запоя Вы выпивали пиво?** |
| 1  2  97  98 | Да  Нет *Переход к GN53*  Затрудняюсь ответить  Отказ от ответа |
| GN52a. | **В случаях, когда Вы выпивали пиво, сколько пива Вы выпивали в среднем в сутки?** (Бутылок (по 0,5л)____________ |
| 97  98 | Затрудняюсь ответить  Отказ от ответа |
| GN52b. | **В** **случаях, когда Вы выпивали пиво, какое самое максималное количество Вы употребляли в сутки?** (Бутылок (по 0,5л)____________ |
| 97  98 | Затрудняюсь ответить  Отказ от ответа |
|  |  |
| GN53. | **Во время данного эпизода запоя Вы выпивали вино?** |
| 1  2  97  98 | Да  Нет *Переход к GN54*  Затрудняюсь ответить  Отказ от ответа |
| GN53a. | **В случаях, когда Вы выпивали вино, сколько вина Вы выпивали в среднем в сутки? (грамм)________________** |
| 97  98 | Затрудняюсь ответить  Отказ от ответа |
| GN53b. | **В** **случаях, когда Вы выпивали вино, какое самое максималное количество вина Вы употребляли в сутки?** (Грамм)____________ |
| 97  98 | Затрудняюсь ответить  Отказ от ответа |
|  |  |
| GN54. | **Во время данного эпизода запоя Вы выпивали креплёное вино?** |
| 1  2  97  98 | Да  Нет *Переход к GN55*  Затрудняюсь ответить  Отказ от ответа |
| GN54a. | **В случаях, когда Вы выпивали креплёное вино, сколько креплёного вина Вы выпивали в среднем в сутки?** (Грамм)____________ |
| 97  98 | Затрудняюсь ответить  Отказ от ответа |
| GN54b. | **В** **случаях, когда Вы выпивали креплёное вино, какое самое максималное количество креплёного вина Вы употребляли в сутки ?** (Грамм)____________ |
| 97  98 | Затрудняюсь ответить  Отказ от ответа |
|  |  |
| GN55. | **Во время данного эпизода запоя Вы выпивали крепкие спиртые напитки (водку, коньяк, виски)?** |
| 1  2  97  98 | Да  Нет *Переход к GN56*  Затрудняюсь ответить  Отказ от ответа |
| GN55a. | **В случаях, когда Вы выпивали крепкие спиртые напитки, сколько Вы выпивали в среднем в сутки?** (Грамм)____________ |
| 97  98 | Затрудняюсь ответить  Отказ от ответа |
|  |  |
| GN55b. | **В** **случаях, когда Вы выпивали крепкие спиртые напитки, какое самое максималное количество Вы употребляли в сутки?** (Грамм)____________ |
| 97  98 | Затрудняюсь ответить  Отказ от ответа |
|  |  |
| GN56. | **Во время данного эпизода запоя Вы выпивали спирт (медицинский/технический/другой)?** |
| 1  2  97  98 | Да  Нет *Переход к GN57*  Затрудняюсь ответить  Отказ от ответа |
| GN56a. | **В случаях, когда Вы выпивали спирт (медицинский/технический/другой), сколько Вы выпивали в среднем в сутки?** (Грамм)____________ |
| 97  98 | Затрудняюсь ответить  Отказ от ответа |
| GN56b. | **В** **случаях, когда Вы выпивали спирт, какое самое максималное количество Вы употребляли в сутки?** (Грамм)____________ |
| 97  98 | Затрудняюсь ответить  Отказ от ответа |
|  |  |
|  |  |
|  |  |
|  |  |
| GN57. | **Во время данного эпизода запоя Вы выпивали алкогольные коктейли (н-р, джин-и-тоник)?** |
| 1  2  97  98 | Да  Нет *Переход к G27*  Затрудняюсь ответить  Отказ от ответа |
| GN57a. | **В случаях, когда Вы выпивали алкогольные коктейли, сколько Вы выпивали в среднем в сутки?** (Грамм)____________ |
| 97  98 | Затрудняюсь ответить  Отказ от ответа |
| GN57b. | **В** **случаях, когда Вы выпивали алкогольные коктейли, какое самое максималное количество Вы употребляли в сутки?** (Грамм)____________ |
| 97  98 | Затрудняюсь ответить  Отказ от ответа |
| G27. | **Во время данного эпизода запоя употребляли ли Вы спиртосодержащие жидкости, не предназначенные для употребления в качестве напитков? Здесь мы имеем ввиду такие жидкости как одеколоны, медицинские настойки, лосьоны.** |
| 1  2  97  98 | Да  Нет *Переход к GN58*  Затрудняюсь ответить *Переход к GN58*  Отказ от ответа *Переход к GN58* |
| G27a. | **В случаях, когда Вы выпивали другие спиртовые настойки, сколько Вы выпивали в среднем в сутки?** (Грамм)____________ |
| 97  98 | Затрудняюсь ответить  Отказ от ответа |
|  |  |
| G27b. | **В** **случаях, когда Вы выпивали другие спиртовые настойки, какое самое максималное количество Вы употребляли в сутки?** (Грамм)____________ |
| 97  98 | Затрудняюсь ответить  Отказ от ответа |
|  |  |

**Oбращение за наркологической помощью**

| **GN58.** | **Каковы причины Вашего поступления в стационар/обращения за наркологической помощью?** *Возможно несколько вариантов ответа.* |
| --- | --- |
| 1  2  3  4  5  6  7 8  9  10  11 | Не мог сам прекратить пить  Плохое состояние здоровья, не позволяющее выпивать  Появились галлюцинации  Повлияли просьбы кого-то из близких или друзей  По просьбе/направлению работодателя  Порекомендовал врач  Боялся потерять работу  Боялся умереть  Хочу закодироваться  Были финансовые причины  Хочу "почистить" организм, поправить здоровье |
| 97  98 | Затрудняюсь ответить  Отказ от ответа |
|  | **Сейчас я Вам задам вопросы о каждом виде помощи, направленной на снижение или полного прекращения употребления алкоголя, которую Вы могли получать.** |
|  |  |
| GN59. | Получали ли Вы когда-либо помощь (или консультации) медиков, наркологов, других специалистов по поводу проблем с алкоголем до данной госпитализации? |
| 1  2 | Да  Нет Переход к GN69 |
| 97  98 | Затрудняюсь ответить  Отказ от ответа Переход к GN69 |
|  |  |
| **GN60.** | **Получали ли Вы когда-либо помощь в наркологическом диспансере, медикаментозую помощь?** |
| 1  2 | Да  Нет Переход к GN61 |
| 97  98 | Затрудняюсь ответить  Отказ от ответа Переход к GN61 |
|  |  |
| **GN60a.** | **Сколько раз Вы получали данную помощь?** Количество раз**____________** |
| 97  98 | Затрудняюсь ответить  Отказ от ответа |
|  |  |
| **GN61.** | **Получали ли Вы когда-либо помощь нарколога на дому или в частной клинике (медикаментозная помощь, н-р, капельница)?** |
| 1  2 | Да  Нет Переход к GN62 |
| 97  98 | Затрудняюсь ответить  Отказ от ответа Переход к GN62 |
|  |  |
| **GN61a.** | **Сколько раз Вы получали данную помощь?** Количество раз____________ |
| 97  98 | Затрудняюсь ответить  Отказ от ответа |
|  |  |
|  |  |
|  |  |
|  |  |
|  |  |
| **GN62.** | **Кодировались ли Вы когда-либо (н-р, с использованием гипноза, вшивания, торпеды, уколов)?** |
| 1  2 | Да  Нет Переход к GN63 |
| 97  98 | Затрудняюсь ответить  Отказ от ответа Переход к GN63 |
|  |  |
| **GN62a.** | **Сколько раз Вы кодировались?** Количество раз____________ |
| 97  98 | Затрудняюсь ответить  Отказ от ответа |
| **GN63.** | **Получали ли Вы когда-либо помощь психолога?** |
| 1  2 | Да  Нет Переход к GN64 |
| 97  98 | Затрудняюсь ответить  Отказ от ответа Переход к GN64 |
|  |  |
| **GN63a.** | **Сколько раз Вы получали данную помощь?** Количество раз____________ |
| 97  98 | Затрудняюсь ответить  Отказ от ответа |
|  |  |
| **GN64.** | **Проходили ли Вы курс психотерапии в связи с употреблением алкоголя?** |
| 1  2 | Да  Нет Переход к GN65 |
| 97  98 | Затрудняюсь ответить  Отказ от ответа Переход к GN65 |
|  |  |
| **GN64a.** | **Сколько раз Вы получали данную помощь?** Количество раз____________ |
| 97  98 | Затрудняюсь ответить  Отказ от ответа |
| **GN65.** | **Проходили ли Вы курс поведенческой терапии в связи с употреблением алкоголя?** |
| 1  2 | Да  Нет Переход к GN66 |
| 97  98 | Затрудняюсь ответить  Отказ от ответа Переход к GN66 |
|  |  |
| **GN65a.** | **Сколько раз Вы получали данную помощь?** Количество раз____________ |
| 97  98 | Затрудняюсь ответить  Отказ от ответа |
|  |  |
| **GN66.** | **Проходили ли Вы курс групповой терапии (12 шагов, анонимные алкоголики) в связи с употреблением алкоголя?** |
| 1  2 | Да  Нет Переход к GN67 |
| 97  98 | Затрудняюсь ответить  Отказ от ответа Переход к GN67 |
|  |  |
| **GN66a.** | **Сколько раз Вы получали данную помощь?** Количество раз____________ |
| 97  98 | Затрудняюсь ответить  Отказ от ответа |
|  |  |
|  |  |
| **GN67.** | **Получали ли Вы когда-либо помощь знахарки/знахаря или другого народного целителя в связи с употреблением алкоголя?** |
| 1  2 | Да  Нет Переход к GN68 |
| 97  98 | Затрудняюсь ответить  Отказ от ответа Переход к GN68 |
|  |  |
| **GN67a.** | **Сколько раз Вы получали данную помощь?** Количество раз____________ |
| 97  98 | Затрудняюсь ответить  Отказ от ответа |
| **GN68.** | **Получали ли Вы такую помощь (или консультацию) в течение последнего года, исключая данную госпитализацию?** |
| 1  2 | Да  Нет |
| 97  98 | Затрудняюсь ответить  Отказ от ответа |

|  | Употребление спиртосодержащих жидкостей |
| --- | --- |
|  |  |
| GN69. | Употребляли ли Вы когда-либо спиртосодержащие жидкости, не предназначенные для употребления в качестве напитков? Здесь мы имеем ввиду такие жидкости как одеколоны, медицинские настойки, спирт, лосьоны ("Боярышник" , “Перец”, "Чистый свет", "Зверобой", “Тройной одеколон”). |
| 1 | Да |
| 2 | Нет Переход к D1 |
| 97  98 | Затрудняюсь ответить  Отказ от ответа |
|  |  |
| GN70. | Когда Вы начали употреблять спиртосодержащие жидкости? |
| 1  2  3  4 | В последний месяц  В последние 6 месяцев  В последний год  Больше года назад |
| 97  98 | Затрудняюсь ответить  Отказ от ответа |
|  |  |
| GN71. | Какие спиртосодержащие жидкости Вы употребляете? *Возможно несколько вариантов ответа.* |
| 1  2  3  4  5  6  7  8  9  10  10a | Настойка боярышника  Трояр  Непитьевой спирт (технический, медицинский и т.д.)  Композиция  Тройной одеколон  Лосьон «Перец»  Лосьон "Зверобой"  Лосьон "Чистый свет"  Жидкость для мытья окон, другие моющие средства  Другие спиртовые настойки  Другое. Уточните .................... .................... |
| 97  98 | Затрудняюсь ответить  Отказ от ответа |
|  |  |
| **GN72.** | **Когда Вы употребляете спиртосодержащие жидкости, не предназначенные для употребления в качестве напитков, включая спирт, как часто Вы их разбавляете водой или другими безалкогольными напитками (н-р, сок, кока-кола)?** |
| 1  2  3  4  5 | Никогда  Редко  Иногда  В большинстве случаев  Всегда |
| 97  98 | Затрудняюсь ответить  Отказ от ответа |
|  |  |
|  |  |
|  |  |
|  |  |
|  |  |
|  |  |
| **GN73.** | **Где Вы приобретаете спиртосодержащие жидкости, не предназначенные для употребления в качестве напитков, включая спирт?** *Возможно несколько вариантов ответа****.*** |
| 1 | В аптеке |
| 2 | В киосках |
| 3  4 | В магазине  На работе |
| 5 | Продают по соседству |
| 6  7  7a | На улице  Другое  Другое. Уточните .................... .................... |
| 97  98 | Затрудняюсь ответить  Отказ от ответа |

| **Модуль D** | **ОБРАЩЕНИЕ ЗА МЕДИЦИНСКОЙ ПОМОЩЬЮ** |
| --- | --- |

**Данный раздел будет касаться обращения за медицинской помощью, а также наличию некоторых заболеваний и применению медицинских препаратов.**

| D1. | **Сколько раз за последние 12 месяцев Вы обращались за медицинской помощью к нижеперечисленным специалистам? Пожалуйста отметьте количество визитов к каждому специалисту.** Вид помощи \| Количество посещений за последние 12 месяцев \| \| \| \| \|; 1. Участковый врач/терапевт \| 0 \| 1 \| 2 \| 3 \| 4 \| 5+; 2. Кардиолог в поликлиннике \| 0 \| 1 \| 2 \| 3 \| 4 \| 5+; 3. Другой специалист в поликлиннике \| 0 \| 1 \| 2 \| 3 \| 4 \| 5+; 4. Кардиолог в больнице/ госпитале/ стационаре \| 0 \| 1 \| 2 \| 3 \| 4 \| 5+; 5. Другой специалист в больнице/госпитале/ стационаре, включая нарколога \| 0 \| 1 \| 2 \| 3 \| 4 \| 5+ | | | | | | |
| --- | --- | --- | --- | --- | --- | --- | --- |
|  |  | | | | | | |
| **D2.** | **Сколько раз Вы были госпитализированы за последние 12 месяцев? (провели в госпитале/больнице ночь/сутки)?**  Количество раз.................................... If “0” *Переход к D4* | | | | | | |
| 97  98 | Затрудняюсь ответить  Отказ от ответа | | | | | | |
| **D3.** | **Сколько дней (суток) Вы провели в больнице/госпитале в каждую из этих госпитализаций?** | | | | | | |
|  | Госпитализация | | Количество дней/суток | | | | |
| 1  2  3  4  5 |  | | .......................................  .......................................  .......................................  .......................................  ....................................... | | | | |
| 6 |  | | ....................................... | | | | |
| 7 |  | | ....................................... | | | | |
| 8 |  | | ....................................... | | | | |
| 9 |  | | ....................................... | | | | |
| 10 |  | | ....................................... | | | | |
| 97  98 | Затрудняюсь ответить  Отказ от ответа | |  | | | | |
|  |  | | | | | | |
| **D4.** | **Сколько раз Вы или кто-либо для Вас вызывали скорую помощь на протяжении последних 12 месяцев?** | | | | | | |
|  | Количество раз.................. | | | | | | |
| 97  98 | Затрудняюсь ответить  Отказ от ответа | | | | | | |
| **D10.** | **Говорил ли Вам когда-либо врач, что у Вас имеются/имелись, или были диагностированы следующие заболевания**: | | | | | | |
|  |  | Да | | Нет | Затрудняюсь ответить | Отказ от ответа |  |
| 1 | Артериальная гипертензия (повышенное давление). *Для женщин: за исключением периода беременности.* | 1 | | 2 | 97 | 98 |  |
| 1a | Артериальная гипертензия во время беременности (*Для женщин)* | 1 | | 2 | 97 | 98 |  |
| 2 | Повышенный уровень холестерина | 1 | | 2 | 97 | 98 |  |
| 3 | Инфаркт миокарда/Cердечный приступ | 1 | | 2 | 97 | 98 |  |
| 4 | Сердечная недостаточность | 1 | | 2 | 97 | 98 |  |
| 5 | Фибрилляция предсердий | 1 | | 2 | 97 | 98 |  |
| 6 | Стенокардия/Ишемическая болезнь сердца | 1 | | 2 | 97 | 98 |  |
| 7 | Инсульт | 1 | | 2 | 97 | 98 |  |
| 8 | Сахарный диабет | 1 | | 2 | 97 | 98 |  |
| 9 | Заболевание почек | 1 | | 2 | 97 | 98 |  |
| 10 | Хронический бронхит | 1 | | 2 | 97 | 98 |  |
| 11 | Онкологические заболевания/Рак | 1 | | 2 | 97 | 98 |  |
| 12 | Бронхиальная астма | 1 | | 2 | 97 | 98 |  |
| 13 | Ревматический артрит | 1 | | 2 | 97 | 98 |  |
| 14 | Остеоартрит (остеоартроз) | 1 | | 2 | 97 | 98 |  |
| 15 | Мигрень | 1 | | 2 | 97 | 98 |  |
| 16 | Депрессия | 1 | | 2 | 97 | 98 |  |
| 17 | Повышенная тревожность | 1 | | 2 | 97 | 98 |  |
|  | *Примечание: D11-D15 вопросы задаются тем респондентам, кто ответил, что имеет повышенное давление. D21-D25 вопросы задаются тем респондентам, кто ответил, что имеет повышенный холестерол. Остальные респонденты переходят к вопросу E1*. | | | | | | |
|  |  | | | | | | |
| **D11.** | **Назначал ли Вам врач (или другой медицинский работник) медикаментозные препараты для лечения артериальной гипертензии?** | | | | | | |
| 1  2 | Да  Нет *Переход к D21 (повышенный уровень холестерина- переход к D16)* | | | | | | |
| 97  98 | Затрудняюсь ответить *Переход к D21* *(повышенный уровень холестерина- переход к D16)*  отказ от ответа *Переход к E1(повышенный уровень холестерина- переход к D16)* | | | | | | |
| **D12.** | **Приобрели ли Вы все медицинские препараты, назначенные врачом?** | | | | | | |
| 1  2 | Да *Переход к D14*  Нет | | | | | | |
| 97  98 | Затрудняюсь ответить  Отказ от ответа *Переход к D14* | | | | | | |
|  |  | | | | | | |
|  |  | | | | | | |
|  |  | | | | | | |
|  |  | | | | | | |
|  |  | | | | | | |
|  |  | | | | | | |
| **D13.** | **Каковы были причины того, что Вы не приобрели все назначенные препараты?** *Возможно несколько вариантов ответа.* | | | | | | |
| 1 | Назначенные препараты отсутствовали в аптеке | | | | | | |
| 2 | Назначенные препараты дорого стоили | | | | | | |
| 3 | Назначенные препараты неэффективны | | | | | | |
| 4 | Назначенные препараты имеют побочные эффекты | | | | | | |
| 5 | Я предпочитаю принимать для снижения артериального давления другие медикаментозные препараты, рекомендованными родственниками/друзьями | | | | | | |
| 6 | Я предпочитаю принимать для лечения артериальной гипертензии другие медикаментозные препараты, рекомендованные фармацевтом | | | | | | |
| 7 | Я предпочитаю принимать для лечения артериальной гипертонии другие альтернативные, немедицинские методы | | | | | | |
| 8  8a | Другое  Другое. Уточните .................... .................... | | | | | | |
| 97  98 | Затрудняюсь ответить  Отказ от ответа | | | | | | |
|  |  | | | | | | |
| **D14.** | **Принимаете ли Вы рекомендованные медикаментозные препараты каждый день?** | | | | | | |
| 1  2  3 | Да *Переход D16*  Нет  Не приобрел/а никаких медицинских препаратов *Переход D16* | | | | | | |
| 97  98 | Затрудняюсь ответить  Отказ от ответа | | | | | | |
| **D15.** | **В чем причина того, что Вы не принимали рекомендованные препараты каждый день?** *Возможно несколько вариантов ответа* | | | | | | |
| 1 | У меня больше нет повышенного давления | | | | | | |
| 2 | Я не знал, что лекарства необходимо принимать каждый день | | | | | | |
| 3 | Врач сказал мне, что я должен принимать лекарства только тогда, когда есть симптомы | | | | | | |
| 4 | Я не могу (финансово) позволить себе принимать их каждый день | | | | | | |
| 5 | Я забываю принимать их каждый день | | | | | | |
| 6 | Я прекращаю принимать их, когда начинаю чувствовать себя хуже | | | | | | |
| 7 | Я прекращаю принимать их, когда начинаю чувствовать себя лучшe | | | | | | |
| 8 | Употребление препаратов каждый день влечет за собой появление побочных эффектов | | | | | | |
| 9 | Я предпочитаю принимать безрецептурные препараты | | | | | | |
| 10.  10a | Другое  Другое. Уточните .................... .................... | | | | | | |
| 97  98 | Затрудняюсь ответить  Отказ от ответа | | | | | | |
|  |  | | | | | | |
| **D16.** | **Рекомендовал ли Вам врач (или другой медицинский работник) медикаментозные препараты (лекарства) для снижения повышенного уровня холестерина)?** | | | | | | |
| 1  2 | Да  Нет *Переход E1* | | | | | | |
| 97  98 | Затрудняюсь ответить *Переход E1*  Отказ от ответа *Переход E1* | | | | | | |
| **D17.** | **Приобрели ли Вы все медикаментозные препараты (лекарства), выписанные врачом?** | | | | | | |
| 1  2 | Да *Переход к D14*  Нет | | | | | | |
| 97  98 | Затрудняюсь ответить  Отказ от ответа | | | | | | |
| **D18.** | **Каковы были причины того, что Вы не приобрели все выписанные препараты?** *Возможно несколько вариантов ответа* | | | | | | |
| 1  2 | Назначенные препараты отсутствовали в аптеке  Назначенные препараты дорого стоили | | | | | | |
| 3 | Назначенные препараты неэффективны | | | | | | |
| 4 | Назначенные препараты имеют побочные эффекты | | | | | | |
| 5 | Я предпочитаю принимать для снижения холестерина другие медикаментозные препараты, рекомендованныв родственниками/друзьями | | | | | | |
| 6 | Я предпочитаю принимать для снижения холестерина другие медикаментозные препараты, рекомендованные фармацевтом | | | | | | |
| 7 | Я предпочитаю принимать для снижения холестерина другие альтернативные, немедицинские методы | | | | | | |
| 8  8a | Другое  Другое. Уточните .................... .................... | | | | | | |
| 97  98 | Затрудняюсь ответить  Отказ от ответа | | | | | | |
|  |  | | | | | | |
| **D19.** | **Принимаете ли Вы рекомедованные лекарства каждый день?** | | | | | | |
| 1.  2  3. | Да *Переход E1*  Нет  Не приобрел/а никаких медицинских препаратов | | | | | | |
| 97  98 | Затрудняюсь ответить  Отказ от ответа | | | | | | |
| **D20.** | **В чем причина того, что Вы не принимали рекомендованные препараты каждый день?** *Возможно несколько вариантов ответа* | | | | | | |
| 1  2  3  4  5  6  7  8  9  10  10a | У меня больше нет повышенного холестерина  Я не знал, что лекарства необходимо принимать каждый день.  Врач сказал мне, что я должен принимать лекарства только тогда, когда есть симптомы.  Я не могу (финансово) позволить себе принимать их каждый день.  Я забываю принимать их каждый день.  Я прекращаю принимать их, когда начинаю чувствовать себя хуже.  Я прекращаю принимать их, когда начинаю чувствовать себя лучшe.  Употребление препаратов каждый день влечет за собой появление побочных эффектов.  Я предпочитаю принимать безрецептурные препараты.  Другое  Другое. Уточните .................... .................... | | | | | | |
| 97  98 | Затрудняюсь ответить  Отказ от ответа | | | | | | |
| **D21.** | **Помимо выписанных препаратов приобретали ли Вы другие медикаменты/лекарства не рекомендованные врачом (те, которые порекомендовали фармацевт, друзья/родственники) для снижения повышенного давления и/или холестерина?** | | | | | | |
| 1  2 | Да  Нет | | | | | | |
| 97  98 | Затрудняюсь ответить  Отказ от ответа | | | | | | |
|  |  | | | | | | |
|  |  | | | | | | |
|  |  | | | | | | |

| **Модуль E** \| **ДЕПРЕССИЯ И ТРЕВОГА** **Сейчас я хотела бы поговорить о том, как Вы себя чувствовали в последние 2 недели.** **Как часто за последние 2 недели Вас беспокоили** **следующие проблемы?** *Покажите карточку 6.* | | Ни разу | Несколько дней | Более  Недели | Почти  каждый  день |  |  |
| --- | --- | --- | --- | --- | --- | --- | --- |
| **E1.** | Вам не хотелось ничего делать | 0 | 1 | 2 | 3 |  |  |
| **E2.** | У Вас было плохое настроение, Вы были подавлены или испытывали чувство безысходности | 0 | 1 | 2 | 3 |  |  |
| **E3.** | Вам было трудно заснуть, у Вас был прерывистый сон, или Вы слишком много спали | 0 | 1 | 2 | 3 |  |  |
| **E4.** | Вы были утомлены, или у Вас было мало сил | 0 | 1 | 2 | 3 |  |  |
| **E5.** | У Вас был плохой аппетит, или Вы переедали | 0 | 1 | 2 | 3 |  |  |
| **E6.** | Вы плохо о себе думали: считали себя неудачником (неудачницей)  или были в себе разочарованы, или считали, что подвели свою семью | 0 | 1 | 2 | 3 |  |  |
| **E7.** | Вам было трудно сосредоточиться (например, на чтении газеты или на просмотре телепередач) | 0 | 1 | 2 | 3 |  |  |
| **E8.** | Вы двигались или говорили настолько медленно, что окружающие это замечали? Или, наоборот, Вы были настолько суетливы или взбудоражены, что двигались гораздо больше обычного | 0 | 1 | 2 | 3 |  |  |
| **E9.** | Вас посещали мысли о том, что Вам лучше было бы умереть, или о том, чтобы причинить себе какой-нибудь вред | 0 | 1 | 2 | 3 |  |  |
| **E10.** | Oцените, насколько трудно Вам было работать, заниматься домашними делами или общаться с людьми из-за этих проблем? | Совсем не трудно | Немного трудно | Очень трудно | Чрезвычайно трудно |  |  |
|  |  | 0 | 1 | 2 | 3 |  |  |

| **Как часто за последние 2 недели Вас беспокоили**  **следующие проблемы?** *Покажите карточку 6.* | | Ни разу | Несколько дней | Более  Недели | Почти  каждый  день |  |  |
| --- | --- | --- | --- | --- | --- | --- | --- |
| **E11.** | Вы нервничали, тревожились или испытывали сильный стресс | 0 | 1 | 2 | 3 |  |  |
| **E12.** | Вы были неспособны успокоиться или контролировать свое волнение | 0 | 1 | 2 | 3 |  |  |
| **E13.** | Вы слишком сильно волновались по различным поводам | 0 | 1 | 2 | 3 |  |  |
| **E14.** | Вам было трудно расслабиться | 0 | 1 | 2 | 3 |  |  |
| **E15.** | Вы были настолько суетливы, что Вам было тяжело усидеть на месте | 0 | 1 | 2 | 3 |  |  |
| **E16.** | Вы легко злились или раздражались | 0 | 1 | 2 | 3 |  |  |
| **E17.** | Вы испытывали страх, словно должно произойти нечто ужасное | 0 | 1 | 2 | 3 |  |  |
| **E18.** | Oцените, насколько трудно Вам было работать, заниматься домашними делами или общаться с людьми из-за этих проблем? | Совсем не трудно | Немного трудно | Очень трудно | Чрезвычайно трудно |  |  |
|  |  | 0 | 1 | 2 | 3 |  |  |

| **Модуль H** | **КУРЕНИЕ** |
| --- | --- |

**Сейчас я задам несколько вопросов о курении**

| **H1.** | **Курите ли Вы в настоящее время?** *Возможен только один вариант ответа.* |
| --- | --- |
| 1 2 3 | Нет и никогда не курил (а) *Переход к K1*  Нет, бросил (а) *Переход к H2*  Да, курю *Переход к H3* |
| 97  98 | Затрудняюсь ответить *Переход к H3*  Отказ от ответа |
| H2. | Как давно Вы бросили курить? *Возможен только один вариант ответа.* |
| 1 2 3 4 | Не более1 года назад  От 1 до 5 лет назад  От 5 до 10 лет назад  Более 10 лет назад |
| 97  98 | Затрудняюсь ответить  Отказ от ответа |
| H3. | Что Вы курите (курили)чаще всего? *Возможен только один вариант ответа.* |
| 1 2 3 4  4a | Папиросы  Сигареты с фильтром  Сигареты без фильтра  Другое  Другое. Уточните .................... .................... |
| 97  98 | Затрудняюсь ответить  Отказ от ответа |
| H4. | **Когда Вы курите (курили), сколько Вы обычно выкуриваете (выкуривали) за день?** *Возможен только один вариант ответа.* |
| 1 2 3 | 10 сигарет и менее  Более 10, но не более 20 сигарет  Более 20-сигарет |
| 97  98 | Затрудняюсь ответить  Отказ от ответа |
| H5. | **В каком возрасте Вы начали курить регулярно?** ________Лет |
| 97  98 | Затрудняюсь ответить  Отказ от ответа |
|  |  |
| H6. | Советовали/рекомендовали ли Вам когда-либо врачи (участковый врач, терапевт, кардиолог, другие специалисты) бросить курить? |
| 1  2 | Да *Переход к H7*  Нет *Переход к K1* |
| 97  98 | Затрудняюсь ответить  Отказ от ответа |
| **H7.** | **Предложена ли была Вам помощь для того, чтобы бросить курить?** |
| 1  2 | Да  Нет *Переход к K1* |
| 97  98 | Затрудняюсь ответить  Отказ от ответа *Переход к K1* |
| **H8** | **Какая именно помощь была предложена?** *Возможно несколько вариантов ответа***.** |
| 1 | Направление на никотиновую заместительную терапию |
| 2 | Направление на поведенческую терапию |
| 3 | Направление на 12-ти шаговую программу |
| 4 | Кодирование |
| 5 | Материальная мотивация |
| 6  6a | Другое  Другое. Уточните .................... .................... |
| 97  98 | Затрудняюсь ответить  Отказ от ответа |
|  |  |
| **H8a** | **Изменилась ли частота Вашего курения после поступления в данное отделение?** |
| 1 | Нет |
| 2  3 | Да, увеличилась  Да, уменьшилась |
| 4  5 | Да, бросил/а курить  Да, начал/а курить |
| 97  98 | Затрудняюсь ответить  Отказ от ответа |
|  |  |
|  |  |
|  |  |
|  |  |

| **Модуль K** | **ПСИХОСОЦИАЛЬНЫЕ ФАКТОРЫ** |
| --- | --- |

| **K1.** **Происходили ли с Вами следующие события или проблемы в течение последних 6 месяцев?** | Нет | Да | Затрудняюсь ответить | Отказ от ответа |
| --- | --- | --- | --- | --- |
| **Вы перенесли тяжелое заболевания, травму, или физическое насилие?** | 2 | 1 | 97 | 98 |
| **Кто-то из Ваших близких перенес тяжелое заболевания, травму, или физическое насилие?** | 2 | 1 | 97 | 98 |
| **Умер кто-то из Ваших родителей, ребенок, супруг (а), проживающий (ая) в гражданском браке** | 2 | 1 | 97 | 98 |
| **Умер кто-то близкий Вашей семье: друг,** **другой родственник (тётя, бабушка, двоюродный брат/сестра)** | 2 | 1 | 97 | 98 |
| **Вы расстались/пережили развод из-за семейных сложностей** | 2 | 1 | 97 | 98 |
| **Вы разорвали крепкие взаимоотношения** | 2 | 1 | 97 | 98 |
| **У Вас возникли серьeзные проблемы с близким другом, родственником, соседями** | 2 | 1 | 97 | 98 |
| **Вы стали безработным/ой или безрезультатно искали работу больше месяца** | 2 | 1 | 97 | 98 |
| **Вас уволили** | 2 | 1 | 97 | 98 |
| **У Вас возникли серьезные финансовые проблемы** | 2 | 1 | 97 | 98 |
| **У Вас были проблемы с полицией и Вы должны были предстать перед судом** | 2 | 1 | 97 | 98 |
| **У Вас украли что-то ценное или Вы потеряли что-то ценное для Вас** | 2 | 1 | 97 | 98 |

**Сейчас мы подходим к заключительному разделу нашего опроса. Вначале я задам Вам несколько вопросов о событиях, которые могли случиться с Вами и Вашими близкими за последние 6 месяцев.**

| **K2. Наносили ли Вам телесные повреждения в течение последнего года?** | |
| --- | --- |
| 1  2 | Да  Нет |
| 97  98 | Затрудняюсь ответить  Отказ от ответа |
|  |  |
|  |  |

| **Модуль X** | **ОБСТОЯТЕЛЬСТВА ИНТЕРВЬЮ** |
| --- | --- |

*Последние вопросы – об обстоятельствах проведения интервью. На них отвечает только сам интервьюер. Их зачитывать не надо.*

| **X1.** | **Как Вы оцениваете надежность ответов в данном интервью?** |
| --- | --- |
|  |  |
| 1 | Удовлетворительно |
| 2 | Не вполне удовлетворительно. Например, не на все вопросы следовали ответы, или возможно, небольшие перерывы влияли на качество ответов |
| 3 | Плохо. Например, на многие вопросы не было ответов, или, возможно, частые (постоянные) перерывы влияли на качество ответов. |
|  |  |
| **X2.** | **Присутствовал ли кто-либо из других людей (кроме респондента) в том же помещении во время проведения интервью?** |
|  |  |
| 1 | Да |
| 2 | Нет |
| X3. | **Были ли перерывы в ходе интервью?** |
| 1 2 | Да  Нет *Переход к X5* |
| X4. | Опишите причины перерывов и их длительность……………….. |
| X5. | **Любые другие комментарии, включая указания на вопросы, которые казались трудными респонденту**………………….. |
