# Supplementary material for: Know Your Heart: Rationale, design and conduct of a cross-sectional study of cardiovascular structure, function and risk factors in 4500 men and women aged 35-69 years from two Russian cities, 2015-18
Source: Wellcome Open Res. 2018 Dec 3;3:67. Originally published 2018 Jun 4. [Version 3] doi: 10.12688/wellcomeopenres.14619.3 (PMC6073094; doi:10.12688/wellcomeopenres.14619.3)
Supplement: Supplementary file 3 [file wellcomeopenres-3-16294-s0002.tgz › d9a03f66-5fce-45a3-8a2e-d18febd22997_Supplementary_file_3._Questionnaires_and_data_collection_tools.zip!/questionnaires_181121_publish_version2/181118_HC_proforma_survey_EN.docm]

|  |  |
| --- | --- |

**Health Examination Questionnaire and Pro Forma**

International Project on Cardiovascular Disease in Russia

2015-2018

Published version 2.0 November 2018

Russia has one of the highest rates of cardiovascular disease in the world. The International Project on Cardiovascular Disease in Russia (IPCDR) was set up to understand the reasons for this. A substantial component of this study was the Know Your Heart Study devoted to characterising the nature and causes of cardiovascular disease in Russia by conducting large cross-sectional surveys in two Russian cities Novosibirsk and Arkhangelsk. The study population was 4542 men and women aged 35-69 years recruited from the general population. Fieldwork took place between 2015-18. There were two study components: 1) a baseline interview to collect information on socio-demographic characteristics and cardiovascular risk factors, usually conducted at home, and 2) a comprehensive health check at a primary care clinic which included detailed examination of the cardiovascular system.

The International Project on Cardiovascular Disease in Russia (IPCDR) project was funded in part by a Wellcome Trust Strategic Award [100217]. The project was also funded by the Arctic University of Norway, UiT in Tromsø; Norwegian Institute of Public Health; the Norwegian Ministry of Health and Social Affairs.

Development of the questionnaire was led by the London School of Hygiene & Tropical Medicine in collaboration with the Northern State Medical University, Arkhangelsk, Novosibirsk State Medical University, the Research Institute of Internal and Preventive Medicine - Branch of IC&G SB RAS, Novosibirsk and colleagues from other institutions.

This is a paper version of the questionnaire and proforma used for data collection although all data were collected electronically using data capture created using SURVANT (NETELIXIS IT SOLUTIONS 2015. SURVANT survey authoring and data collection software: version 1.0. <http://www.survant.net>.)

Further details about the study are available here: Know Your Heart: Rationale, design and conduct of a cross-sectional study of cardiovascular structure, function and risk factors in 4500 men and women aged 35-69 years from two Russian cities, 2015-18
Cook S, Malyutina S, Kudryavtsev AV, Averina M, Bobrova N, Boytsov S, Brage S, Clark T *et al.*

Content

[Module J. Registration/Last meal	4](#_Toc512505991)

[Module K. Blood pressure measurement	5](#_Toc512505992)

[Module R. Blood sample collection.	7](#_Toc512505994)

[Module A. Cardiology history	9](#_Toc512505995)

[Module B. Angina.	11](#_Toc512505996)

[Module C. Medications.	14](#_Toc512505996)

[Module D. General health	18](#_Toc512505997)

[Module E. Respiratory	19](#_Toc512505999)

[Module F. Other diseases	22](#_Toc512506001)

Module G. Questions for women	24

Module H. Alcohol consumption	27

[Module I. [Smoking	30](#_Toc512506003)](#_Toc512506003)

[[Module L. Anthropometry and body composition	31](#_Toc512506003)](#_Toc512506009)

[Module O. ECG and Vicoder	33](#_Toc512506003)

[Module O3. Actiheart	36](#_Toc512506009)

[Module P4. [Actiheart collection	38](#_Toc512506003)](#_Toc512506003)

[[Module M. Physical functioning tests	39](#_Toc512506003)](#_Toc512506009)

[Module P. ECHO and vascular ultrasound	42](#_Toc512506003)

[Module S. Spirometry	44](#_Toc512506009)

| **Module J** | **REGISTRATION/LAST MEAL** |
| --- | --- |

| **J1.**  **J2**. | **When was the last time that you ate or drank, apart from water?**  «____»___________20___y (DATE)  **Record time of last meal.** *Please use 24 hour clock*  _______:_______ |
| --- | --- |
| **J3.** | **What did you eat?** |
|  | Snack 1  A full meal 2  Only drink 3 |
|  | Difficult to answer 97 |
|  | Refuse to answer 98 |

*Stress the importance of accuracy to the participant.* **Now I will ask you some questions about your food and drink today. This can affect the results of some tests so it is important to be as accurate as possible.**

| **J4.** | **Did you drink anything during the previous 4 hours?** | | |
| --- | --- | --- | --- |
|  | Yes | 1 |  |
|  | No | 2 | *Go to J6* |
|  | Difficult to answer | 97 | *Go to J6* |
|  | Refuse to answer | 98 | *Go to J6* |
|  |  |  |  |
| **J5.** | **What did you drink? (Please indicate all that apply)** | | |
|  |  | | |
|  | Any alcoholic drinks | 1 |  |
|  | Coffee, black tea, coca-cola, pepsi-cola,any drinks containing caffeine | 2 |  |
|  | Other | 3 |  |
|  | Have not been drinking during the previous 4 hours | 4 |  |
|  | Difficult to answer | 97 |  |
|  | Refuse to answer | 98 |  |
|  |  |  |  |
| **J6.** | **Have you consumed alcohol drinks within the last 24 hours before the medical examination?** | | |
|  | Yes | 1 |  |
|  | No | 2 |  |
|  | Difficult to answer | 97 |  |
|  | Refuse to answer | 98 |  |
|  |  |  |  |

| **Module K** | ***BLOOD PRESSURE MEASUREMENT*** |
| --- | --- |

**I am now going to measure your blood pressure.** *Stress the importance of accuracy to the participant*

| **K1.** | **Room temperature (to nearest 0.1ºC)** | | | | | | | | | |
| --- | --- | --- | --- | --- | --- | --- | --- | --- | --- | --- |
|  |  | | |  |  | | **,** |  | ºС | |
|  | No measurement could be obtained | | | | 98 | | | | | |
| **Blood Pressure Measurements Instructions***.*  1. Plug cable a into laptop  2. Start Blood Pressure software  3. Make three measurements leaving 2 minutes between each measurement.  4. Export measurement (to CSV) | | | | | | | | | | |
| **K5**. | **File collection for Blood Pressure measurement**  *Before continuing make sure the measurement has been taken successfully and participant data has been exported. Once ready, press Next to proceed and collect the data into the system.* | | | | | | | | | |
| **K5a.** | **Interviewer-Blood Pressure Measurement successful, data exported and collected?** | | | | | | | | | |
|  | Yes | | | 1 | | | | | | |
|  | No | | | 2 *Go to K5b* | | | | | | |
| **K2.** | *Record which arm was used for measuring Blood Pressure.*  **The arm for measuring blood pressure was…** | | | | | | | | | |
|  |  | | | | | | | | | |
|  | Right | | | 1 | | |  | | | |
|  | Left | | | 2 | | |  | | | |
| **K3.** | **Arm circumference (to nearest 0.1 cm)*** | | | | | | | | | |
|  |  | |  | |  | | **,** |  | сm | |
|  |  | | | |  | | | | | |
|  | No measurement could be obtained | | | | 98 | | | | | |
| **K4.** |  | | | |  | | | | | |
|  | **Blood pressure cuff used** | | | | |  | | | | |
|  | Small | | | | | 1 | | | | |
|  | Medium | | | | | 2 | | | | |
|  | Large | | | | | 3 | | | | |
| **K5b**. | **Why Blood Pressure measurement was not successful?**   1. Participant refused 2. Not applicable to participant 3. Couldn't export data 4. Couldn't collect data 5. Other *Go to K5c* | | | | | | | | | |
| **K5c.** | **Please explain the reason why Blood Pressure measurement wasn’t successful**____ | | | | | | | | | |
| **K5d.** | **Please enter any comments you wish to enter on blood pressure section (for example if blood pressure was very high and needed to postpone the examination).** | | | | | | | | | |
|  |  |  | |  |  | |  | | |  |
| **K14.** | **Oxygen saturation** |  | |  | % | |  | | |  |

| **Module R** | **BLOOD SAMPLE COLLECTION** |
| --- | --- |

| **T2_a.** | **Room temperature (to nearest 0.1ºC)** | | | | | |
| --- | --- | --- | --- | --- | --- | --- |
|  |  |  |  | , |  | ºС |
|  | No measurement could be obtained | | 98 | | | |

| **R1.** | **What was the blood sample body drawing site?** | | | | | | | |  |
| --- | --- | --- | --- | --- | --- | --- | --- | --- | --- |
|  | From right arm | | | 1 | | *Go to R3* | | |  |
|  | From left arm | | | 2 | | *Go to R3* | | |  |
|  | blood has not been drawn | | | 3 | | *Go to R3* | | |  |
|  | blood was drawn from the other body site | | | 4 | |  | | |  |
|  |  | | |  | |  | | |  |
| **R2.** | **Please, specify the blood sample body drawing site.** | | | | | | | |  |
|  | | | | | | | | |  |
| **R3.** | **Was SST vacutainer 1 (8,5 ml) filled (with gold top)?** | | | | | | | |  |
|  |  | | | | | | | |  |
|  | Yes, completely | | | 1 | |  | | |  |
|  | Yes, partially | | | 2 | |  | | |  |
|  | No | | | 3 | |  | | |  |
|  |  | | |  | |  | | |  |
| **R4.** | **Was SST vacutainer 2 (8,5 ml) filled (with gold top)?** | | | | | | | |  |
|  |  | | | | | | | |  |
|  | Yes, completely | 1 | | | | | | | |
|  | Yes, partially | 2 | | | | | | | |
|  | No | 3 | | | | | | | |
|  |  |  | | | | | | | |
| **R5.** | **Was SST vacutainer 3 (8,5 ml) filled (with gold top)?** | | | | | | | |  |
|  |  | | | | | | | |  |
|  | Yes, completely | | 1 | |  | | |  |  |
|  | Yes, partially | | 2 | |  | | |  |  |
|  | No | | 3 | |  | | |  |  |
|  |  | |  | |  | | |  |  |
| **R6.** | **Was SST vacutainer 4 (8,5 ml) filled (with gold top)?** | | | | | | |  |  |
|  |  | | | | | | |  |  |
|  | Yes, completely | | 1 | |  | | |  |  |
|  | Yes, partially | | 2 | |  | | |  |  |
|  | No | | 3 | |  | | |  |  |
|  |  | |  | |  | | |  |  |
| **R7.** | **Was vacutainer (10 ml) with EDTA 1 filled (purple top)** | | | | | | |  |  |
|  |  | |  | |  | | |  |  |
|  | Yes, completely | | 1 | |  | | |  |  |
|  | Yes, partially | | 2 | |  | | |  |  |
|  | No | | 3 | |  | | |  |  |
|  |  | |  | |  | | |  |  |
| **R8.** | **Was vacutainer (6 ml) with EDTA 2 filled (purple top)** | | | | | | |  |  |
|  |  | | | | | | |  |  |
|  | Yes, completely | | 1 | | | |  |  |  |
|  | Yes, partially | | 2 | | | |  |  |  |
|  | No | | 3 | | | |  |  |  |
| **R9.** | **Real time start:** _______:_______:_______ | | | | | | |  |  |

| **Module A** | **CARDIOLOGY HISTORY** |
| --- | --- |

|  | **A1.** | | | | **Have you ever had your blood pressure measured by a doctor or other medical professional?** | | | | | | |
| --- | --- | --- | --- | --- | --- | --- | --- | --- | --- | --- | --- |
|  | | | Yes | | | | 1 | | | | |
|  | | | No | | | | 2 *Go to A5* | | | | |
|  | | | Difficult to answer | | | | 97 | | | | |
|  | | | Refuse to answer | | | | 98 | | | | |
|  | | |  | | | |  | | | | |
| **A2.** | | | **Have you ever been told by a doctor or nurse that you have high blood pressure?** | | | | | | | | |
|  | | |  | | | | | | | | |
|  | | | Yes | | | | | 1 | | | |
|  | | | No | | | | | 2 *Go to A5* | | | |
|  | | | Difficult to answer | | | | | 97 | | | |
|  | | | Refuse to answer | | | | | 98 | | | |
|  | | |  | | | | |  | | | |
| **A3.** | | | **Has a doctor prescribed drugs to control your blood pressure?** | | | | | | | | |
|  | | |  | | | | | | | | |
|  | | | Yes | | | | | 1 | | | |
|  | | | No | | | | | 2 *Go to A5* | | | |
|  | | | Difficult to answer | | | | | 97 | | | |
|  | | | Refuse to answer | | | | | 98 | | | |
|  | | |  | | | | |  | | | |
| **A4.** | | | **Do you take these drugs?** | | | | |  | | | |
|  | | | Every day | | | | | 1 | | | |
|  | | | Only sometimes such as when I feel unwell | | | | | 2 | | | |
|  | | | Never | | | | | 3 | | | |
|  | | | Difficult to answer | | | | | 97 | | | |
|  | | | Refuse to answer | | | | | 98 | | | |
|  | | |  | | | | |  | | | |
|  | **A5.** | | | | **Have you ever been told by a doctor or nurse that you have diabetes mellitus?** | | | | | | |
|  |  | | | |  | | | | | | |
|  |  | | | | No | | | | 1 *Go to A8* | |  |
|  |  | | | | Yes, and I use insulin | | | | 2 | |  |
|  |  | | | | Yes, and I control it with drugs | | | | 3 | |  |
|  |  | | | | Yes, and I control it with diet only | | | | 4 | |  |
|  |  | | | | Difficult to answer | | | | 97 *Go to A8* | |  |
|  |  | | | | refuse to answer | | | | 98 *Go to A8* | |  |
|  |  | | | |  | | | |  | |  |
|  | **A6.** | | | | **What type of diabetes did the doctor say you had?** | | | | | | |
|  |  | | | |  | | | | | | |
|  |  | | | | Type 1 diabetes | | | | 1 | |  |
|  |  | | | | Type 2 diabetes | | | | 2 | |  |
|  |  | | | | Difficult to answer | | | | 97 | |  |
|  |  | | | | Refuse to answer | | | | 98 | |  |
|  |  | | | |  | | | |  | |  |
|  | **A7.** | | | | **At what age were you diagnosed with diabetes?**________ Years | | | | | | |
|  |  | | | |  | | | | |  | |
|  |  | | | | Difficult to answer | | | | | 97  Re | |
|  |  | | | | Refuse to answer | | | | | 98 | |
|  |  | | | |  | | | | | | |
|  | | **A8.** | | **Have you ever been told by a doctor that you have high cholesterol?** | | | | | | | |
|  | |  | |  | | | | | | | |
|  | |  | | | | Yes | | 1 | | | |
|  | |  | | | | No | | 2 | | | |
|  | |  | | | | Difficult to answer | | 97  Re | | | |
|  | |  | | | | Refuse to answer | | 98 | | | |

| **Module B** | **ANGINA** |
| --- | --- |

| **B1.** | | **Have you ever had any pain or discomfort in your chest?** | | | | | | |
| --- | --- | --- | --- | --- | --- | --- | --- | --- |
|  | |  | | | | | | |
|  | | Yes | | 1 | | |  |  |
|  | | No | | 2 *Go to B11* | | |  |  |
|  | | Difficult to answer | | 97 | | |  |  |
|  | | Refuse to answer | | 98 | | |  |  |
|  | |  | |  | | |  |  |
| **B2.** | | **Do you get this pain or discomfort in your chest when you walk uphill or hurry?** | | | | | | |
|  | |  | | | | | | |
|  | | Yes | | 1 | | |  |  |
|  | | No | | 2 *Go to B8* | | |  |  |
|  | | Never walk uphill or in a hurry | | 3 | | |  |  |
|  | | Difficult to answer | | 97 | | |  |  |
|  | | Refuse to answer | | 98 | | |  |  |
|  | |  | |  | | |  |  |
| **B3.** | | **Do you get this pain or discomfort when you walk at an ordinary pace on the level?** | | | | | | |
|  | |  | | | | | | |
|  | | Yes | | 1 | | |  |  |
|  | | No | | 2 | | |  |  |
|  | | Never walk at an ordinary pace on ground level | | 3 | | |  |  |
|  | | Difficult to answer | | 97 | | |  |  |
|  | | Refuse to answer | | 98 | | |  |  |
|  | |  | |  | | |  |  |
| **B4.** | | **When you get any pain or discomfort in your chest, what do you do?** | | | | | | |
|  | | Stop | | 1 | | |  |  |
|  | | Slow down | | 2 | | |  |  |
|  | | Continue at the same pace | | 3 | | |  |  |
|  | | Difficult to answer | | 97 | | |  |  |
|  | | Refuse to answer | | 98 | | |  |  |
|  | |  | |  | | |  |  |
| **B5.** | | **Does pain or discomfort in the chest go away when you stand still?** | | | | | | |
|  | | Yes | | 1 | | |  |  |
|  | | No | | 2 *Go to B7* | | |  |  |
|  | | Difficult to answer | | 97 *Go to B7* | | |  |  |
|  | | Refuse to answer | | 98 *Go to B7* | | |  |  |
|  | |  | |  | | |  |  |
| **B6.** | | **How soon does pain or discomfort in the chest go away?** | | | | | | |
|  | | 10 minutes or less | | 1 | | |  |  |
|  | | More than 10 minutes | | 2 | | |  |  |
|  | | Difficult to answer | | 97 | | |  |  |
|  | | Refuse to answer | | 98 | | |  |  |
|  |  | |  | |  | | |  |
| **B7** | **Where do you get this pain or discomfort?** | | | | | | |  |
|  |  | |  | |  | | |  |
|  |  |  |  |  |  | | |  |
|  |  |  |  |  |  |  |  |  |
|  |  |  |  |  |  |  |  |  |
|  |  |  |  |  |  |  |  |  |
|  |  |  |  |  |  |  |  |  |
|  |  |  |  |  |  |  |  |  |
|  |  |  |  |  |  |  |  |  |
|  |  |  |  |  |  | | |  |
|  |  | | | | | | |  |
| **B8.** | | **Have you ever had a severe pain across the front of your chest lasting for half an hour or more?** | | | | | | |
|  | | Yes | | 1 | |  | | |
|  | | No | | 2 | | *Go to B11* | | |
|  | | Difficult to answer | | 97 | | *Go to B11* | | |
|  | | Refuse to answer | | 98 | | *Go to B11* | | |
|  | |  | |  | |  | | |
| **B9.** | | **Did you see a doctor because of this pain?** | | | | | | |
|  | | Yes | | 1 | |  | | |
|  | | No | | 2 | | *Go to B11* | | |
|  | | Difficult to answer | | 97 | | *Go to B11* | | |
|  | | Refuse to answer | | 98 | | *Go to B11* | | |
|  | |  | |  | |  | | |
| **B10.** | | **What did he or she say it was?** | | | | | | |
|  | |  | | | | | | |
|  | | Angina (ischemic heart disease) | | 1 | |  | | |
|  | | Myocardial infarction | | 2 | |  | | |
|  | | Something else | | 3 | |  | | |
|  | | Did not say | | 4 | |  | | |
|  | | Difficult to answer | | 97 | |  | | |
|  | | Refuse to answer | | 98 | |  | | |
|  | |  | |  | |  | | |
| **B11.** | | **Have you ever been told by a doctor that you have/had or been diagnosed with following diseases:** | | | | | | |

| **B11_1. Myocardial infarction/heart attack** |  |  | |
| --- | --- | --- | --- |
| Yes (and I was hospitalised) | 1 |  | |
| Yes (but I was not hospitalised) | 2 |  | |
| No | 3 |  | |
| Difficult to answer | 97 |  | |
| Refuse to answer | 98 |  | |
| **B11_2.** **Angina/ Ischemic Heart Disease** |  |  | |
|  |  |  | |
| Yes (and I was hospitalised) | 1 |  | |
| Yes (but I was not hospitalised) | 2 |  | |
| No | 3 |  | |
| Difficult to answer | 97 |  | |
| Refuse to answer | 98 |  | |
|  |  |  | |
| **B11_3. Cerebral stroke** |  |  | |
| Yes (and I was hospitalised) | 1 |  | |
| Yes (but I was not hospitalised) | 2 |  | |
| No | 3 |  | |
| Difficult to answer | 97 |  | |
| Refuse to answer | 98 |  | |
|  |  |  | |
| **B11_4. Operations/interventions on the heart** |  |  | |
|  |  |  | |
| Yes (and I was hospitalised) | 1 *Go to B11_4_4a* |  | |
| Yes (but I was not hospitalised) | 2 *Go to B11_4_4a* | |  |
| No | 3 *Go to B11_5* |  | |
| Difficult to answer | 97 |  | |
| Refuse to answer | 98 |  | |
|  |  |  | |
| **B11_4_4a. What operation did you have (choose all that apply)** |  |  | |
|  |  |  | |
| Coronary artery bypass graft | 1 |  | |
| Valve replacement | 2 |  | |
| Implantation of stent in coronary arteries | 3 |  | |
| Other | 4 |  | |
| Difficult to answer | 97 |  | |
| Refuse to answer | 98 |  | |
| **B11_5. Chronic Kidney Disease** |  |  | |
|  |  |  | |
| Yes (and I was hospitalised) | 1 |  | |
| Yes (but I was not hospitalised) | 2 |  | |
| No | 3 |  | |
| Difficult to answer | 97 |  | |
| Refuse to answer | 98 |  | |
| **B11_6. Chronic disease of bronchi and lungs** |  |  | |
| Yes (and I was hospitalised) | 1 |  | |
| Yes (but I was not hospitalised) | 2 |  | |
| No | 3 |  | |
| Difficult to answer | 97 |  | |
| Refuse to answer | 98 |  | |

| **Module C** | **MEDICATIONS USE** |
| --- | --- |

| **C1.** | **Do you currently use any medications including inhalers?** | | |
| --- | --- | --- | --- |
|  | Yes | 1 |  |
|  | No | 2 | *Go to D1* |

**Please, show the packaging of used medications. If there is no package, what are the names of used medications? What are the doses and frequency of used medications? Please include any inhalers.** *Interviewer! Please, register up to 7 used medications. Dose of medication refers to strength of medication e.g. mg per tablet. Don’t register ointments, creams, herbal infusions and food supplements.*

|  | Name of the medication | Dose  (e.g. mg) | Reason for taking |  | Number of units of medication used during  one take | | | | Frequency of use of medication | | |  |
| --- | --- | --- | --- | --- | --- | --- | --- | --- | --- | --- | --- | --- |
|  |  |  |  |  |  |  |  | tablets / capsules/inhalers |  |  |  | *Codes for frequency of use of medications:*  01 daily 3 times a day  02 daily 2 times a day  03 daily once a day  04 3 times a week  05 2 times a week  06 once a week  07 fewer than once a week  08 not regularly, only when needed  97 difficult to answer  98 Refuse to answer |
| **C2.** |  |  |  |  |  |  |  | ml - injections |  |  |  |  |
|  |  |  |  |  |  |  |  | insulin units |  |  |  |  |
|  |  |  |  |  |  |  |  | drops |  |  |  |  |
|  |  |  |  |  |  |  |  |  |  |  |  |  |
|  |  |  |  |  |  |  |  | tablets / capsules/inhalers |  |  |  |  |
| **C3.** |  |  |  |  |  |  |  | ml - injections |  |  |  |  |
|  |  |  |  |  |  |  |  | insulin units |  |  |  |  |
|  |  |  |  |  |  |  |  | drops |  |  |  |  |
|  |  |  |  |  |  |  |  |  |  |  |  |  |
|  |  |  |  |  |  |  |  | tablets / capsules/inhalers |  |  |  |  |
| **C4.** |  |  |  |  |  |  |  | ml - injections |  |  |  |  |
|  |  |  |  |  |  |  |  | insulin units |  |  |  |  |
|  |  |  |  |  |  |  |  | drops |  |  |  |  |
|  | Name of the medication | Dose  (e.g. mg) | Reason for taking |  | Number of units of medication used during  one take | | | | Frequency of use of medication | | |  |
|  |  |  |  |  |  |  |  | tablets / capsules/inhalers |  |  |  | *Codes for frequency of use of medications:*  01 daily 3 times a day  02 daily 2 times a day  03 daily once a day  04 3 times a week  05 2 times a week  06 once a week  07 fewer than once a week  08 not regularly, only when needed  97 difficult to answer  98 Refuse to answer |
| **C5.** |  |  |  |  |  |  |  | ml - injections |  |  |  |  |
|  |  |  |  |  |  |  |  | insulin units |  |  |  |  |
|  |  |  |  |  |  |  |  | drops |  |  |  |  |
|  |  |  |  |  |  |  |  |  |  |  |  |  |
|  |  |  |  |  |  |  |  | tablets / capsules/inhalers |  |  |  |  |
| **C6.** |  |  |  |  |  |  |  | ml - injections |  |  |  |  |
|  |  |  |  |  |  |  |  | insulin units |  |  |  |  |
|  |  |  |  |  |  |  |  | drops |  |  |  |  |
|  |  |  |  |  |  |  |  |  |  |  |  |  |
|  |  |  |  |  |  |  |  | tablets / capsules/inhalers |  |  |  |  |
| **C7.** |  |  |  |  |  |  |  | ml - injections |  |  |  |  |
|  |  |  |  |  |  |  |  | insulin units |  |  |  |  |
|  |  |  |  |  |  |  |  | drops |  |  |  |  |

| **C9.** | **Record whether medications were seen** | |
| --- | --- | --- |
|  | Medications seen | 1 |
|  | Medications not seen, self-report only | 2 |

**C10. Please enter any comments you want to record on the cardiology questionnaire (for example participant had difficulty hearing and needed to see the screen).**

| **Module D** | **GENERAL HEALTH** |
| --- | --- |

| **D1.** | **How do you rate your own health in general?** | | |
| --- | --- | --- | --- |
|  |  |  | |
|  | Very good | 1 |  |
|  | Good | 2 |  |
|  | Neither good, poor or bad | 3 |  |
|  | Poor | 4 |  |
|  | Very poor | 5 |  |
|  | Difficult to answer | 97 |  |
|  | Refuse to answer | 98 |  |

| **Мodule E** | **RESPIRATORY** |
| --- | --- |

|  |  | **I now want to ask you some questions about your breathing** | | | |
| --- | --- | --- | --- | --- | --- |
|  | **E1.** | **Do you usually cough first thing in the morning in winter?** *Stress the word “usually” to the participant.* | | | |
|  |  | Yes | | 1 |  |
|  |  | No | | 2 |  |
|  |  | Difficult to answer | | 97 |  |
|  |  | Refuse to answer | | 98 |  |
|  |  |  | |  |  |
|  | **E2.** | **Do you usually cough during the day or at night in winter?** *Stress the word “usually” to the participant.* | | | |
|  |  | Yes | | 1 |  |
|  |  | No | | 2 |  |
|  |  | Difficult to answer | | 97 |  |
|  |  | Refuse to answer | | 98 |  |
|  |  |  | |  |  |
|  | **E3.** | *If participant answered NO to E1 AND E2 do not ask this question* **Do you cough like this on most days for as much as three months each year?** | | | |
|  |  | Yes | | 1 |  |
|  |  | No | | 2 |  |
|  |  | Difficult to answer | | 97 |  |
|  |  | Refuse to answer | | 98 |  |
|  |  |  | |  |  |
|  | **E4.** | **Do you usually bring up phlegm from your chest first thing in the morning in winter?** *Stress the word “usually” to the participant.* | | | |
|  |  | Yes | | 1 |  |
|  |  | No | | 2 |  |
|  |  | Difficult to answer | | 97 |  |
|  |  | Refuse to answer | | 98 |  |
|  |  |  | |  |  |
|  | **E5.** | **Do you usually bring up any phlegm from your chest during the day- or at night – in winter?** *Stress the word “usually” to the participant* | | | |
|  |  | Yes | | 1 |  |
|  |  | No | | 2 |  |
|  |  | Difficult to answer | | 97 |  |
|  |  | Refuse to answer | | 98 |  |
|  |  |  | |  |  |
|  | **E6.** | *If participant answered NO to E4 AND E5 do not ask this question* **Do you bring up phlegm like this on most days for as much as three months each year?** | | | |
|  |  | Yes | | 1 |  |
|  |  | No | | 2 |  |
|  |  | Difficult to answer | | 97  98 |  |
|  |  | Refuse to answer | | 98 |  |
|  |  |  | |  |  |
|  | **E7.** | **Are you troubled by shortness of breath when hurrying on level ground or walking up a slight hill?** | | | |
|  |  | Yes | | 1 |  |
|  |  | No | | 2 | *Go to E12* |
|  |  | Difficult to answer | | 97 |  |
|  |  | Refuse to answer | | 98 |  |
|  |  |  | |  |  |
| **E8.** | | | **Do you get short of breath walking with people of your own age group on level ground?** | | |
|  | | | Yes | 1 |  |
|  | | | No | 2 |  |
|  | | | Difficult to answer | 97 |  |
|  | | | Refuse to answer | 98 |  |
|  | | |  |  |  |
| **E9.** | | | **Do you have to stop for breath when walking at your own pace on level ground?** | | |
|  | | |  | | |
|  | | | Yes | 1 |  |
|  | | | No | 2 | *Go to E12* |
|  | | | Never walks at own pace on level ground | 3 |  |
|  | | | Difficult to answer | 97 |  |
|  | | | Refuse to answer | 98 |  |
|  | | |  |  |  |
| **E10.** | | | **Do you have to stop for breath when walking about 100m or for a few minutes on level ground?** | | |
|  | | |  | | |
|  | | | Yes | 1 |  |
|  | | | No | 2 | *Go to E12* |
|  | | | Never walks 100m | 3 |  |
|  | | | Difficult to answer | 97 |  |
|  | | | Refuse to answer | 98 |  |
|  | | |  |  |  |
| **E11.** | | | **Do you get breathless while undressing or feel too breathless to leave your house?** | | |
|  | | |  | | |
|  | | | Yes | 1 |  |
|  | | | No | 2 |  |
|  | | | Difficult to answer | 97 |  |
|  | | | Refuse to answer | 98 |  |
|  | | |  |  |  |
| **E12.** | | | **Have you had attacks of wheezing or whistling in your chest at any time in the last 12 months?** | | |
|  | | | Yes | 1 |  |
|  | | | No | 2 |  |
|  | | | Difficult to answer | 97 |  |
|  | | | Refuse to answer | 98 |  |
|  | | |  |  |  |
| **E13.** | | | **Have you at any time in the past 12 months been woken at night by an attack of shortness of breath?** | | |
|  | | | Yes | 1 |  |
|  | | | No | 2 |  |
|  | | | Difficult to answer | 97 |  |
|  | | | Refuse to answer | 98 |  |

| **Мodule F** | **OTHER DISEASES** |
| --- | --- |

|  | **I now want to ask you about problems with other body systems.** | | | | |
| --- | --- | --- | --- | --- | --- |
| **F1.** | **Do you have any diseases of the liver diagnosed by a physician?** | | | | |
|  | Yes | | 1 |  | |
|  | No | | 2 | *Go to F 4* | |
|  | Difficult to answer | | 97 | *Go to F 4* | |
|  | Refuse to answer | | 98 | *Go to F 4* | |
|  |  | |  |  | |
| **F2.** | **What are these diseases of liver?** *Please select all that apply.* | | | | |
|  | *Interviewer – if participant reports jaundice but no diagnosis code as “other”.* | | | | |
|  | Hepatitis | | 1 | |  |
|  | Fatty liver disease | | 2 |  | |
|  | Cirrhosis | | 3 |  | |
|  | Liver cancer | | 4 |  | |
|  | Other | | 5 |  | |
|  | Difficult to answer | | 97 |  | |
|  | Refuse to answer | | 98 |  | |
|  |  | |  |  | |
| **F3.** | **What did the physician tell you had caused your liver disease?** *Please select all that apply.* | | | | |
|  | Alcohol | 1 | |  | |
|  | Being overweight | 2 | |  | |
|  | Virus | 3 | |  | |
|  | Other | 4 | |  | |
|  | Difficult to answer | 97 | |  | |
|  | Refuse to answer | 98 | |  | |
|  |  |  | |  | |
| **F4.** | **Have you ever been diagnosed with cancer?** | | | | |
|  | Yes | | 1 |  | |
|  | No | | 2 | *Go to F6* | |
|  | Difficult to answer | | 97 | *Go to F6* | |
|  | Refuse to answer | | 98 | *Go to F6* | |

| **F5.** | **Please type in what part of the body was affected________________** | | |
| --- | --- | --- | --- |
|  |  |  |  |
|  | Difficult to answer | 97 |  |
|  | Refuse to answer | 98 |  |
|  |  |  |  |
| **F6.** | **During the past 12 months, have you ever gone to a doctor with any traumas / injuries?** *Interviewer! Here only physical, but not psychological traumas are assumed.* | | |
|  | Yes | 1 |  |
|  | No | 2 | *Go to section G for women or to section H for men* |
|  | Difficult to answer | 97 | *Go to section G for women or to section H for men* |
|  | Refuse to answer | 98 | *Go to section G for women or to section H for men* |
|  |  |  |  |
| **F7.** | **What were these traumas / injuries?** *Please select all that apply.* | | |
|  | Fracture/broken bone | 1 |  |
|  | Bruising, sprain, dislocation | 2 |  |
|  | Cut/Open wound/ Bite | 3 |  |
|  | Concussion | 4 |  |
|  | Other | 5 |  |
|  | Difficult to answer | 97 |  |
|  | Refuse to answer | 98 |  |

| **Мodule G** | **QUESTIONS ONLY FOR WOMEN** |
| --- | --- |

*Please establish the gender of the participant as it will determine which questions will be asked next (the section following is for female participants only and will be skipped for male participants). If participant is male go to Section H.*

| **G1.** | **Have you ever been pregnant?** | | | | | |
| --- | --- | --- | --- | --- | --- | --- |
|  | Yes | | 1 | | | |
|  | No | | 2 *Go to G6* | | | |
|  | Difficult to answer | | 97 | | | |
|  | Refuse to answer | | 98 | | | |
|  |  | |  | | | |
| **G2.** | **How many pregnancies did you have?** *Enter number of times pregnant.* | | | | | |
|  |  | |  | | | |
|  |  | |  | | | |
|  |  | |  | | | |
|  | Difficult to answer | | | | | 97 |
|  | Refuse to answer | | | | | 98 |
|  |  | | | | |  |
| **G3.** | **In total during your life, how many of the following have you had?** | | | | | |
|  |  | |  | | | |
|  | Live births | |  | | | |
|  |  | |  | | | |
|  | Still births (28 weeks+) | |  | | | |
|  |  | |  | | | |
|  |  | |  | | | |
|  | Miscarriages (less than 14 weeks) | |  | | | |
|  |  | |  | | | |
|  | Miscarriages (14-27 weeks) | |  | | | |
|  |  | |  | | | |
|  | Difficult to answer | | | | | 97 |
|  | Refuse to answer | | | | | 98 |
|  |  | | | | |  |
| **G4.** | **Are you currently pregnant, or think you may be pregnant?** | | | | | |
|  | Yes | | 1 | | | |
|  | No | | 2 | | | |
|  | Difficult to answer | | 97 | | | |
|  | Refuse to answer | | 98 | | | |
|  |  | |  | | | |
| **G5a.** | **During any of your past pregnancies or your current pregnancy, have you ever been told by a doctor you had/have high blood pressure in pregnancy (Hypertension in pregnancy)?** | | | | | |
|  | Yes | | 1 | | | |
|  | No | | 2 | | | |
|  | Difficult to answer | | 97 | | | |
|  | Refuse to answer | | 98 | | | |
|  |  | |  | | | |
| **G5b.** | **During any of your past pregnancies or your current pregnancy, have you ever been told by a doctor you had/have Gestational Diabetes revealed during pregnancy?** | | | | | |
|  | Yes | | 1 | | | |
|  | No | | 2 | | | |
|  | Difficult to answer | | 97 | | | |
|  | Refuse to answer | | 98 | | | |
|  |  | |  | | | |
| **G5c.** | **During any of your past pregnancies or your current pregnancy, have you ever been told by a doctor you had/have Pre-eclampsia?** *Pre-eclampsia - a complication during pregnancy, the patient shows a significant increase in blood pressure, swelling and appearance of seizures with loss of consciousness.* | | | | | |
|  | Yes | | 1 | | | |
|  | No | | 2 | | | |
|  | Difficult to answer | | 97 | | | |
|  | Refuse to answer | | 98 | | | |
|  |  | |  | | | |
| **G6.** | **About how old were you when you had your first menstrual period?** | | | | | |
|  |  | |  | | | |
|  |  | | Years | | | |
|  |  | |  | | | |
| Difficult to answer | | | | | 97 | |
| Refuse to answer | | | | | 98 | |
|  | | | | |  | |
| **G7.** | **In the past 12 months have you had a period or menstrual bleeding?** | | | | | |
|  |  | |  | | | |
|  | Yes | | 1 *Go to G9* | | | |
|  | No | | 2 | | | |
|  | Difficult to answer | | 97 | | | |
|  | Refuse to answer | | 98 | | | |
|  |  | |  | | | |
| **G8.** | **If you have not had a period in the last 12 months were your periods stopped by:** | | | | | |
|  | Surgery | | | 1 | | |
|  | Chemotherapy or radiation therapy | | | 2 | | |
|  | Pregnancy or breastfeeding | | | 3 | | |
|  | Natural stop/ menopause | | | 4 | | |
|  | Other | | | 5 | | |
|  | Difficult to answer | | | 97 | | |
|  | Refuse to answer | | | 98 | | |
|  |  | | |  | | |
| **G9.** | **When was your last period?** | | | | | |
|  | Year  Month | |  | | | |
|  |  | |  | | | |
|  |  | |  | | | |
|  | Difficult to answer | | | | | 97 |
|  | Refuse to answer | | | | | 98 |
|  |  | |  | | | |
| **G9a.** | **If you cannot remember the month and year please give your age at the time.** *______*Years. | | | | | |
|  | Difficult to answer | | 97 | | | |
|  | Refuse to answer | | 98 | | | |
|  |  | |  | | | |
| **G10.** | **Have you ever taken Hormone Replacement Therapy (HRT)?** | | | | | |
|  | *Interviewer – this question does not refer to hormones used for contraception. You should clarify this for participants if they ask* | | | | | |
|  |  |  | | | | |
|  | Yes | 1 | | | | |
|  | No | 2 *Go to Section H* | | | | |
|  | Difficult to answer | 97 | | | | |
|  | Refuse to answer | 98 | | | | |
|  |  |  | | | | |
| **G11.** | **When did you first start HRT (hormone replacement therapy)?** | | | | | |
|  | Year  Month |  | | | | |
|  |  |  | | | | |
|  | Difficult to answer | 97 | | | | |
|  | Refuse to answer | 98 | | | | |
|  |  |  | | | | |
| **G11a.** | **If you cannot remember the month and year please give your age at the time.** *Please enter age in years.*  Years  Years | | | | | |
|  |  | | | | | |
|  |  | | | | | |
|  | Difficult to answer | | | | | 97 |
|  | Refuse to answer | | | | | 98 |
|  |  | | | | | |
| **G12.** | **Are you currently on HRT (hormone replacement therapy)?** | | | | | |
|  |  | |  | | | |
|  | Yes | | 1 *Go to Section H* | | | |
|  | No | | 2 | | | |
|  | Difficult to answer | | 97 | | | |
|  | Refuse to answer | | 98 | | | |
|  |  | |  | | | |
| **G13.** | **When did you stop taking HRT (hormone replacement therapy)?** | | | | | |
|  |  | | | | | |
|  | Year  Month |  | | | | |
|  |  |  | | | | |
|  | Difficult to answer | 97 | | | | |
|  | Refuse to answer | 98 | | | | |
|  |  |  | | | | |
|  |  |  | | | | |
| **G13a.** | **If you cannot remember the month and year please give your age at the time.** *Please enter age in years.*  Years | | | | | |
|  |  | | | | | |
|  |  | | | | | |
|  | Difficult to answer | | | | | 97 |
|  | Refuse to answer | | | | | 98 |
| **Мodule H** | | | | | | **ALCOHOL CONSUMPTION** |

**Now I am going to ask you some questions about your use of alcoholic beverages in the past year. Because alcohol use can affect many areas of health (and may interfere with certain medications), it is important for us that you will be as accurate as you can be.**

**In this section we ask about drinks or ‘portions’. One drink/portion of alcohol is 10 g ethanol and is equal to:** *SHOW FLASH CARD*

**-250 ml non-strong beer (4-6% ABV)**

**-150 ml strong beer (7-9% ABV)**

**-100 ml normal/table wine**

**- 60 ml fortified wine**

**-30 ml vodka or other strong spirit**

| **H1.** | **How often do you have a drink containing alcohol?** | | |
| --- | --- | --- | --- |
|  | Never | | 1 *Go to H9* |
|  | Monthly or less | | 2 |
|  | 2-4 times per month | | 3 |
|  | 2-3 times per week | | 4 |
|  | 4 or more times per week | | 5 |
|  | Difficult to answer | | 97 |
|  | Refuse to answer | | 98 |
|  |  | |  |
| **H2.** | **How many drinks (portions) containing alcohol do you have on a typical day when you are drinking?** | | |
|  | 1 or 2 | | 1 |
|  | 3 or 4 | | 2 |
|  | 5 or 6 | | 3 |
|  | 7, 8 or 9 | | 4 |
|  | 10 or more | | 5 |
|  | Difficult to answer | | 97 |
|  | Refuse to answer | | 98 |
| **H3.** | **How often do you have six or more drinks on one occasion?**  *Interviewer - Remind participant 6 portions is equal to approximately 3 x 500ml cans of beer, 3 large (200ml) glasses of wine or 180ml of vodka (3.5 x 50ml glasses).* | | |
|  | Never | | 1 |
|  | Less than monthly | | 2 |
|  | Monthly | | 3 |
|  | Weekly | | 4 |
|  | Daily or almost daily | | 5 |
|  | Difficult to answer | | 97 |
|  | Refuse to answer | | 98 |
|  |  | |  |
| **H4.** | **How often during the last year have you found that you were not able to stop drinking once you had started?** | | |
|  | Never | | 1 |
|  | Less than monthly | | 2 |
|  | Monthly | | 3 |
|  | Weekly | | 4 |
|  | Daily or almost daily | | 5 |
|  | Difficult to answer | | 97 |
|  | Refuse to answer | | 98 |
|  |  | |  |
| **H5.** | **How often during the last year have you failed to do what was normally expected of you because of drinking?** | | |
|  | Never | | 1 |
|  | Less than monthly | | 2 |
|  | Monthly | | 3 |
|  | Weekly | | 4 |
|  | Daily or almost daily | | 5 |
|  | Difficult to answer | | 97 |
|  | Refuse to answer | | 98 |
|  |  | |  |
| **H6.** | **How often during the last year have you needed a drink first thing in the morning to get yourself going after a heavy drinking session?** | | |
|  | Never | | 1 |
|  | Less than monthly | | 2 |
|  | Monthly | | 3 |
|  | Weekly | | 4 |
|  | Daily or almost daily | | 5 |
|  | Difficult to answer | | 97 |
|  | Refuse to answer | | 98 |
| **H7.** | **How often during the last year have you had a feeling of guilt or remorse as a result of your drinking?** | | |
|  | Never | 1 | |
|  | Less than monthly | 2 | |
|  | Monthly | 3 | |
|  | Weekly | 4 | |
|  | Daily or almost daily | 5 | |
|  | Difficult to answer | 97 | |
|  | Refuse to answer | 98 | |
| **H8.** | **How often during the last year have you been unable to remember what happened the night before because of your drinking?** | | |
|  |  | | |
|  | Never | 1 | |
|  | Less than monthly | 2 | |
|  | Monthly | 3 | |
|  | Weekly | 4 | |
|  | Daily or almost daily | 5 | |
|  | Difficult to answer | 97 | |
|  | Refuse to answer | 98 | |
|  |  |  | |
| **H9.** | **Have you or someone else been injured because of your drinking?** | | |
|  | No | 1 | |
|  | Yes, but not in the last year | 2 | |
|  | Yes, during the last year | 3 | |
|  | Difficult to answer | 97 | |
|  | Refuse to answer | 98 | |
|  |  |  | |
| **H10.** | **Has a relative or friend or a doctor or another health worker been concerned about your drinking or suggested you cut down?** | | |
|  | No | 1 | |
|  | Yes, but not in the last year | 2 | |
|  | Yes, during the last year | 3 | |
|  | Difficult to answer | 97 | |
|  | Refuse to answer | 98 | |

| **Мodule I** | **SMOKING** |
| --- | --- |

**Now I ask you some questions about smoking.**

| **I1.** | **Are you a current smoker?** | | |
| --- | --- | --- | --- |
|  | Never smoked | 1 | *Go to J1* |
|  | No, ex-smoker | 2 |  |
|  | Yes, a regular smoker | 3 | *Go to I3* |
|  | Yes, I smoke but less that 1 cigarette per day | 4 | *Go to I3* |
|  | Difficult to answer | 97 | *Go to I3* |
|  | Refuse to answer | 98 | *Go to J1* |
|  |  |  |  |
| **I2.** | **How many years ago did you stop smoking regularly?** *Please choose one answer*. | | |
|  | Up to one year ago | 1 |  |
|  | More than 1, up to 5 years ago | 2 |  |
|  | More than 5, up to 10 years ago | 3 |  |
|  | More than 10 years ago | 4 |  |
|  | Difficult to answer | 97 |  |
|  | Refuse to answer | 98 | *Go to J1* |
|  |  |  |  |
| **I3.** | **What do you smoke most often?** *Please choose one answer*. | | |
|  | Cigarettes (papirosy) | 1 |  |
|  | Filtered cigarattes | 2 |  |
|  | Unfiltered cigarattes | 3 |  |
|  | Other | 4 |  |
|  | Other. Specify | 4a |  |
|  | Difficult to answer | 97 |  |
|  | Refuse to answer | 98 |  |
|  |  |  |  |
| **I4.** | **When you smoke/smoked, how many per day is/was usual?** | | |
|  | Up to 10 | 1 |  |
|  | More than 10, up to 20 | 2 |  |
|  | More than 20 | 3 |  |
|  | Not a regular smoker (<1 cigarette per day) | 4 | *Go to J1* |
|  | Difficult to answer | 97 |  |
|  | Refuse to answer | 98 |  |
|  |  |  |  |
| **I5.** | **How old were you when you started smoking regularly?** Years_______ | | |
|  | Difficult to answer | 97 |  |
|  | Refuse to answer | 98 |  |
|  |  |  |  |

**I6. Please enter any comments you want to record on the questionnaire (for example, participant had difficulty hearing and needed to see the screen**)__________

| **Мodule L** | **ANTHROPOMETRY AND BODY COMPOSITION** |
| --- | --- |

| **L1. Waist 1 (in cm to nearest mm)** | | | | | | |  |  |  |  |  |  |
| --- | --- | --- | --- | --- | --- | --- | --- | --- | --- | --- | --- | --- |
|  |  |  |  | **,** |  | cm |  |  |  |  |  |  |
| *If measurement is not taken please insert zero (0) in the text field.* | | | | | | |  |  |  |  |  |  |
|  |  |  |  |  |  |  |  |  |  |  |  |  |
| **L2. Hip 1 (in cm to nearest mm)** | | | | | | |  |  |  |  |  |  |
|  |  |  |  | **,** |  | cm |  |  |  |  |  |  |
|  |  |  |  |  |  |  |  |  |  |  |  |  |
| *If measurement is not tken please insert zero (0) in the text field.* | | | | | | |  |  |  |  |  |  |
| **L3. Waist 2 (in cm to nearest mm)** | | | | | | |  |  |  |  |  |  |
|  |  |  |  | **,** |  | cm |  |  |  |  |  |  |
|  |  |  |  |  |  |  |  |  |  |  |  |  |
| *If measurement is not taken please insert zero (0) in the text field.* | | | | | | |  |  |  |  |  |  |
| **L4. Hip 2 (in cm to nearest mm)** | | | | | | |  |  |  |  |  |  |
|  |  |  |  | **,** |  | cm |  |  |  |  |  |  |
|  |  |  |  |  |  |  |  |  |  |  |  |  |
| *If measurement is not taken please insert zero (0) in the text field.* | | | | | | |  |  |  |  |  |  |
| **L5. Height 1 (in cm to nearest mm)** | | | | | | |  |  |  |  |  |  |
|  |  |  |  | **,** |  | cm |  |  |  |  |  |  |
| *If measurement is not taken please insert zero (0) in the text field.* | | | | | | |  |  |  |  |  |  |
| **L6. Height 2 (in cm to nearest mm)** | | | | | | |  |  |  |  |  |  |
| *If measurement is not taken please insert zero (0) in the text field.* | | | | | | |  |  |  |  |  |  |

| **L7.** | **Body composition measured.**  **I am now going to use the Tanita Body Analyser***.  Tell the participant that the contradictions for body composition analysis are having a pacemaker and pregnancy. In the absence of contraindications and consent of the participants use the Tanita body composition analyser in standard mode. If there are contraindications or the participant refuses body composition analysis put the analyser in weight only mode and record weight. Please tell the participant that there are no risks for pregnant women or people with pacemakers when weight only mode on Tanita device is used.* | | | | | | | | |
| --- | --- | --- | --- | --- | --- | --- | --- | --- | --- |
|  | Body composition analysis measured | | | | | | 1 | |  |
|  | Body composition analysis not carried out (pacemaker) | | | | | | 2 | |  |
|  | Body composition analysis not carried out (pregnant) | | | | | | 3 | |  |
|  | Body composition analysis not carried out (other reason) | | | | | | 4 | |  |
|  |  | | | | | |  | |  |
|  | *If measurement is not taken please insert zero (0) in the text field. Collect data from Tanita in next question if answer is 1.* | | | | | | | | |
|  | *Collect the data file for the participant for the Tanita body analyzer. Before continuing make sure the measurement has been taken successfully and participant data has been exported. Once ready, press Next to proceed and collect the data into the system.* | | | | | | | | |
| **L7c.** | **Interviewer - was the measurement successfully taken, data exported and collected?** *Answer yes only if the data collection in the previous screen has been successful!* | | | | | | | | |
|  | Yes | | | | | | 1 | |  |
|  | No | | | | | | 2 | | *Go to L7d* |
|  |  | | | | | |  | |  |
| **L7d.** | **If no why was Tanita measurement not successful?** | | | | | | | | |
|  | Participant refused | | | | | | 1 | |  |
|  | Not applicable to participant | | | | | | 2 | |  |
|  | Couldn’t export data | | | | | | 3 | |  |
|  | Couldn’t collect data | | | | | | 4 | |  |
|  | Other | | | | | | 5 | | *Go to L7e* |
|  |  | | | | | |  | |  |
| **L7e.** | **Please explain reason why Tanita measurement wasn’t successful**_______ | | | | | | | | |
|  |  | | | | | | | | |
| **L7b.** | **Weight 1 (to nearest 0.1 kg).** *If L7= 2,3,4* | | | | | | | | |
|  |  | | | | | | | | |
|  |  |  |  |  | **.** |  | | kg | |

| **Module О** | **ECG AND VICODER** |
| --- | --- |

| **T1.** | **Room Temperature (to nearest 0.1˚C).** | | | | | |
| --- | --- | --- | --- | --- | --- | --- |
|  |  |  |  | , |  | ºС |
|  |  | |  | | | |

**I am now going to measure your heart activity with the Cardiax device**.

*Explain details to the participant if required. Proceed with setting up the equipment as required and proceed when ready.*

**Cardiax Measurement Instructions.**

1. Plug cable a into laptop.
2. Start Cardiax software.
3. Place electrode on participant.
4. Take measurement.
5. Export measurement (to CSV).
6. Save electrocardiogram as PDF.

**M1.** *Collect the data file(s) for the participant for the Cardiax measurement. Before continuing make sure the measurement has been taken successfully and participant data has been exported. Once ready, press Next to proceed and collect the data into the system.*

**M1_2.** *Collect the PDF file(s) for the participant for the Cardiax measurement.*

| **O1a.** | **Was the measurement successfully taken, data exported and collected?** *Answer yes only if the data collection in the previous screen has been successful!* | |
| --- | --- | --- |
|  | Yes | 1 |
|  | No | 2 *Go to O1b* |
|  |  |  |
| **M1ab.** | *Please give a brief explanation of the results from the Cardiax measurement*. | |
| **O1b.** | **If no why Cardiax measurement was not successful?** | |
|  | Participant refused | 1 |
|  | Not applicable to participant | 2 |
|  | Couldn’t export data | 3 |
|  | Couldn’t collect data | 4 |
|  | Other | 5 *Go to O1c* |
|  |  |  |
| **O1c. Please explain reason why cardiax measurement wasn’t successful?**______ | | |

**Vicoder**

| **T4.** | **Room Temperature (to nearest 0.1˚C)** | | | | | |
| --- | --- | --- | --- | --- | --- | --- |
|  |  |  |  | , |  | ºС |

**I am now going to use the Vicorder Machine**.

*Explain details to the participant if required. Proceed with setting up the equipment as required and proceed when ready. Record three measurements of pulse wave velocity, 1 measure of blood pressure and three measures of pulse wave analysis*.

**O2e.** **Did the participant smoke in the past 3 hours?**

1. Yes
2. No

**O2f. Record the distance from the supra-sternal notch to the bottom of the neck cuff in cm to the nearest mm.** *Distance from supra-sternal notch to TOP of thigh cuff to be entered directly into Vicorder software.*

cm

**O2g. Record distance from shoulder at level of sternal notch to top of thigh cuff (only to be entered if participant has a protruding stomach) in cm to the nearest mm.**

*Distance from supra-sternal notch to TOP of thigh cuff to be entered directly into Vicorder software.*

. cm

**O2. Collect the data file for the participant for the Vicorder measurement.** *Before continuing make sure the measurement has been taken successfully and participant data has been exported. Once ready, press Next to proceed and collect the data into the system.*

**O2_1. Collect the data file for the participant for the Vicorder measurement.** *Before continuing make sure the measurement has been taken successfully and participant data has been exported. Once ready, press Next to proceed and collect the data into the system.*

**O2h. Was the femoral or carotid cuff repositioned at any point during vicorder examination?**

1. Yes *Go to O2_i*
2. No *Go to* *O2_b*

**O2i. If the cuff were repositioned please enter the new distance measured between the supra- sternal notch and the top of the thigh cuff.**

cm

**O2j. If the cuff were repositioned please enter the new distance measured between the supra- sternal notch and the bottom of the carotid cuff.**

. cm

| **O2b.** | **Interviewer - Was the vicorder measurement successfully taken, data exported and collected?** *Answer yes only if the data collection in the previous screen has been successful!* | |
| --- | --- | --- |
|  | Yes | 1 |
|  | No | 2 *Go to O2c* |
|  |  |  |
| **O2c.** | **Record reason vicorder measurement was not successful?** | |
|  | Participant refused | 1 |
|  | Not applicable to participant | 2 |
|  | Couldn’t export data | 3 |
|  | Couldn’t collect data | 4 |
|  | Other | 5 *Go to O2d* |
|  |  |  |
| **O2d.** | **Record reason why Vicorder measurement wasn’t successful.** | |
|  |  | |
| **O2k.** | **Please enter any comments you want to record on the vicorder examination.** | |

| **Мodule О3** | | | | | | | **ACTIHEART** | | | | | | | | | | |  |
| --- | --- | --- | --- | --- | --- | --- | --- | --- | --- | --- | --- | --- | --- | --- | --- | --- | --- | --- |
| **P5a.** | **Would you like to have an Actiheart device fitted and calibrated to measure your Physical activity?** | | | | | | | | | | | | | | | | | |
|  | Yes | | | | | | | | | | | 1 | | | | | | |
|  | No | | | | | | | | | | | 2 | | | | | | |
| *Explain details to the participant if required. Proceed with setting up the equipment as required and proceed when ready. 200m self-paced walk test: Ensure participant is standing quiet and still at least 1min prior to walk test.* | | | | | | | | | | | | | | | | | | |
| **I am now going to set up the Actiheart Machine.** | | | | | | | | | | | | | | | | | | |
|  | | | | | | | | | | | | | | | | | | |
| **O4.** | | **Real time start:** | | | | | | | _______:_______ | | | | | |  | | | |
|  | | |  |  |  | | |  | |  |  | |  |  | |  | | |
|  | | |  |  |  | | |  | |  |  | |  |  | |  | | |
| **O5.** | **Duration of the test:** | | | | | _______:_______ | | | | | | | | | | | | |
|  | | |  |  |  | | |  | |  |  | |  |  | |  | | |
| **O6. Total number of completed laps:** | | | | |  | | |  | |  |  | |  |  | |  | | |
|  | | |  |  |  | | |  | |  |  | |  |  | |  | | |
| **O6a.** | | **Distance on final lap in meters (In-complete laps only):** | | | | | | | | | | | | | | |  | |
|  | |  | | | | | | | | | |  | | | | | | |
| **O7.** | | **Was any walking aid used?** | | | | | | | | | |  | | | | | | |
|  | | Yes | | | | | | | | | | 1 | | | | | | |
|  | | No | | | | | | | | | | 2 *Go to O8* | | | | | | |
| **O7a.** | | **What type (e.g frame/stick), specify** ________ | | | | | | | | | |  | | | | | | |
|  | |  | | | | | | | | | |  | | | | | | |
| **O8.** | | **Comments:_______** | | | | | | | | | |  | | | | | | |
| **I am now going to collect the Actiheart measurement** *Explain details to the participant if required. Proceed with setting up the equipment as required and proceed when ready*. *Collect the data file(s) for the participant for the Actiheart measurement. Before continuing make sure the measurement has been taken successfully and participant data has been exported. Once ready, press Next to proceed and collect the data into the system.* | | | | | | | | | | | | | | | | | | |
| **P5b.** | | **Was the actiheart exercise measurement successfully taken, data exported and collected?** Answer yes only if the data collection in the previous screen has been successful! | | | | | | | | | | | | | | | | |
|  | | Yes | | | | | | | | | | 1 *Go to P5e* | | | | | | |
|  | | No | | | | | | | | | | 2 *Go to P5c* | | | | | | |
|  | |  | | | | | | | | | |  | | | | | | |
| **P5c.** | | **Reason Actiheart measurement not successful:** | | | | | | | | | | | | | | | | |
|  | | Participant refused | | | | | | | | | | 1 | | | | | | |
|  | | Not applicable to participant | | | | | | | | | | 2 | | | | | | |
|  | | Couldn’t export data | | | | | | | | | | 3 | | | | | | |
|  | | Couldn’t collect data | | | | | | | | | | 4 | | | | | | |
|  | | Other | | | | | | | | | | 5 *Go to P5d* | | | | | | |
|  | |  | | | | | | | | | |  | | | | | | |
| **P5d.** | | **Why was the Actiheart exercise data collection not successful?** | | | | | | | | | | | | | | | | |
|  | |  | | | | | | | | | |  | | | | | | |
| **P5e.** | | **Interviewer - Was the Actiheart successfully fitted and calibrated?** | | | | | | | | | | | | | | | | |
|  | | Yes | | | | | | | | | | 1 | | | | | | |
|  | | No | | | | | | | | | | 2 *Go to P5f* | | | | | | |
| **P5f.** | | **Reason Actiheart set up not successful?** | | | | | | | | | |  | | | | | | |
|  | | Participant refused | | | | | | | | | | 1 | | | | | | |
|  | | Not applicable to participant | | | | | | | | | | 2 | | | | | | |
|  | | Calibration failed | | | | | | | | | | 3 | | | | | | |
|  | | Other | | | | | | | | | | 4 | | | | | | |
| **P5g.** | | **Please explain the reason why the Actiheart set up wasn’t successful?** | | | | | | | | | | | | | | | | |

| **Мodule P4** | **ACTIHEART COLLECTION** |
| --- | --- |

**I am now going to collect the Actiheart measurement.**

*Explain details to the participant if required. Proceed with setting up the equipment as required and proceed when ready.*

| **P4.** | **Collect the data file(s) for the participant for the Actiheart measurement.** *Before continuing make sure the measurement has been taken successfully and participant data has been exported. Once ready, press Next to proceed and collect the data into the system.* | | |
| --- | --- | --- | --- |
|  |  | | |
| **P4a.** | **Interviewer - Was the measurement successfully taken, data exported and collected?**  *Answer yes only if the data collection in the previous screen has been successful!* | | |
|  | Yes | | 1 *Go to P4d* |
|  | No | | 2 |
|  |  | |  |
| **P4b.** | **Why was the Actiheart data collection not successful?** | | |
|  |  | | |
|  | Participant refused | | 1 |
|  | Not applicable to participant | | 2 |
|  | Couldn’t export data | | 3 |
|  | Couldn’t collect data | | 4 |
|  | Other | | 5 *Go to P4c* |
|  |  | |  |
| **P4c.** | **Please explain the reason why the Actiheart data collection wasn’t successful?** | | |
|  |  | | |
| **P4d.** | | **When did participant stop wearing the Actiheart device according to their diary? (Date)** | |
| «____»___________20___г | | | |
|  | | | |
| **P4e.** | | **When did participant stop wearing the Actiheart device according to their diary? (Time)** | |
|  | | _______:_______ | |

| **Мodule M** | **PHYSICAL FUNCTIONING TESTS** |
| --- | --- |

**We are now going to perform a Grip Strength Test.**

**Grip Strength.**

*Grip strength should be measured with participant sitting down in chair with arm support. VERY IMPORTANT: Adjust the lever of the Hand Dynamometer so it suits the hand. It should rest on middle piece of the four fingers.*

|  | **M1a.** | | **Was participant willing to do this test?** | | |  | |
| --- | --- | --- | --- | --- | --- | --- | --- |
|  |  | | Yes | | | 1 | |
|  |  | | No | | | 2 | |
|  |  | | Unable for health reasons | | | 3 | |
|  |  | | Unable for other reason (e.g. equipment not available) | | | 4 | |
|  |  | |  | | |  | |
|  | **M1b.** | | **Please record the reason why the test was not carried out.** | | | | |
| **LEFT HAND** | | | | **RIGHT HAND** | | |  |
| **М2.** | | **1st measurement (to nearest 0,1 kg)** \| \| \| **,** \| \| **kg** No measurement could be obtained 98 | | **М3.** | **1st measurement (to nearest 0,1 kg)** \| \| \| **,** \| \| **kg** No measurement could be obtained 98 | |  |
| **М4.** | | **2^nd^ measurement (to nearest 0,1 kg)** \| \| \| **,** \| \| **kg** No measurement could be obtained 98 | | **М5.** | **2^nd^ measurement (to nearest 0,1 kg)** \| \| \| **,** \| \| **kg** No measurement could be obtained 98 | |  |
| **М6.** | | **3^rd^ measurement (to nearest 0,1 кг)** \| \| \| **,** \| \| **kg** No measurement could be obtained 98 | | **М7.** | **3^rd^ measurement (to nearest 0,1 kg)** \| \| \| **,** \| \| **kg** No measurement could be obtained 98 | |  |

| **M8.** | **Hand Dominance** | Right-Handed | 1 |  |  |
| --- | --- | --- | --- | --- | --- |
|  | Left-Handed | | 2 |  |  |
|  | Ambidexterous (can write equally well with both hands) | | 3 |  |  |

**Balance test (one leg)**

**We are now going to perform a Balance Test.**

|  | **M9a.** | **Was participant willing to do this test?** | | | | | | | | | | |
| --- | --- | --- | --- | --- | --- | --- | --- | --- | --- | --- | --- | --- |
|  |  | Yes | | | | | | | | 1 | | |
|  |  | No | | | | | | | | 2 | | |
|  |  | Unable for health reasons | | | | | | | | 3 | | |
|  |  | Unable for other reason (e.g. equipment not available) | | | | | | | | 4 | | |
|  |  |  | | | | | | | | |  | |
|  | **M9b.** | **If participant is unwilling or unable to do test please record the reason why**___ | | | | | | | | | | |
|  |  |  | | | | | | | | | | |
| **M10.** | | | **Balance test 1^st^ measurement. Eyes open.**  **Time balance held (eyes open) to the nearest 0.1 second.** | | | | | | | | |  |
|  | | |  | | | | | |  | | |  |
|  | | |  |  |  |  | **.** |  | Seconds | | |  |
|  | | | *If Participant unable to stand unsupported please press “Refuse to answer”.* | | | | | | | | |  |
|  | | |  |  |  |  |  |  |  |  |  |  |
|  | | |  | | | | | |  | | |  |
| **M11.** | | | **Balance test 2^nd^ measurement. Eyes closed.**  **Time balance held (eyes closed) to the nearest 0.1 second.** | | | | | | | | |  |
|  | | |  | | | | | |  | | |  |
|  | | |  |  |  |  | **.** |  | Seconds | | |  |
|  | | |  | | | | | |  | | |  |
|  | | | *If Participant unable to stand unsupported please press “Refuse to answer”.* | | | | | | | | |  |

**Chair stands**

**We are now going to perform a Chair Stands Test.**

| **M12a.** | | **Was participant willing to do this test?** | | | | | | | | | | | | | | |
| --- | --- | --- | --- | --- | --- | --- | --- | --- | --- | --- | --- | --- | --- | --- | --- | --- |
|  | | Yes | | | | | | | | | 1 | | | | | |
|  | | No | | | | | | | | | 2 | | | | | |
|  | | Unable for health reasons | | | | | | | | | 3 | | | | | |
|  | | Unable for other reason (e.g. equipment not available) | | | | | | | | | 4 | | | | | |
|  | |  | | | | | | | | | | | | | |  |
| **M12b.** | | **If participant is unwilling or unable to do test please record the reason why**._____ | | | | | | | | | | | | | | |
|  |  | | | | | | | | | | | | | | | |
| **M13.** | **Time taken for chair stands (nearest 0.1 second).** | | | | | | | | | | | | | | | |
|  |  | | | | | | | | | | | | | | | |
|  |  | |  |  |  | | . | |  | | | | | |  | |
|  |  | | | | | | | | | | | | | | | |
|  | *If Participant unable to stand unsupported please press "Refuse to answer"* | | | | | | | | | | | | | | | |
|  |  | | | | | | | | | | | | | | | |
| **M14.** | **Number of chair stands completed (maximum 10).** | | | | | | | | | | | | | | | |
|  |  | | | | | | | | | | |  | | | | |
|  |  | | | | |  | |  | | | | |  | | | |
|  |  | | | | | | | | | | | | | | | |
|  | *If Participant unable to stand unsupported please press "Refuse to answer"* | | | | | | | | | | | | | | | |
|  |  | | | | | | | | | | | | | | | |
| **M15.** | **If less than 10 chair stands completed what was the reason for doing less.** | | | | | | | | | | | | | | | |
|  |  | | | | | | | | | | | | | | | |
|  | Participant unable to stand unsupported | | | | | | | | | 1 | | | | | | |
|  | Participant became breathless | | | | | | | | | 2 | | | | | | |
|  | Participant stopped and unable to continue | | | | | | | | | 3 | | | | | | |
|  | You or participant felt the test was unsafe | | | | | | | | | 4 | | | | | | |
|  | Test took longer than 2 minutes | | | | | | | | | 5 | | | | | | |
|  | Other reason | | | | | | | | | 6 | | | |  | | |
|  |  | | | | | | | | |  | | | |  | | |
| **M15a. Other reason (specify)**__________ | | | | | | | | | | | | | | | | |

| **Мodule P** | **ECHO and VASCULAR ULTRASOUND** |
| --- | --- |

| **T5.** | **Room Temperature (to nearest 0.1˚C).** | | | | | |
| --- | --- | --- | --- | --- | --- | --- |
|  |  |  |  | , |  | ºС |

| **P1.** | **Was the Vascular Ultrasound successfully completed?** | | |
| --- | --- | --- | --- |
|  |  | | |
|  | Yes | 1 |  |
|  | No, participant refused | 2 |  |
|  | No, other reason | 3 | *Go to P1a* |
|  |  |  |  |
|  | *Please make sure that the files are collected and stored using the Participant ID in the EchoPac Computer.* | | |
| **P1b.** | **Please explain reason why vacular ultrasound measurement wasn’t successful?**____ | | |
| **P1_1.**  **P1_2** | **Time vascular ultrasound examination began** _______:_______  **Time vascular ultrasound examination ended** _______:_______ | | |
| **P3.** | **ECHO completed.** |  |  |
|  | Yes | 1 |  |
|  | No, participant refused | 2 |  |
|  | No, other reason | 3 | *Go to P3a* |
|  | *Please make sure that the files are collected and stored using the Participant ID in the EchoPac Computer*. | | |
| **P3a.** | **Please explain the reason why the ECHO measurement wasn’t successful?** | | |
|  |  | | |
| **P2_1.** | **Time ECHO began** _______:_______ | | |
| **P2_2.** | **Time ECHO ended**_______:_______ | | |

|  | *Questions P1c and P1d only for Novosibirsk*  *Please make sure that the files are collected and stored using the Participant ID in the EchoPac Computer.* | | |
| --- | --- | --- | --- |
|  |  | | |
| **P1c.** | **Was the abdominal ultrasound measurement successfully completed?** | | |
|  |  | | |
|  | Yes | 1 |  |
|  | No, participant refused | 2 |  |
|  | No, other reason | 3 | *Go to P1d* |
|  |  |  |  |
| **P1d.** | **Please explain the reason why Abdominal Ultrasound measurement wasn’t successful?**_______ | | |
|  |  |  |  |

| **Мodule S** | **SPIROMETRY** |
| --- | --- |

| **T6.** | **Room Temperature(to nearest 0.1˚C)** |
| --- | --- |
|  |  |
|  | **. ˚C** |

**I am now going to use the Spirometer Machine.**

*Explain details to the participant and ask if they want to participate. Proceed with setting up the equipment as required and proceed when ready. Contraindications for spirometry are chest infection in the last month, history of detached retina, myocardial infarction in past month, surgery to eyes, chest or abdomen in past 3 months, pregnancy 1st or 3rd trimester, currently on medication for tuberculosis*.

| **S1b.** | **Spirometry completed (for subset of participants)** | |
| --- | --- | --- |
|  | Yes | 1 |
|  | No, participant refused | 2 |
|  | No, participant not asked to do spirometry | 3 |
|  | No, contraindication | 4 *Go to S2* |
|  | No, other reason (please specify) | 5 *Go to S1d* |
|  |  |  |
| **S1.** | **Collect the data file for the participant for the Spirometry measurement.**  *Before continuing make sure the measurement has been taken successfully and participant data has been exported. Once ready, press Next to proceed and collect the data into the system.* | |
|  |  |  |
| **S1d.** | **Other reason. Please specify why Spirometry was not successful.** | |
|  |  | |
| **S2.** | **Contraindications for spirometry.** |  |
|  | Chest infection in the past month | 1 |
|  | History of detached retina | 2 |
|  | Myocardial infarction in past month | 3 |
|  | Surgery to eyes, chest or abdomen in past 3 months | 4 |
|  | Pregnancy 1^st^ or 3^rd^ trimester | 5 |
|  | Currently on medication for tuberculosis | 6 |
|  |  |  |
| **S1c.** | **In the past hour did the participant** |  |
|  | Have a drink containing caffeine | 1 |
|  | Use an inhaler | 2 |
|  | Smoke | 3 |
|  | None of the above | 4 |
|  |  |  |
