# Supplementary material for: Know Your Heart: Rationale, design and conduct of a cross-sectional study of cardiovascular structure, function and risk factors in 4500 men and women aged 35-69 years from two Russian cities, 2015-18
Source: Wellcome Open Res. 2018 Dec 3;3:67. Originally published 2018 Jun 4. [Version 3] doi: 10.12688/wellcomeopenres.14619.3 (PMC6073094; doi:10.12688/wellcomeopenres.14619.3)
Supplement: Supplementary file 3 [file wellcomeopenres-3-16294-s0002.tgz › d9a03f66-5fce-45a3-8a2e-d18febd22997_Supplementary_file_3._Questionnaires_and_data_collection_tools.zip!/questionnaires_181121_publish_version2/181118_HC_proforma_survey_RU.docx]

|  |  |
| --- | --- |

**Проформа и опросник этапа медицинского обследования**

Международный проект по сердечно-сосудистым заболеваниям в России

2015-2018

Версия публикации 2.0 Ноябрь 2018

В России один из самых высоких уровней сердечно-сосудистых заболеваемости в мире. Целью международного проекта по сердечно-сосудистой заболеваемости в России является понимание причин данного положения. Весомым компонентом данного исследования было исследование От Сердца к Сердцу, направленного на выявление природы и причин сердечно-сосудистой заболеваемости в России. В рамках данного проекта были проведены масштабные перекрёстные опросы в двух городах России, Новосибирске и Архангельске. Ислледование охватило 4542 мужчин и женщин в возрате 35-69 лет, набранных из генеральной популяции в период 2015-2018. Исследование включало два компонента: 1)опрос населения для получения информации по социально-демографическим характеристикам и сердечно-сосудистым факторам риска; в большинстве случаев опрос проводился на дому. 2)комплексное медицинское обследование в клинике, включающее детальное изучение сердечно-сосудистой системы.

Международный проект "Сердечно-сосудистые заболевания в России" был частично финансирован Wellcome Trust Strategic Award [100217]. Проект также был финансирован Арктическим Университетом Норвегии, Университет Тромсё, Норвежским Институтом Общественного Здоровья и Министерством Здравоохранения и Социального Обеспечения Норвегии.

Разработка опросников велась под руководством Лондонской Школы Гигиены и Тропической Медицины совместно с Северным государственным медицинским университетом, г.Архангельск, Новосибирским государственным медицинским университетом, Научно-исследовательским институтом терапии и профилактической медицины-филиала «ФИЦ ИЦиГ» СО РАН, г. Новосибирск и коллегами других институтов.

Данная бумажная версия опросника и проформы были использованы для сбора данных электронно используя SURVANT (NETELIXIS IT SOLUTIONS 2015. SURVANT survey authoring and data collection software: version 1.0. <http://www.survant.net>.)

Подробные детали исследования опубликованы в Know Your Heart: Rationale, design and conduct of a cross-sectional study of cardiovascular structure, function and risk factors in 4500 men and women aged 35-69 years from two Russian cities, 2015-18
Cook S, Malyutina S, Kudryavtsev AV, Averina M, Bobrova N, Boytsov S, Brage S, Clark T et al.

Содержание

[Модуль J. Регистрация/сведения о последнем приёме пищи	4](#_Toc512505991)

[Модуль K. Измерение артериального давления	5](#_Toc512505992)

[Модуль R. Забор крови.	7](#_Toc512505994)

[Модуль A. Кардиологический анамнез	9](#_Toc512505995)

[Модуль B. Стенокардия напряжения.	11](#_Toc512505996)

[Модуль C. Медикаменты.	14](#_Toc512505996)

[Модуль D. Самооценка состояния здоровья	18](#_Toc512505997)

[Модуль E. Респираторная функция	19](#_Toc512505999)

[Модуль F. Другие заболевания	21](#_Toc512506001)

[Модуль G. Вопросы только для женщин	23](#_Toc512506003)

Модуль H. Вопросы по употреблению алкоголя	26

[Модуль I. [Курение	29](#_Toc512506003)](#_Toc512506003)

[[Модуль L. Окружность талии, бедер, рост, вес и состав тела	30](#_Toc512506003)](#_Toc512506009)

[Модуль O. ЭКГ и Викодер	32](#_Toc512506003)

[Модуль O3. Актихарт	35](#_Toc512506009)

[Модуль P4. [Мониторинг ЧСС и расхода энергии/сбор данных	37](#_Toc512506003)](#_Toc512506003)

[[Модуль M. Проверка физических функций	38](#_Toc512506003)](#_Toc512506009)

[Модуль P. ЭХО и УЗИ сосудов	41](#_Toc512506003)

Модуль S. Сприрометрия………………………………………………………………………..43

| **Модуль J** | **РЕГИСТРАЦИЯ/СВЕДЕНИЯ О ПОСЛЕДНЕМ ПРИЕМЕ ПИЩИ** |
| --- | --- |

*Сделайте акцент на важности точных ответов для участника.***Теперь я задам вам несколько вопросов о потреблении пищи и напитков сегодня. Это может повлиять на результаты нескольких тестов, поэтому важно, чтобы ваши ответы были наиболее точными**.

| **J1.** **Когда вы в последний раз что-либо ели или пили, кроме воды?**  «____»___________20___г (ДАТА)  **J2. Время последнего приема пищи.** *Пожалуйста, используйте 24-часовой формат***:**  _______:_______ | | | |
| --- | --- | --- | --- |
| **J3. Что Вы ели?** | | | |
|  | Легкую закуску | 1 |  |
|  | Полноценный прием пищи  Tолько напитки | 2  3 |  |
|  | Затрудняюсь ответить | 97 |  |
|  | Отказ от ответа | 98 |  |
|  |  |  |  |

| **J4.** | **Пили ли Вы что-нибудь в течение последних 4-х часов?** | | |
| --- | --- | --- | --- |
|  | Да | 1 |  |
|  | Нет | 2 | *Переход к J6* |
|  | Затрудняюсь ответить | 97 | *Переход к J6* |
|  | Отказ от ответа | 98 | *Переход к J6* |

| **J5.** | **Какие это были напитки?** *Возможно несколько вариантов ответа.* | | |
| --- | --- | --- | --- |
|  |  | | |
|  | Любые алкогольные напитки | 1 |  |
|  | Кофе, черный чай, кока-колу, пепси, другие напитки, содержащие кофеин | 2 |  |
|  | Другое | 3 |  |
|  | Затрудняюсь ответить | 97 |  |
|  | Отказ от ответа | 98 |  |
|  |  |  |  |
| **J6.** | **Употребляли ли Вы алкогольные напитки в течение последних 24 часов до медицинского осмотра?** | | |
|  | Да | 1 |  |
|  | Нет | 2 |  |
|  | Затрудняюсь ответить | 97 |  |
|  | Отказ от ответа | 98 |  |

| **Модуль К** | **ИЗМЕРЕНИЕ АРТЕРИАЛЬНОГО ДАВЛЕНИЯ** |
| --- | --- |

**Сейчас я измерю Ваше артериальное давление.**

*Сделайте акцент для участника нн важности получения точных результатов.*

| **K1.** | **Температура в кабинете (до ближайшего 0,1ºC)** | | | | | | | | |
| --- | --- | --- | --- | --- | --- | --- | --- | --- | --- |
|  |  | | |  |  | | **,** |  | ºС |
|  | Измерение не проводилось | | | | 98 | | | | |
| **Инструкция по измерению артериального давления***.*  1. Подключите кабель OMRON к ноутбуку.  2. Запустите программное обеспечение для измерения артериального давления.  3. Сделайте три измерения, делая двухминутные перерывы между ними.  4. Экспортируйте данные измерений в формате CSV. | | | | | | | | | |
| **K5.** | **Сбор файл(ы) для участника для измерения артериального давления.**  *Прежде чем продолжить, убедитесь, что измерение было успешно проведено и данные участника были экспортированы. После этого нажмите "Далее", чтобы ввести данные в систему* | | | | | | | | |
|  |  | | | | | | | | |
| **K5a.** | **Интервьюер - измерение успешно произведено, данные экспортированы и собраны?** | | | | | | | | |
| Да | | 1 | | | | | | | |
| Нет | | 2 *Переход к K5b* | | | | | | | |
|  |  | | | | | | | | |
| **K2.** | *Укажите, на какой руке измерялось артериальное давление*  **На какой руке измерялось артериальное давление?** | | | | | | | | |
|  |  | | | | | | | | |
|  | Правая | | | | 1 | |  | | |
|  | Левая | | | | 2 | |  | | |
| **K3.** | **Окружность руки (до ближайшего 0,1 см)** | | | | | | | | |
|  |  | | |  |  | | **,** |  | см |
|  | Измерение не проводилось | | | | 98 | | | | |
| **K4**. | **Размер манжеты** | | | | |  | | | |
|  | Маленький | | | | | 1 | | | |
|  | Средний | | | | | 2 | | | |
|  | Большой | | | | | 3 | | | |
|  |  | | | | | | | | |
| **K5b.** | **Почему измерение артериального давления не было успешным?** | | | | | | | | |
|  |  | | | | | | | | |
|  | Участник отказался | | 1 | | | | | | |
|  | Не применимо к участнику | | 2 | | | | | | |
|  | Не удалось экспортировать данные | | 3 | | | | | | |
|  | Не удалось собрать данные | | 4 | | | | | | |
|  | Другое | | 5 *Переход к K5c* | | | | | | |
|  |  | |  |  |  | |  | | |
| **K5c.** | **Пожалуйста, объясните, почему измерение артериального давления не было успешным**_______ | | | | | | | | |

**K5d. Введите любые комментарии, которые вы считаете необходимыми, в связи с измерением АД (например, если АД было очень высоким, и вам пришлось перенести обследование на более поздний срок).**

| **K14.** | **Насыщение кислородом** |  |  | % |
| --- | --- | --- | --- | --- |

| **Модуль R** | **ЗАБОР КРОВИ** |
| --- | --- |

| **T2_a.** | **Температура в кабинете (до ближайшего 0,1ºC)** | | | | | |
| --- | --- | --- | --- | --- | --- | --- |
|  |  |  |  | , |  | ºС |
|  | Измерение не проводилось | | 98 | | | |

| **R1.** | **Откуда осуществлялся забор крови?** | | | | | | | |  |
| --- | --- | --- | --- | --- | --- | --- | --- | --- | --- |
|  | Из правой руки | | | 1 | | *Переход к R3* | | |  |
|  | Из левой руки | | | 2 | | *Переход к R3* | | |  |
|  | Забор крови не осуществлялся | | | 3 | | *Переход к R3* | | |  |
|  | Забор крови из другого участка | | | 4 | |  | | |  |
|  |  | | |  | |  | | |  |
| **R2.** | **Укажите, откуда осуществлялся забор крови.** | | | | | | | |  |
|  | | | | | | | | |  |
| **R3.** | **Вакуумная пробирка (8,5 мл) для отделения сыворотки 1 (с оранжевым колпачком) была наполнена** | | | | | | | |  |
|  | Да, полностью | | | 1 | |  | | |  |
|  | Да, частично | | | 2 | |  | | |  |
|  | Нет | | | 3 | |  | | |  |
|  |  | | |  | |  | | |  |
| **R4.** | **Вакуумная пробирка (8,5 мл) для отделения сыворотки 2 (с оранжевым колпачком) была наполнена** | | | | | | | |  |
|  | Да, полностью | 1 | | | | | | | |
|  | Да, частично | 2 | | | | | | | |
|  | Нет | 3 | | | | | | | |
|  |  |  | | | | | | | |
| **R5.** | **Вакуумная пробирка (8,5 мл) для отделения сыворотки 3 (с оранжевым колпачком) была наполнена** | | | | | | | |  |
|  | Да, полностью | | 1 | |  | | |  |  |
|  | Да, частично | | 2 | |  | | |  |  |
|  | Нет | | 3 | |  | | |  |  |
|  |  | |  | |  | | |  |  |
| **R6.** | **Вакуумная пробирка (8,5 мл) для отделения сыворотки 4 (с оранжевой крышкой) была наполнена** | | | | | | |  |  |
|  | Да, полностью | | 1 | |  | | |  |  |
|  | Да, частично | | 2 | |  | | |  |  |
|  | Нет | | 3 | |  | | |  |  |
|  |  | |  | |  | | |  |  |
| **R7.** | **Вакуумная пробирка (10 мл) с ЭДТА 1 (с фиолетовой крышкой) была наполнена** | | | | | | |  |  |
|  |  | |  | |  | | |  |  |
|  | Да, полностью | | 1 | |  | | |  |  |
|  | Да, частично | | 2 | |  | | |  |  |
|  | Нет | | 3 | |  | | |  |  |
|  |  | |  | |  | | |  |  |
| **R8.** | **Вакуумная пробирка (6 мл) с ЭДТА 2 (с фиолетовой крышкой) была наполнена** | | | | | | |  |  |
|  |  | | | | | | |  |  |
|  | Да, полностью | | 1 | | | |  |  |  |
|  | Да, частично | | 2 | | | |  |  |  |
|  | Нет | | 3 | | | |  |  |  |
| **R9.** | **Реальное время забора крови:** _______:_______:_______ | | | | | | |  |  |

| **Модуль A** | **КАРДИОЛОГИЧЕСКИЙ АНАМНЕЗ** |
| --- | --- |

| **A1.** | **Измеряли ли Вы когда-либо артериальное давление у врача или другого медицинского работника?** | | | |
| --- | --- | --- | --- | --- |
|  |  | | | |
|  | Да | 1 | |  |
|  | Нет | 2 | | *Переход к A5* |
|  | Затрудняюсь ответить | 97 | |  |
|  | Отказ от ответа | 98 | |  |
|  |  |  | |  |
| **A2.** | **Говорил ли Вам врач или или медсестра когда-либо, что у Вас повышенное давление?** | | | |
|  |  | | | |
|  | Да | 1 | |  |
|  | Нет | 2 | | *Переход к A5* |
|  | Затрудняюсь ответить | 97 | |  |
|  | Отказ от ответа | 98 | |  |
|  |  |  | |  |
| **A3.** | **Назначал ли Вам врач медикаментозные препараты для лечения Вашего повышенного давления?** | | | |
|  |  | | | |
|  | Да | 1 | |  |
|  | Нет | 2 | | *Переход к A5* |
|  | Затрудняюсь ответить | 97 | |  |
|  | Отказ от ответа | 98 | |  |
|  |  |  | |  |
| **A4.** | **Принимаете ли Вы эти препараты?** | | | |
|  |  | | | |
|  | Каждый день | 1 | |  |
|  | Только иногда, например, тогда, когда чувствую себя плохо | 2 | |  |
|  | Никогда | 3 | |  |
|  | Затрудняюсь ответить | 97 | |  |
|  | Отказ от ответа | 98 | |  |
|  |  |  | |  |
| **A5.** | **Говорил ли вам когда-нибудь врач или медсестра о том что у Вас сахарный диабет?** | | | |
|  |  | | | |
|  | Нет | | 1 | *Переход к A8* |
|  | Да, и я принимаю инсулин | | 2 |  |
|  | Да, и я принимаю препараты для снижения сахара крови (кроме инсулина) | | 3 |  |
|  | Да, и я только соблюдаю диету | | 4 |  |
|  | Затрудняюсь ответить | | 97 | *Переход к A8* |
|  | Отказ от ответа | | 98 | *Переход к A8* |
|  |  | |  |  |
| **A6.** | **Какой тип сахарного диабета установил Вам врач?** | | | |
|  |  | | | |
|  | 1 тип | | 1 |  |
|  | 2 тип | | 2 |  |
|  | Затрудняюсь ответить | | 97 |  |
|  | Отказ от ответа | | 98 |  |
|  |  | |  |  |
| **A7.** | **В каком возрасте Вам был поставлен этот диагноз?** ______лет | | | |
|  |  | |  |  |
|  | Затрудняюсь ответить | | 97 |  |
|  | Отказ от ответа | | 98 |  |
|  |  | |  |  |
| **A8.** | **Говорил ли Вам врач когда-либо, что у Вас повышенный холестерин?** | | | |
|  | Да | | 1 |  |
|  | Нет | | 2 |  |
|  | Затрудняюсь ответить | | 97 |  |
|  | Отказ от ответа | | 98 |  |

| **Модуль B** | **СТЕНОКАРДИЯ НАПРЯЖЕНИЯ** |
| --- | --- |

| **B1.** | **Бывает ли у Вас боль или неприятное ощущение в грудной клетке?** | | |
| --- | --- | --- | --- |
|  |  | | |
|  | Да | 1 |  |
|  | Нет | 2 | *Переход к B11* |
|  | Затрудняюсь ответить | 97 |  |
|  | Отказ от ответа | 98 |  |
|  |  |  |  |
| **B2.** | **Возникает ли эта боль или неприятное ощущение, когда Вы идете в гору или спешите?** | | |
|  | Да | 1 |  |
|  | Нет | 2 |  |
|  | Никогда не ходит быстро и не поднимается в гору | 3 |  |
|  | Затрудняюсь ответить | 97 |  |
|  | Отказ от ответа | 98 |  |
|  |  |  |  |
| **B3.** | **Возникает ли эта боль или неприятное ощущение при ходьбе обычным шагом по ровному месту?** | | |
|  | Да | 1 |  |
|  | Нет | 2 |  |
|  | Никогда не ходит обычным шагом по ровному месту | 3 |  |
|  | Затрудняюсь ответить | 97 |  |
|  | Отказ от ответа | 98 |  |
|  |  |  |  |
| **B4.** | **Что Вы делаете, если боль или неприятное ощущение возникает во время ходьбы?** | | |
|  | Останавливаюсь | 1 |  |
|  | Снижаю темп ходьбы | 2 |  |
|  | Продолжаю идти, не снижая темпа | 3 |  |
|  | Затрудняюсь ответить | 97 |  |
|  | Отказ от ответа | 98 |  |
|  |  |  |  |
| **B5.** | **Проходит ли эта боль или неприятное ощущение, когда вы останавливаетесь?** | | |
|  |  | | |
|  | Да | 1 |  |
|  | Нет | 2 | *Переход к B7* |
|  | Затрудняюсь ответить | 97 | *Переход к B7* |
|  | Отказ от ответа | 98 | *Переход кB7* |
|  |  |  |  |
| **B6.** | **Как быстро проходит эта боль или неприятное ощущение в груди?** | | |
|  |  | | |
|  | Через 10 минут или быстрее | 1 |  |
|  | Более, чем через 10 минут | 2 |  |
|  | Затрудняюсь ответить | 97 |  |
|  | Отказ от ответа | 98 |  |
|  |  |  |  |
| **B7.** | **Можете ли Вы показать, где Вы ее ощущаете?** | | |
|  |  | | |
|  |  |  |  |
|  |  | | |
| **B8.** | **Была ли у Вас когда-либо сильная боль, пронизывающая грудную клетку и продолжавшаяся полчаса или более?** | | |
|  | Да | 1 |  |
|  | Нет | 2 | *Переход к B11* |
|  | Затрудняюсь ответить | 97 | *Переход к B11* |
|  | Отказ от ответа | 98 | *Переход к B11* |
|  |  |  |  |
| **B9.** | **Обращались ли Вы по поводу этой боли к врачу?** | | |
|  |  | | |
|  | Да | 1 |  |
|  | Нет | 2 | *Переход к B11* |
|  | Затрудняюсь ответить | 97 | *Переход к B11* |
|  | Отказ от ответа | 98 | *Переход к B11* |
|  |  |  |  |
| **B10.** | **Что сказал врач по этому поводу, какой диагноз был поставлен?** | | |
|  |  | | |
|  | Стенокардия (ишемическая болезнь сердца) | 1 |  |
|  | Инфаркт миокарда | 2 |  |
|  | Какая-то другая причина | 3 |  |
|  | Ничего не сказал | 4 |  |
|  | Затрудняюсь ответить | 97 |  |
|  | Отказ от ответа | 98 |  |

**B11. Говорил ли Вам когда-либо врач, что у Вас имеются/имелись или были диагностированы следующие заболевания:**

| **B11_1. Инфаркт миокарда/Cердечный приступ** | | |  |  |  |
| --- | --- | --- | --- | --- | --- |
| Да, и я был(а) госпитализирован(а) | 1 | |  |  |  |
| Да, и я не был(а) госпитализирован(а) | 2 | |  |  |  |
| Нет | 3 | |  |  |  |
| Затрудняюсь ответить | 97 | |  |  |  |
| Отказ от ответа | 98 | |  |  |  |
| **B11_2.** **Стенокардия/Ишемическая болезнь сердца** | | |  |  |  |
| Да, и я был(а) госпитализирован(а) | 1 | |  |  |  |
| Да, и я не был(а) госпитализирован(а) | 2 | |  |  |  |
| Нет | 3 | |  |  |  |
| Затрудняюсь ответить | 97 | |  |  |  |
| Отказ от ответа | 98 | |  |  |  |
|  | | |  |  |  |
| **B11_3. Мозговой инсульт** | | |  |  |  |
| Да, и я был(а) госпитализирован(а) | 1 | |  |  |  |
| Да, и я не был(а) госпитализирован(а) | 2 | |  |  |  |
| Нет | 3 | |  |  |  |
| Затрудняюсь ответить | 97 | |  |  |  |
| Отказ от ответа | 98 | |  |  |  |
|  | | |  |  |  |
| **B11_4. Операции/вмешательства на сердце** | | |  |  |  |
| Да, и я был(а) госпитализирован(а) | 1 *Переход к B11_4_4a* | | |  |  |
| Да, и я не был(а) госпитализирован(а) | 2 *Переход к B11_4_4a* | | |  |  |
| Нет | 3 *Переход к B11_5* | | |  |  |
| Затрудняюсь ответить | 97 | |  |  |  |
| Отказ от ответа | 98 | |  |  |  |
|  |  | |  |  |  |
| **B11_4_4a. Какую операция на сердце Вы перенесли (выберите все, которые у Вас были)** | | | |  |  |
| Aорто-коронарное шунтирование | 1 | |  |  |  |
| Протезирование клапанов | 2 | |  |  |  |
| Cтентирование коронарных артерий | 3 | |  |  |  |
| Другое | 4 | |  |  |  |
| Затрудняюсь ответить | 97 | |  |  |  |
| Отказ от ответа | 98 | |  |  |  |
| **B11_5. Хроническое заболевание почек** |  | |  |  |  |
| Да, и я был(а) госпитализирован(а) | 1 | |  |  |  |
| Да, и я не был(а) госпитализирован(а) | 2 | |  |  |  |
| Нет | 3 | |  |  |  |
| Затрудняюсь ответить | 97 | |  |  |  |
| Отказ от ответа | 98 | |  |  |  |
| **B11_6. Хронические болезни бронхо-легочной системы** | | | |  |  |
| Да, и я был(а) госпитализирован(а) | 1 | |  |  |  |
| Да, и я не был(а) госпитализирован(а) | 2 | |  |  |  |
| Нет | 3 | |  |  |  |
| Затрудняюсь ответить | 97 | |  |  |  |
| Отказ от ответа | 98 |  | | |  |

| **Модуль С** | | **МЕДИКАМЕНТЫ** | | |  |  |
| --- | --- | --- | --- | --- | --- | --- |

| **C1.** | **Принимаете ли вы в настоящее время какие-либо лекарственные препараты,включая ингаляторы?** | | |
| --- | --- | --- | --- |
|  | Да | 1 |  |
|  | Нет | 2 | *Переход к D1* |

**Покажите, пожалуйста, упаковки принимаемых препаратов. Если упаковки нет, то как называются эти препараты, в какой дозировке и с какой частотой принимаются? Пожалуйста, включите ингаляторы.** *Интервьюер, пожалуйста, зарегистрируйте максимум до 7-ми принимаемых препаратов. Дозировка препарата определяется "силой" препарата, н-р, количество мг в таблетке. Не регистрируйте мази, кремы и травяные сборы, биологически активные и пищевые добавки.*

|  | Название препарата | Дозировка (мг в таблетке) | Для чего принимается препарат |  | Число единиц препарата принимаемых за один раз | | | | Частота приема препарата | | |  |
| --- | --- | --- | --- | --- | --- | --- | --- | --- | --- | --- | --- | --- |
|  |  |  |  |  |  |  |  | табл./капсул/доз спрея |  |  |  | *Коды частоты приема препарата:*  01 ежедневно 3 раза в день  02 ежедневно 2 раза в день  03 ежедневно 1 раз в день  04 3 раза в неделю  05 2 раза в неделю  06 1 раз в неделю  07 регулярно, но реже чем 1 раз в неделю  08 нерегулярно, по необходимости  97 затрудняюсь ответить  98 отказ от ответа |
| **C2.** |  |  |  |  |  |  |  | мл/инъекций |  |  |  |  |
|  |  |  |  |  |  |  |  | ед. инсулина |  |  |  |  |
|  |  |  |  |  |  |  |  | капель |  |  |  |  |
|  |  |  |  |  |  |  |  |  |  |  |  |  |
|  |  |  |  |  |  |  |  | табл./капсул/доз спрея |  |  |  |  |
| **C3.** |  |  |  |  |  |  |  | мл/инъекций |  |  |  |  |
|  |  |  |  |  |  |  |  | ед.инсулина |  |  |  |  |
|  |  |  |  |  |  |  |  | капель |  |  |  |  |
|  |  |  |  |  |  |  |  |  |  |  |  |  |
|  |  |  |  |  |  |  |  | табл./капсул/доз спрея |  |  |  |  |
| **C4.** |  |  |  |  |  |  |  | мл/инъекций |  |  |  |  |
|  |  |  |  |  |  |  |  | ед.инсулина |  |  |  |  |
|  |  |  |  |  |  |  |  | капель |  |  |  |  |

|  | Название препарата | Дозировка (мг в таблетке) | Для чего принимается препарат |  | Число единиц препарата принимаемых за один раз | | | | Частота приема препарата | | |  |
| --- | --- | --- | --- | --- | --- | --- | --- | --- | --- | --- | --- | --- |
|  |  |  |  |  |  |  |  | табл./капсул/доз спрея |  |  |  | *Коды частоты приема препарата:*  01 ежедневно 3 раза в день  02 ежедневно 2 раза в день  03 ежедневно 1 раз в день  04 3 раза в неделю  05 2 раза в неделю  06 1 раз в неделю  07 регулярно, но реже чем 1 раз в неделю  08 нерегулярно, по необходимости  97 затрудняюсь ответить  98 отказ от ответа |
| **C5.** |  |  |  |  |  |  |  | мл/инъекций |  |  |  |  |
|  |  |  |  |  |  |  |  | ед. инсулина |  |  |  |  |
|  |  |  |  |  |  |  |  | капель |  |  |  |  |
|  |  |  |  |  |  |  |  |  |  |  |  |  |
|  |  |  |  |  |  |  |  | табл./капсул/доз спрея |  |  |  |  |
| **C6.** |  |  |  |  |  |  |  | мл/инъекций |  |  |  |  |
|  |  |  |  |  |  |  |  | ед. инсулина |  |  |  |  |
|  |  |  |  |  |  |  |  | капель |  |  |  |  |
|  |  |  |  |  |  |  |  |  |  |  |  |  |
|  |  |  |  |  |  |  |  | табл./капсул/доз спрея |  |  |  |  |
| **C7.** |  |  |  |  |  |  |  | мл/инъекций |  |  |  |  |
|  |  |  |  |  |  |  |  | ед. инсулина |  |  |  |  |
|  |  |  |  |  |  |  |  | капель |  |  |  |  |

| **C9_** | **Интервьюер, пожалуйста, укажите, видели ли Вы медицинские препараты респондента?** | |
| --- | --- | --- |
|  | Медицинские препараты видел/а | 1 |
|  | Медицинские препараты не видел/а, запись произведена только со слов респондента | 2 |

**C10. Введите любые комментарии, которые вы считаете необходимыми, в связи с кардиологическим опросом (например, если участник плохо слышит и ему приходилось смотреть на экран).**

| **Модуль D** | **САМООЦЕНКА ЗДОРОВЬЯ** |
| --- | --- |

| **D1.** | **Как в целом Вы оцениваете состояние Вашего здоровья?** | | |
| --- | --- | --- | --- |
|  |  |  | |
|  | Очень хорошее | 1 |  |
|  | Хорошее | 2 |  |
|  | Ни хорошее, ни плохое | 3 |  |
|  | Плохое | 4 |  |
|  | Очень плохое | 5 |  |
|  | Затрудняюсь ответить | 97 |  |
|  | Отказ от ответа | 98 |  |

| **Модуль E** | **РЕСПИРАТОРНАЯ ФУНКЦИЯ** |
| --- | --- |

|  | *Предупредите участника, что будете задавать вопросы о его/ее дыхании.*  **Сейчас я задам Вам несколько вопросов о Вашем дыхании.** | | |
| --- | --- | --- | --- |
| **E1.** | **Кашляете ли Вы обычно при пробуждении по утрам в зимнее время?** *Акцентируйте внимание участника на слове "обычно"* | | |
|  | Да | 1 |  |
|  | Нет | 2 |  |
|  | Затрудняюсь ответить | 97 |  |
|  | Отказ от ответа | 98 |  |
|  |  |  |  |
| **E2.** | **Вы обычно кашляете зимой (днём или ночью)?** | | |
|  | Да | 1 |  |
|  | Нет | 2 |  |
|  | Затрудняюсь ответить | 97 |  |
|  | Отказ от ответа | 98 |  |
|  |  |  |  |
| **E3.** | *Вопрос задается только тем, кто ответил "ДА" на вопросы Е1 или Е2* **Вы кашляете таким образом большинство дней в течение по крайней мере трёх месяцев в году?** | | |
|  | Да | 1 |  |
|  | Нет | 2 |  |
|  | Затрудняюсь ответить | 97 |  |
|  | Отказ от ответа | 98 |  |
|  |  |  |  |
| **E4** | **Откашливаете ли Вы обычно мокроту при пробуждении по утрам в зимнее время?** *Акцентируйте внимание участника на слове "обычно"* | | |
|  | Да | 1 |  |
|  | Нет | 2 |  |
|  | Затрудняюсь ответить | 97 |  |
|  | Отказ от ответа | 98 |  |
|  |  |  |  |
| **E5** | **Откашливаете ли Вы обычно мокроту в зимнее время** **днём или ночью?** *Акцентируйте внимание участника на слове "обычно"* | | |
|  | Да | 1 |  |
|  | Нет | 2 |  |
|  | Затрудняюсь ответить | 97 |  |
|  | Отказ от ответа | 98 |  |
|  |  |  |  |
| **E6** | *Cпрашивать, только при ответе «Да» на вопросы E4 и E5*  **Откашливаете ли Вы мокроту большинство дней по крайней мере 3 месяца подряд в течение года в течение года?** | | |
|  | Да | 1 |  |
|  | Нет | 2 |  |
|  | Затрудняюсь ответить | 97 |  |
|  | Отказ от ответа | 98 |  |
|  |  |  |  |
| **E7.** | **Испытываете ли Вы одышку, когда быстро идете по ровной дороге или поднимаетесь пешком на небольшой холм?** | | |
|  | Да | 1 |  |
|  | Нет | 2 | *Переход к E12* |
|  | Никогда не хожу быстро и в гору | 3 |  |
|  | Затрудняюсь ответить | 97 |  |
|  | Отказ от ответа | 98 |  |
|  |  |  |  |
| **E8.** | **Бывает ли у Вас одышка, когда вы идете по ровной дороге вместе с людьми Вашего возраста?** | | |
|  | Да | 1 |  |
|  | Нет | 2 |  |
|  | Никогда не хожу с людьми своего возраста по ровной поверхности | 3 |  |
|  | Затрудняюсь ответить | 97 |  |
|  | Отказ от ответа | 98 |  |
|  |  |  |  |
| **E9.** | **Приходится ли Вам останавливаться для того, чтобы перевести дыхание, когда Вы идете по ровной поверхности в обычном для Вас темпе?** | | |
|  | Да | 1 |  |
|  | Нет | 2 | *Переход к E12* |
|  | Никогда не хожу в своём темпе по ровной дороге | 3 |  |
|  | Затрудняюсь ответить | 97 |  |
|  | Отказ от ответа | 98 |  |
|  |  |  |  |
| **E10.** | **Приходилось ли Вам останавливаться из-за одышки после 100 метров (или нескольких минут) ходьбы по ровной дороге?** | | |
|  | Да | 1 |  |
|  | Нет | 2 | *Переход к E12* |
|  | Никогда не хожу100 м | 3 |  |
|  | Затрудняюсь ответить | 97 |  |
|  | Отказ от ответа | 98 |  |
|  |  |  |  |
| **E11.** | **Появляется ли у Вас одышка при раздевании или чувство, что из-за одышки не можете выйти из своего дома?** | | |
|  | Да | 1 |  |
|  | Нет | 2 |  |
|  | Затрудняюсь ответить | 97 |  |
|  | Отказ от ответа | 98 |  |
|  |  |  |  |
| **E12.** | **Возникали ли у Вас приступы свистящего дыхания/хрипов или свист в груди в течение последних 12 месяцев?** | | |
|  | Да | 1 |  |
|  | Нет | 2 |  |
|  | Затрудняюсь ответить | 97 |  |
|  | Отказ от ответа | 98 |  |
|  |  |  |  |
| **E13.** | **В течение последних 12 месяцев просыпались ли Вы ночью из-за приступа удушья?** | | |
|  | Да | 1 |  |
|  | Нет | 2 |  |
|  | Затрудняюсь ответить | 97 |  |
|  | Отказ от ответа | 98 |  |

| **Модуль F** | **ДРУГИЕ ЗАБОЛЕВАНИЯ** |
| --- | --- |

|  | **Теперь я задам Вам вопросы, касающиеся других систем организма** | | | |
| --- | --- | --- | --- | --- |
| **F1.** | **Имеются ли у Вас какие-либо заболевания печени, диагностированные врачом?** | | | |
|  | Да | | 1 |  |
|  | Нет | | 2 | *Переход к F4* |
|  | Затрудняюсь ответить | | 97 | *Переход к F4* |
|  | Отказ от ответа | | 98 | *Переход к F4* |
|  |  | |  |  |
| **F2.** | **Какие это заболевания печени?** *Возможны несколько вариантов ответа.* *Интервьюер, если респондент ответил "желтухa (но без диагноза) ", отметьте Другое.* | | | |
|  | Гепатит | | 1 |  |
|  | Oжирение печени | | 2 |  |
|  | Цирроз печени | | 3 |  |
|  | Рак печени | | 4 |  |
|  | Другое | | 5 |  |
|  | Затрудняюсь ответить | | 97 |  |
|  | Отказ от ответа | | 98 |  |
|  |  | |  |  |
| **F3** | **Какие возможные причины Вашего заболевания печени указал врач?** *Возможны несколько вариантов ответa.* | | | |
|  | Алкоголь | | 1 |  |
|  | Избыточный вес | | 2 |  |
|  | Вирус | | 3 |  |
|  | Другое | | 4 |  |
|  | Затрудняюсь ответить | | 97 |  |
|  | Отказ от ответа | | 98 |  |
|  |  | |  |  |
| **F4.** | **Был ли у вас когда-нибудь диагностирован рак?** | | | |
|  | Да | | 1 |  |
|  | Нет | | 2 | *Переход к F6* |
|  | Затрудняюсь ответить | | 97 | *Переход к F6* |
|  | Отказ от ответа | | 98 | *Переход к F6* |
|  |  | |  |  |
| **F5** | | **В какой части тела было выявлено онкологическое заболевание / рак?** | | |
|  | | Затрудняюсь ответить | 97 |  |
|  | | Отказ от ответа | 98 |  |
|  | |  |  |  |
| **F6.** | | **Обращались ли Вы в течение последних 12 месяцев к врачу по поводу травм/физических повреждений?** *Здесь подразумеваются только физические, а не психологические травмы.* | | |
|  | | Да | 1 |  |
|  | | Нет | 2 | *Переход к G для женщин или H для мужчин* |
|  | | Затрудняюсь ответить | 97 | *Переход к G для женщин  или H для мужчин* |
|  | | Отказ от ответа | 98 | *Переход к G для женщин или H для мужчин* |
| **F7.** | | **Каков был вид травмы?** *(Возможны несколько вариантов ответа)* | | |
|  | | Перелом | 1 |  |
|  | | Синяк, растяжение связок, вывих | 2 |  |
|  | | Порез, укус, открытая рана | 3 |  |
|  |  | Сотрясение | 4 |  |
|  | | Другое | 5 |  |
|  | | Затрудняюсь ответить | 97 |  |
|  | | Отказ от ответа | 98 |  |

| **Модуль G** | **ВОПРОСЫ ТОЛЬКО ДЛЯ ЖЕНЩИН** |
| --- | --- |

*Пожалуйста, установите пол участника, как это будет определять, какие вопросы будут заданы в следующем (следует раздел предназначен только для женщин-участниц, и будут пропускаться для участников мужского пола). Для респондентов мужского пола переход в раздел H.*

| **G1.** | **Были ли Вы когда-либо беременны?** | | | | | | | | | | | | | | | | | | | |  |
| --- | --- | --- | --- | --- | --- | --- | --- | --- | --- | --- | --- | --- | --- | --- | --- | --- | --- | --- | --- | --- | --- |
|  | Да | | | | | | | | | | | 1 | |  | | | | | | |  |
|  | Нет | | | | | | | | | | | 2 | | *Переход к G6* | | | | | | |  |
|  | Затрудняюсь ответить | | | | | | | | | | | 97 | |  | | | | | | |  |
|  | Отказ от ответа | | | | | | | | | | | 98 | |  | | | | | | |  |
| **G2.** | **Сколько было у Вас беременностей?** *Введите количество беременностей* | | | | | | | | | | | | | | | | | | | |  |
|  |  | | | | | | | |  | | | | | | | | | | | |  |
| **G3.** | **В течение всей жизни сколько у Вас было:** | | | | | | | | | | | | | | | | | | | |  |
|  | Беременностей, закончившихся рождением ребёнка | | | | | | | |  | | | | | | | | | | | |  |
|  |  | | | | | | | |  | | | | | | | | | | | |  |
|  | Мертворождений (на сроке 28+недель) | | | | | | | |  | | | | | | | | | | | |  |
|  |  | | | | | | | |  | | | | | | | | | | | |  |
|  | Выкидышей (на сроке менее чем 14 недель) | | | | | | | |  | | | | | | | | | | | |  |
|  |  | | | | | | | |  | | | | | | | | | | | |  |
|  | Выкидышей (на сроке 14-27 недель) | | | | | | | |  | | | | | | | | | | | |  |
|  | Затрудняюсь ответить | | | | | | | | 97 | | | | | | | | | | | |  |
|  | Отказ от ответа | | | | | | | | 98 | | | | | | | | | | | |  |
|  |  | | | | | | | |  | | | | | | | | | | | |  |
| **G4.** | **В данный момент Вы беременны или думаете, что беременны?** | | | | | | | | | | | | | | | | | | | |  |
|  | Да | | | | | | | | | | | 1 | |  | | | | | | |  |
|  | Нет | | | | | | | | | | | 2 | |  | | | | | | |  |
|  | Затрудняюсь ответить | | | | | | | | | | | 97 | |  | | | | | | |  |
|  | Отказ от ответа | | | | | | | | | | | 98 | |  | | | | | | |  |
|  |  | | | | | | | | | | |  | |  | | | | | | |  |
| **G5a.** | **Во время Ваших беременностей прошлых или настоящей говорил ли Вам врач, что у Вас повышенное давление во время беременности (гипертония беременных)?** | | | | | | | | | | | | | | | | | | | |  |
|  | Да | | | | | | | | | | | 1 | |  | | | | | | |  |
|  | Нет | | | | | | | | | | | 2 | |  | | | | | | |  |
|  | Затрудняюсь ответить | | | | | | | | | | | 97 | |  | | | | | | |  |
|  | Отказ от ответа | | | | | | | | | | | 98 | |  | | | | | | |  |
|  |  | | | | | | | | | | |  | |  | | | | | | |  |
| **G5b.** | **Во время Ваших беременностей прошлых или настоящей говорил ли Вам врач, что у Вас гестационный диабет, выявленный во время беременности?** | | | | | | | | | | | | | | | | | | | |  |
|  | Да | | | | | | | | | | | 1 | |  | | | | | | |  |
|  | Нет | | | | | | | | | | | 2 | |  | | | | | | |  |
|  | Затрудняюсь ответить | | | | | | | | | | | 97 | |  | | | | | | |  |
|  | Отказ от ответа | | | | | | | | | | | 98 | |  | | | | | | |  |
|  |  | | | | | | | | | | |  | |  | | | | | | |  |
| **G5c.** | **Во время Ваших беременностей прошлых или настоящей говорил ли Вам врач, что у Вас** [**преэклампсия**](http://surgeryzone.net/info/informaciya-po-akusherstvu-i-ginekologii/preeklampsiya.html)**?** *Преэкламсия - это осложнение беременности, проявляющееся значительным повышением артериального давления, появлением отеков и судорог с потерей сознания* | | | | | | | | | | | | | | | | | | | |  |
|  | Да | | | | | | | | | | | 1 | |  | | | | | | |  |
|  | Нет | | | | | | | | | | | 2 | |  | | | | | | |  |
|  | Затрудняюсь ответить | | | | | | | | | | | 97 | |  | | | | | | |  |
|  | Отказ от ответа | | | | | | | | | | | 98 | |  | | | | | | |  |
|  |  | | | | | | | | | | |  | |  | | | | | | |  |
| **G6.** | **Сколько Вам было лет, когда у Вас начались месячные?** | | | | | | | | | | | | | | | | | | | |  |
|  |  | | | | | | | | | | |  | | | | | | | | |  |
|  |  | | | |  | |  | | | | | Лет | | | | | | | | |  |
|  | Затрудняюсь ответить | | | | | | | | | | | 97 | | | | | | | | |  |
|  | Отказ от ответа | | | | | | | | | | | 98 | | | | | | | | |  |
|  |  | | | | | | | | | | |  | | | | | | | | |  |
| **G7.** | **Была ли у Вас менструация в последние 12 месяцев?** | | | | | | | | | | | | | | | | | | | |  |
|  | Да | | | | | | | | | | | 1 | | *Переход к G9* | | | | | | |  |
|  | Нет | | | | | | | | | | | 2 | |  | | | | | | |  |
|  | Затрудняюсь ответить | | | | | | | | | | | 97 | |  | | | | | | |  |
|  | Отказ от ответа | | | | | | | | | | | 98 | |  | | | | | | |  |
|  |  | | | | | | | | | | |  | |  | | | | | | |  |
| **G8.** | **Каковы причины прекращения менструации:** | | | | | | | | | | | | | | | | | | | |  |
|  | Хирургическое вмешательство/операция | | | | | | | | | 1 | | | | | |  | | | | |  |
|  | Химио- или радио-терапия | | | | | | | | | 2 | | | | | |  | | | | |  |
|  | Беременность или грудное вскармливание | | | | | | | | | 3 | | | | | |  | | | | |  |
|  | Естественное прекращение месячных | | | | | | | | | 4 | | | | | |  | | | | |  |
|  | Другое | | | | | | | | | 5 | | | | | |  | | | | |  |
|  | Затрудняюсь ответить | | | | | | | | | 97 | | | | | |  | | | | |  |
|  | Отказ от ответа | | | | | | | | | 98 | | | | | |  | | | | |  |
|  |  | | | | | | | | |  | | | | | |  | | | | |  |
| **G9.** | **Когда у Вас были месячные в последний раз?** | | | | | | | | | | | | | | | | | | | |  |
|  |  | | | | | | | | | | |  | | | | | | | | |  |
|  |  |  |  | Месяц | | | | | | |  | |  | | | |  |  | Год |  |  |
|  | Затрудняюсь ответить | | | | | | | | | | | 97 | | | | | | | | |  |
|  | Отказ от ответа | | | | | | | | | | | 98 | | | | | | | | |  |
|  |  | | | | | | | | | | |  | | | | | | | | |  |
| **G9a.** | **Если Вы не помните точной даты, сколько Вам было лет в тот момент?**_____ Лет | | | | | | | | | | | | | | | | | | | |  |
|  | Затрудняюсь ответить | | | | | | | | | | | 97 | | | | | | | | |  |
|  | Отказ от ответа | | | | | | | | | | | 98 | | | | | | | | |  |
|  |  | | | | | | | | | | |  | | | | | | | | |  |
| **G10.** | **Принимали ли Вы когда-либо гормональную заместительную терапию?** *(Этот вопрос не относится к гормональным препаратам, принимаемым для контрацепции. Уточните это респондентам.)* | | | | | | | | | | | | | | | | | | | |  |
|  | Да | | | | | | | | | | 1 | | | | |  | | | | |  |
|  | Нет | | | | | | | | | | 2 | | | | | *Переход к разделу H* | | | | |  |
|  | затрудняюсь ответить | | | | | | | | | | 97 | | | | |  | | | | |  |
|  | отказ от ответа | | | | | | | | | | 98 | | | | |  | | | | |  |
| **G11.** | **Когда Вы впервые начали принимать гормональную заместительную терапию?** | | | | | | | | | | | | | | | | | | | |  |
|  |  | | | | | | | | | | |  | | | | | | | | |  |
|  |  |  |  | Месяц | | | | | | |  | |  | | | |  |  | Год |  |  |
|  | Затрудняюсь ответить | | | | | | | | | | | 97 | | | | | | | | |  |
|  | Отказ от ответа | | | | | | | | | | | 98 | | | | | | | | |  |
|  |  | | | | | | | | | | |  | | | | | | | | |  |
|  | **Если Вы не помните точной даты, сколько Вам было лет в тот момент.** *Пожалуйста, укажите возраст в годах* | | | | | | | | | | | | | | | | | | | |  |
|  |  | | | | | | | | | | |  | | | | | | | | |  |
|  |  | | | | |  | |  | | | | Лет | | | | | | | | |  |
| **G11a.** | Затрудняюсь ответить | | | | | | | | | | | 97 | | | | | | | | |  |
|  | Отказ от ответа | | | | | | | | | | | 98 | | | | | | | | |  |
|  |  | | | | | | | | | | |  | | | | | | | | |  |
| **G12.** | **Принимаете ли Вы в настоящее время гормональную заместительную терапию?** | | | | | | | | | | | | | | | | | | | |  |
|  | Да | | | | | | | | | | | 1 | | | *Переход к разделу H* | | | | | |  |
|  | Нет | | | | | | | | | | | 2 | | |  | | | | | |  |
|  | Затрудняюсь ответить | | | | | | | | | | | 97 | | |  | | | | | |  |
|  | Отказ от ответа | | | | | | | | | | | 98 | | |  | | | | | |  |
|  |  | | | | | | | | | | |  | | |  | | | | | |  |
| **G13.** | **Когда Вы прекратили принимать гормональную заместительную терапию?** | | | | | | | | | | | | | | | | | | | |  |
|  |  | | | | | | | | | | |  | | | | | | | | |  |
|  |  |  |  | Месяц | | | | | | |  | |  | | | |  |  | Год |  |  |
|  | Затрудняюсь ответить | | | | | | | | | | | 97 | | | | | | | | |  |
|  | Отказ от ответа | | | | | | | | | | | 98 | | | | | | | | |  |
|  |  | | | | | | | | | | |  | | | | | | | | |  |
| **G13a.** | **Если Вы не помните точной даты, сколько Вам было лет в тот момент?** | | | | | | | | | | | | | | | | | | | |  |
|  |  | | | | |  | |  | | | | Лет | | | | | | | | |  |
|  | Затрудняюсь ответить  Отказ от ответа | | | | | | | | | | | 97 | | | | | | | | |  |
|  |  |  |  |  |  |  |  |  |  |  |  | 98 | | | | | | | | |  |

| **Модуль H** | | **ВОПРОСЫ ПО УПОТРЕБЛЕНИЮ АЛКОГОЛЯ** | |  |
| --- | --- | --- | --- | --- |

**Теперь я задам Вам несколько вопросов о Вашем употреблении алкоголя в последние 12 месяцев. Поскольку употребление алкоголя может повлиять на разные аспекты здоровья и может сказываться на приеме некоторых препаратов, для нас важно, чтобы Ваши ответы были как можно более точными.**

**Сначала я задам несколько вопросов о количестве алкогольных напитков или "порциях", которые Вы могли употребить. Один напиток или порция равна** *Покажите карточку*:

**-250 мл некрепкого пива (4-6% алкоголя)**

**-150 мл крепкого пива (7-9% алкоголя)**

**-100 мл столового, некрепленого вина (11-13% алкоголя)**

**- 60 мл крепленого вина (18-22% алкоголя)**

**- 30 мл водки или других крепких напитков (40% алкоголя)**

| **H1.** | **Как часто вы выпиваете порцию напитка, содержащего алкоголь?** | | | |
| --- | --- | --- | --- | --- |
|  | Никогда | | 1 | *Переход к H9* |
|  | Раз в месяц или реже | | 2 |  |
|  | 2-4 раза в месяц | | 3 |  |
|  | 2-3 раза в неделю | | 4 |  |
|  | 4 или более раз в неделю | | 5 |  |
|  | Затрудняюсь ответить | | 97 |  |
|  | Отказ от ответа | | 98 |  |
|  |  | |  |  |
| **H2.** | **Сколько стандартных порций напитков, содержащих алкоголь, Вы выпиваете в типичный день, когда пьете?** | | | |
|  | 1 или 2 | | 1 |  |
|  | 3 или 4 | | 2 |  |
|  | 5 или 6 | | 3 |  |
|  | 7 ,8 или 9 | | 4 |  |
|  | 10 или более | | 5 |  |
|  | Затрудняюсь ответить | | 97 |  |
|  | Отказ от ответа | | 98 |  |
|  |  | |  |  |
| **H3.** | **Как часто Вы за один случай выпиваете шесть или более порций?** *Напомните участнику, что 6 порций равны примерно трём банкам пива по 500 мл, трём большим бокалам вина (200 мл) или 180 мл водки (три с небольшим рюмок по 50 мл).* | | | |
|  | Никогда | | 1 |  |
|  | Реже, чем 1 раз в месяц | | 2 |  |
|  | Ежемесячно | | 3 |  |
|  | Еженедельно | | 4 |  |
|  | Ежедневно или почти ежедневно | | 5 |  |
|  | Затрудняюсь ответить | | 97 |  |
|  | Отказ от ответа | | 98 |  |
|  |  | |  |  |
| **H4.** | **Как часто за последний год у Вас бывало, что Вы не могли остановиться, начав пить?** | | | |
|  | Никогда | | 1 |  |
|  | Реже, чем 1 раз в месяц | | 2 |  |
|  | Ежемесячно | | 3 |  |
|  | Еженедельно | | 4 |  |
|  | Ежедневно или почти ежедневно | | 5 |  |
|  | Затрудняюсь ответить | | 97 |  |
|  | Отказ от ответа | | 98 |  |
|  |  | |  |  |
| **H5.** | **Как часто за последний год из-за выпивки Вы не делали того, что от Вас обычно ожидалось?** | | | |
|  | Никогда | | 1 |  |
|  | Реже, чем 1 раз в месяц | | 2 |  |
|  | Ежемесячно | | 3 |  |
|  | Еженедельно | | 4 |  |
|  | Ежедневно или почти ежедневно | | 5 |  |
|  | Затрудняюсь ответить | | 97 |  |
|  | Отказ от ответа | | 98 |  |
|  |  | |  |  |
| **H6.** | **Как часто за последний год Вам необходимо было выпить с утра, чтобы прийти в себя после того, как вы много пили накануне?** | | | |
|  |  | | | |
|  | Никогда | | 1 |  |
|  | Реже, чем 1 раз в месяц | | 2 |  |
|  | Ежемесячно | | 3 |  |
|  | Еженедельно | | 4 |  |
|  | Ежедневно или почти ежедневно | | 5 |  |
|  | Затрудняюсь ответить | | 97 |  |
|  | Отказ от ответа | | 98 |  |
|  |  | |  |  |
| **H7.** | **Как часто за последний год у Вас было чувство вины или угрызений совести после выпивки?** | | | |
|  | Никогда | | 1 |  |
|  | Реже, чем 1 раз в месяц | | 2 |  |
|  | Ежемесячно | | 3 |  |
|  | Еженедельно | | 4 |  |
|  | Ежедневно или почти ежедневно | | 5 |  |
|  | Затрудняюсь ответить | | 97 |  |
|  | Отказ от ответа | | 98 |  |
|  |  |  | | |
| **H8.** | **Как часто за последний год Вы не могли вспомнить, что было накануне вечером, из-за того, что Вы выпивали?** | | | |
|  | Никогда | | 1 |  |
|  | Реже, чем 1 раз в месяц | | 2 |  |
|  | Ежемесячно | | 3 |  |
|  | Еженедельно | | 4 |  |
|  | Ежедневно или почти ежедневно | | 5 |  |
|  | Затрудняюсь ответить | | 97 |  |
|  | Отказ от ответа | | 98 |  |
|  |  |  | | |
| **H9.** | **Получали ли Вы или кто-нибудь другой травму в результате того, что Вы выпивали?** | | | |
|  | Нет | 1 | |  |
|  | Да, но не за последний год | 2 | |  |
|  | Да, в течение последнего года | 3 | |  |
|  | Затрудняюсь ответить | 97 | |  |
|  | Отказ от ответа | 98 | |  |
|  |  |  | |  |
| **H10.** | **Случалось ли, что Ваш родственник, друг, врач или какой-либо другой медицинский работник высказывали озабоченность по поводу Вашего употребления алкоголя или предлагали Вам пить меньше?** | | | |
|  |  | | | |
|  | Нет | 1 | |  |
|  | Да, но не за последний год | 2 | |  |
|  | Да, в течение последнего года | 3 | |  |
|  | Затрудняюсь ответить | 97 | |  |
|  | Отказ от ответа | 98 | |  |

| **Модуль I** | **КУРЕНИЕ** |
| --- | --- |

**Сейчас я задам несколько вопросов о курении**

| **I1.** | **Курите ли Вы в настоящее время?** | | |
| --- | --- | --- | --- |
|  | Нет и никогда не курил (а) | 1 | *Переход к J1* |
|  | Нет, бросил (а) | 2 |  |
|  | Да, курю регулярно | 3 | *Переход к I3* |
|  | Да, я курю, но менее 1 сигареты в день | 4 | *Переход к I3* |
|  | Затрудняюсь ответить | 97 | *Переход к I3* |
|  | Отказ от ответа | 98 | *Переход к J1* |
|  |  |  |  |
| **I2.** | **Как давно Вы бросили курить?** *Только один вариант ответа*. | | |
|  | Не более 1 года назад | 1 |  |
|  | От 1 до 5 лет назад | 2 |  |
|  | От 5 до 10 лет назад | 3 |  |
|  | более 10 лет назад | 4 |  |
|  | Затрудняюсь ответить | 97 |  |
|  | Отказ от ответа | 98 | *Переход к J1* |
|  |  |  |  |
| **I3.** | **Что Вы курите (курили) чаще всего?** *Только один вариант ответа*. | | |
|  | Папиросы | 1 |  |
|  | Сигареты с фильтром | 2 |  |
|  | Сигареты без фильтра | 3 |  |
|  | Другое | 4 |  |
|  | Другое (уточните) | 4a |  |
|  | Затрудняюсь ответить | 97 |  |
|  | Отказ от ответа | 98 |  |
|  |  |  |  |
| **I4.** | **Когда Вы курите (курили), сколько Вы обычно выкуриваете (выкуривали) за день?** | | |
|  | 10 сигарет и менее | 1 |  |
|  | Более 10, но не более 20 сигарет | 2 |  |
|  | Более 20-сигарет | 3 |  |
|  | Меньше 1 сигареты в день, нерегулярно | 4 | *Переход к J1* |
|  | Затрудняюсь ответить | 97 |  |
|  | Отказ от ответа | 98 |  |
|  |  |  |  |
| **I5.** | **В каком возрасте Вы начали курить регулярно?** лет_________________ | | |
|  | Затрудняюсь ответить | 97 |  |
|  | Отказ от ответа | 98 |  |
|  |  |  |  |

**I6. Введите любые комментарии, которые вы считаете необходимыми, в связи с опросом (например, если участник плохо слышит и ему приходилось смотреть на экран)__________**

| **Модуль L** | **ОКРУЖНОСТЬ ТАЛИИ, БЕДЕР, РОСТ, ВЕС И СОСТАВ ТЕЛА** |
| --- | --- |

| **L1. Талия 1 (в сантиметрах до ближайшего мм)** | | | | | | | | | | | | |
| --- | --- | --- | --- | --- | --- | --- | --- | --- | --- | --- | --- | --- |
|  | | | | | | | | | | | | |
|  | |  | |  | |  | | **,** | |  | | см |
|  | |  | |  | |  | |  | |  | |  |
| *Если измерение не проводилось, пожалуйста, укажите "0" в текстовом поле.* | | | | | | | | | | | | |
| **L2. Бедра 1 (в сантиметрах до ближайшего мм)** | | | | | | | | | | | | |
|  | | | | | | | | | | | | |
|  | |  | |  | |  | | **,** | |  | | см |
|  | |  | |  | |  | |  | |  | |  |
| *Если измерение не проводилось, пожалуйста, укажите "0" в текстовом поле.* | | | | | | | | | | | | |
| **L3. Талия 2 (в сантиметрах до ближайшего мм)** | | | | | | | | | | | | |
|  | | | | | | | | | | | | |
|  | |  | |  | |  | | **,** | |  | | см |
|  | |  | |  | |  | |  | |  | |  |
| *Если измерение не проводилось, пожалуйста, укажите "0" в текстовом поле.* | | | | | | | | | | | | |
| **L4. Бедра 2 (в сантиметрах до ближайшего мм)** | | | | | | | | | | | | |
|  | | | | | | | | | | | | |
|  | |  | |  | |  | | **,** | |  | | см |
|  | |  | |  | |  | |  | |  | |  |
| *Если измерение не проводилось, пожалуйста, укажите "0" в текстовом поле.* | | | | | | | | | | | | |
| **L5.** | **Рост 1 (в сантиметрах до ближайшего мм)** | | | | | | | | | | | |
|  |  | | | | | | | | | | | |
|  |  |  |  | |  | | **.** | |  | | см | |
|  |  |  |  | |  | |  | |  | |  | |
|  | *Если измерение не проводилось, пожалуйста, укажите "0" в текстовом поле.* | | | | | | | | | | | |
|  | *Попросите пациента пройтись по комнате между каждым измерением роста.* | | | | | | | | | | | |
| **L6.** | **Рост 2 (в сантиметрах до ближайшего мм)** | | | | | | | | | | | |
|  |  | | | | | | | | | | | |
|  |  |  |  | |  | | **.** | |  | | см | |
|  | *Если измерение не проводилось, пожалуйста, укажите "0" в текстовом поле.* | | | | | | | | | | | |

| **L7.** | **Анализ состава тела.**  **Теперь я собираюсь произвести исследование с использованием анализатора состава тела "Tanita"**.  *Сообщите участнику, что противопоказаниями для проведения анализа состава тела являются беременность и наличие кардиостимулятора*. *При отсутствии противопоказаний и согласии участника на проведение анализа состава тела используйте режим «состав тела» анализатора “Tanita” (стандартная процедура). При наличии противопоказаний или отказе участника от анализа состава тела переведите анализатор “Tanita” в режим весов (альтернативный метод). Поясните участнику, что в использование анализатора “Tanita” режиме весов не несет никаких рисков для беременных женщин и лиц с кардиостимуляторами.* | | | | | | | | |
| --- | --- | --- | --- | --- | --- | --- | --- | --- | --- |
| **L7a.** | Анализ состава тела проведен | | | | | | 1 | |  |
|  | Анализ состава тела не проведен (кардиостимулятор) | | | | | | 2 | |  |
|  | Анализ состава тела не проведен (беременность) | | | | | | 3 | |  |
|  | Анализ состава тела не проведен (другая причина) | | | | | | 4 | |  |
|  |  | | | | | |  | |  |
|  | *Если измерение не проводилось, пожалуйста, укажите ноль (0) в текстовом поле. Проведите измерение с помощью Tanita (следующий пункт), если выбран первый вариант.* | | | | | | | | |
|  | *Сохраните файл данных участника для анализатора состава тела "Tanita". Прежде чем продолжить, убедитесь, что измерение было успешно произведено и данные участника были экспортированы. После этого нажмите "Далее", чтобы продолжить и собрать данные в систему.* | | | | | | | | |
| **L7c.** | **Интервьюер - измерение успешно произведено, данные экспортированы и собраны?** *Отвечайте "Да", только если сбор данных на предыдущем экране был успешным!* | | | | | | | | |
|  | Да | | | | | | 1 | |  |
|  | Нет | | | | | | 2 | | *Переход к L7d* |
|  |  | | | | | |  | |  |
| **L7d.** | **Почему измерение с помощью “Танита” не было успешным?** | | | | | | | | |
|  |  | | | | | | | | |
|  | Участник отказался | | | | | | 1 | |  |
|  | Не применимо к участнику | | | | | | 2 | |  |
|  | Не удалось экспортировать данные | | | | | | 3 | |  |
|  | Не удалось собрать данные | | | | | | 4 | |  |
|  | Другое | | | | | | 5 | | *Переход к L7e* |
|  |  | | | | | |  | |  |
| **L7e.** | **Пожалуйста, объясните, почему измерение с помощью "Танита" не было успешным**________________________________________________________ | | | | | | | | |
|  |  | | | | | | | | |
| **L7b.** | **Вес 1 (до ближайшего 0,1 кг)** *В случае если L7=2,3,4* | | | | | | | | |
|  |  | | | | | | | | |
|  |  |  |  |  | **.** |  | | кг | |

| **Модуль О** | **ЭКГ И ВИКОРДЕР** |
| --- | --- |

| **T1.** | **Температура в кабинете (до ближайшего 0,1ºC)** | | | | | |
| --- | --- | --- | --- | --- | --- | --- |
|  |  |  |  | , |  | ºС |
|  |  | |  | | | |

**Сейчас я собираюсь оценить работу вашего сердца методом электрокардиограммы с помощью устройства Cardiax**.

*Объясните детали участнику, если требуется. Настройте оборудование в соответствии с требованиями и приступайте, когда будете готовы.*

**Инструкция по проведению обследования с помощью устройства Cardiax.**

1. Подключите кабель устройства к ноутбуку.

2. Запустите программное обеспечение Cardiax.

3. Разместите электроды на груди участника в соответствии с инструкцией.

4. Сделайте измерения.

5. Экспорт результаты измерения в формате CSV.

6. Сохранить ЭКГ с форматом PDF.

**M1.** *Сохраните файл (ы) данных участника для обследования с помощью Cardiax.*

*Прежде чем продолжить, убедитесь, что измерение было успешно произведено и данные участника были экспортированы. После этого нажмите "Далее", чтобы продолжить и собрать данные в систему.*

**M1_2.** *Сохраните PDF файл (ы) данных участника для обследования с помощью Cardiax.*

| **O1a.** | **Интервьюер - измерение успешно произведено, данные экспортированы и собраны?** *Отвечайте "Да", только если сбор данных на предыдущем экране был успешным!* | |
| --- | --- | --- |
|  | Да | 1 |
|  | Нет | 2 *Переход к O1b* |
|  |  |  |
| **M1ab.** | *Пожалуйста, дайте краткое заключение по результатам ЭКГ (для последующей выдачи участнику вместе с другими результатами обследования).* | |
| **O1b.** | **Почему измерение с помощью Cardiax не было успешным?** | |
|  | Нет, участник отказался | 1 |
|  | Не применимо к данному респонденту | 2 |
|  | Данные не экспортированы | 3 |
|  | Данные не собраны | 4 |
|  | Другое | 5 *Переход к O1c* |
|  |  |  |
| **O1c Пожалуйста, объясните, почему измерение с помощью Cardiax не было успешным?________** | | |

**Викодер**

| **T4.** | **Температура в кабинете (до ближайшего 0,1ºC)** | | | | | |
| --- | --- | --- | --- | --- | --- | --- |
|  |  |  |  | , |  | ºС |

**Теперь я проведу измерение скорости распроранения пульсовой волны (скорости движения крови по Вашим сосудам) с помощью устройства «Викордер».**

*Объясните детали к участнику, если требуется.Произведите настройку оборудования в соответствии с требованиями и приступайте, когда будете готовы.Запишите три результата измерения скорости распространения пульсовой волны, результат одного измерения артериального давления и три результата проведения анализа пульсовой волны.*

**O2e.** **Курил ли участник в последние 3 часа?**

1. Да
2. Нет

**O2f. Укажите расстояние от яремной ямки до нижней части шейной манжеты в см до ближайшего мм.** *Расстояние от яремной ямки до ВЕРХНЕЙ части бедренной манжеты вводится непосредственно в программу устройства «Викордер».*

cm

**O2g. Укажите расстояние от плеча на уровне яремной ямки на верхней части бедренной манжеты** *(вводится только если у участника сильно выступает живот) в см до ближайшего мм. Расстояние от яремной ямки до ВЕРХНЕЙ части бедренной манжеты вводится непосредственно в программу устройства «Викордер».*

. cm

**O2. Соберите файл данных для участника для измерения ВИКОРДЕР.** *Прежде чем продолжить, убедитесь, что измерение было успешно произведено и данные участника были экспортированы. После этого нажмите "Далее", чтобы продолжить и собрать данные в систему.*

**O2_1. Соберите файл данных для участника для измерения ВИКОРДЕР IMG.**

*Прежде чем продолжить, убедитесь, что измерение было успешно произведено и данные участника были экспортированы. После этого нажмите "Далее", чтобы продолжить и собрать данные в систему.*

**O2h. Перемещалась ли феморальная или каротидная манжета во время обследования?**

1. Да *Переход к* *O2_i*
2. Нет *Переход к* *O2_b*

**O2i. Если манжета перемещалась, укажите результаты нового измерения расстояния от яремной ямки до верха бедренной манжеты.**

cm

**O2j.** **Если манжета перемещалась, укажите результаты нового измерения расстояния от яремной ямки до низа каротидной манжеты.**

. cm

| **O2b.** | **Интервьюер - измерение успешно произведено, данные экспортированы и собраны?** *Отвечайте "Да", только если сбор данных на предыдущем экране был успешным!* | |
| --- | --- | --- |
|  | Да | 1 |
|  | Нет | 2 *Переход к O2c* |
|  |  |  |
| **O2c.** | **Почему измерение с помощью устройства Vicorder не было успешным?** | |
|  | Нет, участник отказался | 1 |
|  | Не применимо к данному респонденту | 2 |
|  | Данные не экспортированы | 3 |
|  | Данные не собраны | 4 |
|  | Другое | 5 *Переход к O2d* |
|  |  |  |
| **O2d.** | **Пожалуйста, объясните, почему измерение с помощью Vicorder не было успешным?**_______ | |
|  |  | |
| **O2k.** | **Введите любые комментарии, которые вы считаете необходимыми, в связи с обследованием при помощи устройства «Викордер»**_______ | |

| **Модуль О3** | **АКТИХАРТ** |
| --- | --- |

| **P5a.** | **Вы хотели бы, чтобы Вам установили и настроили устройство Actiheart для измерения физической активности?** | | | | | | | | | | | | | | | |
| --- | --- | --- | --- | --- | --- | --- | --- | --- | --- | --- | --- | --- | --- | --- | --- | --- |
|  | Да | | | | | | | | | | 1 | | | | | |
|  | Нет | | | | | | | | | | 2 | | | | | |
| *Объясните детали участнику, если требуется. Настройте оборудование в соответствии с требованиями и приступайте, когда будете готовы. Перед началом теста "Ходьба, расстояние 200 м в удобном темпе"(для настройки устройства Actiheart) убедитесь, что участник стоял спокойно и не разговаривал, по крайней мере 1 мин до начала этого теста.* | | | | | | | | | | | | | | | | |
| **Сейчас я проведу настройку устройства** | | | | | | | | | | | | | | | | |
|  | | | | | | | | | | | | | | | | |
| **O4.** | | **Реальное время начала теста ходьбы:** | | | | | | _______:_______ | | | | | |  | | |
|  | | |  |  |  | |  | |  |  | |  |  | |  | |
|  | | |  |  |  | |  | |  |  | |  |  | |  | |
| **O5.** | **Продолжительность теста:** | | | | | _______:_______ | | | | | | | | | | |
|  | | |  |  |  | |  | |  |  | |  |  | |  | |
| **O6. Всего завершенных кругов:** | | | | |  | |  | |  |  | |  |  | |  | |
|  | | |  |  |  | |  | |  |  | |  |  | |  | |
| **O6a.** | | **Дистанция, пройденная на незавершенном круге (только для незавершенного круга):** | | | | | | | | | | | | | |  |
|  | |  | | | | | | | | |  | | | | | |
| **O7.** | | **Была необходима помощь/поддержка при ходьбе?** | | | | | | | | |  | | | | | |
|  | | Да | | | | | | | | | 1 | | | | | |
|  | | Нет | | | | | | | | | 2 *Переход к O8* | | | | | |
| **O7a.** | | **Если да, какая (н-р, опоры-ходунки трость**)**?** (укажите)__________________________ | | | | | | | | |  | | | | | |
|  | |  | | | | | | | | |  | | | | | |
| **O8.** | | **Комментарии:** | | | | | | | | |  | | | | | |
| **Я сейчас собираюсь собрать измерения Actiheart.** *Объясните детали участнику, если требуется.* *Настройте оборудование в соответствии с требованиями и приступайте, когда будете готовы.*  **Сбор файл (ы) данных для участника для измерения Actiheart .** *Прежде чем продолжить, убедитесь, что измерение было успешно произведено и данные участника были экспортированы. После этого нажмите "Далее", чтобы продолжить и собрать данные в систему.* | | | | | | | | | | | | | | | | |
| **P5b.** | | **Интервьюер - измерение успешно произведено, данные экспортированы и собраны?** *Отвечайте "Да", только если сбор данных на предыдущем экране был успешным!* | | | | | | | | | | | | | | |
|  | | Да | | | | | | | | | 1 *Переход к P5e* | | | | | |
|  | | Нет | | | | | | | | | 2 *Переход к P5c* | | | | | |
|  | |  | | | | | | | | |  | | | | | |
|  | |  | | | | | | | | | | | | | | |
| **P5c.** | | **Почему сбор данных с помощью Actiheart не был успешным?** | | | | | | | | | | | | | | |
|  | |  | | | | | | | | | | | | | | |
|  | | Участник отказался | | | | | | | | | 1 | | | | | |
|  | | Не применимо к участнику | | | | | | | | | 2 | | | | | |
|  | | Данные не экспортированы | | | | | | | | | 3 | | | | | |
|  | | Данные не собраны | | | | | | | | | 4 | | | | | |
|  | | Другое | | | | | | | | | 5 *Переход к P5d* | | | | | |
|  | |  | | | | | | | | |  | | | | | |
| **P5d.** | | **Пожалуйста, объясните, почему сбор данных с помощью Actiheart не был успешным?** | | | | | | | | | | | | | | |
|  | |  | | | | | | | | |  | | | | | |
| **P5e.** | | **Интервьюер - Устройство Actiheart успешно установлено и настроено?** | | | | | | | | | | | | | | |
|  | | Да | | | | | | | | | 1 | | | | | |
|  | | Нет | | | | | | | | | 2 *Переход к P5f* | | | | | |
| **P5f.** | | **Почему не удалось настроить устройство Actiheart?** | | | | | | | | |  | | | | | |
|  | |  | | | | | | | | |  | | | | | |
|  | | Участник отказался | | | | | | | | | 1 | | | | | |
|  | | Не применимо к участнику | | | | | | | | | 2 | | | | | |
|  | | Калибровка не удалась | | | | | | | | | 3 | | | | | |
|  | | Другое | | | | | | | | | 4 | | | | | |
|  | |  | | | | | | | | | | | | | | |
| **P5g.** | | **Пожалуйста, объясните, почему настройка устройства Actiheart не была успешной?** | | | | | | | | | | | | | | |

| **Модуль P4** \| **МОНИТОРИНГ ЧСС И РАСХОДА ЭНЕРГИИ/СБОР ДАННЫХ** **Я сейчас собираюсь собрать измерения Actiheart.** | | | |
| --- | --- | --- | --- |
| *Объясните детали участнику, если требуется.*  *Настройте оборудование в соответствии с требованиями и приступайте, когда будете готовы.*  **P4. Сбор файл (ы) данных для участника для измерения Actiheart.**  *Прежде чем продолжить, убедитесь, что измерение было успешно произведено и данные участника были экспортированы. После этого нажмите "Далее", чтобы продолжить и собрать данные в систему.* | | | |
| **P4a.** | **Интервьюер - измерение успешно произведено, данные экспортированы и собраны?** *Отвечайте "Да", только если сбор данных на предыдущем экране был успешным!* | | |
|  | Да | | 1 *Переход к P4d* |
|  | Нет | | 2 |
|  |  | |  |
| **P4b.** | **Почему сбор данных с помощью Actiheart не был успешным?** | | |
|  |  | | |
|  | Участник отказался | | 1 |
|  | Не применимо к участнику | | 2 |
|  | Данные не экспортированы | | 3 |
|  | Данные не собраны | | 4 |
|  | Другое | | 5 *Переход к P4c* |
|  |  | |  |
| **P4c.** | **Пожалуйста, объясните, почему сбор данных с помощью Actiheart не был успешным?** | | |
|  |  | | |
| **P4d.** | | **Согласно дневнику, когда участник прекратил носить устройство Actiheart? (дата)** | |
| «____»___________20___г | | | |
|  | | | |
| **P4e.** | | **Согласно дневнику, когда участник прекратил носить устройство Actiheart? (время)** | |
|  | | _______:_______ | |

| Модуль М | ПРОВЕРКА ФИЗИЧЕСКИХ ФУНКЦИЙ |
| --- | --- |

**Теперь мы собираемся выполнить тест на измерение силы сжатия.**

**Сила сжатия**

*В ходе измерения силы сжатия пациент должен сидеть на стуле с подлокотниками. ВАЖНО! Отрегулируйте ручку динамометра таким образом, чтобы она удобно лежала в руке. Она должна находиться на средней части четырех пальцев.*

| **M1a.** | | **Согласился ли пациент на прохождение данного теста?** | | | |  |
| --- | --- | --- | --- | --- | --- | --- |
|  | |  | | | |  |
|  | | Да | | | 1 |  |
|  | | Нет | | | 2 |  |
|  | | Проведение теста невозможно по медицинским причинам | | | 3 |  |
|  | | Проведение теста невозможно по другим причинам (например, отсутствие оборудования) | | | 4 |  |
|  | |  | | |  |  |
| **M1b.** | | **Если пациент не хочет или не может выполнить тест, укажите причину** | | | |  |
| **ЛЕВАЯ РУКА** | | | **ПРАВАЯ РУКА** | | | |
| **М2.** | **Первое измерение (до ближайшего 0,1 кг)** \| \| \| **,** \| \| **кг** Измерение не проводилось 98 | | **М3.** | **Первое измерение (до ближайшего 0,1 кг)** \| \| \| **,** \| \| **кг** Измерение не проводилось 98 | | |
| **М4.** | **Второе измерение (до ближайшего 0,1 кг)** \| \| \| **,** \| \| **кг** Измерение не проводилось 98 | | **М5.** | **Второе измерение (до ближайшего 0,1 кг)** \| \| \| **,** \| \| **кг** Измерение не проводилось 98 | | |
| **М6.** | **Третье измерение (до ближайшего 0,1 кг)** \| \| \| **,** \| \| **кг** Измерение не проводилось 98 | | **М7.** | **Третье измерение (до ближайшего 0,1 кг)** \| \| \| **,** \| \| **кг** Измерение не проводилось 98 | | |

| **M8.** | **Доминирующая рука** | | | |
| --- | --- | --- | --- | --- |
|  | Правая | 1 |  |  |
|  | Левая | 2 |  |  |
|  | Одинаково хорошо владеет (пишет) обеими руками | 3 |  |  |

**Тест на равновесие одна нога**

**Теперь мы собираемся выполнить тест на равновесие.**

|  | **M9a.** | **Согласился ли пациент на прохождение данного теста?** | | | | | | | | | | |
| --- | --- | --- | --- | --- | --- | --- | --- | --- | --- | --- | --- | --- |
|  |  | Да | | | | | | | | 1 | | |
|  |  | Нет | | | | | | | | 2 | | |
|  |  | Проведение теста невозможно по медицинским причинам | | | | | | | | 3 | | |
|  |  | Проведение теста невозможно по другим причинам (например, отсутствие оборудования) | | | | | | | | 4 | | |
|  |  |  | | | | | | | | |  | |
|  | **M9b.** | **Если пациент не хочет или не может выполнить тест, укажите причину**_____ | | | | | | | | | | |
|  |  |  | | | | | | | | | | |
| **M10.** | | | **Тест на равновесие 1, с открытыми глазами.**  **Время, в течение которого пациенту удавалось сохранять равновесие  (с открытыми глазами) до ближайшей 0,1 секунды** | | | | | | | | |  |
|  | | |  | | | | | |  | | |  |
|  | | |  |  |  |  | **.** |  | сек. | | |  |
|  | | | *Если Участник не в состоянии стоять без поддержки, пожалуйста, нажмите "* Отказ от ответа *"* | | | | | | | | |  |
|  | | |  |  |  |  |  |  |  |  |  |  |
|  | | |  | | | | | |  | | |  |
| **M11.** | | | **Тест на равновесие 2, с закрытыми глазами.**  **Время, в течение которого пациенту удавалось сохранять равновесие  (с закрытыми глазами) до ближайшей 0,1 секунды** | | | | | | | | |  |
|  | | |  | | | | | |  | | |  |
|  | | |  |  |  |  | **.** |  | сек. | | |  |
|  | | |  | | | | | |  | | |  |
|  | | | *Если Участник не в состоянии стоять без поддержки, пожалуйста, нажмите "* Отказ от ответа *"* | | | | | | | | |  |

**Тест "Вставание со стула"**

**Теперь мы собираемся выполнить тест "Вставание со стула".**

| **M12a.** | | **Согласился ли пациент на прохождение данного теста?** | | | | | | | | | | | | | | |
| --- | --- | --- | --- | --- | --- | --- | --- | --- | --- | --- | --- | --- | --- | --- | --- | --- |
|  | | Да | | | | | | | | | 1 | | | | | |
|  | | Нет | | | | | | | | | 2 | | | | | |
|  | | Проведение теста невозможно по медицинским причинам | | | | | | | | | 3 | | | | | |
|  | | Проведение теста невозможно по другим причинам (например, отсутствие оборудования) | | | | | | | | | 4 | | | | | |
|  | |  | | | | | | | | | | | | | |  |
| **M12b.** | | **Если пациент не хочет или не может выполнить тест, укажите причину.** | | | | | | | | | | | | | | |
|  |  | | | | | | | | | | | | | | | |
| **M13.** | **Время, затраченное на выполнение теста (ближайшее до 0,1 секунды).** | | | | | | | | | | | | | | | |
|  |  | | | | | | | | | | | | | | | |
|  |  | |  |  |  | | . | |  | | | | | |  | |
|  |  | | | | | | | | | | | | | | | |
|  | *Если Участник не в состоянии стоять без поддержки, пожалуйста, нажмите "Тест не может быть выполнен"* | | | | | | | | | | | | | | | |
| **M14.** | **Количество завершенных вставаний со стула (максимум 10).** | | | | | | | | | | | | | | | |
|  |  | | | | | | | | | | |  | | | | |
|  |  | | | | |  | |  | | | | |  | | | |
|  |  | | | | | | | | | | | | | | | |
|  | *Если Участник не в состоянии стоять без поддержки, пожалуйста, нажмите "Тест не может быть выполнен"* | | | | | | | | | | | | | | | |
| **M15.** | **Если количество завершенных вставаний менее 10, укажите причину**._______ | | | | | | | | | | | | | | | |
|  |  | | | | | | | | | | | | | | | |
|  | Пациент не может стоять без поддержки | | | | | | | | | 1 | | | | | | |
|  | Пациент слишком запыхался | | | | | | | | | 2 | | | | | | |
|  | Пациент остановился и не смог продолжить | | | | | | | | | 3 | | | | | | |
|  | Либо вам, либо пациенту показалось, что выполнение теста небезопасно | | | | | | | | | 4 | | | | | | |
|  | Тест занял более двух минут | | | | | | | | | 5 | | | | | | |
|  | Другая причина | | | | | | | | | 6 | | | |  | | |
| **M15a. Другая причина (уточните)**___________ | | | | | | | | | | | | | | | | |

| Модуль Р | ЭХО И УЗИ СОСУДОВ |
| --- | --- |

| **T5.** | **Температура в кабинете (до ближайшего 0,1ºC)** | | | | | |
| --- | --- | --- | --- | --- | --- | --- |
|  |  |  |  | , |  | ºС |
|  | Измерение не проводилось | | 98 | | | |

| **P1.** | **Ультразвуковое исследование сосудов было завершено** | | |
| --- | --- | --- | --- |
|  |  | | |
|  | Да | 1 |  |
|  | Нет, участник отказался | 2 |  |
|  | Нет, другая причина | 3 | *Переход к P1a* |
|  |  |  |  |
|  | *Пожалуйста, убедитесь, что файлы собраны и сохранены с использованием ID участника в на компьютере с установленной программой EchoPac.* | | |
| **P1b.** | **Пожалуйста, объясните, почему ультразвуковое исследование сосудов не было успешным?**________ | | |
| **P1_1.**  **P1_2** | **Время начала УЗИ сосудов** _______:_______  **Время окончания УЗИ сосудов** _______:_______ |  |  |
| **P3.** | **ЭХО было завершено** |  |  |
|  | Да | 1 |  |
|  | Нет, участник отказался | 2 |  |
|  | Нет, другая причина | 3 | *Переход к P3a* |
|  |  |  |  |
|  | *Пожалуйста, убедитесь, что файлы собраны и сохранены с использованием ID участника в на компьютере с установленной программой EchoPac.* | | |
| **P3a.** | **Пожалуйста, объясните, почему ЭХО не было успешным?**_______ | | |
|  |  | | |
| **P2_1.** | **Время начала ЭХО** _______:_______ | | |
| **P2_2.** | **Время окончания ЭХО** _______:_______ | | |

|  | *Вопросы только для Новосибирска.*  *Пожалуйста, убедитесь, что файлы собраны и сохранены с использованием ID участника в на компьютере с установленной программой EchoPac.* | | |
| --- | --- | --- | --- |
| **P1c.** | **Абдоминальное ультразвуковое исследование успешно произведено?** | | |
|  |  | | |
|  | Да | 1 |  |
|  | Нет, участник отказался | 2 |  |
|  | Нет, другая причина | 3 | *Переход к P1d* |
|  |  |  |  |
| **P1d.** | **Пожалуйста, объясните, почему Абдоминальное ультразвуковое исследование не было успешным?**_______ | | |
|  |  |  |  |

| **Модуль S** | **СПИРОМЕТРИЯ** |
| --- | --- |

| **T6.** | **Температура в кабинете (до ближайшего 0,1ºC)** | | | | | |
| --- | --- | --- | --- | --- | --- | --- |
|  |  |  |  | , |  | ºС |
|  | Измерение не проводилось | | 98 | | | |

| **Теперь мы собираемся выполнить спирометрию.**  *Объясните детали участнику и спросите о желании провести тест.*  *Настройте оборудование в соответствии с требованиями и приступайте, когда будете готовы*. *Противопоказания для спирометрии: инфекция дыхательных путей в предшествующем месяце, отслоение сетчатки в анамнезе, инфаркт миокарда в предшествующем месяце, хирургические операции глаза, груди или живота в последние 3 месяца, беременность 1-го или 3-го триместра, текущее лечение туберкулеза.* | | |
| --- | --- | --- |
| **S1b.** | **Спирометрия выполнена (для подгруппы участников)** | |
|  |  |  |
|  | Да | 1 |
|  | Нет, участник отказался | 2 |
|  | Нет, участнику не предлагалась спирометрия | 3 |
|  | Нет, есть противопоказания | 4 *Переход к* S2 |
|  | Нет, другая причина (пожалуйста, укажите) | 5 *Переход к S1d* |
|  |  |  |
|  |  |  |
| **S1.** | **Сохраните файл данных участника для спирометрии.** *Прежде чем продолжить, убедитесь, что измерение было успешно произведено и данные участника были экспортированы. После этого нажмите "Далее", чтобы продолжить и собрать данные в систему.* | |
| **S1d.** | **Пожалуйста, объясните, почему спирометрия не была успешной** | |
|  |  | |
|  |  |  |
| **S2.** | **Противопоказания к спирометрии** |  |
|  | Инфекции дыхательных путей в предшествующем месяце | 1 |
|  | Отслоение сетчатки в анамнезе | 2 |
|  | Инфаркт миокарда в предшествующем месяце | 3 |
|  | Хирургические операции глаза, груди или живота в последние 3 месяца | 4 |
|  | Беременность, 1-й или 3-й триместр | 5 |
|  | Текущее лечение туберкулеза | 6 |
|  |  |  |
| **S1c.** | **В предшествующий час участник** |  |
|  | Выпил напиток, содержащий кофеин | 1 |
|  | Пользовался ингалятором | 2 |
|  | Курил | 3 |
|  | Не делал ничего из вышеперечисленного | 4 |
