# Supplementary material for: Know Your Heart: Rationale, design and conduct of a cross-sectional study of cardiovascular structure, function and risk factors in 4500 men and women aged 35-69 years from two Russian cities, 2015-18
Source: Wellcome Open Res. 2018 Dec 3;3:67. Originally published 2018 Jun 4. [Version 3] doi: 10.12688/wellcomeopenres.14619.3 (PMC6073094; doi:10.12688/wellcomeopenres.14619.3)
Supplement: Peer review history

# Peer review: Referee response for version 3

**DOI:** 10.21956/wellcomeopenres.16294.r34361

**Published:** 2018-12-04

**Authors:** John Lynch

**Affiliations:** 1 School of Population Health, University of Adelaide, Adelaide, SA, Australia

No new comments

I have read this submission. I believe that I have an appropriate level of expertise to confirm that it is of an acceptable scientific standard.

# Peer review: Referee response for version 2

**DOI:** 10.21956/wellcomeopenres.16005.r33447

**Published:** 2018-08-03

**Authors:** Zhengming Chen

**Affiliations:** 1 Nuffield Department of Population Health, University of Oxford, Oxford, UK

The protocol paper by Sarah Cook and colleagues described rationale, design and conduct of a cross-sectional study in Russia for in-depth investigation into CVD phenotypes and their associations with a wide range of socio-economic, psychological and biological factors. The paper was well structured and well written, with all the necessary details provided and major design, operational and analytic issues discussed. Given the extremely high burden of cardiovascular disease in Russia and lack of proper understanding about underlying risk factors in driving the high disease rates, the project is very timely and will shed new light not only on epidemiology of CVD in Russia but also on roles of emerging and novel risk factors in aetiology of CVD in general.

 The project was well designed and conducted, with for example use of electronic data capture system to minimise data entry error and ensure completeness. Although the overall response rate was not particularly high, which is not uncommon for this type of community-based study, the completeness of the data collection, particularly those related to physical examination and sample collection, among the participants was extremely high. Moreover, most of the samples collected were processed within two hours of the collection. There are also procedures in place for data access and collaboration with wider research community. I only have a few minor comments that may further improve the manuscript.

1. It would be helpful to provide a map of Russia showing the geographical locations of the two cities involved in the study, along with capital city Moscow;
2. In the analysis plan, the authors listed three possible areas of work and mentioned specifically left ventricular ejection fraction but not cIMT/plaque measured by carotid ultrasound. cIMT and plaque are commonly used surrogate endpoints for CVD. As such, it would be helpful to see them highlighted specifically in the paper as a research priority.
3.  Although the study was designed to be a cross-sectional survey, it would be helpful to mention briefly whether there is any plan to follow up participants long-term for cause-specific mortality and other health outcomes. If not, then possible reasons for not doing so (eg, lack of power, problem with linkages) should be discussed.
4. Overall this is a well written paper on a major new study that will throw new light on CVD aetiology and epidemiology in Russia where there is substantial unmet need. I have no reservation in recommending indexing.

I have read this submission. I believe that I have an appropriate level of expertise to confirm that it is of an acceptable scientific standard.

# Response

**Published:** 2018-11-21

**Authors:** Sarah Cook

**Affiliations:** London School of Hygiene & Tropical Medicine, UK

We would like to thank Professor Chen very much for his review.

 In response to his comments we have made the following changes:

1. Map showing the location of the cities has been added (Figure 2)
2. We agree with the comment about the importance of carotid intima media thickness and plaque as surrogate endpoints for CVD have added cIMTand plaque as areas of special interest alongside left ventricular ejection fraction.
3. Discussion on the possibilities for follow up has been added to the discussion section.

# Peer review: Referee response for version 2

**DOI:** 10.21956/wellcomeopenres.16005.r33471

**Published:** 2018-08-01

**Authors:** Filip Zemrak

**Affiliations:** 1 St. Bartholomew's Hospital, London, UK

Thank you for the clarifications. I am looking forward to seeing the results.

I have read this submission. I believe that I have an appropriate level of expertise to confirm that it is of an acceptable scientific standard.

# Peer review: Referee response for version 1

**DOI:** 10.21956/wellcomeopenres.15917.r33379

**Published:** 2018-07-04

**Authors:** Filip Zemrak

**Affiliations:** 1 St. Bartholomew's Hospital, London, UK

This paper described the rationale, design and the protocol of a cross-sectional study of cardiovascular disease in Russia.

 Major comments

1. The sample size may not be large enough to describe phenotypes and their associations.
2. The variability of measurements (i.e. echo parameters, ECG) and the measurement techniques (multiple centres, various scales, sphygmometers etc) will be significant.
3. Even with a much more reproducible method (cardiac MRI) in MESA study, which had a similar sample size, the EF was not the best parameter to assess cardiac function/phenotype.

I have read this submission. I believe that I have an appropriate level of expertise to confirm that it is of an acceptable scientific standard, however I have significant reservations, as outlined above.

# Response

**Published:** 2018-07-04

**Authors:** Sarah Cook

**Affiliations:** London School of Hygiene & Tropical Medicine, UK

We thank Dr Filip Zemrak for his comments. Our response is as follows:

 1. One of the major objectives of this study is to make comparisons between the Know Your Heart Study in Russia and the Tromsø 7 Study, Norway. Dr Zemrak may wish to note that as shown in Table 2 of our paper there are 8-10 fold differences in risk of CVD and IHD death between the Russian and Norwegian populations. This is the background against which we are attempting to identify differences in CVD phenotypes and risk factors. It is the basis for our confidence that even our relatively small study (compared to for example UK Biobank or China Kadoorie Biobank) we will find large differences in some key factors as a result. This has been supported by early findings on interim data where we compared cardiovascular risk factors between the two studies. We found strong evidence (i.e. very low p-values) for a difference between the study populations on several of the key parameters of interest (e.g. blood pressure). These results were presented at Regional Health Research Conference, Norway (2016) and the Russian National Congress of Cardiology (2017). A full paper reporting these contrasts is in preparation now that all field work is completed.

 Given the nature of the study (breadth of measures collected) it is not feasible to provide sample size calculations for every possible research question of interest and the sample size calculations given within the paper are designed to cover a range of plausible relevant scenarios. Our paper is a study protocol describing the methodology of the study and providing key information about its conduct and recruitment of subjects. To this extent the imprecision of any effect estimates we subsequently report will be reflected in the confidence intervals. We agree with Dr Zemrak that for some things we will be underpowered, whether this is in comparisons with other studies such as the Tromsø study, or comparisons within the Russian study populations. However, for other things we will be more than adequately powered. We have acknowledged this limitation in the revised version of the paper.

 2. We acknowledge measurement error is a limitation as in any multi-site epidemiological study. As outlined in the paper several steps were taken to minimize variability including the production of detailed standard operating procedures, review of reports on data quality every month throughout the duration of the fieldwork and regular assessment of intra- and inter- reader variability in offline reading of ECHO and vascular ultrasound data. A strength of the study design (as described within the paper) is that in each city a further 200 participants were invited back one year after their initial health check and completed all study procedures for a second time.  From this data we have been able to calculate correction factors which assess the extent of within-person variability in measurements (which includes measurement error due to factors such as variation in observers and devices). From a further paper (currently under review) we can demonstrate our correction factors for a variety of measures are comparable (and generally lower) to those from a very large “gold standard” UK study providing additional reassurance on data validity. Given that this paper is a protocol paper it would not be appropriate to include these more substantive results concerning repeatability of measures. These will be published alongside results of specific analyses as they appear. 

 3. Ejection fraction is not the main or only functional parameter of interest in this study. Others are described in the protocol under details of the health check examination. We mentioned ejection fraction under our illustrative analysis plan as an example of a commonly used parameter within clinical practice which may be used alongside more agnostic methods such as factor analysis but it was not our intention to suggest this is the only parameter to be investigated. As we describe in the protocol paper we are undertaking intra- and inter-site reliability studies of off line reading. Unfortunately we did not have the resources to undertake cardiac MRI on 4500 Russian participants and equivalent number of Norwegian study subjects.

# Peer review: Referee response for version 1

**DOI:** 10.21956/wellcomeopenres.15917.r33258

**Published:** 2018-06-21

**Authors:** John Lynch

**Affiliations:** 1 School of Population Health, University of Adelaide, Adelaide, SA, Australia

This is a ‘model’ example of how a protocol paper should be written. The protocol is exceptionally clearly written and contains all the necessary details of the various study aspects including the study purpose, sampling, recruitment and the various dimensions of data collection, through to an outline of the analysis plan.

 It requires no revisions and can be published as it stands.

I have read this submission. I believe that I have an appropriate level of expertise to confirm that it is of an acceptable scientific standard.
